# Stress-induced recruitment of small heat shock protein 1 at the outer mitochondrial membrane underlies resistance to apoptosis

**DOI:** 10.1038/s41420-026-03268-8

**Published:** 2026-07-29

**Authors:** Ayesha Kiran Mendes, Stijn L. M. in ’t Groen, Vicky De Winter, Vincent Timmerman

**Affiliations:** Peripheral Neuropathy Research Group, Department of Biomedical Sciences, University of Antwerp, Antwerp, Belgium

**Keywords:** Chaperones, Stress and resilience, Apoptosis

## Abstract

Small Heat Shock Protein Family B (HSPB) members are ATP-independent chaperones essential for maintaining cellular proteostasis. Together with chaperones and co-chaperones such as HSP70 and BAG3, they prevent aberrant protein folding and suppress protein aggregation, particularly under proteotoxic stress. HSPBs have also been extensively linked to neurodegenerative disease and in promoting chemoresistance in cancer. We previously showed that members of the HSPB family can reside within the mitochondrial intermembrane space where they contribute to the maintenance of proteostasis, and that acute cytotoxic stress leads to HSPB sequestration to the outer mitochondrial membrane. Here, we further elucidate this mechanism, demonstrating that HSPB1 is selectively enriched within mitochondria under milder thermal stress condition, exhibiting a pronounced association with the outer mitochondrial membrane. We also observed distinct stress-dependent mitochondrial recruitment profiles across HSPB paralogs, revealing a similar behaviour for HSPB8, whereby HSPB1 responded at a comparatively lower stress threshold, indicating functional specialization within the HSPB family. Inducing this mechanism enhances cell survival during hyperthermic stress, preventing Cytochrome c release, and raising the apoptotic activation threshold in our experimental system. These findings indicate that HSPBs, particularly HSPB1 may contribute to mitochondrial stress adaptation prior to Cytochrome c release. This underexplored mechanism may underlie the modulation of stress-related and degenerative disease progression and contribute to the chemoresistance associated with cancer, which have been attributed to HSPBs.

## Introduction

Small Heat Shock Family B (HSPB) Proteins are ATP-independent chaperones, typically ranging from 15 to 40 kDa, that are integral to the maintenance of cellular proteostasis [1, 2]. In concert with other chaperons and co-chaperones such as HSP70 and BAG3, and other heat shock proteins, they prevent aberrant protein folding, suppress aggregate formation, and contribute to cell membrane stabilization, particularly under conditions of proteotoxic stress [1, 3–5]. Within the HSPB family, sub-specialization is evident, with some of the 10 protein members displaying ubiquitous expression, while others are restricted to specific tissues. HSPB1, HSPB2, HSPB5, HSPB6, HSPB7, and HSPB8 are broadly expressed across multiple cell types, whereas other HSPBs such as HSPB9 and HSPB10 exhibit more tissue-specific patterns reflecting their functional diversification [1]. Notably, high levels of HSPBs are found in post-mitotic tissues such as cardiac muscle, skeletal muscle, and the nervous system, underscoring their essential role in maintaining proteostasis in long-lived, terminally differentiated cells [1]. Consequently, dysregulation of HSPBs activity has been implicated as a contributing factor in the development and progression of numerous diseases, including neurodegenerative disorders, various forms of cancer, and metabolic syndromes [2, 3, 6–8].

Although primarily located in the cytosol, we previously demonstrated that certain members of the HSPB family can reside in the mitochondrial intermembrane space (IMS) under basal conditions and rapidly sequester to the outer mitochondrial membrane (OMM) under heat induced proteotoxic stress conditions (≥42 °C for 1 h) [9]. However, the precise functional implications and molecular triggers underlying this sequestration remain to be elucidated.

In the present study, we sought to clarify the biological relevance and underlying mechanism of this stress-induced mitochondrial re-localization of the HSPBs. We hypothesized that this stress induced immediate sequestration of HSPBs to the mitochondria could be a targeted protective response aimed at conferring cytoprotective effects during acute proteotoxic stress. To investigate this, we exposed cells to milder stress conditions and observed that HSPB1 alone rapidly translocates to the mitochondria, and specifically at the OMM whereas other HSPBs, such as HSPB8 (Supplementary Fig. 1), exhibit this behaviour only under more severe conditions. We demonstrate that this targeted mitochondrial sequestration of HSPB1 is critical in maintaining OMM integrity, delaying the mitochondrial outer membrane permeabilization (MOMP) initiation and subsequent apoptotic signaling events. This study highlights a mechanism whereby stress induced mitochondrial enrichment of HSPB1, likely at OMM confers cytoprotection prior to cytochrome c (Cytc) release. This mechamsism may thereby influence the progression of HSPBs-related disorders and degenerative diseases and may also help explaining the link between elevated HSPBs expression and chemoresistance in cancer [8, 10–13].

## Results

### HSPB1 sequesters at the outer mitochondrial membrane in response to mild thermal stress

**Fig. 1:**
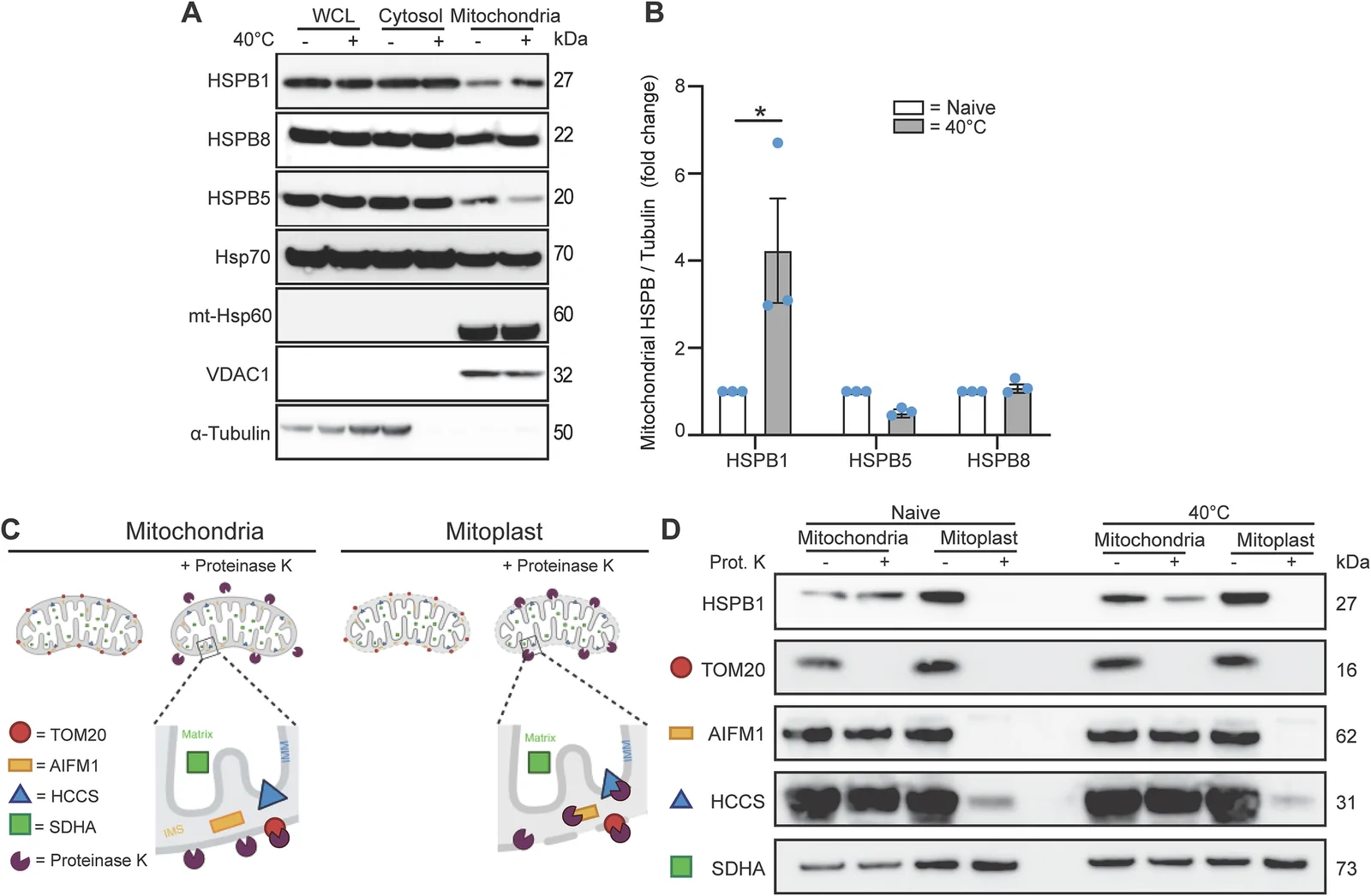
**A** Representative western blot of HSPB1, HSPB8, HSPB5, Hsp70, mt-Hsp60, VDAC1, and α-tubulin in the whole-cell lysate (WCL), cytosolic, and mitochondrial protein fractions with and without preconditioning with 40 °C. **B** Quantification of **A**, showing HSPB1, HSPB5, and HSPB8. **C** Graphical illustration of protein localization and accessibility to proteinase K within the mitochondrial and mitoplast protein fractions (reference 9). **D** Western blot showing HSPB1 alongside protein localization (**C**) following the proteinase K assay. Comparing protein samples derived from naïve and heat-treated cells. Statistical significance throughout this figure was determined using one-way ANOVA followed by Sidak’s post hoc multiple comparison test. ns = no significance, * = *p* < 0.05.

We have previously shown that a heat stress regiment of 42 °C (hence referred as heat shock) facilitates the translocation of HSPB1, HSPB5 and HSPB8 in cell models (Supplementary Fig. 1) towards the OMM [9]. However, such exposure simultaneously incites potential adverse effects, including activation of endoplasmic reticulum stress [14, 15] and perturbations in calcium homeostasis [16, 17], ultimately compromising cellular viability [18, 19]. Hence, our initial aim was to ascertain whether we could elicit a similar stress response from HSPBs under less extreme conditions. Therefore, we applied a 1-h heat stress at 40 °C (hereafter referred to as mild thermal stress), a well-established threshold that activates the heat shock response while compromising cellular viability only minimally and avoiding the confounding effects of higher temperatures [18, 19]. After exposure to this mild thermal stress regimen, mitochondrial fractions were prepared for Western blot analysis. Unlike our previous study [9], only HSPB1 exhibited a significant increment within the mitochondrial fraction of HeLa cells, while HSPB5 and HSPB8, alongside other molecular chaperones like mtHSP60 or HSP70, showed no such accretion (Fig. 1A, B). Similar findings were observed in human induced pluripotent stem cell (hiPSC)-derived myotubes and differentiated SH-SY5Y neurons, with HSPB1, but not HSPB5 or HSPB8, translocating to mitochondria upon exposure to mild thermal stress (Supplementary Fig. 2). In contrast, mitochondrial HSPB1 was not detected in N2a neuroblasts. However, low basal HSPB1 expression, also observed in the whole-cell lysates and cytosolic fractions and consistent with previous reports [9], may have obscured these findings.

To verify the sub-compartmental localization of HSPB1, mitochondrial and mitoplast fractions were treated with proteinase K, allowing assessment of the protein’s accessibility within distinct mitochondrial compartments (Fig. 1C). Consistent with our earlier findings [9], under physiological conditions, mitochondrial HSPB1 was only subject to digestion upon the rupture of the outer membrane, paralleling AIFM1 (Apoptosis-inducing factor, mitochondrion-associated) and thereby affirming its residency in the IMS, rather than the mitochondrial matrix and the inner membrane marked by HCCS (Holocytochrome C Synthase) and SDHA, (Succinate Dehydrogenase Complex Subunit A) respectively [9]. Whereas following the exposure to mild thermal stress, a larger fraction of HSPB1 became vulnerable to digestion in intact mitochondria, similar to TOM20 (Translocase of the outer mitochondrial membrane 20), confirming its translocation to the OMM (Fig. 1D). These results indicate that the mitochondrial fraction of HSPB1 behaves similarly under mild thermal stress, as previously observed at 42 °C in our earlier study [9].

To elucidate the mechanism underlying HSPB1 translocation to the OMM, we focused on the mitochondrial receptor TOM70, a well-characterized component of the TOM complex involved in recruiting cytosolic chaperones at the OMM, especially under stress [20]. Using previously generated HeLa cells expressing HSPB1-V5 (Table 1), we performed co-immunoprecipitation and observed a stress-dependent TOM70-HSPB1 interaction that was absent under basal conditions (Supplementary Fig. 3). These results suggest that TOM70 may facilitate HSPB1 recruitment to the OMM in response to cellular stress.

**Table 1:** Overview of the cell lines used in this study.

Cell line	Description	Origin
HeLa (Control)	-	
HSPB1-V5	HeLa (control) + lentiviral integration of *HSPB1-V5tag* expression construct	Adriaenssens et al. [9]
HSPB1^-/-^	CRISPR generated knockout of HSPB1	This Study
mtHSPBs^Depleted^	CRISPR generated knockout of HSPB1 & HSPB5 + shRNA knockdown of HSPB8	Adriaenssens et al. [9]
*HSPB1* overexpression	mtHSPBs^Depleted^ + lentiviral integration of *HSPB1* cDNA expression construct	This study
*HSPB8* overexpression	mtHSPBs^Depleted^ + lentiviral integration of *HSPB8* cDNA expression construct	This study
*HSPB3* overexpression	mtHSPBs^Depleted^ + lentiviral integration of *HSPB3* cDNA expression construct	This study

### HSPBs sequestration at the OMM modulates mitochondrial integrity

Numerous studies have proved that HSPBs, including HSPB1, can interact with lipid membranes and influence their physical state [4, 5, 21, 22], leading us to hypothesize that such interactions may modulate MOMP in response to stressors [23]. This, in turn, could delay the release of pro-apoptotic factors such as Cytc into the cytosol, loss of mitochondrial membrane potential (Ψm), and the subsequent activation of the intrinsic apoptotic pathway. In this study, we utilized oxidative stress to elicit MOMP through the application of 25–125 µM hydrogen peroxide (H₂O₂) for 1 h (Supplementary Fig. 4)

**Fig. 2:**
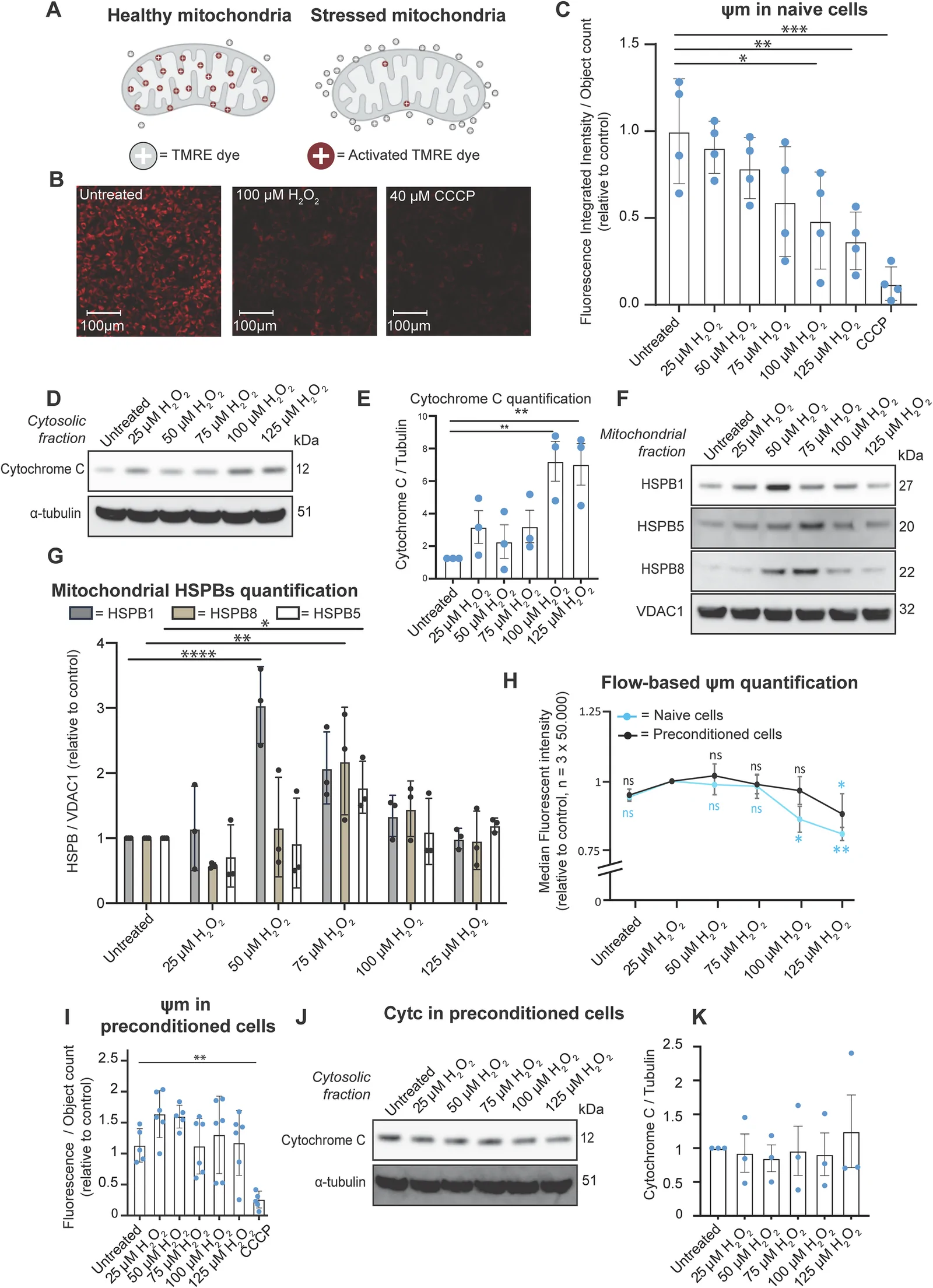
**A** Graphical illustration of the TMRE dye in healthy and stressed mitochondria. **B** Representative images of the fluorescent signal of TMRE in HeLa cells. **C** Quantification of TMRE fluorescence signal in naïve cells following the induction of oxidative stress with H_2_O_2_ or CCCP as control. Data represent mean ± SD (*n* = 4 independent experiments). **D** Representative western blot of Cytc and α-tubulin in the cytosolic protein fraction of naïve cells following H_2_O_2_ supplementation. **E** Quantification of Fig. 1D. Data represent mean ± SEM (*n* = 4 independent experiments). Representative western blot of HSPB1, HSPB8, HSPB5, and VDAC1 in the mitochondrial protein fraction following H_2_O_2_ supplementation. **G** Quantification of Fig. 1. **F** Data represents mean ± SEM (*n* = 3 independent experiments). Quantification of the flow cytometry-based analyses of the TMRE fluorescence signal in combination with H_2_O_2_ supplementation. Data represent the geometric mean of ≥50,000 cells per experiment ± SD (*n* = 3 independent experiments). **I** Quantification of TMRE fluorescence signal in preconditioned cells following the induction of oxidative stress with H_2_O_2_ or CCCP as control. Data represent mean ± SD (*n* = 6 independent experiments). **J** Same as Fig. 1D, but for preconditioned cells. **K** Quantification of Fig. 1J; data represent mean ± SEM (*n* = 4 independent experiments). Statistical significance throughout this figure was determined relative to the appropriate control sample using either one-way ANOVA followed by Sidak’s post hoc multiple comparison test (**C**, **E**, **G**, **I**) or two-way ANOVA followed by Tukey’s post hoc multiple comparison (**H**). For this figure significance is shown relative to the mildest treatment, which served as vehicle control. ns = no significance, * = *p* < 0.05, ** = *p* < 0.01, *** = *p* < 0.001, **** = *p* < 0.0001.

We first examined the H₂O₂ concentrations required to induce MOMP, as reflected by Cytc release and the accompanying loss of Ψm [24, 25]. Utilizing Tetramethylrhodamine (TMRE) to measure Ψm [26], we observed a significant dose-dependent decline in Ψm beginning at 100 µM H₂O₂ (Fig. 2A–C). Consistent with this, western blot analyses revealed increased levels of cytosolic Cytc (Fig. 2D, E) at the same concentrations, suggesting a marked loss of mitochondrial integrity.

Next, we sought to investigate how oxidative stress drives the recruitment of HSPBs towards mitochondria. Western blot analysis of the mitochondrial fraction obtained from H₂O₂-treated cells showed that the localization of HSPB1 in the mitochondria peaked at 50 µM H₂O₂, whereas HSPB5 and HSPB8 demonstrated maximal association at 75 µM H₂O₂ (Fig. 2F, G). This recapitulates the distinct HSPBs sequestration pattern observed earlier in response to thermal stress, wherein HSPB1 translocation to the OMM necessitates a lower threshold compared to HSPB5 and HSPB8. From 100 µM H₂O₂ onwards, the levels of HSPBs within mitochondria decreased, this coincided with the loss of Ψm and Cytc release, consistent with the onset of MOMP.

To investigate whether HSPB1 contributes to membrane stabilization at the OMM, consistent with its reported effects on other lipid bilayers [4, 5, 21, 22], we examined whether prior sequestration of HSPB1 to the OMM modulates mitochondrial susceptibility to stress. For this, a regiment of mild thermal stress (1 h at 40 °C, hereafter referred to as preconditioning) was used to induce HSPB1-recruitment to the OMM, whereafter the cells were exposed to increasing levels of oxidative stress. Using flow cytometry, TMRE-fluorescence was quantified, again revealing a marked reduction in Ψm beginning at 100 µM H₂O₂, in line with previous results (Fig. 2H). In contrast to naïve cells, preconditioned cells maintained TMRE signal up to 125 µM H₂O₂, indicating a clear shift in the depolarization threshold. Live-cell fluorescence imaging further confirmed this preservation of Ψm, demonstrating no detectable reduction in TMRE intensity (Fig. 2I). Consistent with the findings in HeLa cells, the preconditioning regimen preserved mitochondrial membrane potential (Ψm) in myotubes, SH-SY5Y neurons, and N2a neuroblasts. Live-cell imaging analysis of TMRE fluorescence revealed a reduction in signal at 125, 100, and 100 µM H₂O₂ in naïve myotubes, SH-SY5Y neurons, and N2a neuroblasts, respectively. In contrast, preconditioned myotubes and SH-SY5Y neurons maintained stable Ψm across all tested concentrations, whereas in N2a neuroblasts the threshold for Ψm loss was shifted to 125 µM H₂O₂ (Supplementary Fig. 5). Finally, preconditioned HeLa cells showed no detectable cytosolic release of Cytc under oxidative stress, as assessed by western blot (Fig. 2J, K). Together, these findings suggest that HSPB1 localisation at the OMM enhances mitochondrial resistance to oxidative-stress-induced depolarization, thereby raising the threshold for MOMP initiation.

To confirm our hypothesis that the onset of MOMP can be modulated through the translocation of HSPB1 at the OMM, we had to account for compensatory influences since our prior work showed that individual depletion of HSPB1, HSPB5, or HSPB8 does not alter basal mitochondrial morphology within HeLa cells [9]. To investigate this compensation more directly, we performed gene-expression analyses of *HSPB8* in the absence of *HSPB1* in *HSPB1*^*−/−*^ cell lines (Supplementary Fig. 6) under heat shock [9]. Indeed, RT-qPCR revealed that loss of *HSPB1* is compensated by a two-fold increase in *HSPB8* gene expression and a shift in its expression dynamics, with *HSPB8* responding more rapidly to heat stress in *HSPB1*^−/−^ cells compared to the unedited control. Moreover, to verify that HSPB8 can functionally compensate for the loss of HSPB1 at the OMM, we repeated the mitochondrial sub-compartmentalization assessment using proteinase K treatment. We observed that, unlike at 40 °C, exposure to 42 °C results in recruitment of HSPB8 to mitochondria [9] and the OMM specifically (Supplementary Fig. 7). This suggests that HSPB8 has the capability to functionally compensate for loss HSPB1 at the OMM.

**Fig. 3:**
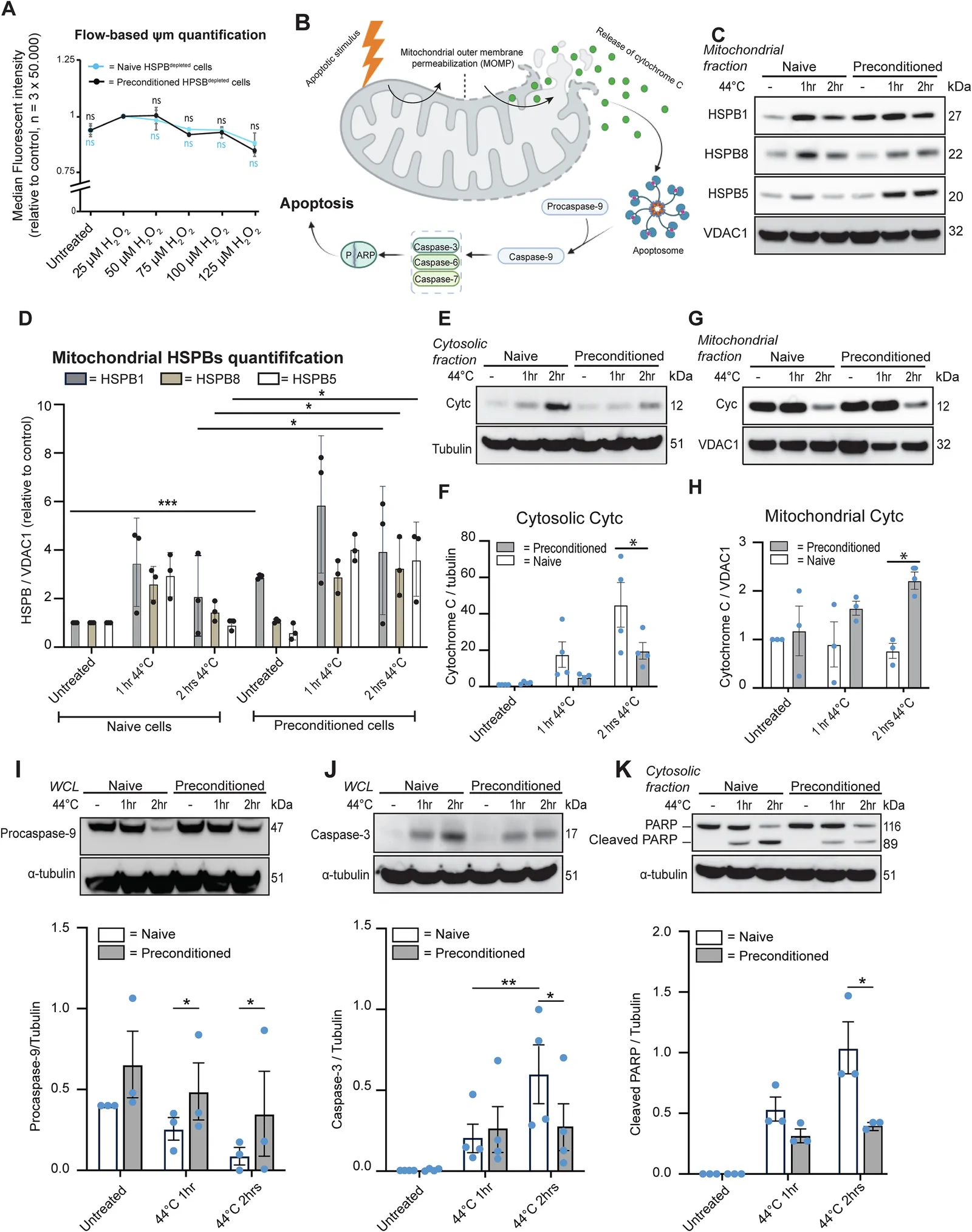
**A** Quantification of the flow cytometry-based analyses of TMRE fluorescence signal in combination with H_2_O_2_ supplementation in *HSPB*^*depleted*^ cells. Data represents the geometric mean of ≥50,000 cells per experiment ± SD (*n* = 3 independent experiments). **B** Graphical illustration of apoptotic signaling cascade upon induction of cytotoxic stress. **C** Western blot of the mitochondrial protein fraction from naïve and preconditioned HeLa cells, showing the effects of lethal heat stress on the expression of HSPB1, HSPB5, HSPB8, and VDAC1. **D** Quantification of mitochondrial HSPBs levels (**C**). Data represent mean ± SEM (*n* = 3 independent experiments). **E** Western blot of the cytosolic protein fraction from naïve and preconditioned cells, showing the effects of lethal heat stress on Cytosolic Cytc levels. **F** Quantification of cytosolic Cytc levels (**E**). Data represent mean ± SEM (*n* = 4 independent experiments). **G** Same as (**E**), but showing Cytc levels in the mitochondrial fraction. **H** Quantification of mitochondrial Cytc levels (**F**). Data represent mean ± SEM (*n* = 3 independent experiments). **I** Representative western blot (top) and corresponding quantification (below) of WCL from naïve and preconditioned cells, showing procaspase-9 levels after one or two hours of lethal heat stress. Data represent mean ± SD (*n* = 4 independent experiments). **J** Same as (**I**), showing caspase-3 levels. **K** Same as (**I**), showing cleaved and uncleaved PARP in the cytosolic protein fraction. Statistical significance throughout this figure was determined relative to the appropriate control sample using either one-way ANOVA followed by Sidak’s post hoc multiple comparison test (**F**–**K**) or two-way ANOVA followed by Tukey’s post hoc multiple comparison (**A**). For this figure significance is shown relative to the mildest treatment, which served as vehicle control. ns = no significance, * = *p* < 0.05, **** = *p* < 0.0001.

To address this possibility, we used a previously established cell line wherein the expression of all predominant mitochondrial HSPB isoforms expressed in HeLa cells (HSPB1, HSPB5, and HSPB8) was abrogated, thereby preventing any confounding impacts of compensatory mechanisms observed in single-gene knockout models (*HSPB*^*depleted*^, Table 1 and Supplementary Fig. 5) [9]. In contrast to the results observed in unmodified HeLa cells, preconditioning did not prevent the decrease in Ψm following exposure to oxidative stress, as measured by flow cytometry (Fig. 3A). This lack of protection in *HSPB*^*depleted*^ also excludes contributions from other major heat-shock chaperones, such as HSP60, HSP70, and HSP90, indicating that the protective effect requires HSPBs themselves.

### HSPBs translocation to the OMM abrogates intrinsic apoptotic pathway

MOMP is a key event in apoptosis during which the OMM loses its integrity allowing the release of pro-apoptotic factors into the cytosol, such as Cytc, apoptosis inducing factor (AIF), and Endonuclease G, inducing downstream apoptotic signalling pathways. Cytc release triggers activation of caspase-9, executioner caspase-3, and ultimately Poly (ADP-ribose) polymerase (PARP) cleavage [27], eventually triggering cell death (Fig. 3B).

Next, we examined if this protective effect could be extended to the downstream intrinsic apoptotic events. To this end, cells were subjected to the preconditioning regimen, followed by a 30-min recovery period and a subsequent burst of one or two hours of hyperthermic stress (44 °C). We then examined the sequestration of HSPBs at the OMM in parallel with cytochrome c release and the ensuing activation of apoptotic signalling using western blot analysis.

Focusing on HSPBs sequestration to the OMM, we found HSPB1 to be exclusively enriched after the preconditioning regiment (Fig. 3C, D), consistent with its recruitment during the initial mild-stress phase. Following exposure to hyperthermic stress for 1 h, mitochondrial levels of HSPB1, HSPB5, and HSPB8 increased significantly in both naïve and preconditioned cells (Fig. 3C, D). When hyperthermic stress was prolonged to two hours, mitochondrial HSPB levels remained elevated in preconditioned cells but declined in naïve cells (Fig. 3C, D). In terms of Cytc dynamics, naïve cells displayed 20-fold and 40-fold increases in cytosolic Cytc levels after one or two hours, respectively (Fig. 3E, F). Comparatively, preconditioned cells showed only 6-fold and 20-fold increases at the same time points (Fig. 3E, F). Conversely, loss of mitochondrial Cytc occurred only in the naïve cells, corresponding with the cytosolic increase (Fig. 3G, H). This increased Cytc release in naïve cells after two hours of hyperthermic stress is accompanied by a marked decrease in procaspase-9, elevated caspase-3 levels, and PARP cleavage (Fig. 3I–K). In contrast, preconditioned cells displayed only minimal to negligible activation of these intrinsic apoptotic events (Fig. 3I–K). These findings indicate that exposure to mild stress confers a cytoprotective phenotype that preserves mitochondrial integrity, inhibiting the release of Cytc into the cytosol, effectively preventing the downstream activation intrinsic apoptotic signalling and promoting cell survival.

### HSPB1 sequestration at the OMM prevents intrinsic apoptosis under hyperthermic stress

**Fig. 4:**
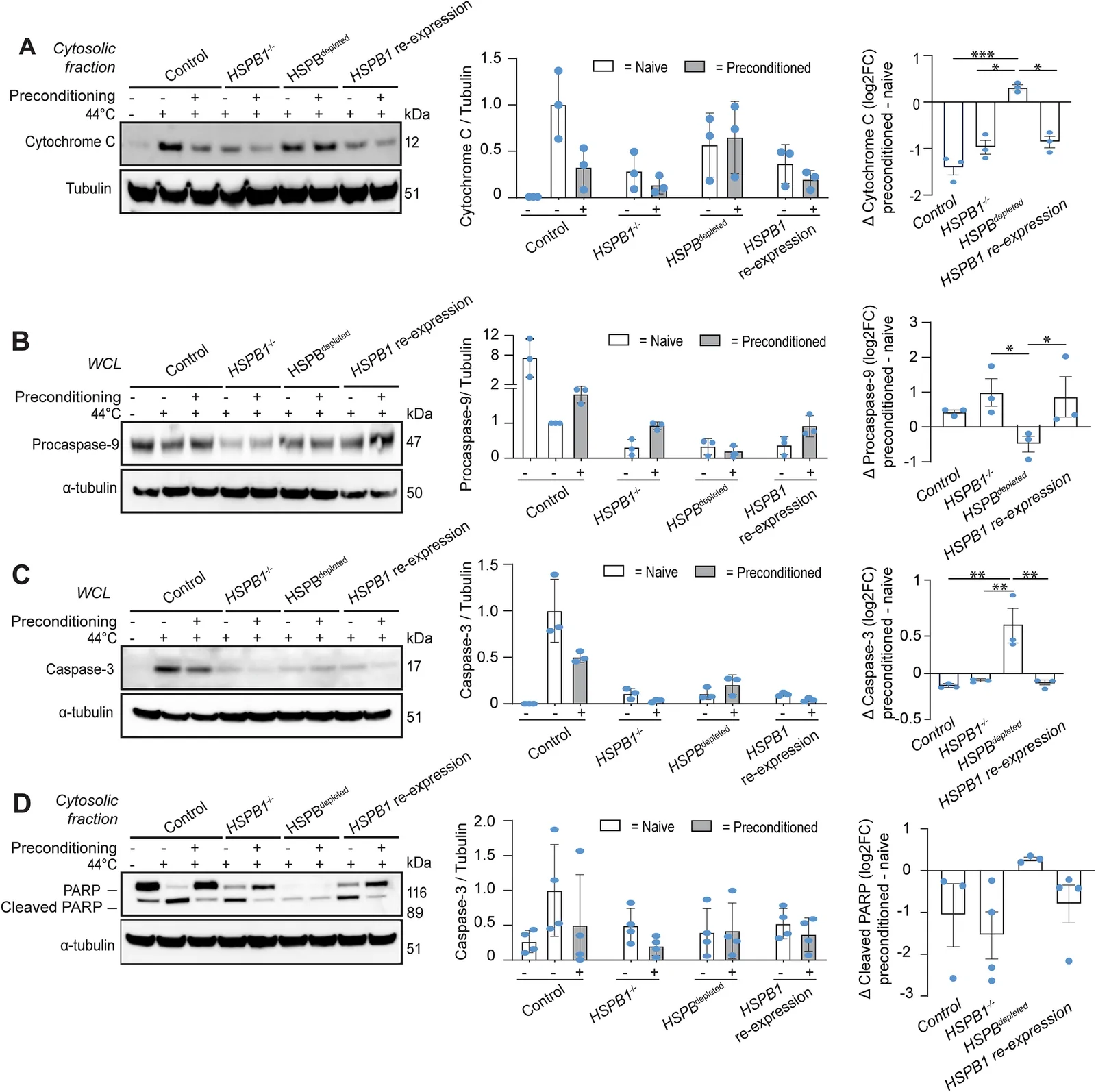
**A** Western blot and corresponding quantification of the cytosolic protein fraction of control, HSPB1⁻^/^⁻, HSPB1-depleted, and HSPB1-overexpressing cells, showing the effects of lethal heat stress on cytosolic Cytc levels in naïve and preconditioned conditions. The quantification depicts the relative protein levels in all conditions (middle) and the difference in Cytc levels between the naïve and preconditioned cells for each cell line (right). Data represent mean ± SD (*n* = 3 independent experiments). **B** Same as (**A**), showing procaspase-9 levels and quantification in WCL. **C** Same as (**A**), showing caspase-3 levels and quantification in WCL. **D** Same as (**A**), showing cleaved and uncleaved PARP in the cytosolic protein fractions. Statistical significance throughout this figure was determined relative to the appropriate control sample using either one-way ANOVA followed by Sidak’s post hoc multiple comparison test. * = *p* < 0.05, ** = *p* < 0.01, *** = *p* < 0.001.

To determine whether the observed cytoprotective effect is governed by HSPB1 exclusively, we performed analogous experiments using *HSPB1*^*−/−*^ cells, *HSPB*^*depleted*^ cells, and an *HSPB1*-overexpression cell line in the *HSPB*^*depleted*^ background (Supplementary Fig. 8A), wherein HSPB1 is the only small heat shock protein able to reside to the mitochondria (Table 1). We found that the loss of HSPB1 alone is insufficient to abolish the cytoprotective effect of the preconditioning regimen. However, upon abrogation of all mitochondrial HSPBs, cells exhibited increased vulnerability and a complete loss of the beneficial response (Fig. 4A–D). Unlike *HSPB1*^-/-^ cells, *HSPB*^*depleted*^ cells exhibited no beneficial effect from the preconditioning regimen, exhibiting a pronounced increase in cytosolic Cytc in response to hyperthermic cell stress (2 h at 44 °C) even after preconditioning (Fig. 4A). This was accompanied by a reduction in procaspase-9, an increase in caspase-3, and a non-substantial increase in cleaved PARP (Fig. 4B–D). In contrast, re-expression of *HSPB1* restored the cytoprotective phenotype (Fig. 4A–D), showing that this impact is mediated by mitochondrial HSPBs and can be conferred solely by HSPB1.

### Mitochondrial targeting HSPBs at OMM can prevent intrinsic apoptosis under hyperthermic stress

**Fig. 5:**
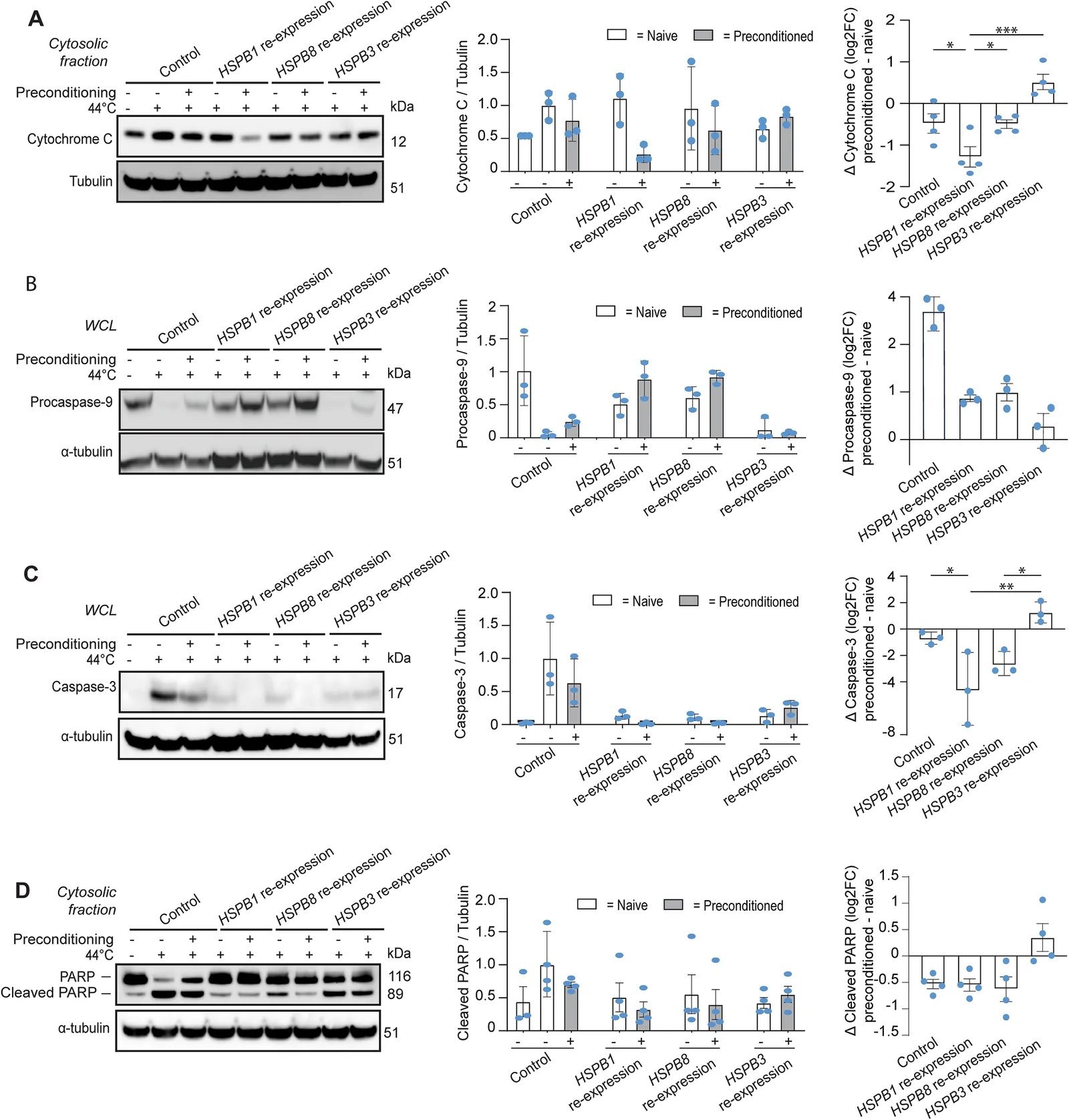
**A** Western blot and corresponding quantification of the cytosolic protein fraction of control, HSPB1, HSPB8, and HSPB3-overexpressing cells, showing the effects of lethal heat stress on cytosolic Cytc levels in naïve and preconditioned conditions. The quantification depicts the relative protein levels in all conditions (middle) and the difference in Cytc levels between the naïve and preconditioned cells for each cell line (right). Data represent mean ± SD (*n* = 3 independent experiments). **B** Same as (**A**), showing procaspase-9 levels and quantification in WCL. **C** Same as (**A**), showing caspase-3 levels and quantification in WCL. **D** Same as (**A**), showing cleaved and uncleaved PARP in the cytosolic protein fractions. Statistical significance throughout this figure was determined relative to the appropriate control sample using either one-way ANOVA followed by Sidak’s post hoc multiple comparison test. * = *p* < 0.05, ** = *p* < 0.01, *** = *p* < 0.001.

Having established that HSPB1 can enhance OMM stability, thereby preventing activation intrinsic apoptotic signalling, and considering the compensatory upregulation of HSPB8, we sought to evaluate the cytoprotective potential of HSPB8 at the OMM. To this end, we generated re-expression HeLa cell lines for HSPB8 and HSPB3 in the HSPB^depleted^ background, using the HSPB3-expressing line as a non-mitochondrial control, since not all HSPB family members translocate to mitochondria (Supplementary Fig. 8B) [9]. As expected, in HSPB1- and HSPB8-expressing preconditioned cells, the release of Cytc into the cytosol was effectively prevented (Fig. 5A), and downstream intrinsic apoptotic signalling was inhibited (Fig. 5B–D). In contrast, HSPB3-expressing cells exhibited Cytc release and activation of apoptotic markers similar to HSPBs-depleted cells (Fig. 5A–D). These results indicate that only HSPBs capable of mitochondrial localization are recruited by preconditioning to prevent Cytc release and subsequent activation of the apoptotic cascade. These results indicate that only HSPBs capable of mitochondrial localization are recruited by preconditioning to prevent Cytc release and subsequent activation of the apoptotic cascade.

Collectively, our study significantly advances the understanding of apoptosis regulation, highlighting stress induced OMM localization of HSPBs, most notably HSPB1 [9] and HSPB8, as a central mechanism of cellular resilience. These findings highlight HSPB-mediated OMM stabilization as a critical therapeutic target, with strong potential for preserving cell viability in disease driven by excessive apoptosis, including neurodegeneration and ischemia [28]. Moreover, the same mechanism may also underlie the HSPBs-driven chemoresistance in cancer [8, 11–13, 29–31].

## Discussion

In the previous study, we showed that HSPBs can reside within the IMS where they contribute to proteostasis, and that their mitochondrial abundance increases following heat shock [9]. Notably, these same HSPBs, particularly HSPB1 and HSPB8, have also been reported to exert a cytoprotective role against apoptotic stimuli [32–36], although the precise mechanism is only partially understood [32, 33, 37]. Considering that HSPBs can associate with lipid membranes under stress conditions [4, 5, 21, 22], we hypothesized that induced translocation of HSPB1 at the OMM could be a missing link in their cytoprotective function, acting upstream of Cytc release to prevent apoptosis.

In this study, we first examined HSPBs mitochondrial translocation under mild heat stress, and observed that mainly HSPB1 exhibited increased mitochondrial presence, likely at the OMM, unlike the broad mitochondrial recruitment reported at the conventional 42 °C threshold [9]. We have also indicated that TOM70, a well-characterized receptor for the import of cytosolic chaperones to the OMM [20], may play a role in the selective recruitment of HSPB1; however, additional experiments employing TOM70 knockdown or knockout cell lines are required to further corroborate this.

Next, we investigated the impact of oxidative stress through treatment with 25 to 125 µM H_2_O_2_ and observed a concentration-dependent and transient recruitment of HSPB1, HSPB5, and HSPB8 to the mitochondria, peaking at intermediate levels of stress and declining at higher concentrations of H_2_O_2_. The point of decline in mitochondrial HSPBs localization correlated with the onset of detectable mitochondrial dysfunction, marked by loss of the mitochondrial membrane potential (TMRE signal) and Cytc release. This suggests that the mitochondrial recruitment of HSPBs may represent an early cytoprotective response potentially becoming overwhelmed or saturated during severe stress conditions.

Members of the HSPB protein family have been increasingly recognized for their potential role in membrane stabilization during cellular stress [4, 21, 22], with evidence suggesting that HSPB1 and HSPB5 can interact with lipid bilayers, as shown with artificial and bacterial lipid membranes (including monolayers and liposomes), and influence membrane integrity and fluidity [4, 5]. We therefore hypothesized that the cytoprotective role of HSPBs may be mediated through their localization to the OMM, whereby HSPBs increase the stability of the OMM against MOMP-inducing conditions. Indeed, our data suggest that a mild heat-stress preconditioning regiment to sequester HSPB1 on the OMM was sufficient in effectively blocking MOMP. This prevents the release of Cytc and the subsequent intrinsic apoptotic cascade, i.e. activation of caspase-9, caspase-3, and the irreversible cleavage of PARP. This sequestration of HSPB1 to the OMM could induce a biological response known as thermotolerance, which refers to the enhanced ability of HeLa cells to withstand subsequent stress following an initial mild thermal stress exposure. The protective effects we observed not only corroborate earlier studies [14, 38] that highlight the general roles of HSPBs in promoting stress tolerance, but also significantly expand our understanding by specifically addressing mitochondrial stress responses mediated by HSPBs.

The observation that sequestration of HSPB1 precedes that of HSPB5 and HSPB8 suggests functional differentiation among HSPB paralogues in stress sensing and membrane engagement, consistent with earlier studies showing HSPB1’s preferential association with fluid, disordered membrane phases [4, 5]. This interpretation is further supported by the ability of other mitochondria-localising HSPBs, notably HSPB8, to compensate for HSPB1 loss, consistent with the genetic compensation and functional redundancy observed in many gene families. This redundancy aligns with prior in vivo studies showing that HSPB1 and HSPB8 are dispensable under basal conditions but become essential during stress or pathological mutation [39–41]. Conversely, the dramatic apoptotic vulnerability in HSPBs-depleted cells underscores the necessity of mitochondrial-targeted HSPBs as a critical protective ensemble. Importantly, rescue experiments involving the re-expression of individual HSPBs demonstrated that the cytoprotective effect is exclusively dependent upon their localization at the OMM, as again exemplified by the inability of cytosolic-only HSPB3 [9] to prevent the release of Cytc.

Mitochondrial dysfunction serves as a fundamental basis for a diverse range of pathologies, including ischemia, neurodegenerative diseases, oncogenesis, and metabolic syndromes [42, 43]. Our results suggest that HSPBs, particularly when recruited to the OMM, can form a dynamic network that preserves mitochondrial integrity. In therapeutic terms, enhancing HSPB1 recruitment or mimicking its membrane-binding motifs could offer cytoprotection in mitochondria-associated degenerative diseases. Conversely, targeted disruption of HSPB1 localization or stability may promote cell clearance. Understanding the regulatory axis controlling HSPBs mitochondrial recruitment may thus unlock novel interventional pathways across both degenerative and malignant contexts. This newly identified mechanism takes on a particular relevance in oncology, wherein HSPB1 overexpression is implicated in chemoresistance and promotes cancer stem cell traits [8, 11–13, 27, 29, 31]. The observation that OMM-sequestered HSPBs can impede apoptosis offers new mechanistic insights into this phenomenon and may open a novel avenue for enhancing the therapeutic efficacy of long-term cancer treatments.

Further investigative studies are needed to fully elucidate the mode of action of OMM-sequestered HSPBs. Considering the broad functional roles of HSPBs, it remains to be determined whether their cytoprotective function primarily relies on physical stabilization of the OMM [4, 5, 44], or is modulated through direct interactions with apoptotic signaling pathways [45, 46] and mitochondrial regulatory components^48,49,50^. Despite the robustness of our findings, several limitations merit consideration. Foremost, our mechanistic experiments were conducted exclusively in vitro using transgenic HeLa cells and artificial stressors, raising questions about the physiological relevance of HSPBs recruitment. While the OMM sequestration of HSPBs has been confirmed in other cell models, HeLa cells lack endogenous expression of Parkin (*PRKN*), which could influence the observed phenotypes in the transgenic HSPB knockout and overexpression lines. Parkin is a key regulator at the interface of mitochondrial quality control and apoptosis, where it modulates OMM ubiquitination and can suppress apoptotic activation in response to mitochondrial damage [47]. The lack of Parkin therefore implies that the observed HSPB recruitment likely occurs independently of canonical Parkin-dependent pathways, potentially omitting key regulatory checkpoints.

Furthermore, the evident functional redundancy seen in *HSPB1*^*-/-*^ cells under stress indicates potential compensation by HSPB8. However, it is not impossible that other chaperones, particularly those belonging to the HSP70 family [48], might also play a role in this. Indeed, this remains speculative in the absence of transcriptomic or proteomic profiling under stress conditions, which could clarify the extent of compensatory chaperone upregulation. Our results open several exciting avenues for future research. Firstly, structural studies using advanced methodologies (e.g., proximity labelling or super-regulation microscopy) are necessary to delineate the precise molecular interactions between HSPB1 and mitochondrial membranes. Secondly, in vivo experiments using animal models of oxidative stress and apoptosis would be essential for validating the physiological relevance of our observations. Finally, investigating whether HSPB1 and HSPB8 mutants associated with clinical neuropathies (such as Charcot-Marie-Tooth disease) [10] affect mitochondrial protective functions could offer direct clinical relevance.

In conclusion, the broadening profile of HSPBs function positions them as compelling targets for further investigation. In the present study, we elucidated on a mechanism wherein HSPB1 assumes a cytoprotective role by associating with the OMM in response to mild cytotoxic stress, thereby broadening our conceptual framework regarding mitochondrial stress responses and apoptosis. These findings not only position HSPBs as pivotal regulators of cell survival but also highlight their therapeutic potential: enhancing the translocation of HSPBs to the OMM could protect against neurodegenerative disorders, while suppressing this activity may help overcome HSPBs associated chemoresistance in cancer.

## Material and methods

### Cell lines and cell culture

HeLa cell lines were acquired from the American Type Culture Collection (ATCC) and were grown in MEM medium supplemented with 10% fetal bovine serum, 1% l-glutamine, and 1% penicillin–streptomycin (Life Technologies) at 37 °C and 5% CO₂. HeLa cells were selected for this study because their HSPBs expression profile, predominantly HSPB1, HSPB5, and HSPB8, is well-defined, making them a suitable model for dissecting isoform-specific mitochondrial functions.

Neuro-2A neuronal crest–derived cells and SH-SY5Y neuroblastoma cells were cultured in MEM medium supplemented with 10% FBS, 1% L-glutamine, 1% non-essential amino acids, and 1% penicillin–streptomycin. Neuro-2A cells were maintained in a proliferative state, while SH-SY5Y cells were analyzed following an eight-day differentiation protocol to induce a motor neuron–like phenotype. Differentiation was carried out using neurobasal medium supplemented with 2% FBS, 1% B-27, + 10 µM retinoic acid, and 50 ng/mL BDNF.

iPSC-derived skeletal muscle cells were generated from myogenic [49]. Myogenic progenitors were maintained in DMEM supplemented with 10% fetal bovine serum (FBS), 100 ng/ml bFGF, 1% L-glutamine, and 1% penicillin–streptomycin. Myotube differentiation was induced by switching to DMEM supplemented with 1% knockout serum replacement and 1% insulin-transferrin-selenium-ethanolamine supplement. For the preconditioning, cells were incubated at 40 °C for 1 h. For experiments involving hyperthermia, this regiment was followed by a 30-min recovery period at 37 °C prior to incubation at 44 °C for 1–2 h.

### Plasmid origin and vector construction

For each of the vectors, we cloned complementary DNA sequences into the respective plasmid backbones: p3xFLAG-CMV-14 (Sigma-Aldrich), pDONR221 and pLenti6/V5 (Life Technologies). Untagged sHSPs, HSPB1, HSPB3, HSPB5, and HSPB8 (Frt-HSPBx, Addgene plasmids #63092–#63101, Addgene) were a kind gift from Harm Kampinga (University of Groningen). shRNAs of the Sigma Mission pLKO.1-library were obtained from the BCCM/LMBP Plasmid collection (https://bccm.belspo.be). We used shRNAs against HSPB8 (home-made 3′ untranslated region). Open reading frames were subcloned using restriction enzymes (FastDigest, Thermo Fisher Scientific) and Gateway cloning (Thermo Fisher Scientific). All polymerase chain reaction (PCR) amplification reactions were performed with the Kapa HiFi HotStart PCR kit (07958897001, Roche Diagnostics Belgium). For Gateway cloning, we followed the company’s protocol using BP- and LR-reaction enzymes (11789020 and 11791020, Thermo Fisher Scientific, Waltham) and pDONR221 and pLENTI6/V5 vectors (Thermo Fisher Scientific).

### Generation of stable cells through lentiviral transduction

Stable cell lines were generated by lentiviral transduction. Lentiviral vectors were used containing the open reading frames of HSPB1 (NM_001540), HSPB3 (NM_006308) and HSPB8 (NM_014365). Firstly, HEK293T cells were transiently transfected using linear polyethylenimine PEI MAX 40 K (24765-1, PolySciences Europe) with packaging vector (pCMV dR8.91), envelope vector (pMD2-VSV), and the cloned pLenti6/V5 (Life Technologies) plasmids. After 48 h, the virus containing supernatant was collected from the HEK293T cells, filtered through a 0.45 μm filter (SLHV033RB, Millipore), and used to infect the destination cells. After 24 h transduction virus medium was removed, and selected for infected cells by adding blasticidin S (5 μg/ml, InvivoGen Europe) or Puromycin (1 μg/ml; InvivoGen Europe). Cells were cultured at 37 °C and 5% CO_2_.

### Protein isolation and fractionations mitochondrial fraction isolation

Cells were collected by trypsinization followed by washing in phosphate-buffered saline (PBS). A portion of the PBS-washed cell pellet was set aside for preparation of whole-cell lysates (WCL). For this, pellets were resuspended in RIPA lysis buffer (137 mM NaCl, 2.7 mM KCl, 10 mM sodium fluoride, 1 mM sodium pyrophosphate, 0.5% Nonidet P-40), supplemented with complete protease inhibitor (78429, Thermo Fisher Scientific) and PhosSTOP inhibitor mix (4906837001, Roche Applied Science). Samples were incubated on ice for 20 min, centrifuged at 20,000 × *g* for 10 min, and the resulting NP-40–soluble supernatant was collected as the whole cell lysate (WCL) fraction for western blot analysis.

The remaining PBS-washed cells were processed for mitochondrial isolation. Pellets were resuspended in mitochondrial isolation buffer (250 mM mannitol, 0.5 mM EGTA, 5 mM HEPES–KOH, pH 7.4) and mechanically disrupted using a 26.5 G needle (303800, Becton Dickinson) with ten strokes. The homogenate was subjected to differential centrifugation: two spins at 600 × *g* for 10 min to remove debris and nuclei, followed by two spins at 7000 × *g* for 10 min to pellet crude mitochondria. The mitochondrial pellet was washed in isolation buffer, while the post-mitochondrial supernatant was retained as the cytosolic fraction. Washed mitochondrial pellets were further purified by two centrifugations at 10,000 × *g* for 10 min, then lysed in RIPA buffer. All isolation steps were performed at 4 °C.

### Proteinase K protection assay

Mitochondria were isolated as described above and subjected to a proteinase K protection assay. Isolated mitochondria were divided over four different tubes. Two tubes contained regular mitochondrial isolation buffer (250 mM mannitol, 0.5 mM EGTA, and 5 mM HEPES–KOH pH 7.4), while two other tubes contained osmotic swelling buffer (0.5 mM EGTA and 5 mM HEPES–KOH pH 7.4) due to the absence of mannitol. Later, we added 10 μg/ml proteinase K (EO0492, Life Technologies) to one tube of each condition (that is, one tube of regular isolation buffer and one tube of osmotic swelling buffer). Mitochondria were subjected to proteinase K for 20 min at 4 °C on a head-over-head rotor. Proteinase K digestion was blocked using 1 mM phenylmethylsulfonyl fluoride (PMSF, sc-482875, Santa Cruz Biotechnology) on ice for 10 min. Mitochondria were isolated by centrifugation at 10,000 × *g* for 10 min at 4 °C. Mitochondrial pellets were resuspended in NuPAGE LDS sample buffer (NP0007, Life Technologies) and analysed by SDS–PAGE and immunoblotting.

### Western blot

Pierce BCA assay (23225, Thermo Fisher Scientific) was used to determine the protein concentration of our samples, whereafter equal amounts of protein lysate were mixed with reducing NuPAGE LDS Sample buffer (NP0007, Life Technologies) supplemented with 100 mM DTT (20290, Thermo Fisher Scientific), heated to 95 °C for 5 min. The cell lysates were fractioned by SDS-PAGE on a 4–12% gel (NP0322BOX, Invitrogen) with SeeBlue Plus2 Pre-stained Protein Standard gels (LC5925, Life Technologies). After separation, proteins were transferred to nitrocellulose membranes (RPN132D, GE Healthcare Life Sciences) for 1 h at 4 °C. Membranes were blocked with 5% milk powder dissolved in PBS-T. All the primary antibodies (Table 2) were incubated overnight at 4 °C, followed by secondary antibody incubation for 1 h at room temperature.

**Table 2:** Primary antibodies used in this study.

Target	Dilution	Catalog No.	Supplier
HSPB1	1:1000	SPA-800	Enzo Life Sciences
HSPB3	1:500	ab213591	Abcam
HSPB5	1:1000	MAB4849	R&D Systems
HSPB8	1:1000	#3059	Cell Signaling Technology
VDAC1	1:1000	ab14734	Abcam
Tubulin	1:10,000	ab7291	Abcam
TOM20	1:1000	ab56783	Abcam
AIFM1	1:1000	GTX102399	Genetex
AIFM1 (alternative)	1:1000	#4642	Cell Signaling Technology
HCCS	1:2000	15118-1-AP	Proteintech
SDHA	1:3000	GTX632636	Genetex
mtHSP60	1:200	sc-13115	Santa Cruz Biotechnology
HSP70	1:5000	ab5439	Abcam
CytC	1:1000	#4272	Cell Signaling Technology
V5	1:1,000	R960-25	Invitrogen
GFP	1:1000	ab290	Abcam
Procaspase-9	1:1000	#9502	Cell Signaling Technology
Caspase-3	1:500	#700182	Invitrogen
PARP	1:1000	sc-7150	Santa Cruz Biotechnology
PARP (alternative)	1:1000	#9545	Cell Signaling Technology

Secondary antibodies were horseradish peroxidase conjugated (Jackson ImmunoResearch Europe). Blots were developed with enhanced Chemiluminescence ECL Plus (32132, Life Technologies) or SuperSignal West Femto Maximum Sensitivity Substrate (34096, Thermo Fisher Scientific), and imaged with an Amersham Imager 600 (GE Healthcare). The images were then saved in 16-bit image files. Band intensities were calculated by quantifying the mean pixel grey values using ImageJ software (90). Mean pixel grey values were calculated for rectangular regions of interest.

### H_2_DCFDA and TMRE measurements

Cells were seeded in 96-well plates (P96-1.5H-N, Cellvis, USA) with a concentration of 5 × 10^4^ cells per well and treated with increasing concentrations of H₂O₂ (25–125 μM; 30%, 8.22287, Merck) for 1 h at 37 °C and 5% CO₂. For ROS measurements, cells were washed with PBS and incubated with phenol-red-free RPMI 1640 medium (11835030, Thermo Fisher Scientific) containing 10 μM H₂DCFDA (Beyotime Biotechnology) for 30 min at 37 °C, after which green fluorescence was quantified using the IncuCyte™ S3 Live-Cell Analysis System (4647, Sartorius). For mitochondrial membrane potential analysis, cells were incubated with 200 nM TMRE (ENZ-52309, Enzo Life Sciences) for 30 min at 37 °C, washed with PBS, and either imaged on the IncuCyte™ S3 to measure red fluorescence or processed for flow cytometry. For flow cytometry experiments, cells were washed, trypsinized, and resuspended in PBS containing 2% (v/v) FBS before acquisition on a Cytek Aurora spectral flow cytometer, data were analysed using FlowJo v10 (Tree Star).

### RT-qPCR

RNA was harvested using the RNeasy Micro Kit (Qiagen) including a 15-min DNase incubation and converted into cDNA using the iScript cDNA synthesis kit (Qiagen) following the manufacturer’s manuals. For RT-qPCR, cDNA was diluted 10x and performed using a QuantStudio 6 Flex. Primers are shown in Table 3.

**Table 3:** Primers used in this study.

Gene	Forward primer	Reverse primer
GAPDH	GAAGGTGAAGGTCGGAGTCA	GAAGATGGTGATGGGATTTC
ACTB	AACCGCGAGAAGATGACCC	GCCAGAGGCGTACAGGGATAG
HSPB1	AAGGATGGCGTGGTGGAGAT	AAAGAACACACAGGTGGCGG
HSPB8	GAAGACCAAAGATGGATACG	TTGTTGAAACTGCTCTCTCC

### Statistics

Statistical significance was determined using Graphpad. Prism have been performed using the statistical tests as stated in each figure legend. Data are presented as mean ± standard error mean (s.e.m.) if not indicated otherwise. The statistical significance was assessed using Student’s *t*-tests, one-way or two-way analysis of variance (ANOVA) with post-hoc multiple comparisons tests (Dunnett’s or Sidak) when more than two groups were analysed. All *P* values below 0.05 were considered significant, with *p* ≤ 0.05 = *, *P* ≤ 0.01 = **, *P* ≤ 0.001 = *** and *P* ≤ 0.0001 = ****. Exact *P* values can be found in each figure legend. We have not excluded any samples.

## Supplementary information

Supplementary figures and tables

Additional information - whole blot images

## Data Availability

The data supporting the findings of this study are provided within the manuscript and its Supplementary Information. Raw western blot images underlying the reported results are included in the supporting documents. Additional data are available from the corresponding author upon reasonable request.
