# Supplementary material for: Stress-induced recruitment of small heat shock protein 1 at the outer mitochondrial membrane underlies resistance to apoptosis
Source: Cell Death Discov. 2026 Jul 29;12:426. doi: 10.1038/s41420-026-03268-8 (PMC13710539; doi:10.1038/s41420-026-03268-8)
Supplement: Supplementary file 1 [file 41420_2026_3268_MOESM1_ESM.pdf]

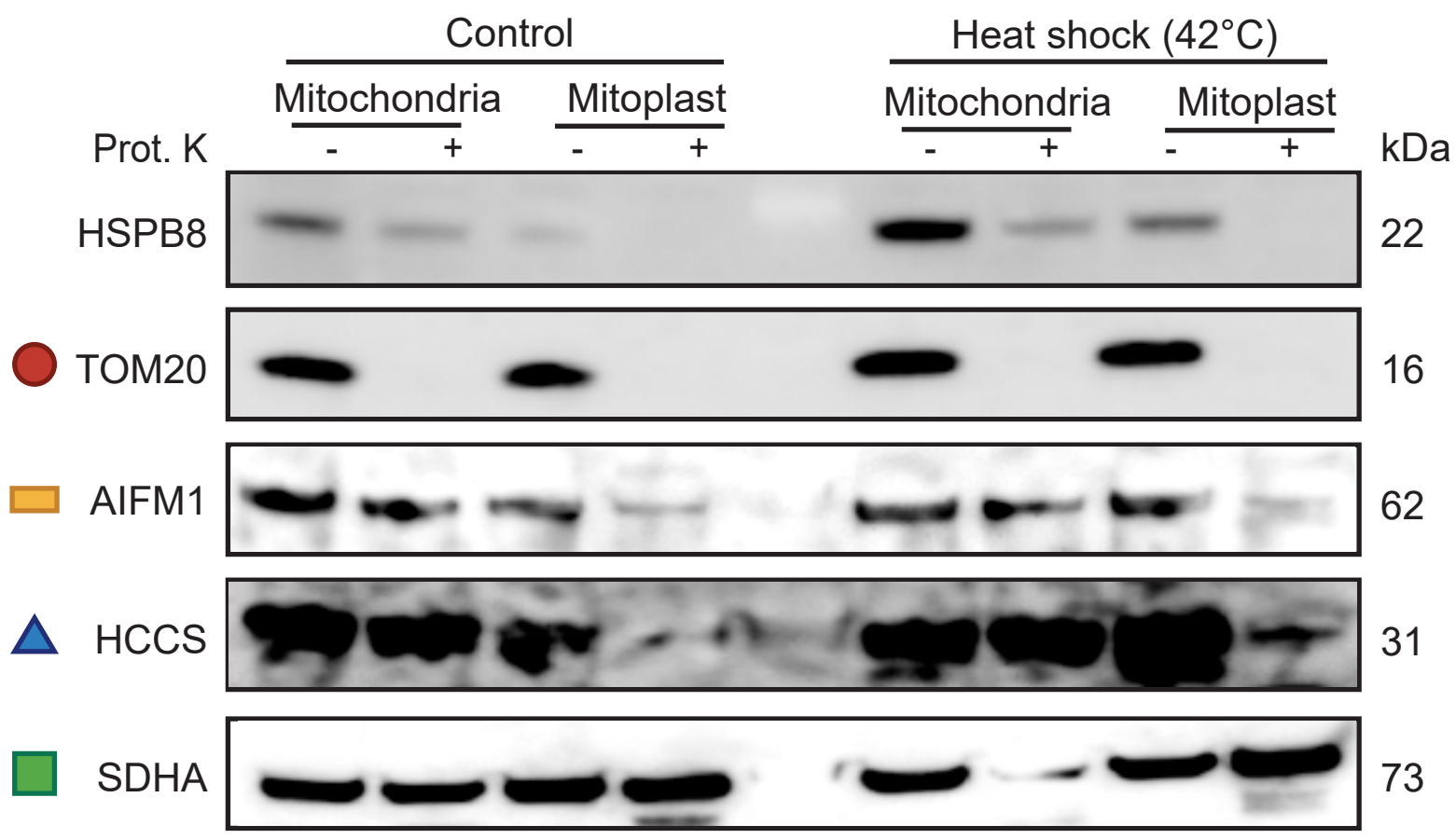


**Supplementary Figure 1: Sub-compartmental localisation of HSPB8 in the mitochondria** Western blot showing HSPB8 alongside mitochondrial protein localization markers (Fig. 1C) following exposure to proteinase K. Comparing the mitochondrial and mitoplast protein fractions derived from naïve and heat-shocked (42C for 1 hour) HeLa cells.

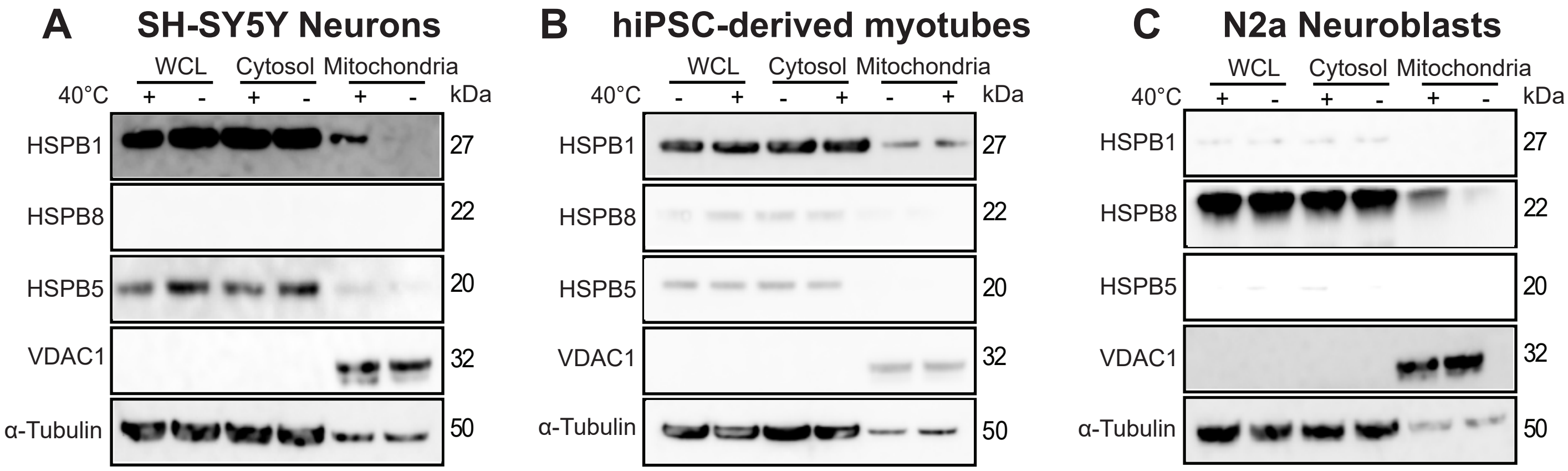


**Supplementary Figure 2: HSPB translocation in response to mild heat stress**

A. Western blot analysis of whole-cell lysates, cytosolic fractions, and mitochondrial fractions from hiPSC-derived myotubes under control conditions and mild thermal stress (40 °C), showing expression of HSPB1, HSPB5, HSPB8, VDAC1, and α-tubulin. B. Same as in A, performed in. SH-SY5Y neurons.  C. Same as in A, performed in N2a neuroblasts

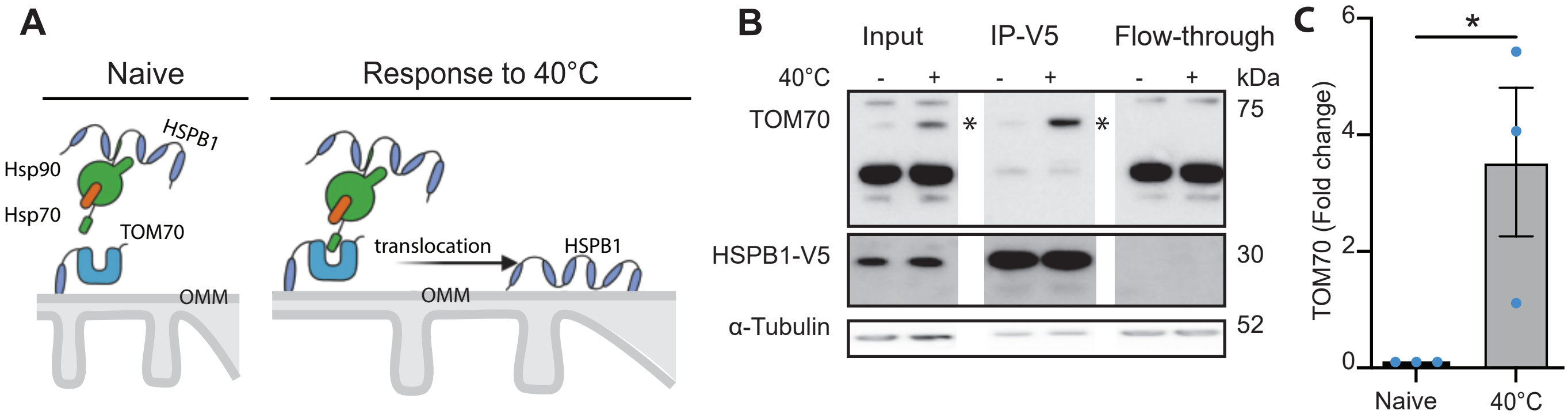


**Supplementary Figure 3: Immunoprecipitation of HSPB1-bound TOM70**

A. Graphical illustration of HSPB1 interacting with cytosolic chaperones Hsp70 and Hsp90, and its subsequent translocation to the mitochondrial outer membrane (MOM) through the TOM70-complex, in response to heat stress. B. Western blot comparison of naïve and heat-treated cells showing TOM70, HSPB1, and α-tubulin following immunoprecipitation of HSPB1-V5 using an anti-V5 antibody. Input, V5-bound, and unbound protein fractions are shown, demonstrating an enrichment of TOM70 (indicated with an asterisk) following heat treatment. C. Quantification of the immunoprecipitated V5-bound fraction of Figure 1F (n = 3 independent experiments). Statistical significance throughout this figure was determined relative to the appropriate control sample using one-way ANOVA followed by Sidak's post hoc multiple comparison test. ns = no significance, * = $p < 0.05$.

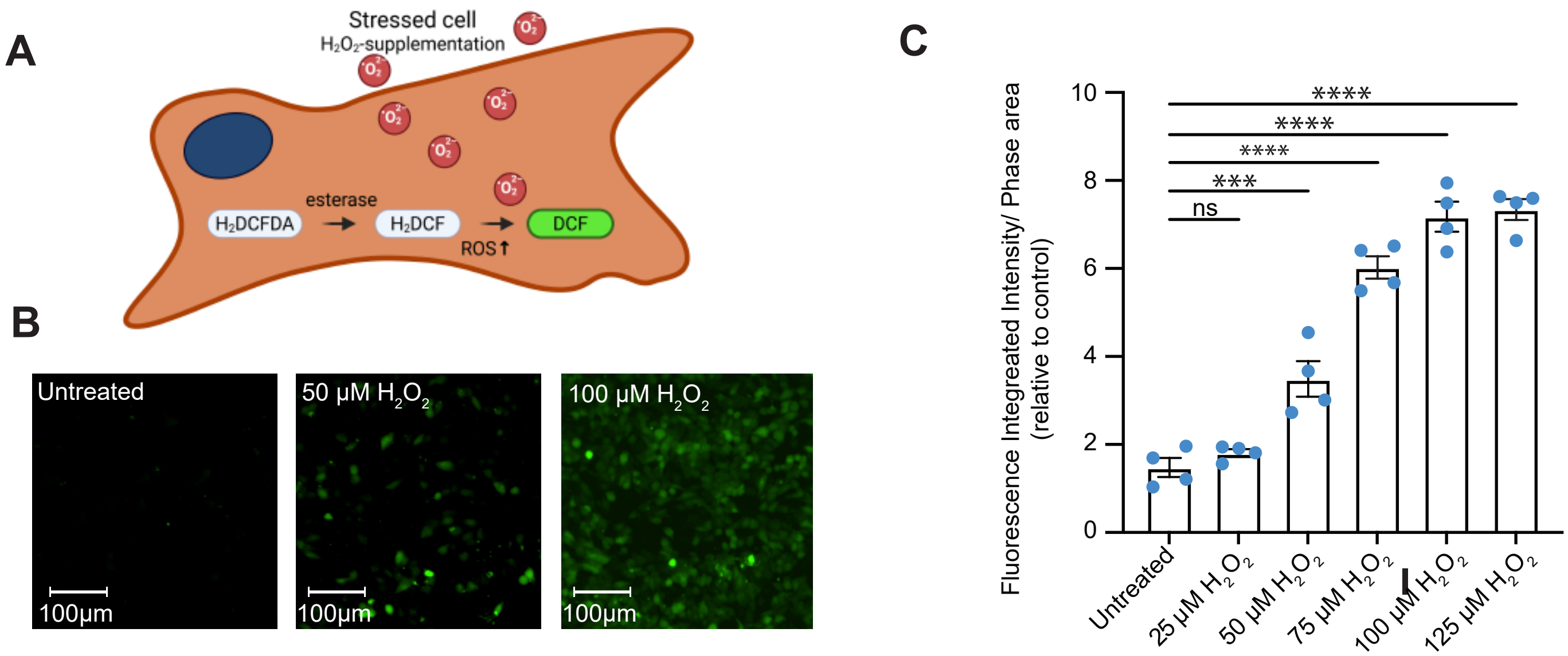


**Supplementary Figure 4: Validation of oxidative stress generation in HeLa cells**
A. Graphical representation of the H2DCFDA Assay, whereby intracellular H2DCFDA is converted into fluorescent active HDCFDA. B. Representative microscopy images showing the fluorescence signals in H2DCFDA -supplemented Hela cells exposed to 100µM H2O2, and CCCP as a control. C. Quantification of the intracellular fluorescent intensity relative the number of cells in each image. Data represents mean ± s.e.m. (n = 4) Statistical significance was determined using One way ANOVA and Dunnett's post-hoc multiple testing correction. *p <0.05, **p<0.01, ***p <0.001, ****p<0.0001.

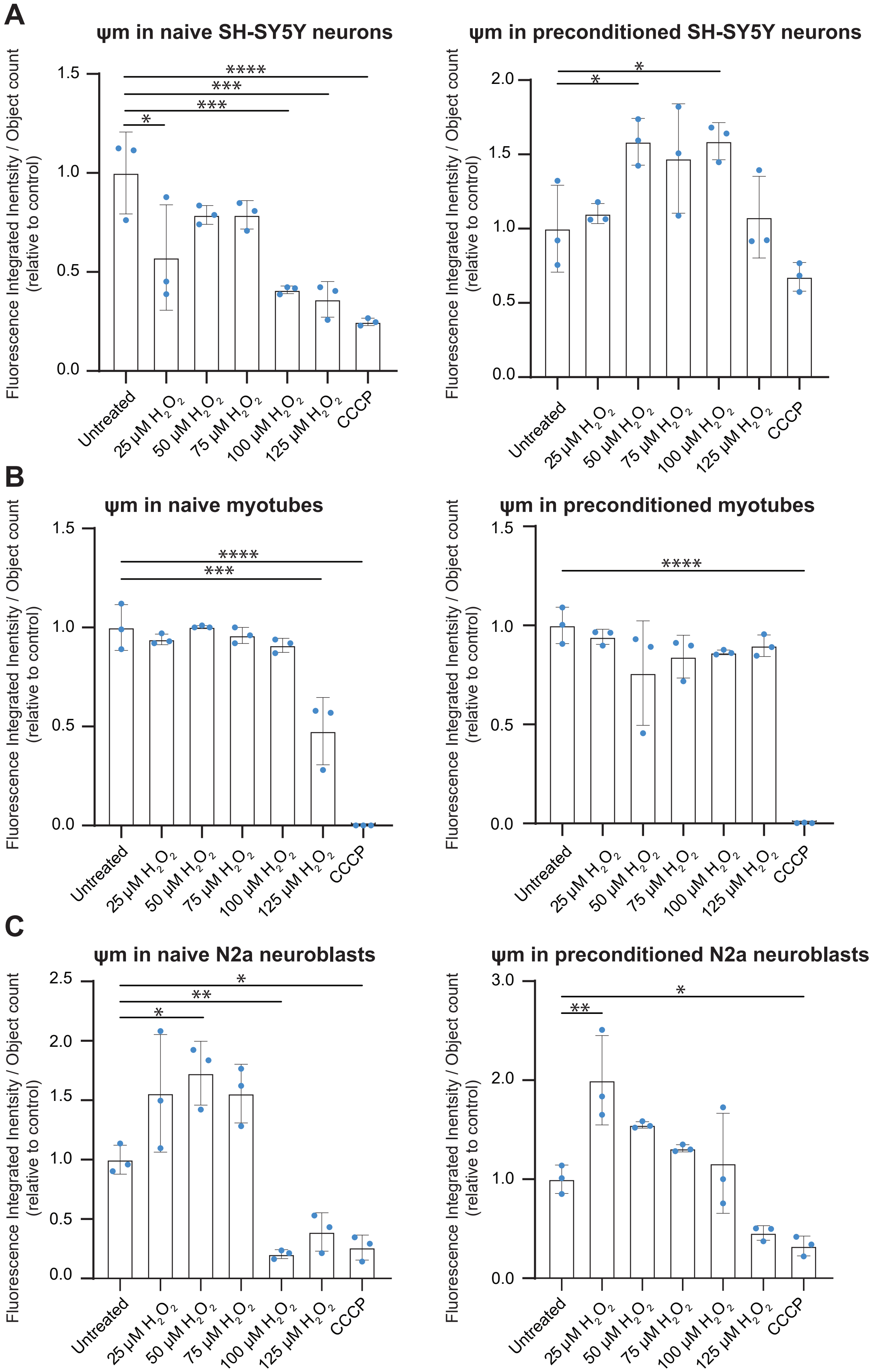


**Supplementary Figure 5: mitochondrial membrane potential in preconditioned cells.**

A. Quantification of TMRE fluorescence signal in naïve cells (left) and preconditioned (right) SH-Y5Y neurons, following the induction of oxidative stress with $H_2O_2$ or CCCP as control. B. Same as Fig. S4A, showing hiPSC-derived myotubes. C. Same as Fig. S4A, N2a neuroblasts. Data represents mean ±SD (n = 4 independent experiments). Statistical significance throughout this figure was determined relative to the untreated control sample using one-way ANOVA followed by Sidak's post hoc multiple comparison test. * = $p < 0.05$, ** = $p < 0.01$, *** = $p < 0.001$**** = $p < 0.0001$.

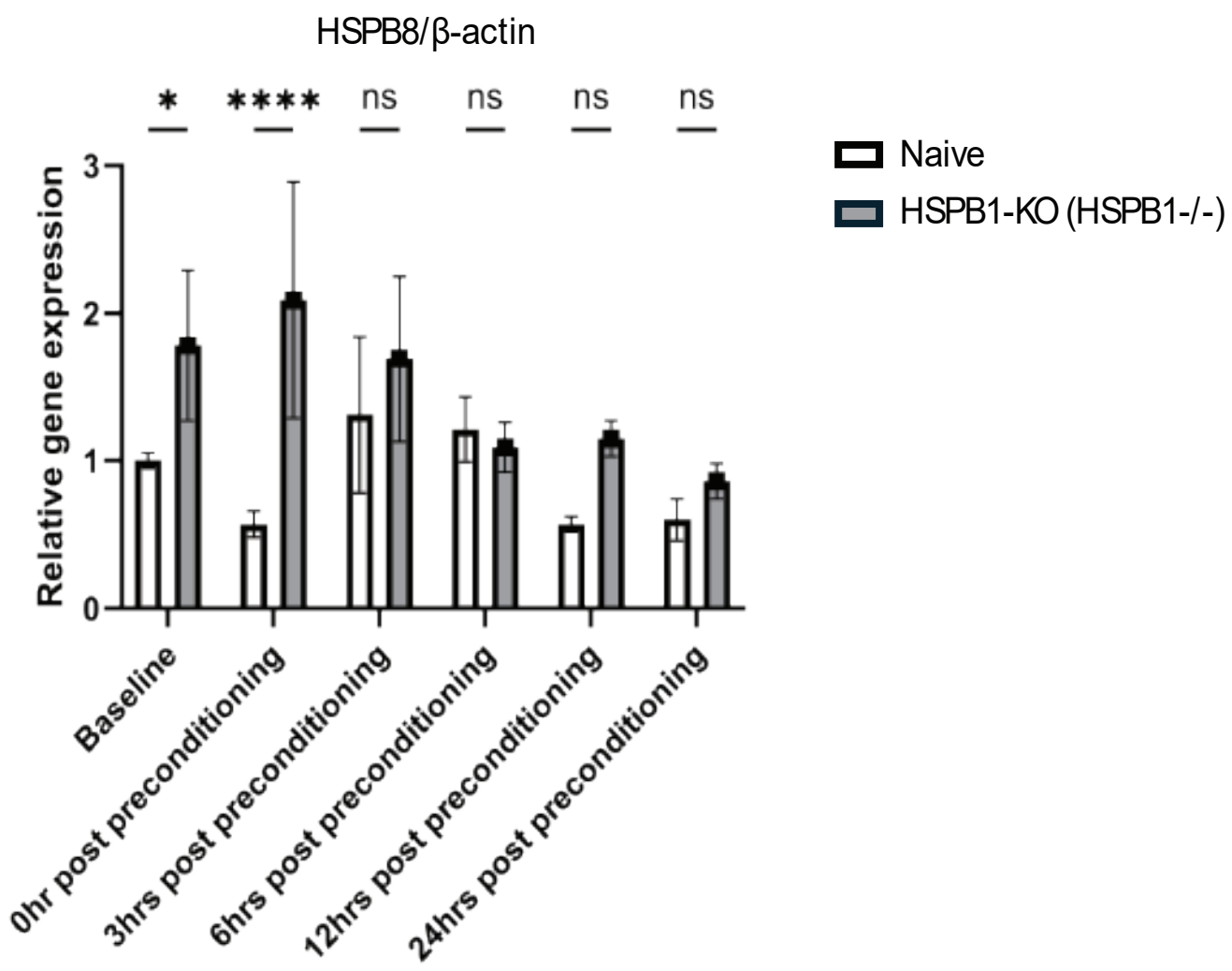


**Supplementary Figure 6: HSPB8 compensation for abrogation of HSPB1**
RT–qPCR analysis comparing the gene expression levels of HSPB8 of naïve and HSPB1-KO (HSPB1−/−) HeLa cells at baseline and up to 24 hours after mild heat-stress preconditioning (40 °C for 1 hour). Data represents mean ±SD (n = 3) Statistical significance was determined using One way ANOVA and Dunnett's post-hoc multiple testing correction. Ns = no significance, *p = <0.05, and **** = p<0.0001.

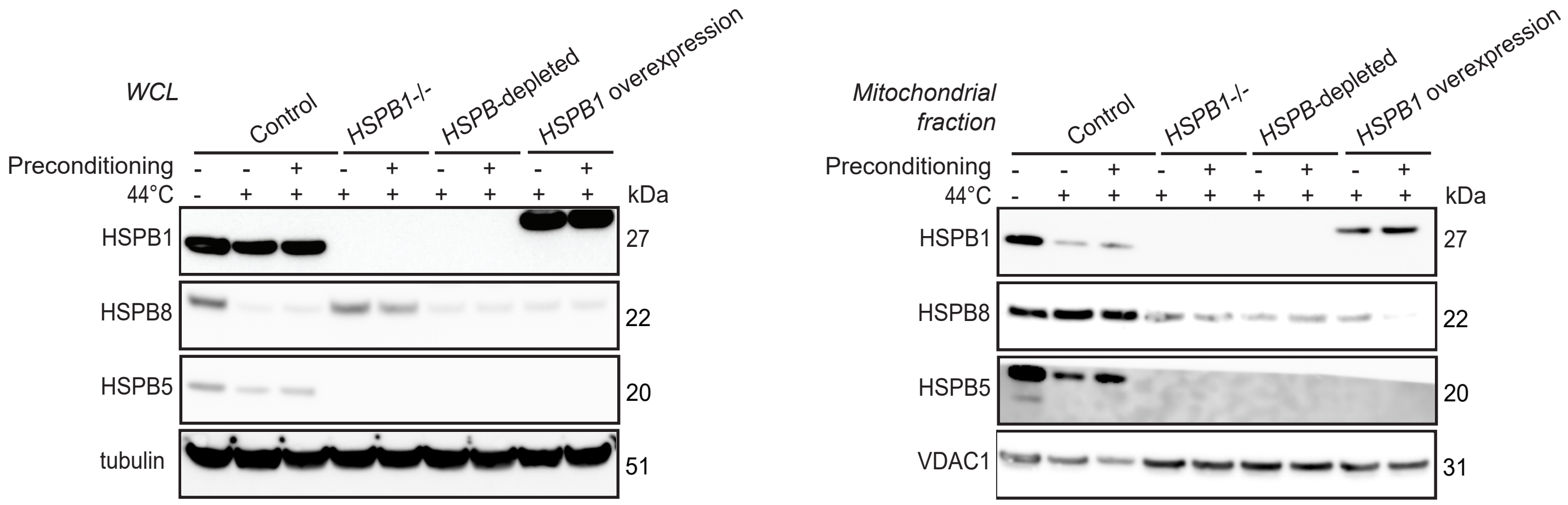


**Supplementary Figure 7: Validation of HSPB1 Knock-out (HSPB1$^{-/-}$), HSPBs$^{depl}$, and HSPB1$^{re\text{-}expression}$ cell lines**
A. Western blot of the WCL of control, HSPB1-/-, HSPBs-depleted, and HSPB1-overexpressing cells, showing the effects of lethal heat stress on HSPB1, HSPB8, and HSPB5 levels in naïve and preconditioned conditions. B. Same as Suppl. Fig. 7A, but in the mitochondrial protein fractions.

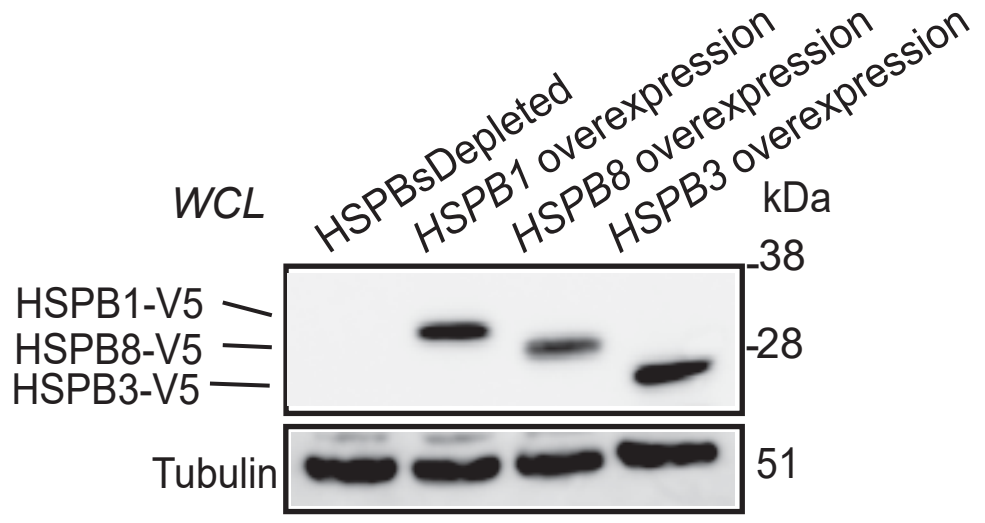


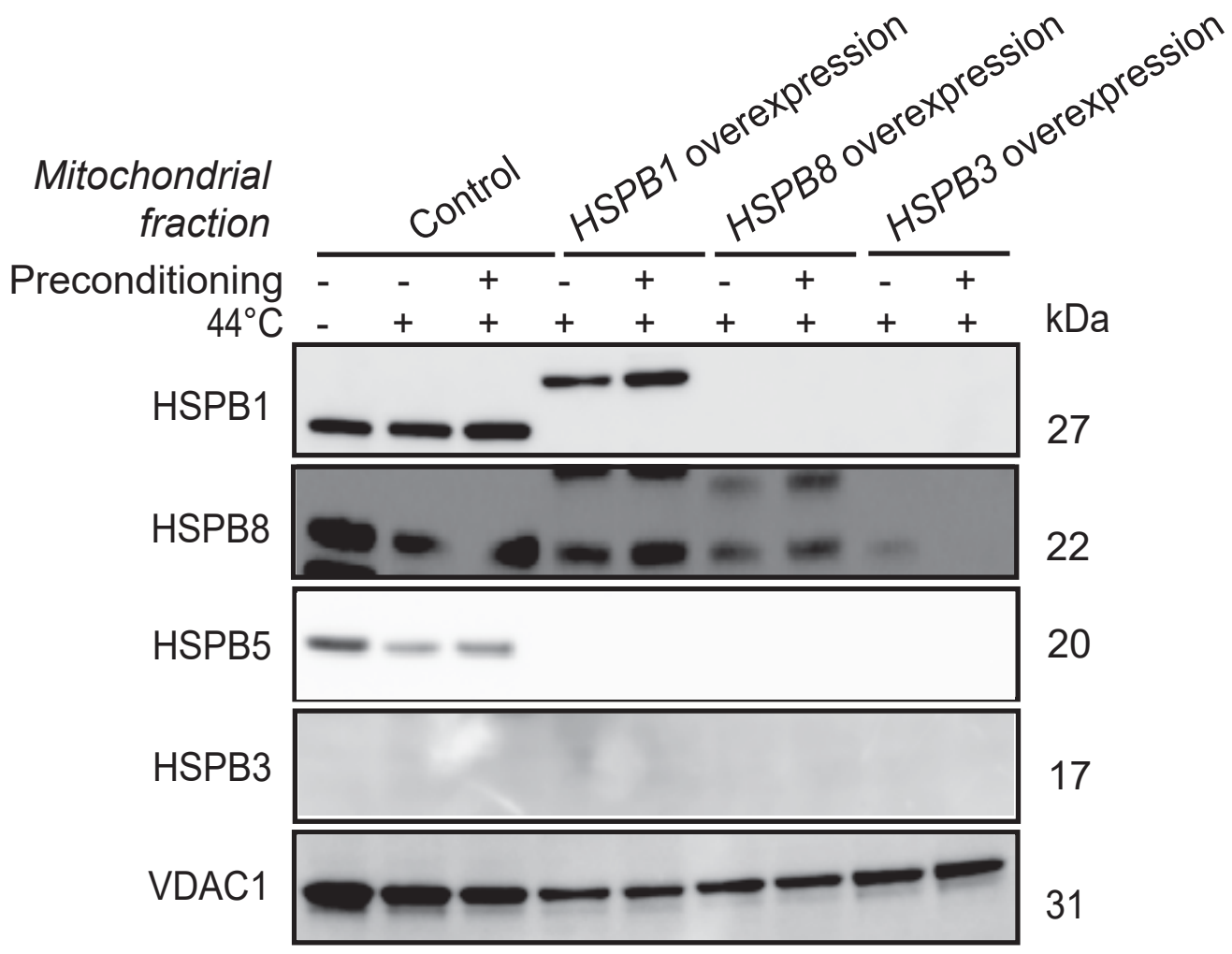


**Supplemental Figure 8: Validation of HSPB8- and HSPB3-rexpression cell lines**

A. Western blot and corresponding quantification of the WCL of control, HSPB1, HSPB8, and HSPB3-overexpressing cells, showing the effects of lethal heat stress on HSPB1, HSPB8, and HSPB3 levels in naïve and preconditioned conditions. B. Same as Suppl. Fig. 4A, but in the mitochondrial protein fractions, and additional showing HSPB5.
