# Supplementary material for: Stress-induced recruitment of small heat shock protein 1 at the outer mitochondrial membrane underlies resistance to apoptosis
Source: Cell Death Discov. 2026 Jul 29;12:426. doi: 10.1038/s41420-026-03268-8 (PMC13710539; doi:10.1038/s41420-026-03268-8)
Supplement: Supplementary file 2 [file 41420_2026_3268_MOESM2_ESM.pdf]

Figure 1:
1A (Replicate 1)

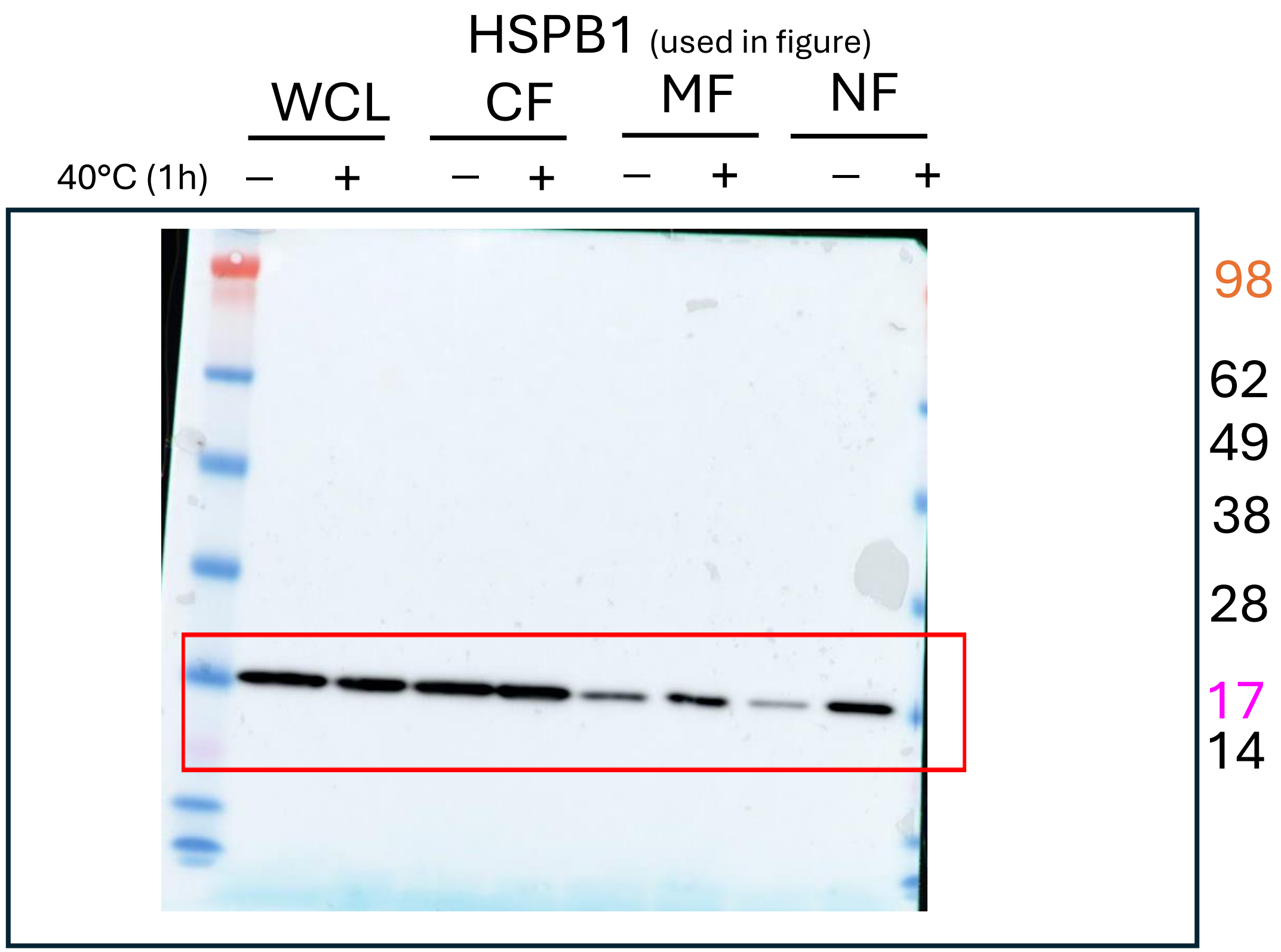

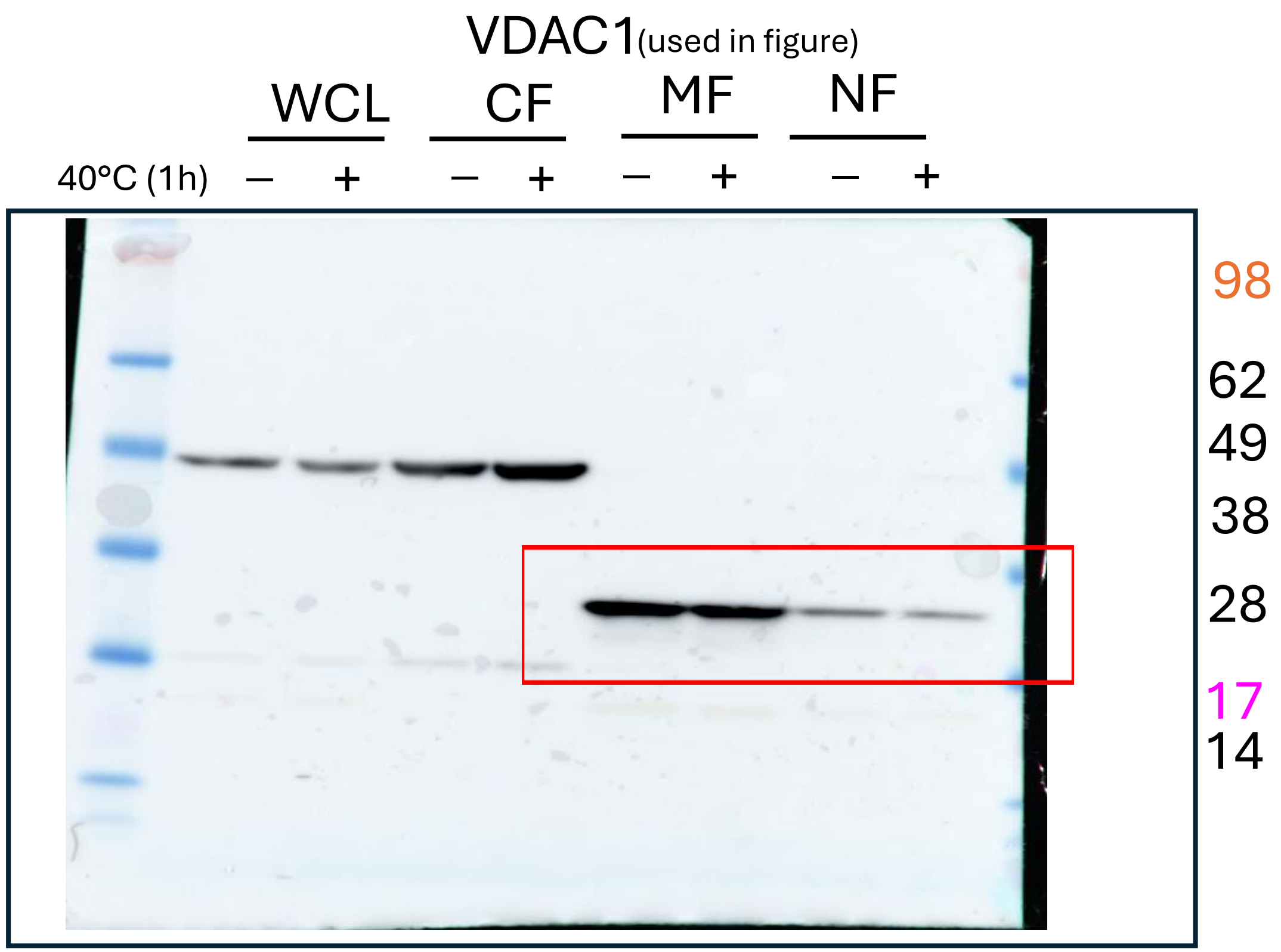

Bis-Tris 4-12% Gel
MES Buffer

WCL (Whole cell lysate)
CF (Cytosolic fraction)
MF (Mitochondrial fraction)
NF (Nuclear fraction)

Figure 1:
1A (Replicate 1)

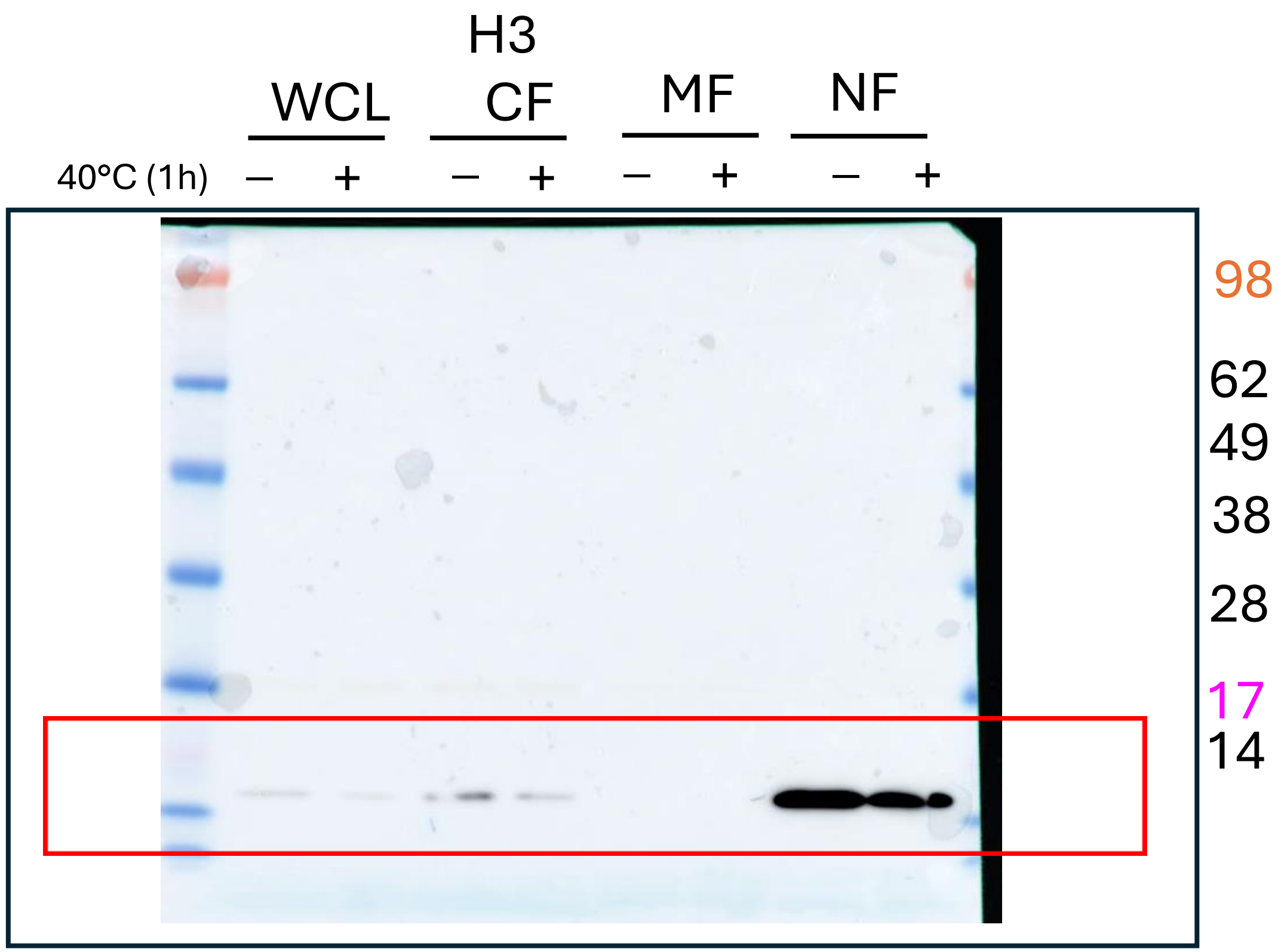

Bis-Tris 4-12% Gel
MES Buffer

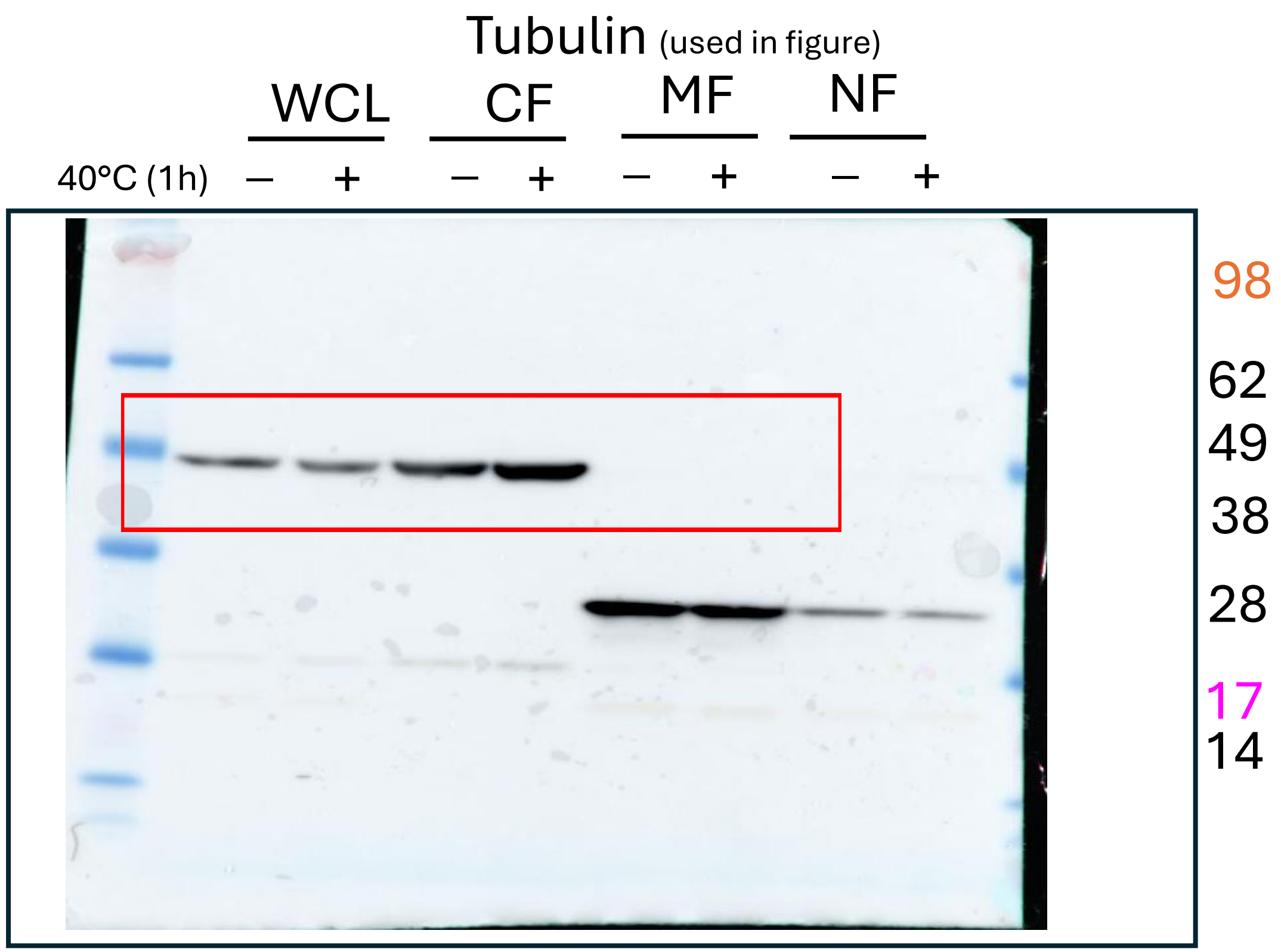

Bis-Tris 4-12% Gel
MES Buffer

WCL (Whole cell lysate)
CF (Cytosolic fraction)
MF (Mitochondrial fraction)
NF (Nuclear fraction)

Figure 1:
1A (Replicate 2)

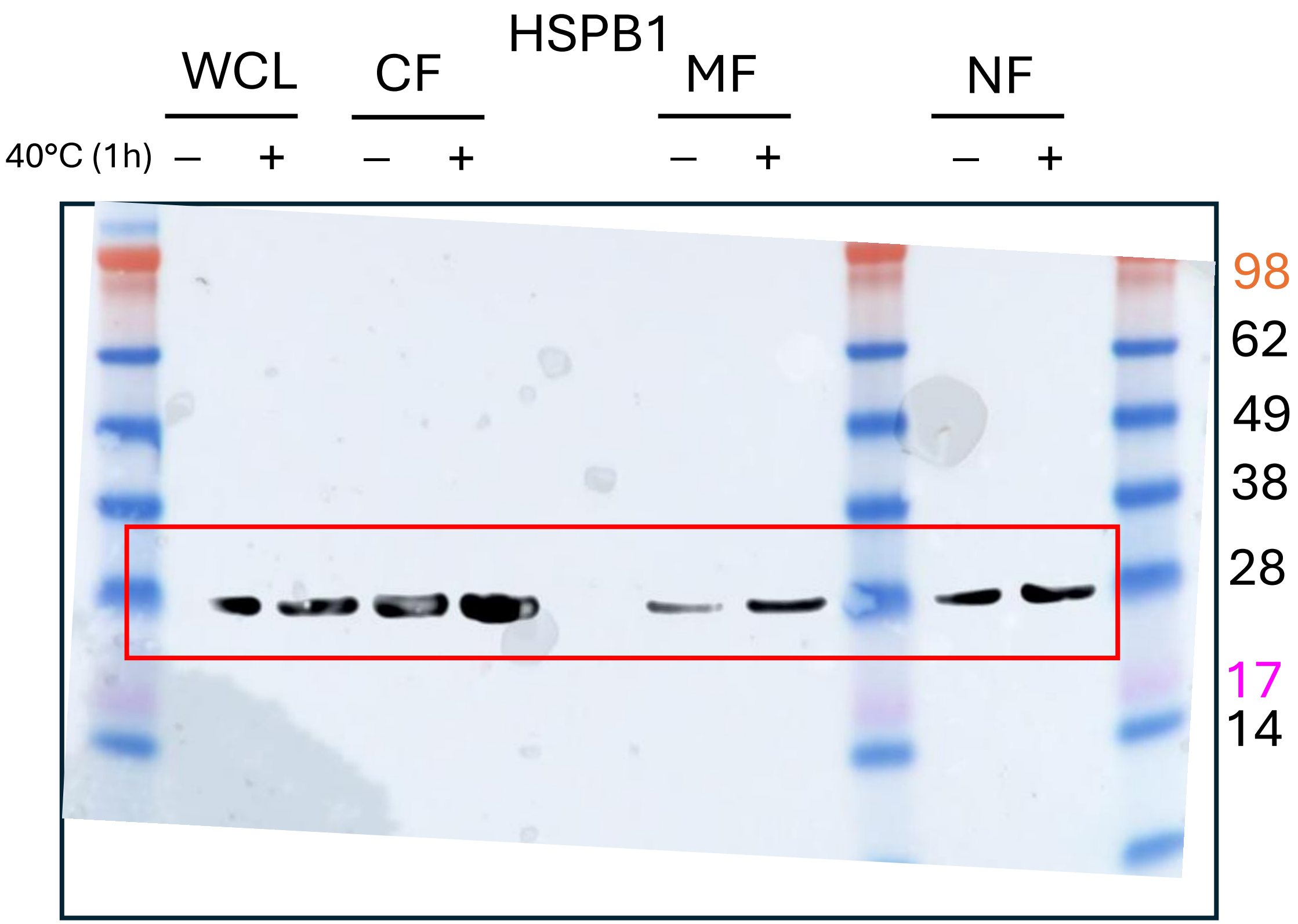

Bis-Tris 4-12% Gel
MES Buffer

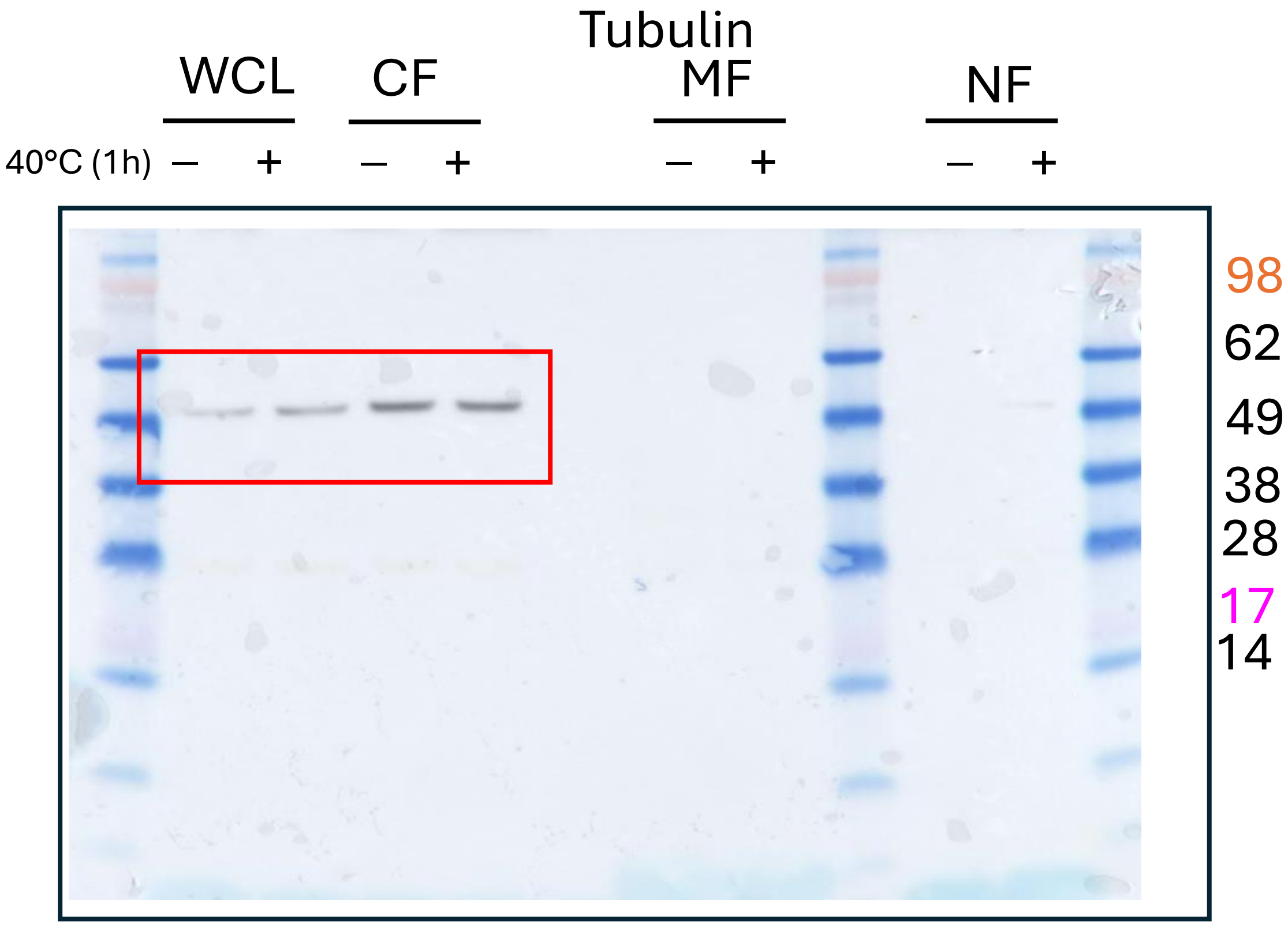

Bis-Tris 4-12% Gel
MES Buffer

WCL (Whole cell lysate)
CF (Cytosolic fraction)
MF (Mitochondrial fraction)
NF (Nuclear fraction)

Figure 1:
1A (Replicate 2)

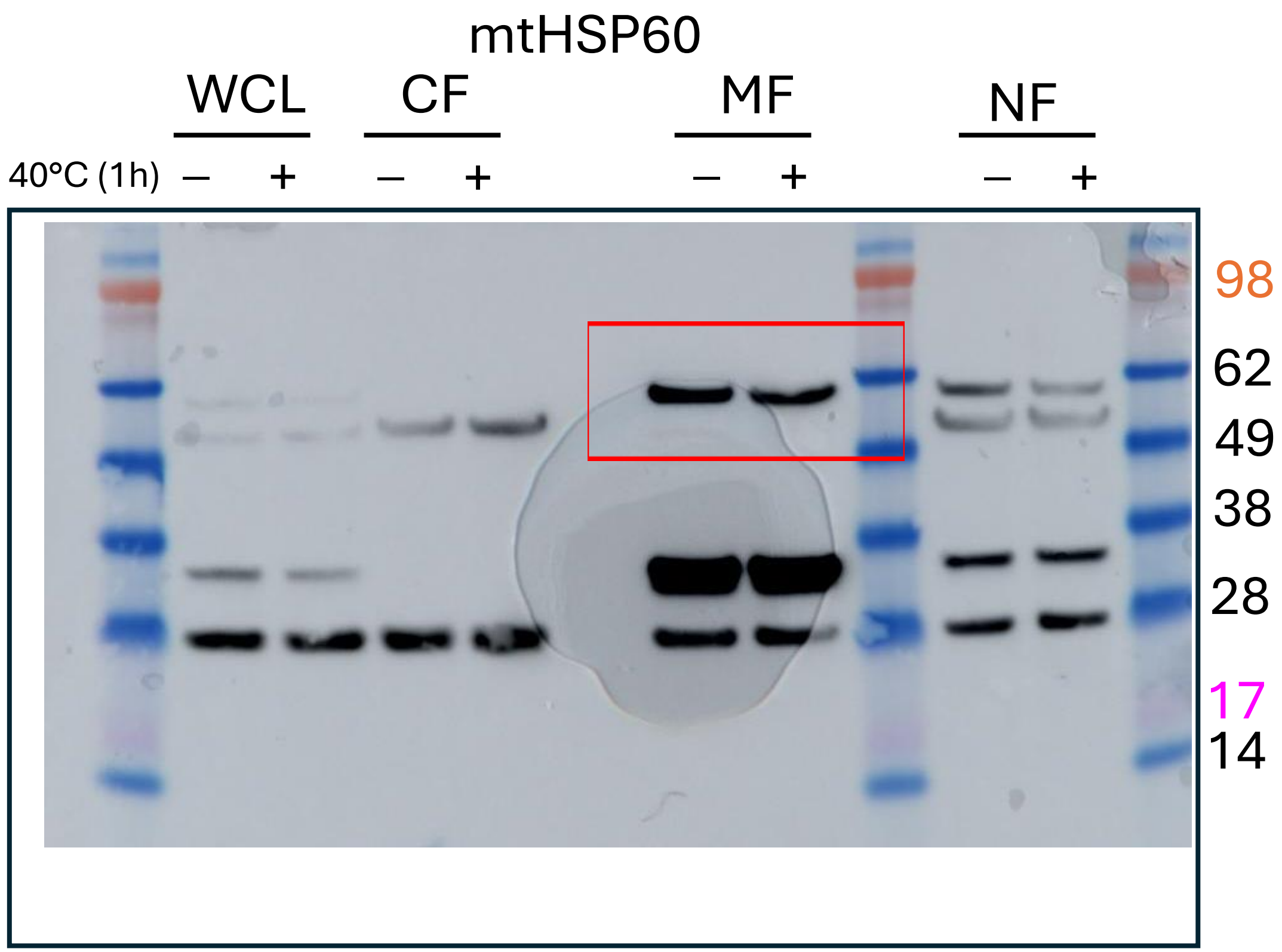

Bis-Tris 4-12% Gel
MES Buffer

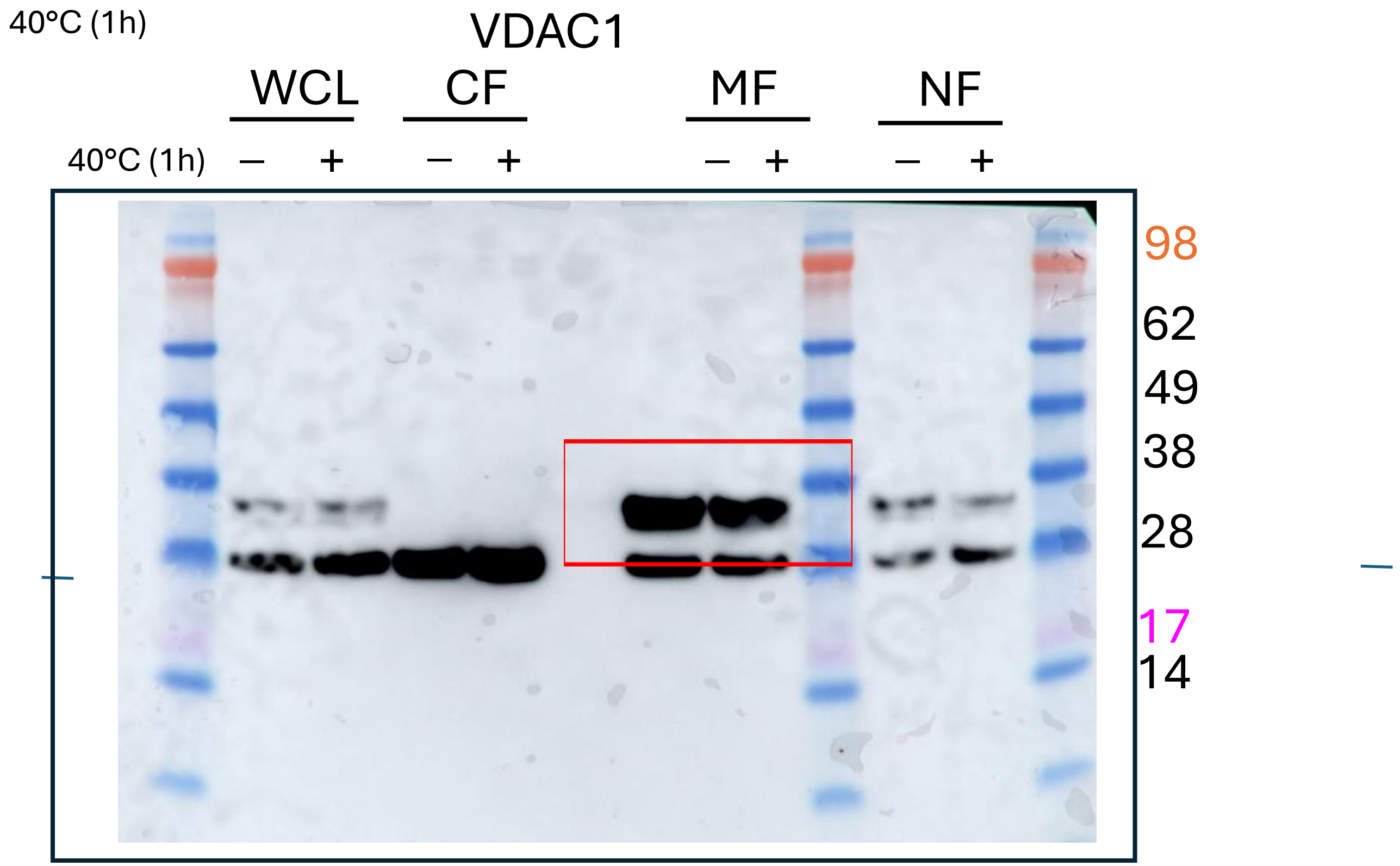

Bis-Tris 4-12% Gel
MES Buffer

WCL (Whole cell lysate)
CF (Cytosolic fraction)
MF (Mitochondrial fraction)
NF (Nuclear fraction)

Figure 1:

1A (Replicate 2)

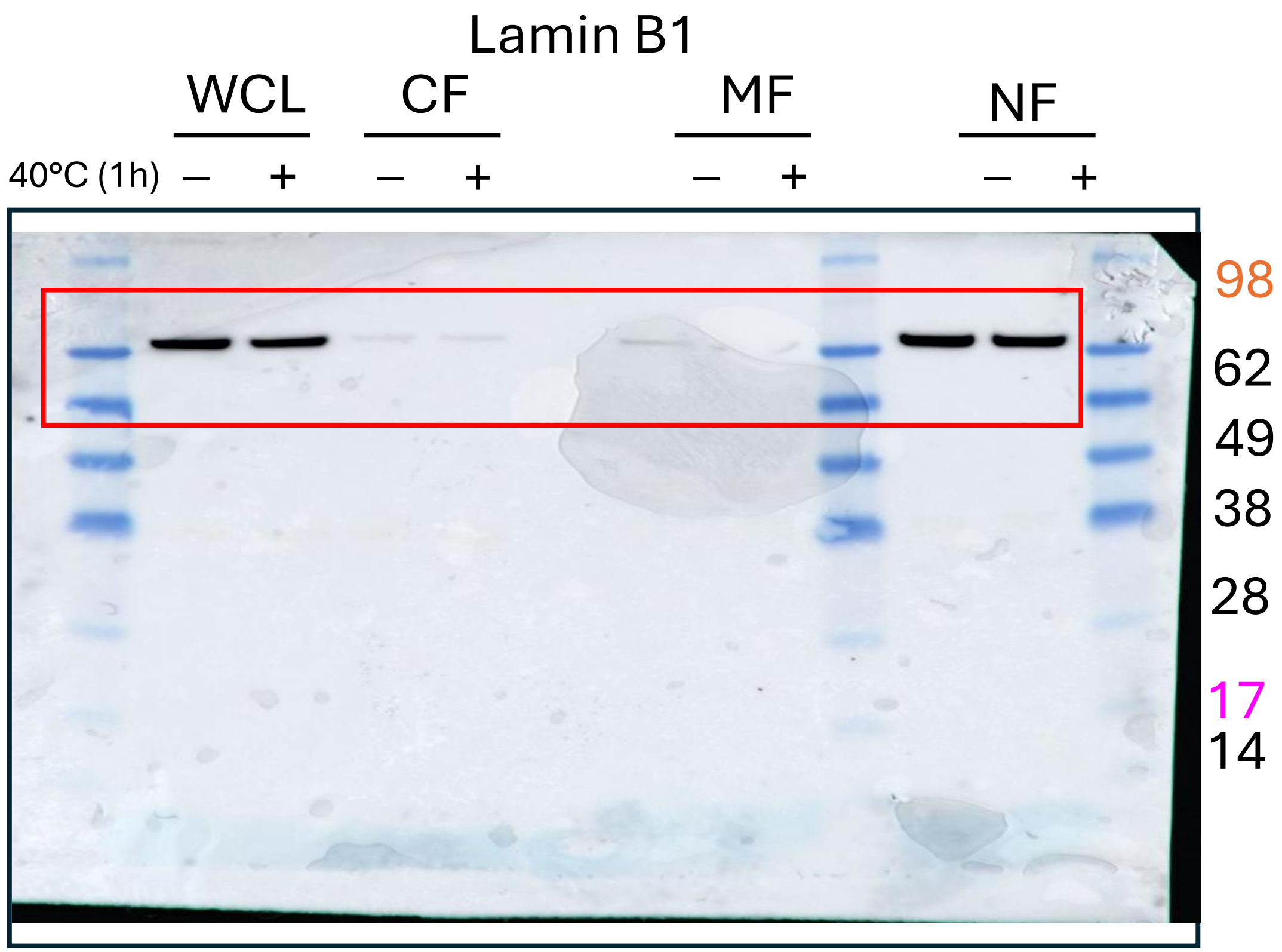

Bis-Tris 4-12% Gel
MES Buffer

WCL (Whole cell lysate)
CF (Cytosolic fraction)
MF (Mitochondrial fraction)
NF (Nuclear fraction)

Figure 1:
1A (Replicate 3)

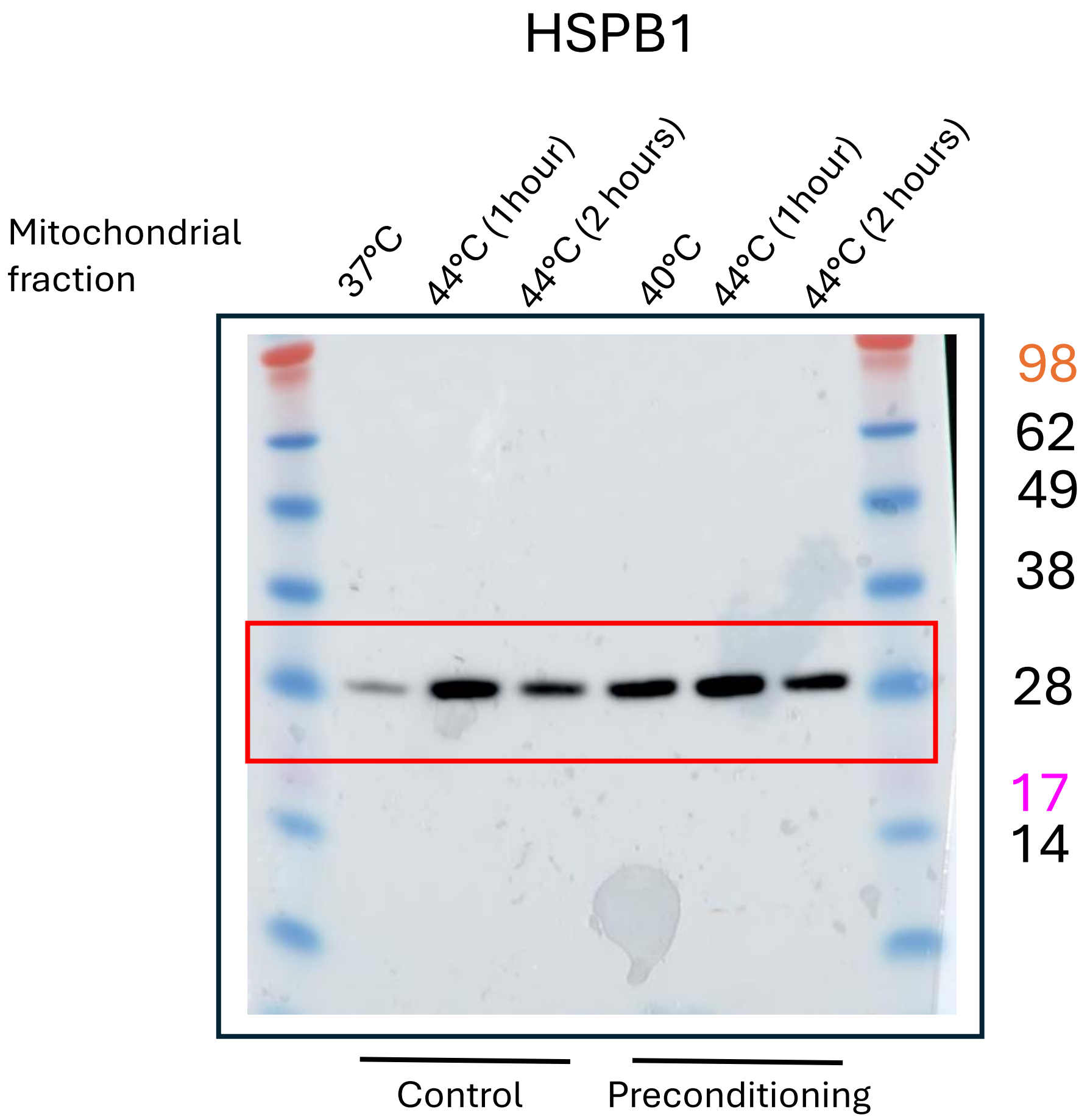

Bis-Tris 4-12% Gel
MES Buffer

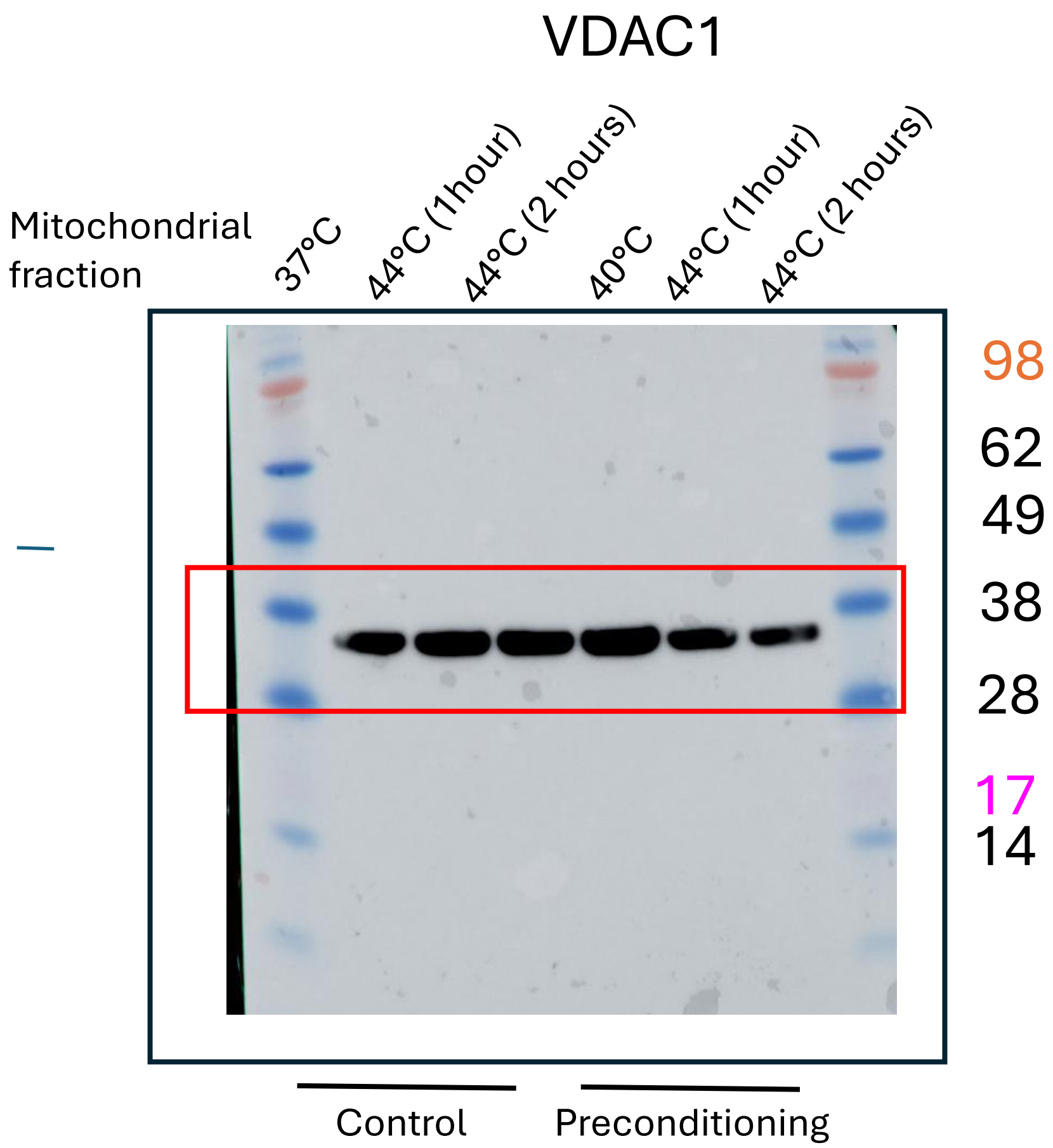

Bis-Tris 4-12% Gel
MES Buffer

Figure 1:
1A (Replicate 4)

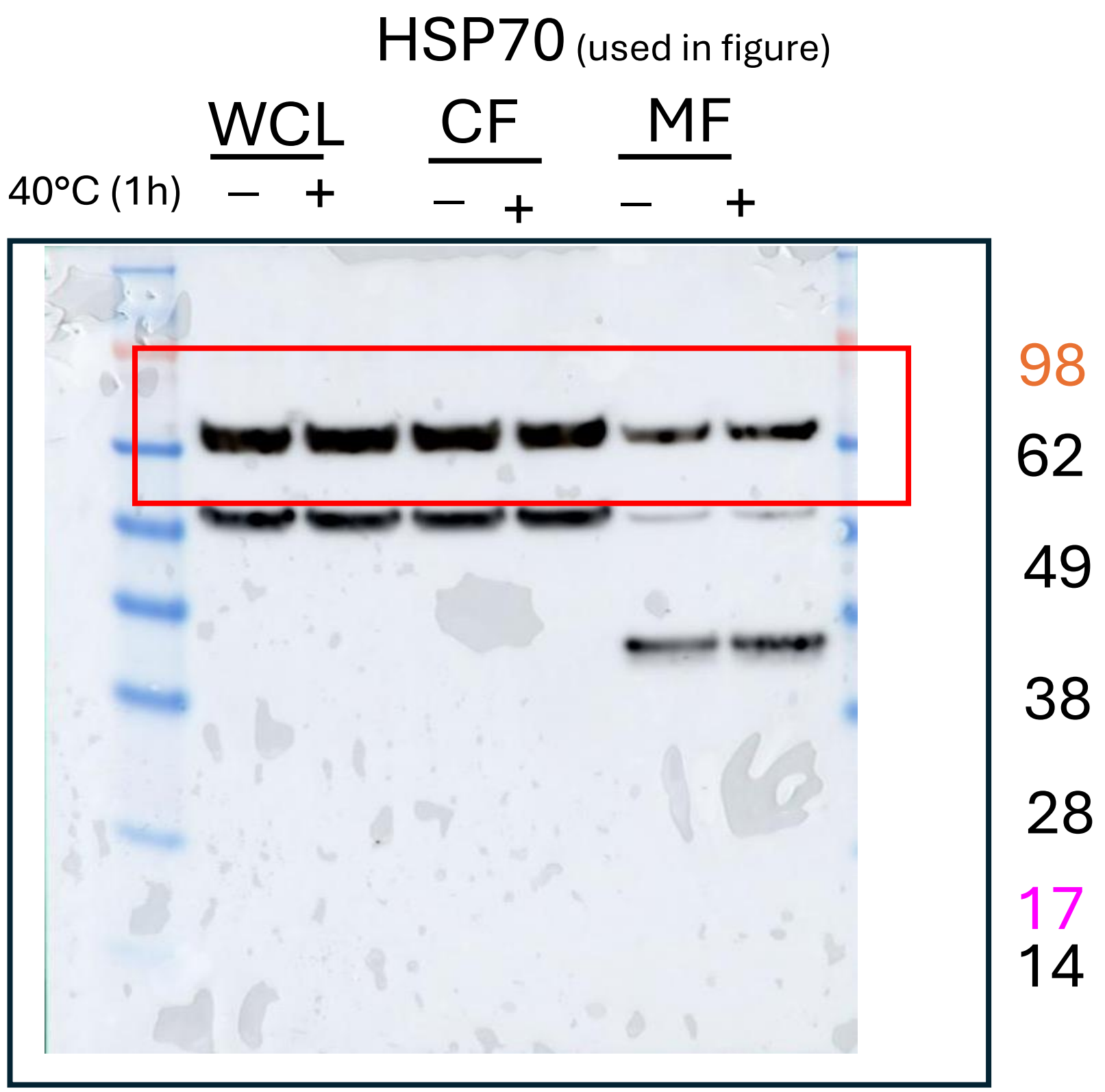

Bis-Tris 4-12% Gel
MES Buffer

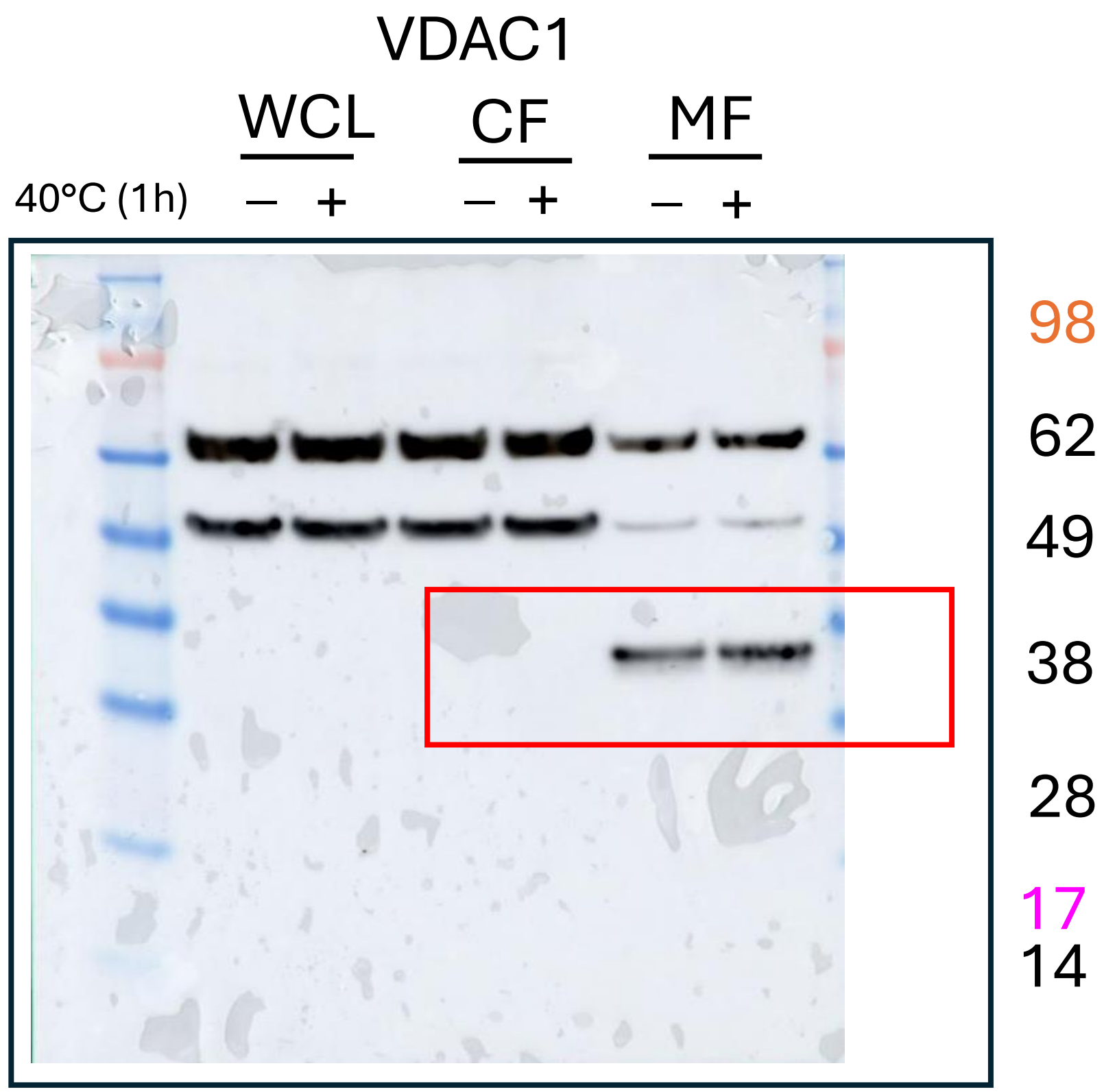

Bis-Tris 4-12% Gel
MES Buffer

WCL (Whole cell lysate)
CF (Cytosolic fraction)
MF (Mitochondrial fraction)
NF (Nuclear fraction)

Figure 1:
1A (Replicate 4)

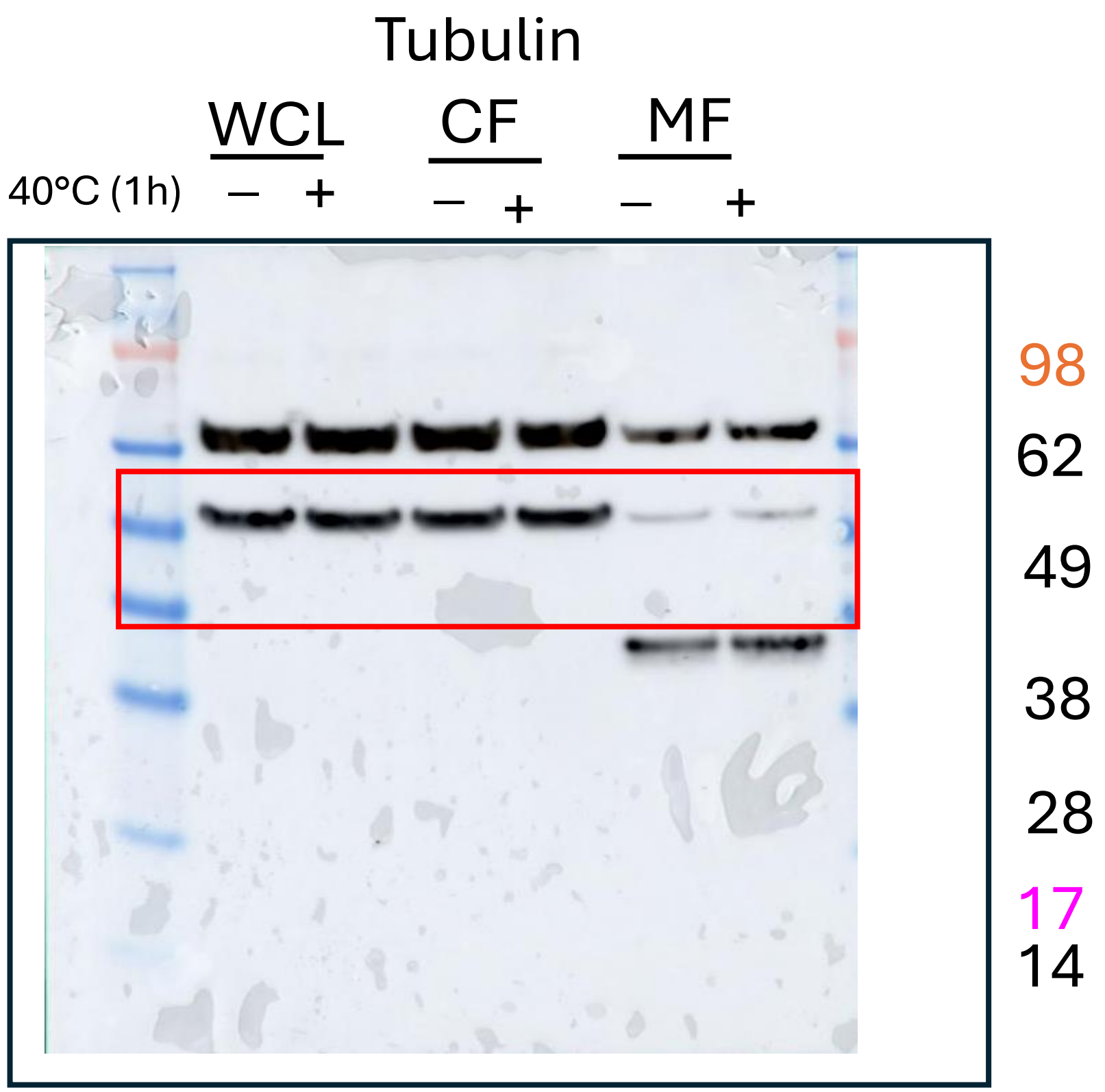

Bis-Tris 4-12% Gel
MES Buffer

WCL (Whole cell lysate)
CF (Cytosolic fraction)
MF (Mitochondrial fraction)
NF (Nuclear fraction)

Figure 1:

1A (Replicate 5)

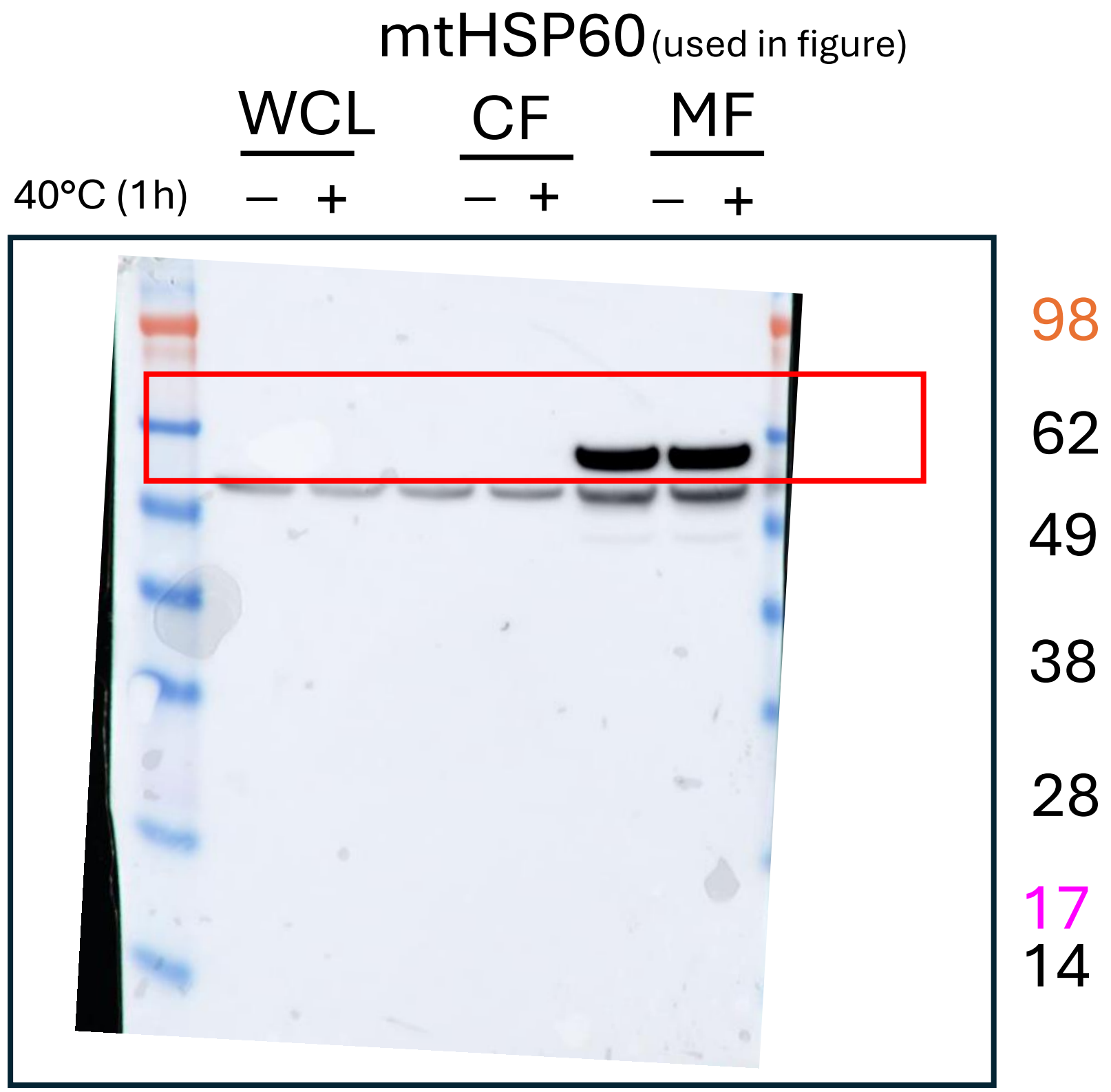

Bis-Tris 4-12% Gel
MES Buffer

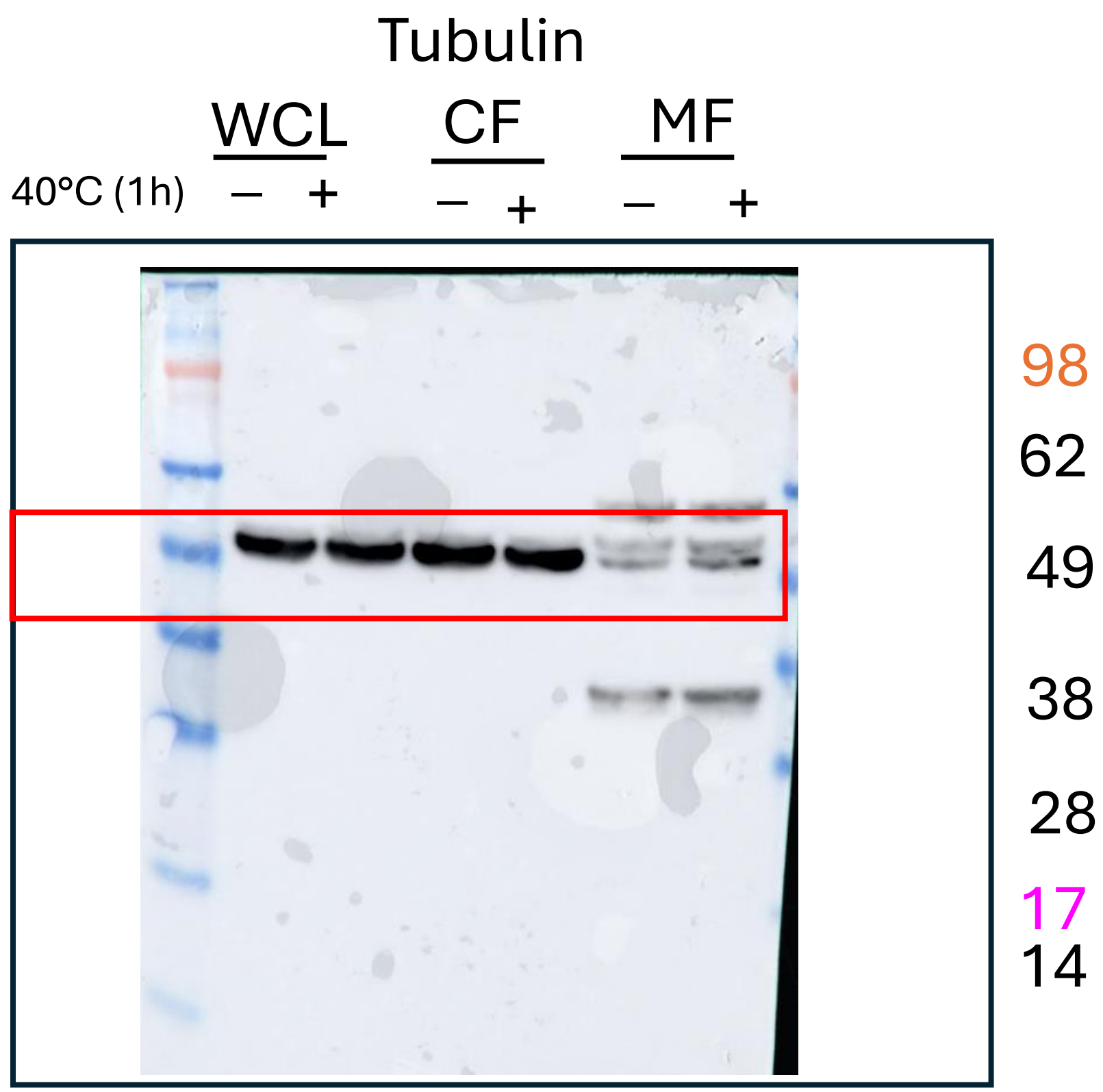

Bis-Tris 4-12% Gel
MES Buffer

WCL (Whole cell lysate)
CF (Cytosolic fraction)
MF (Mitochondrial fraction)
NF (Nuclear fraction)

Figure 1:
1A (Replicate 5)

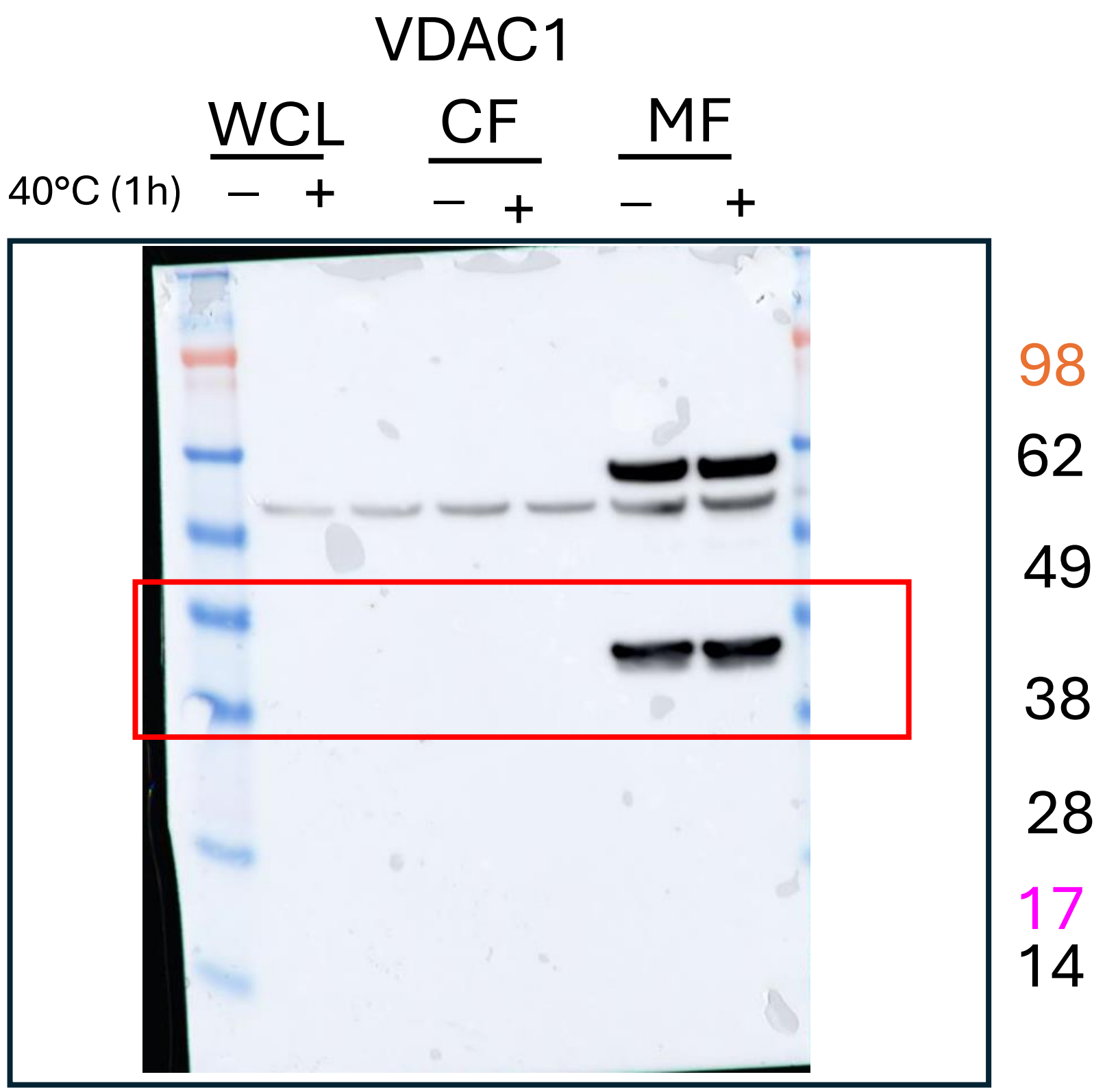

Bis-Tris 4-12% Gel
MES Buffer

WCL (Whole cell lysate)
CF (Cytosolic fraction)
MF (Mitochondrial fraction)
NF (Nuclear fraction)

Figure 1:
1A (Replicate 6)

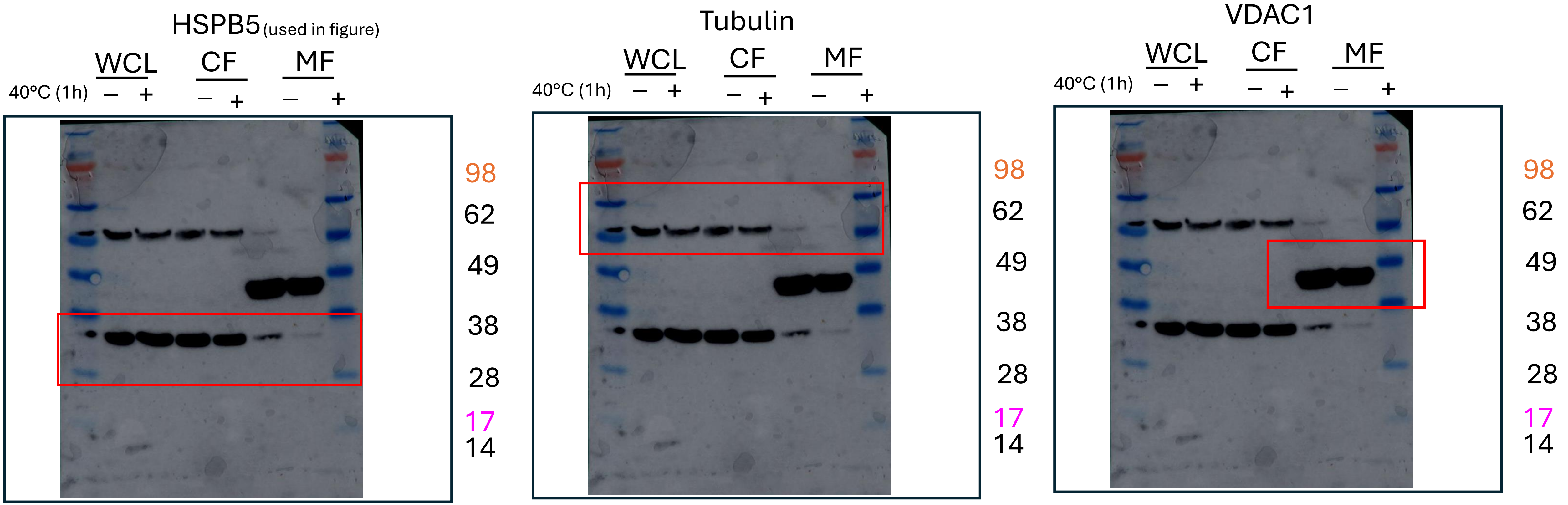

Bis-Tris 4-12% Gel
MES Buffer

Bis-Tris 4-12% Gel
MES Buffer

Bis-Tris 4-12% Gel
MES Buffer

WCL (Whole cell lysate)
CF (Cytosolic fraction)
MF (Mitochondrial fraction)
NF (Nuclear fraction)

Figure 1:
1A (Replicate 7)

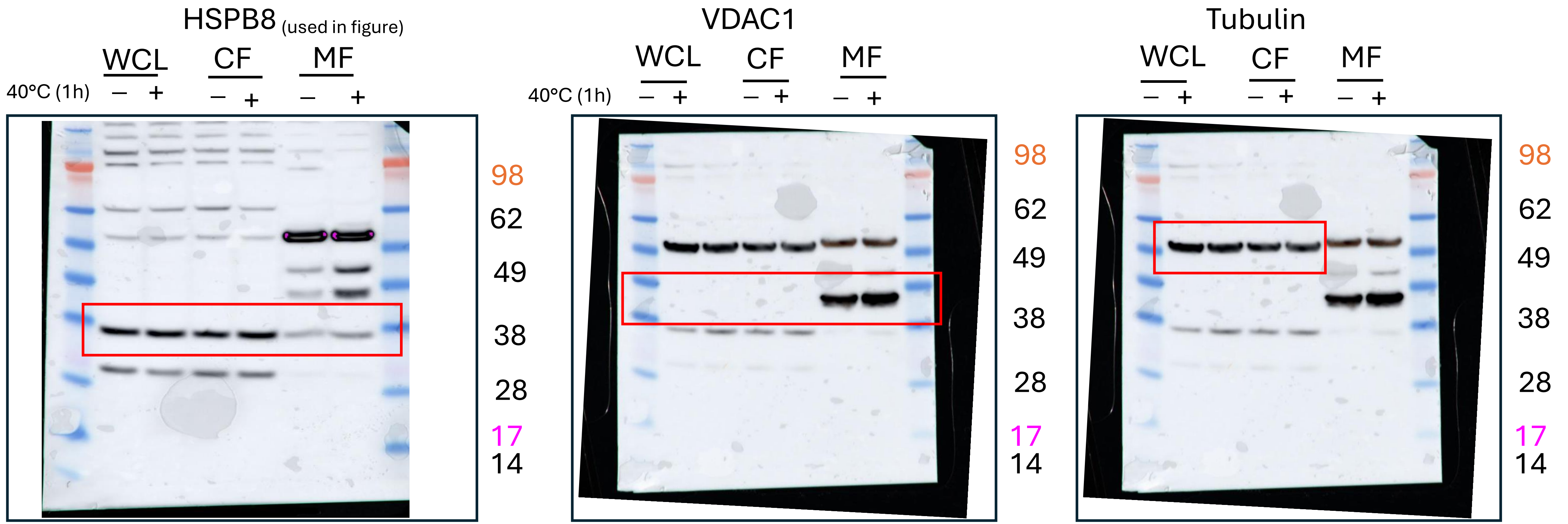

Bis-Tris 4-12% Gel
MES Buffer

Bis-Tris 4-12% Gel
MES Buffer

WCL (Whole cell lysate)
CF (Cytosolic fraction)
MF (Mitochondrial fraction)
NF (Nuclear fraction)

Figure 1:
1A (Replicate 7)

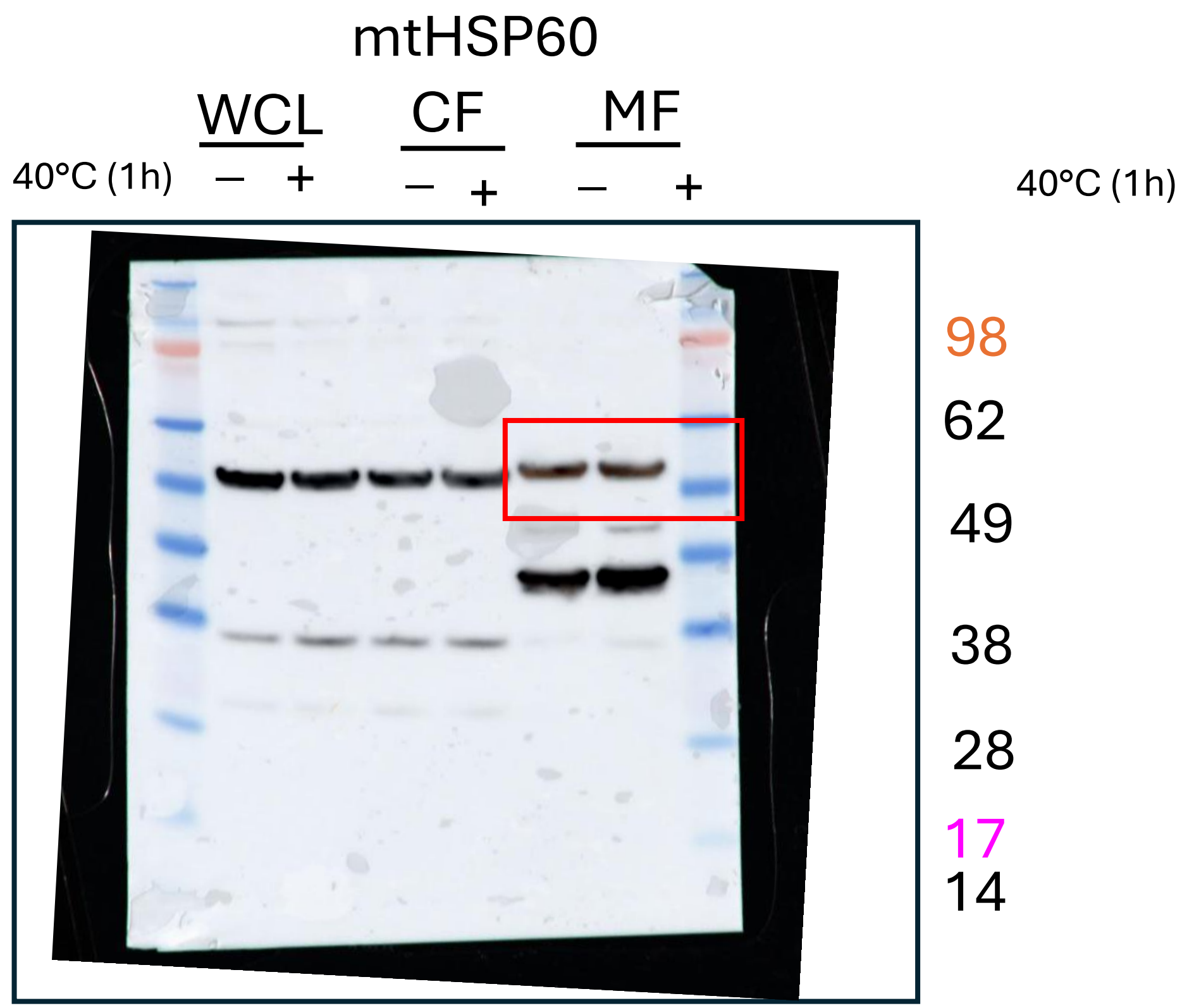

Bis-Tris 4-12% Gel
MES Buffer

WCL (Whole cell lysate)
CF (Cytosolic fraction)
MF (Mitochondrial fraction)
NF (Nuclear fraction)

Figure 1:
1A (Replicate 8,9, 10)

Mitochondrial fraction

HSPB5

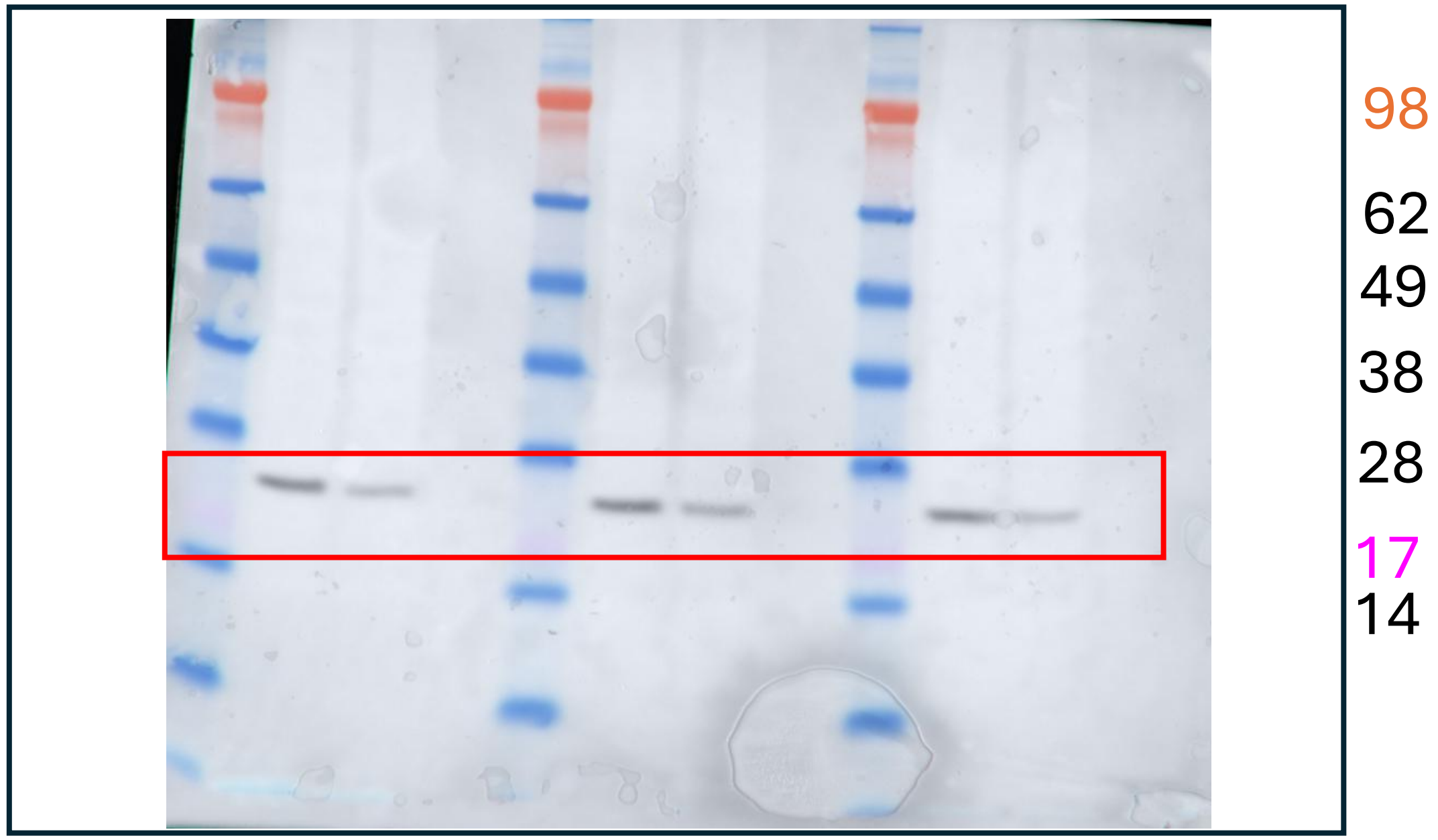

Bis-Tris 4-12% Gel
MES Buffer

Mitochondrial fraction

VDAC1

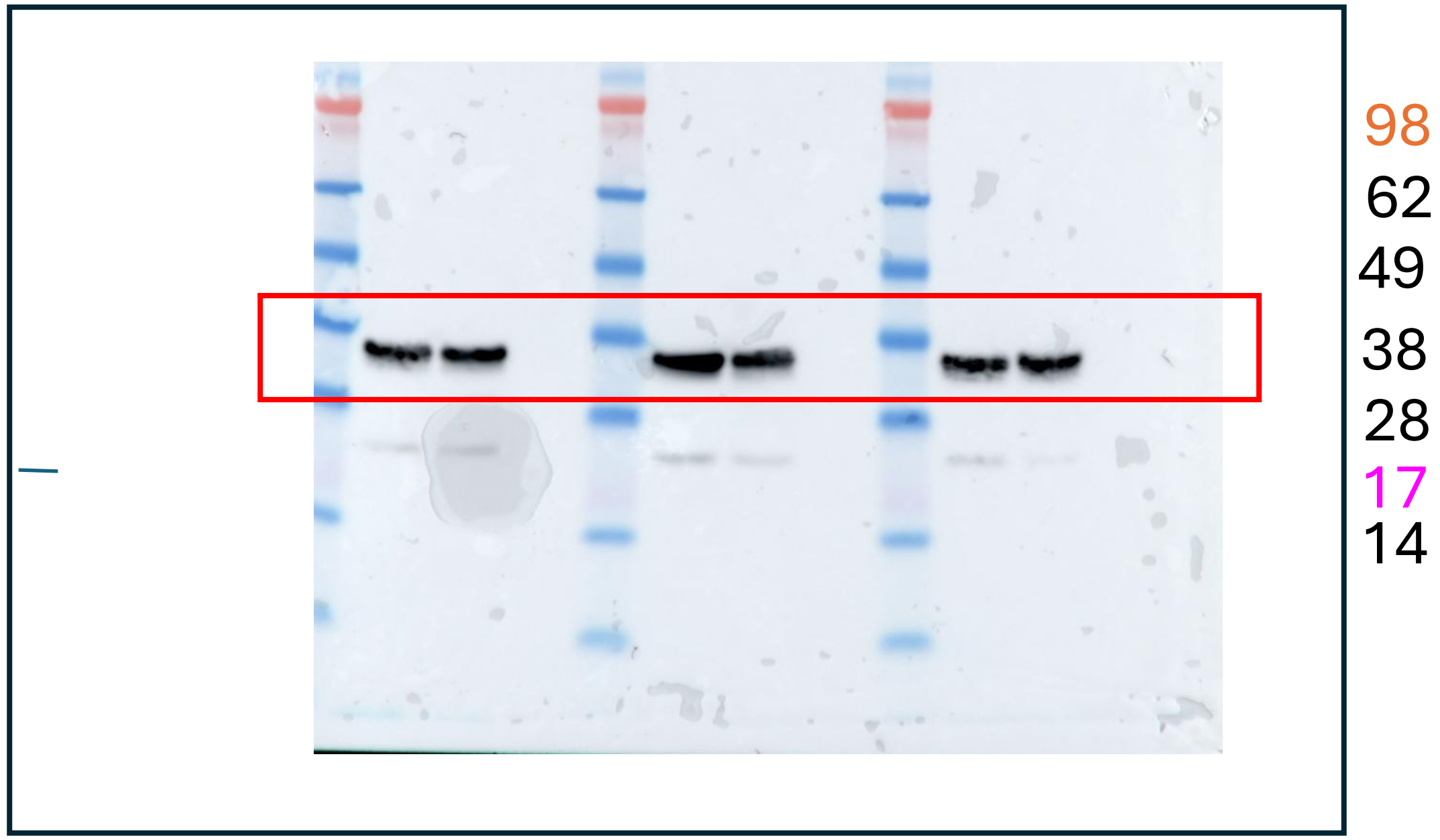

Bis-Tris 4-12% Gel
MES Buffer

Figure 1:
1A (Replicate 11 & 12)

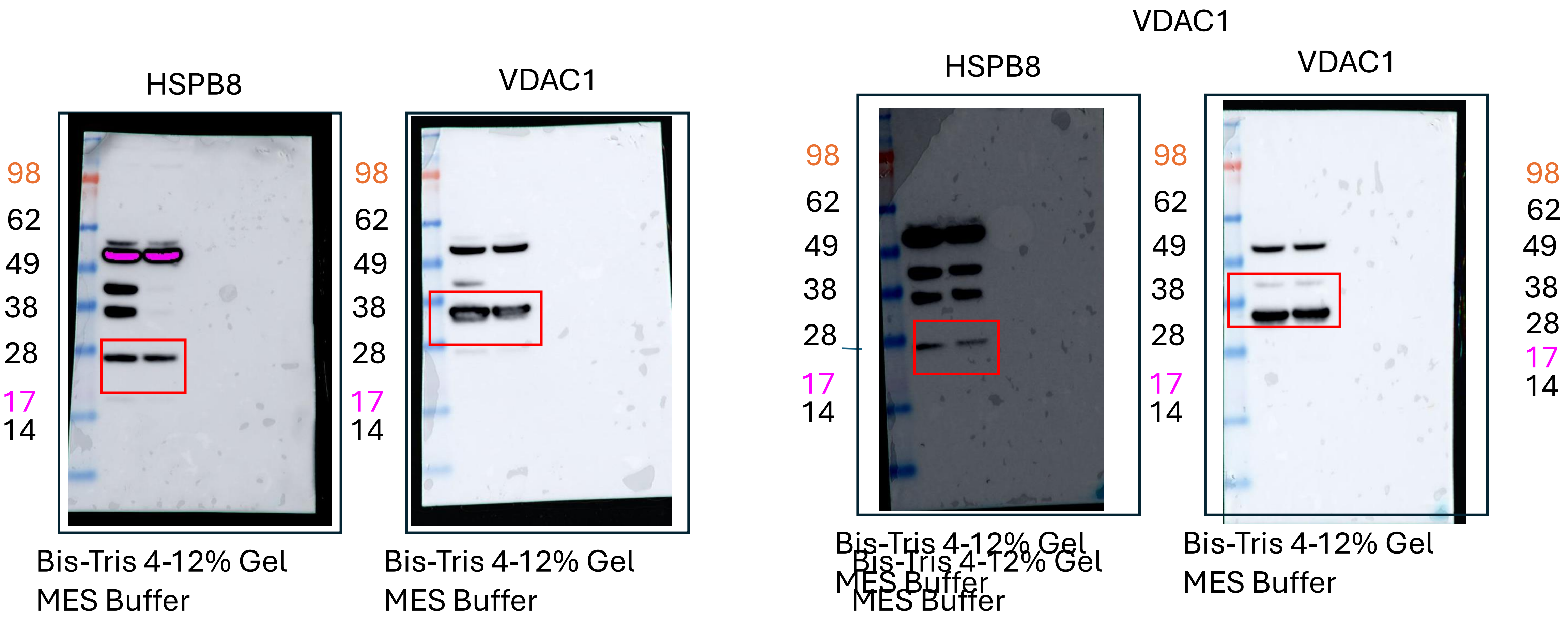

Figure 1:
1

HSPB1

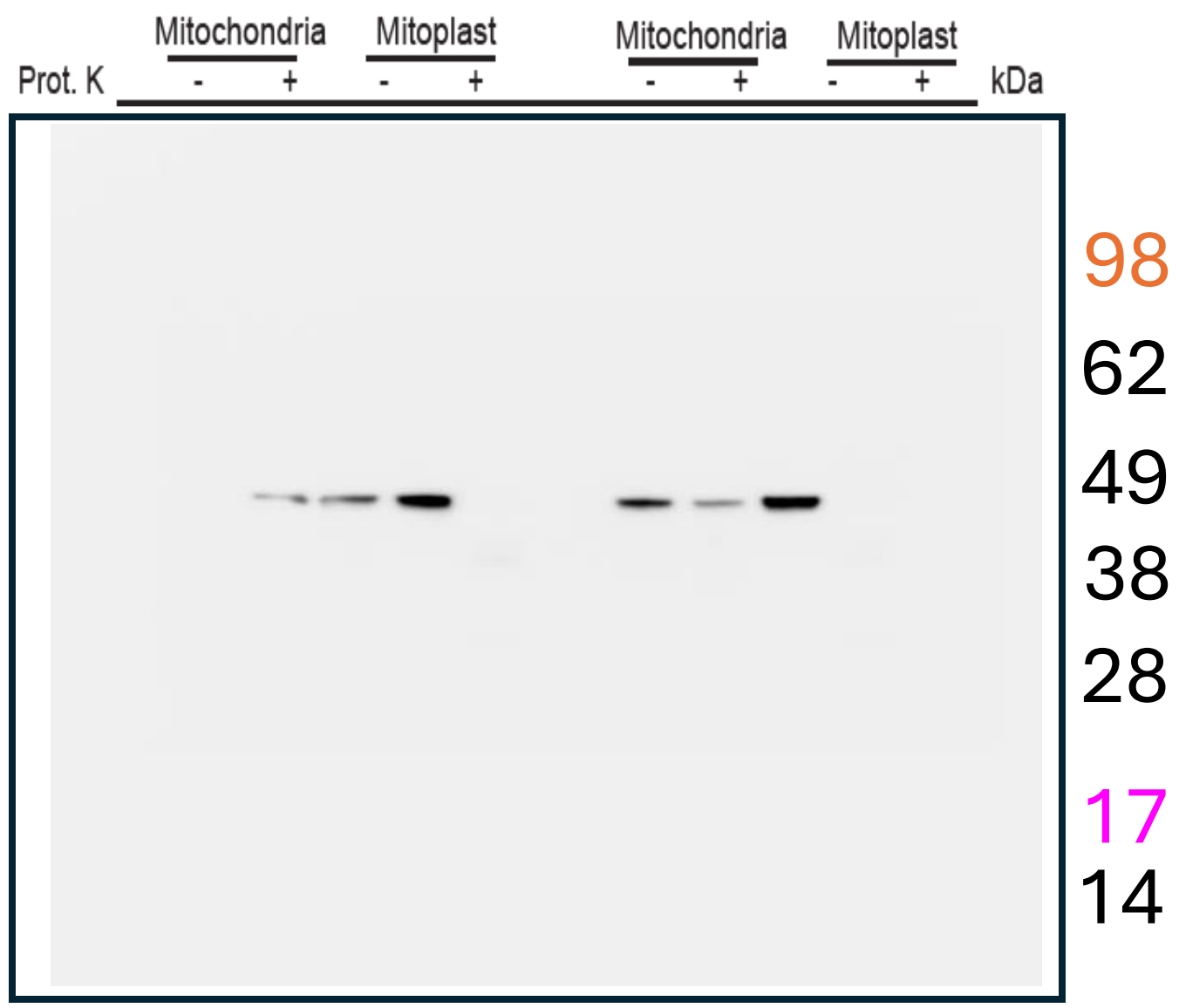

Bis-Tris 4-12% Gel
MES Buffer

Length markers of the same blot

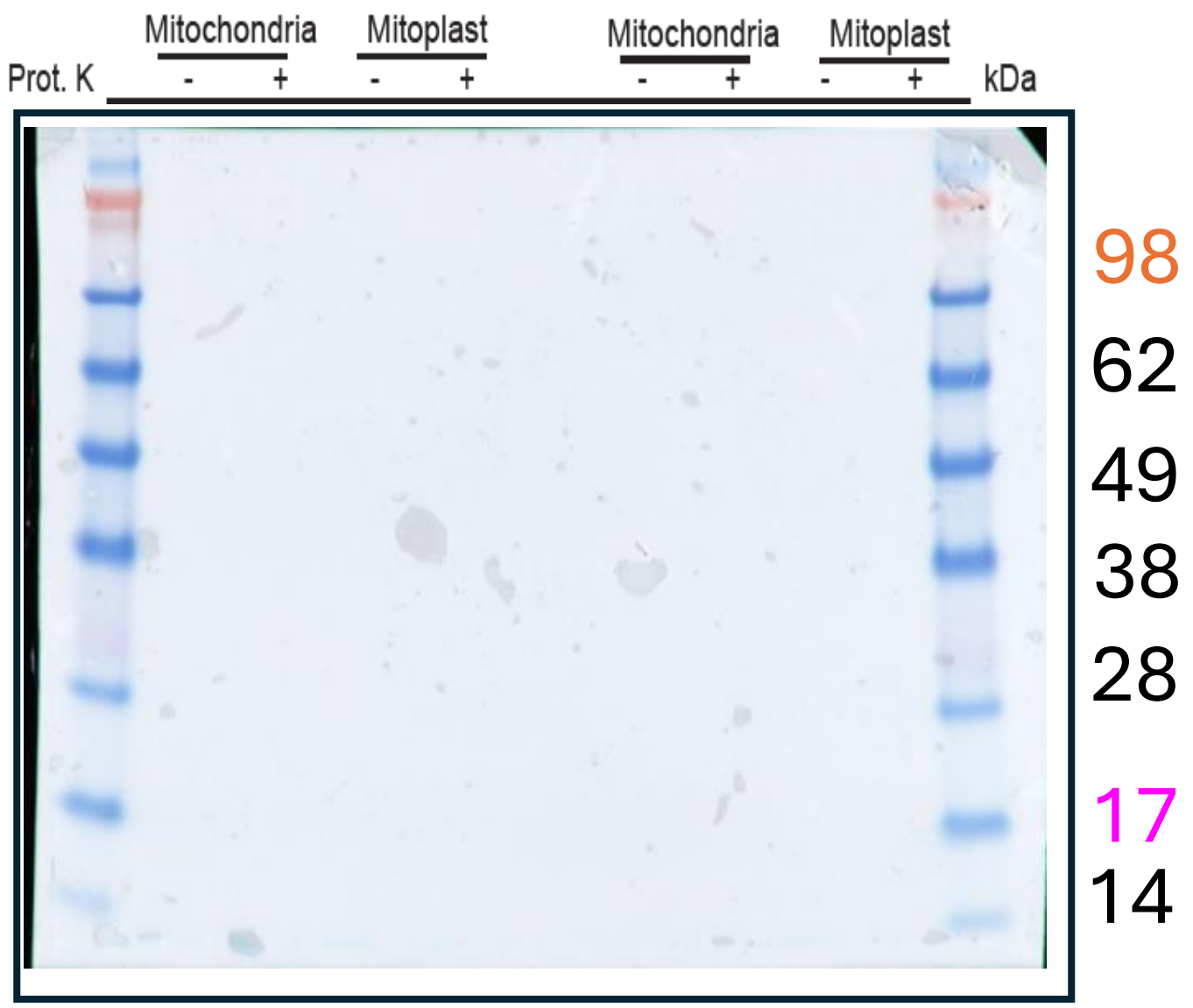

Bis-Tris 4-12% Gel
MES Buffer

For targets that could not be probed on the same membrane, the **same lysate set** was loaded on **separate SDS–PAGE gels and transferred to separate membranes**;

Figure 1:
1D (HSPB1)

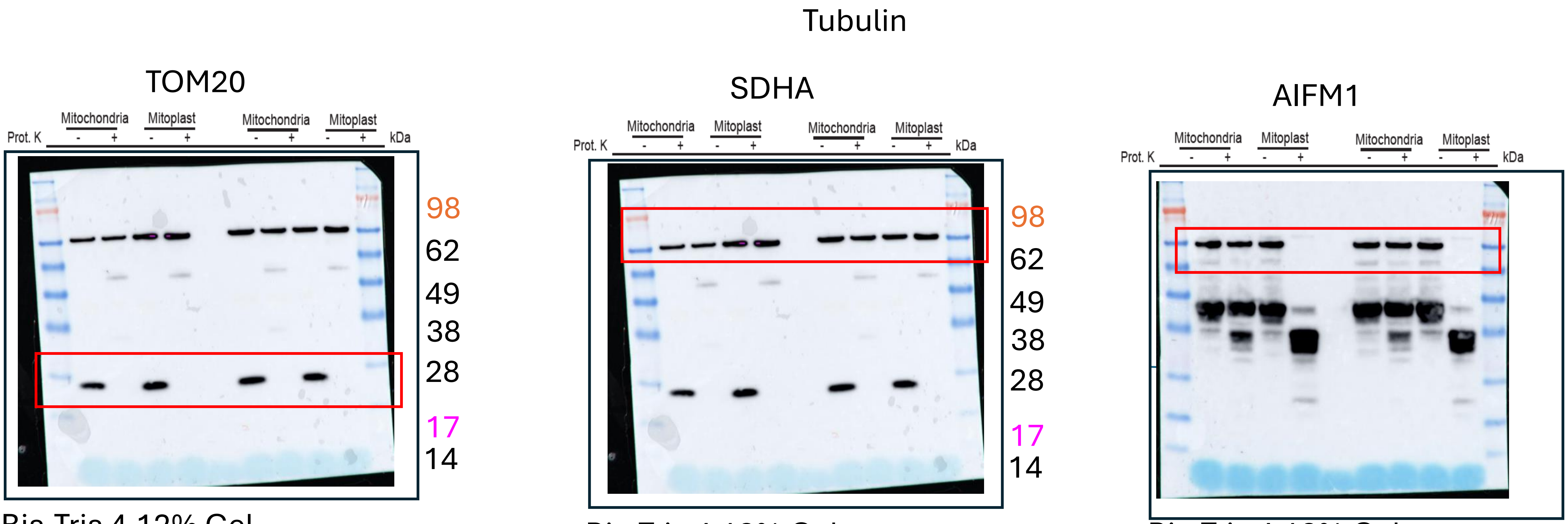

Bis-Tris 4-12% Gel
MES Buffer

Bis-Tris 4-12% Gel
MES Buffer

Bis-Tris 4-12% Gel
MES Buffer

For targets that could not be probed on the same membrane, the **same lysate set** was loaded on **separate SDS–PAGE gels and transferred to separate membranes**;

Figure 1:
1D (HSPB1)

HCCS

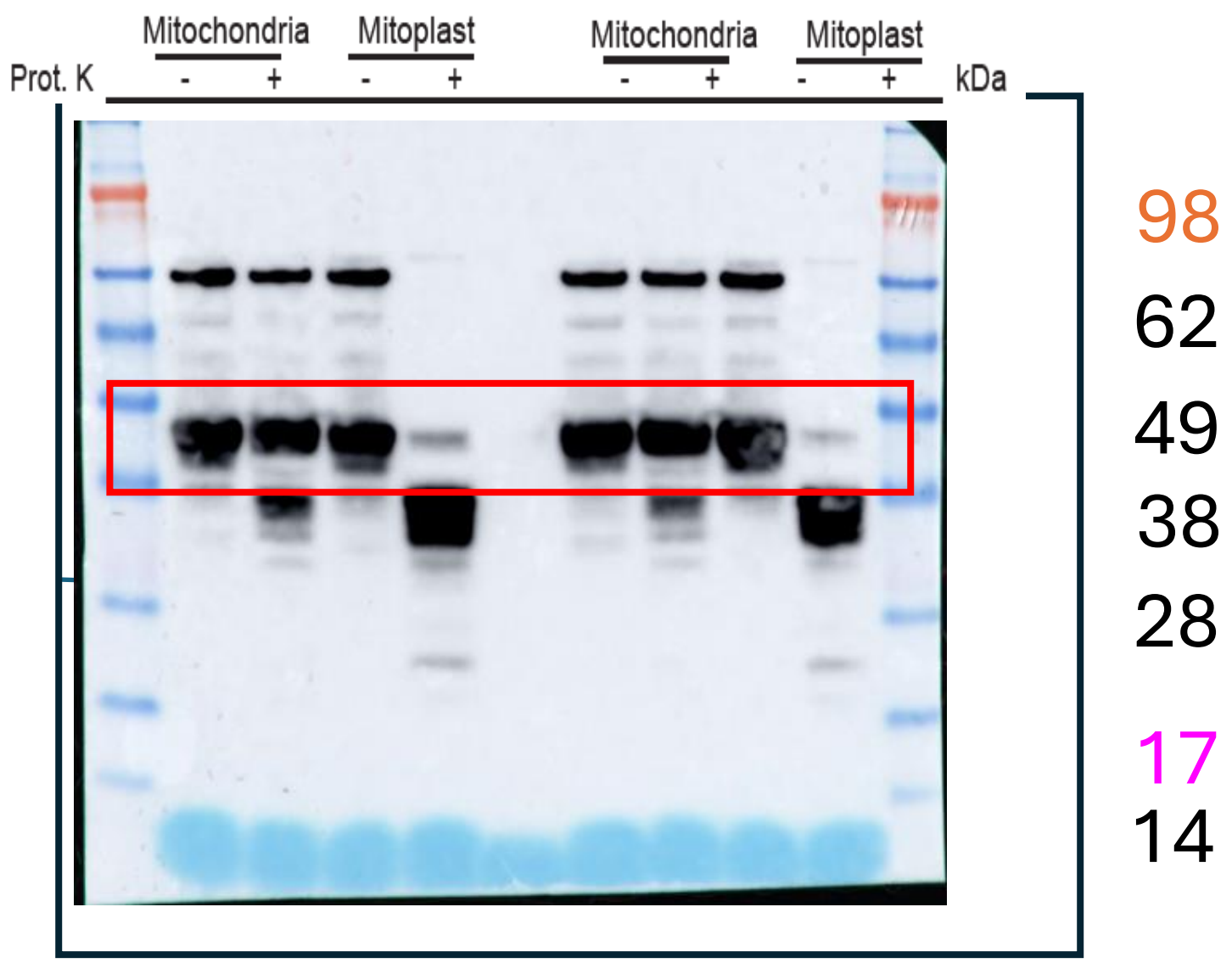

Bis-Tris 4-12% Gel
MES Buffer

For targets that could not be probed on the same membrane, the **same lysate set** was loaded on **separate SDS–PAGE gels and transferred to separate membranes**;

Figure 2:
2D (Replicate 1)

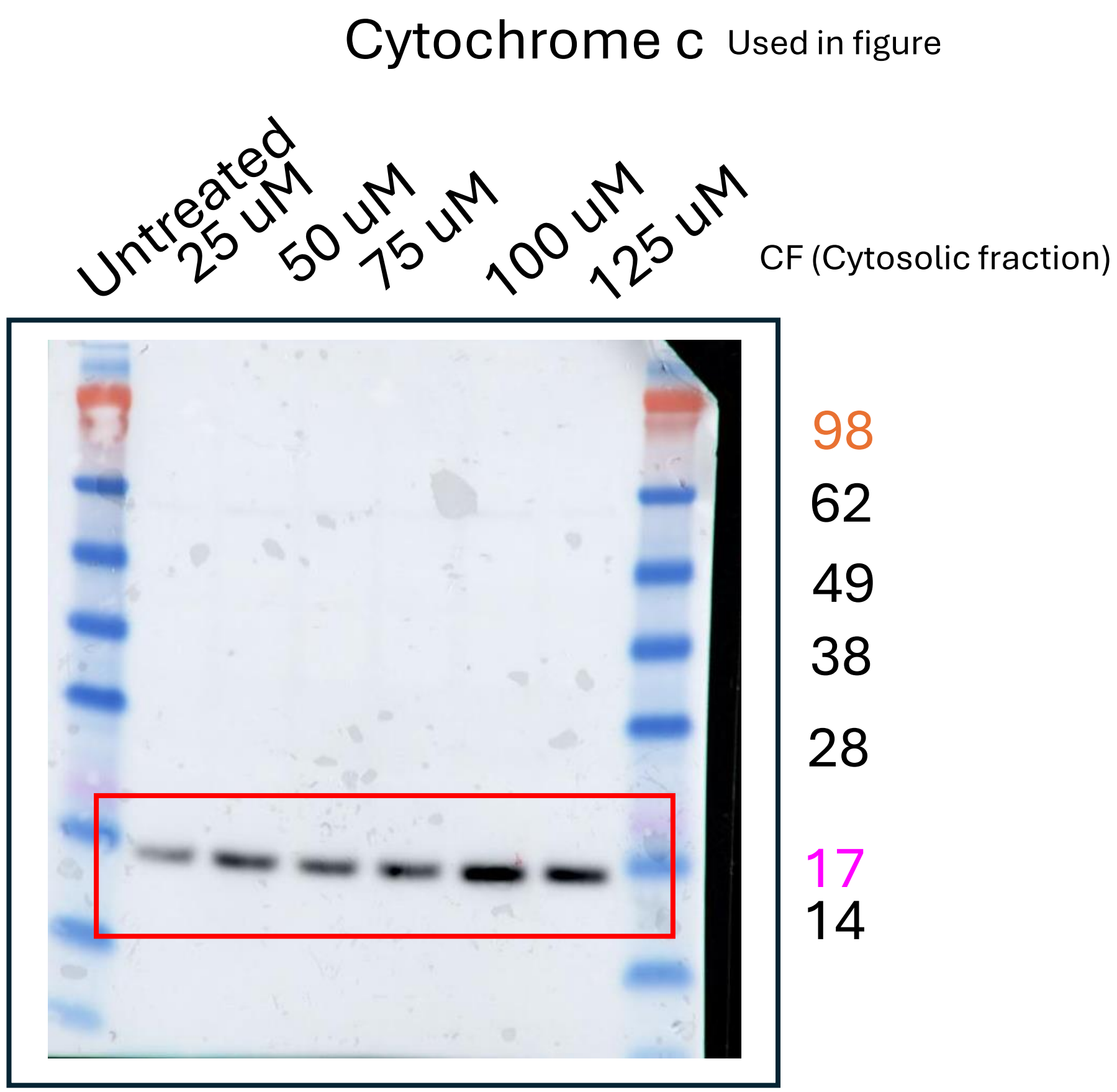

Bis-Tris 4-12% Gel
MES Buffer

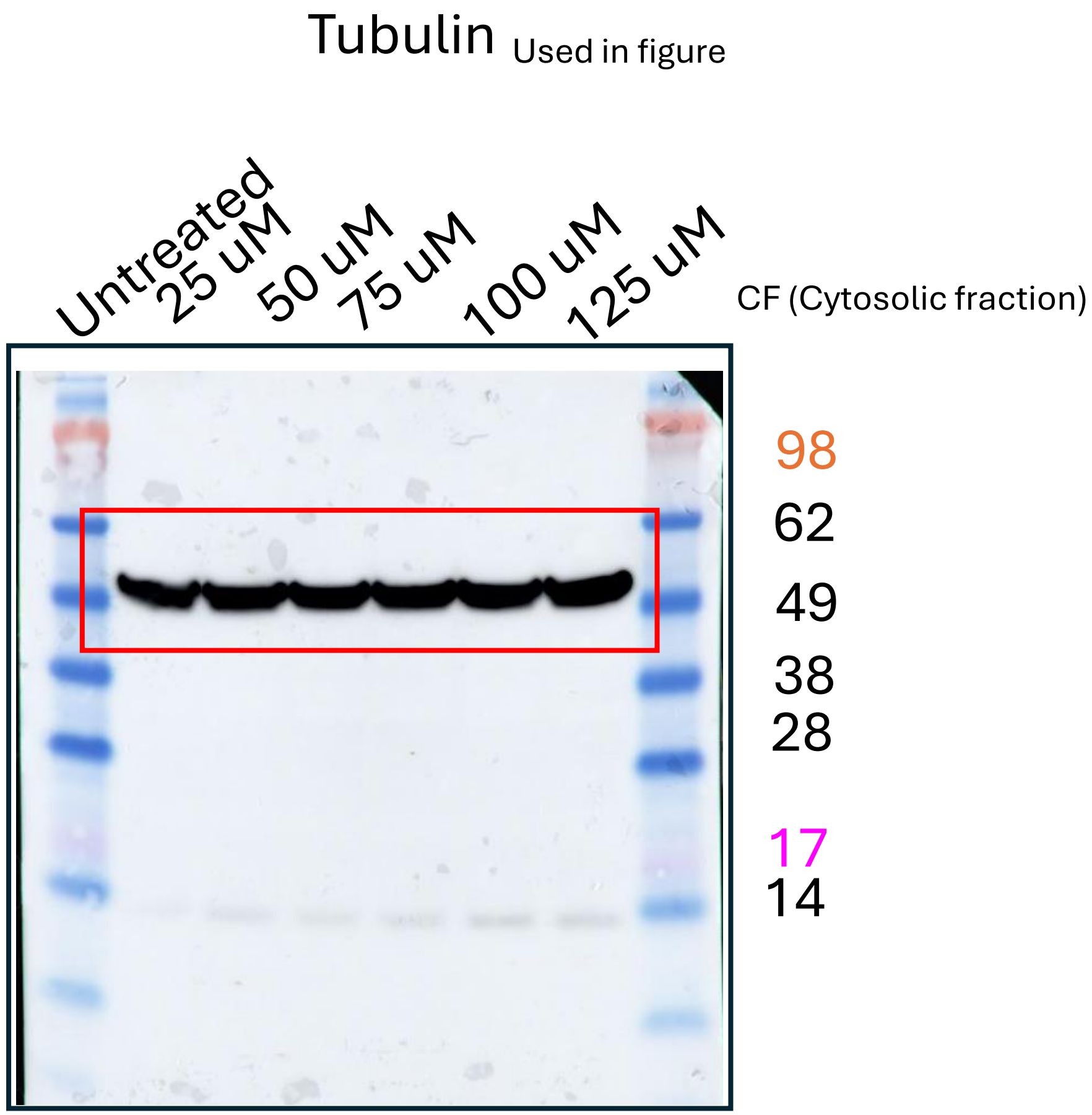

Bis-Tris 4-12% Gel
MES Buffer

Figure 2:
2D (Replicate 2)

Cytochrome c

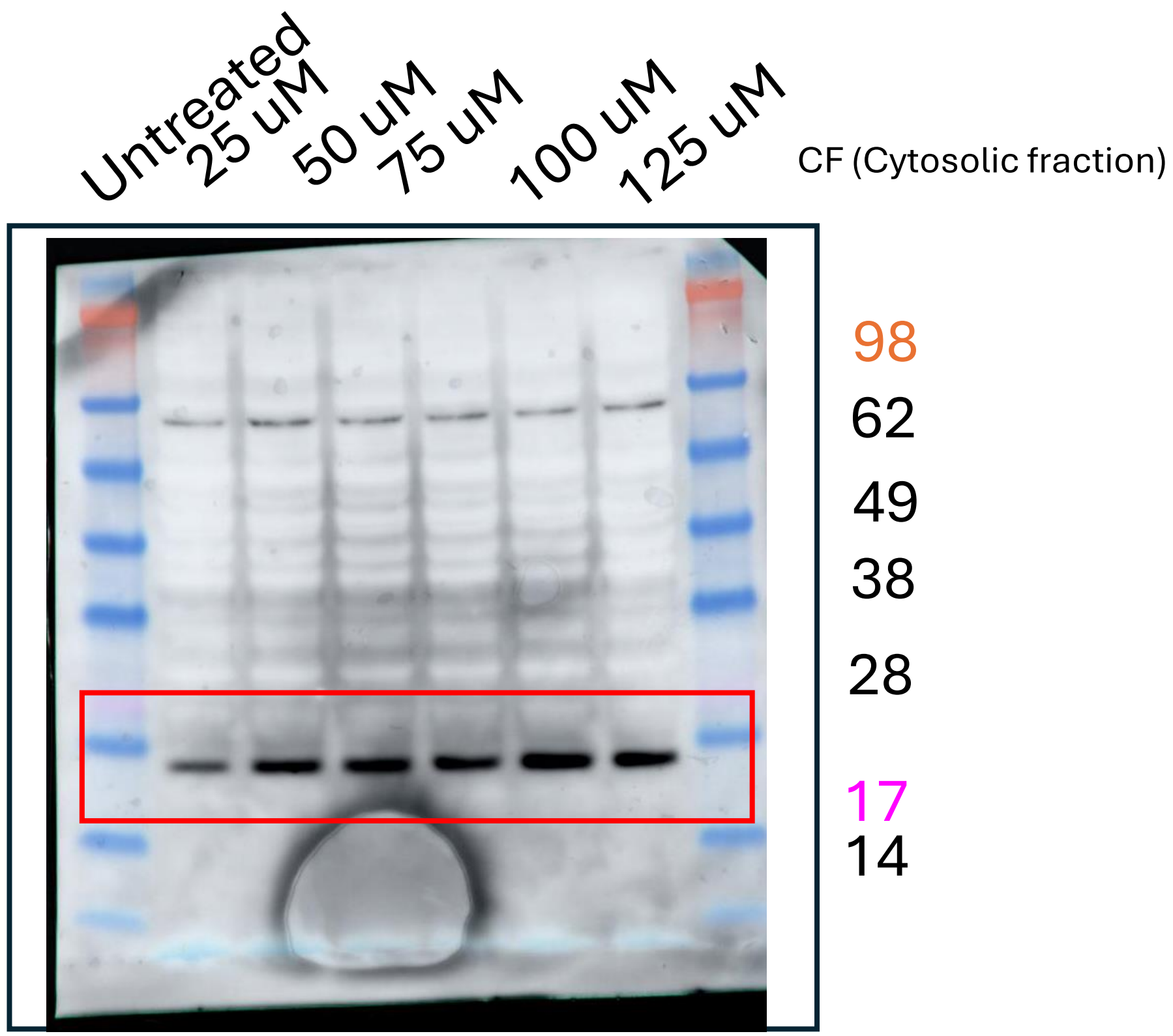

Bis-Tris 4-12% Gel
MES Buffer

Tubulin

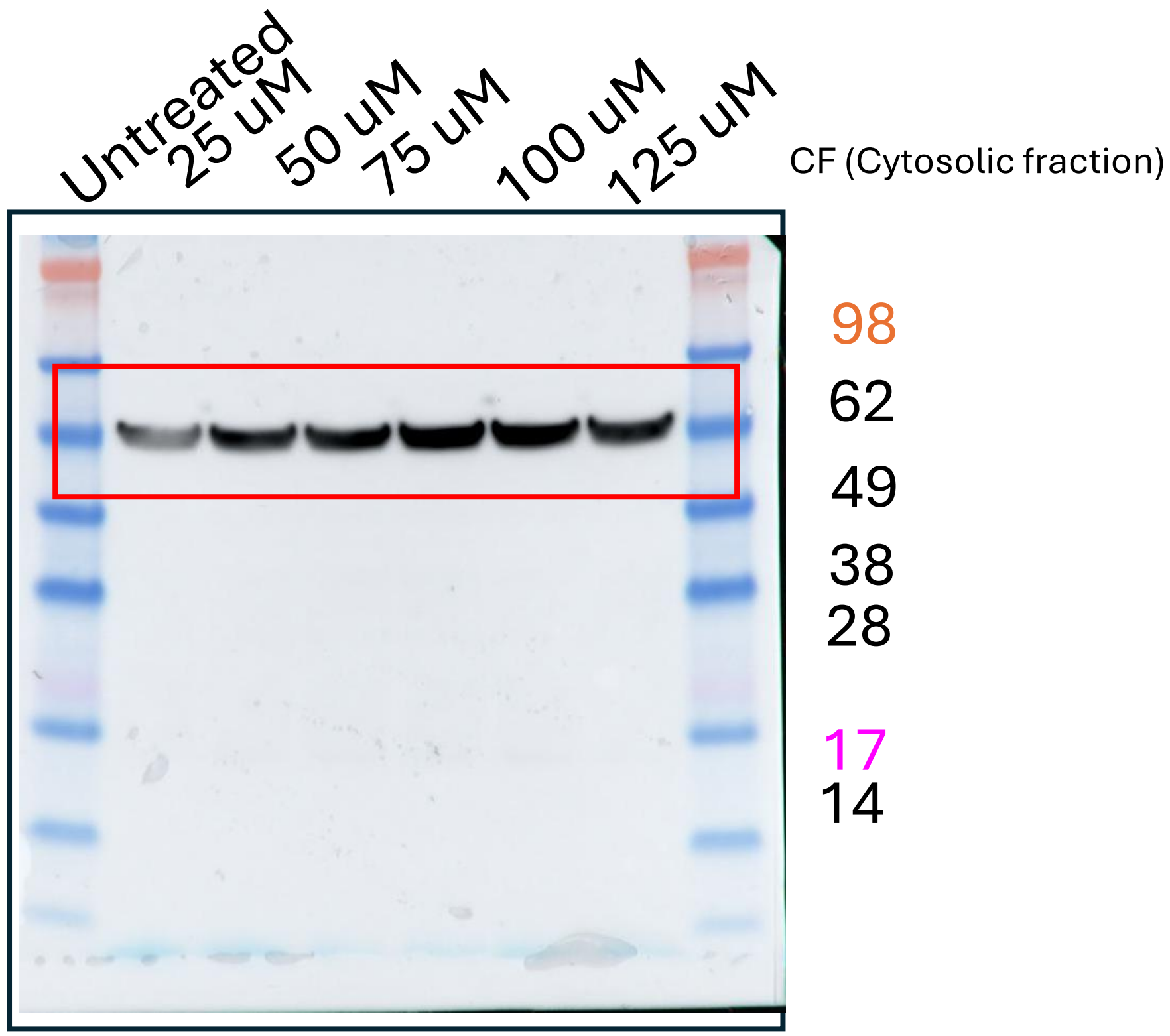

Bis-Tris 4-12% Gel
MES Buffer

Figure 2:
2D (Replicate 3)

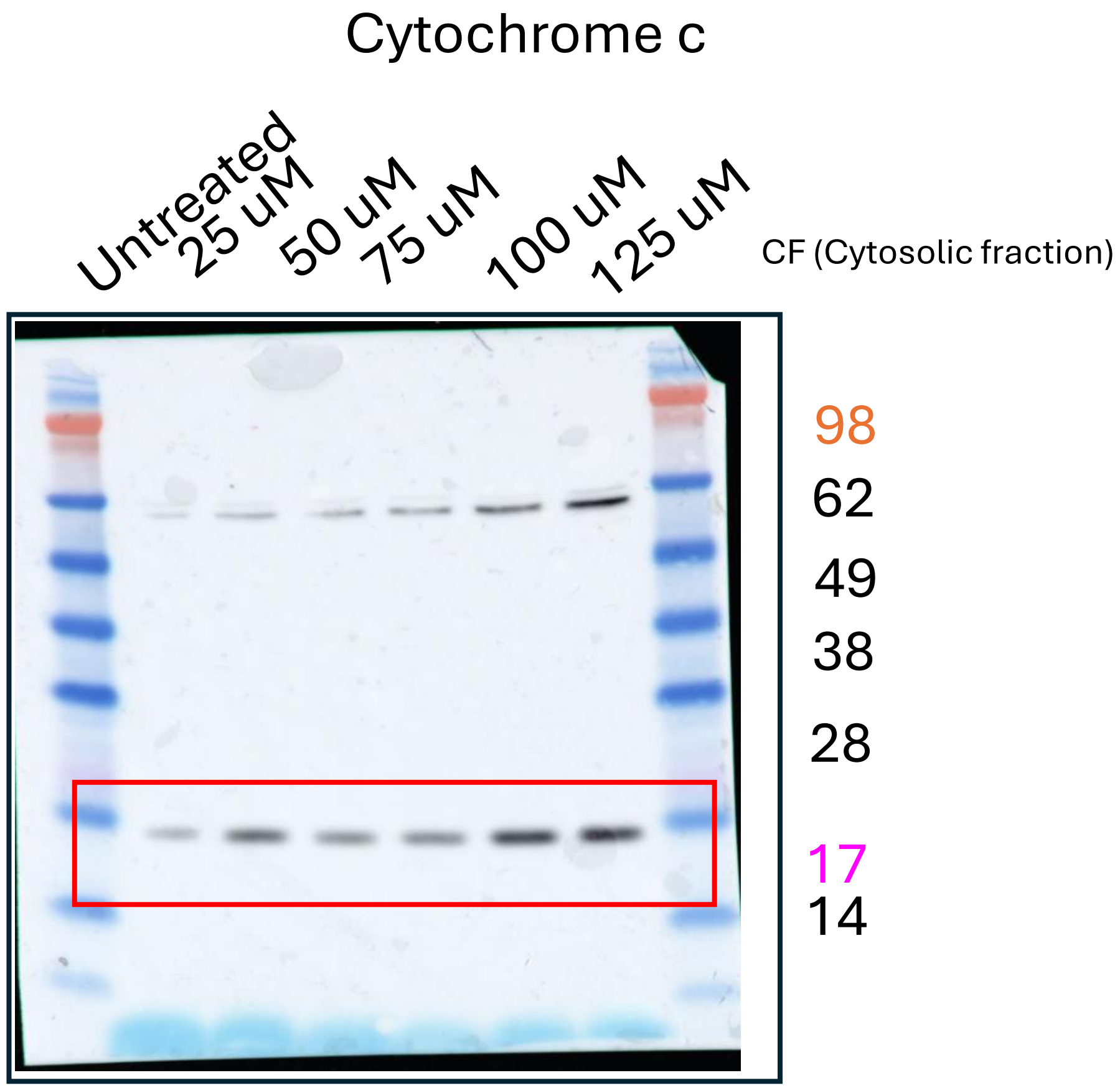

Bis-Tris 4-12% Gel
MES Buffer

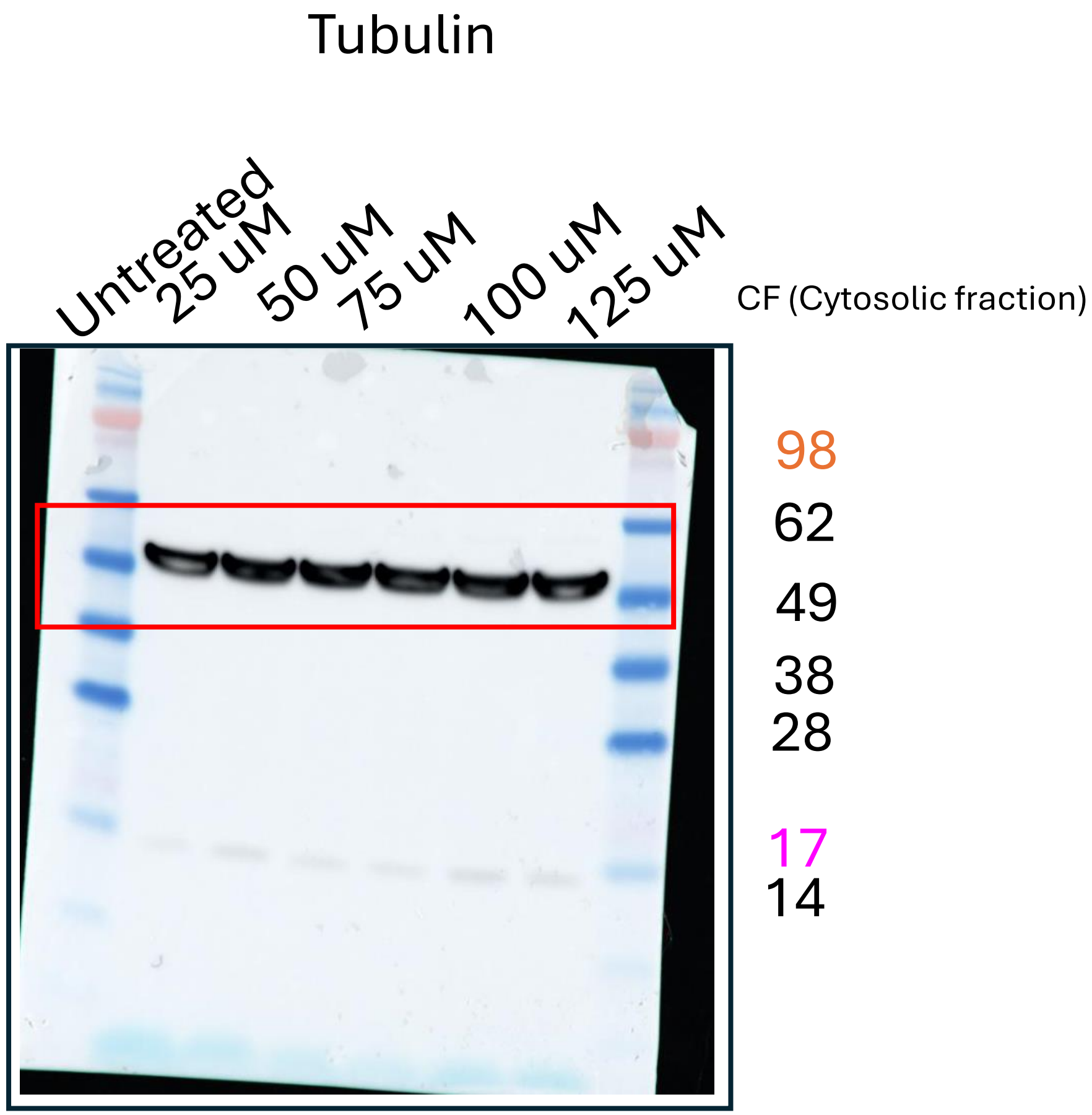

Bis-Tris 4-12% Gel
MES Buffer

Figure 2:
2F (Replicate 1)

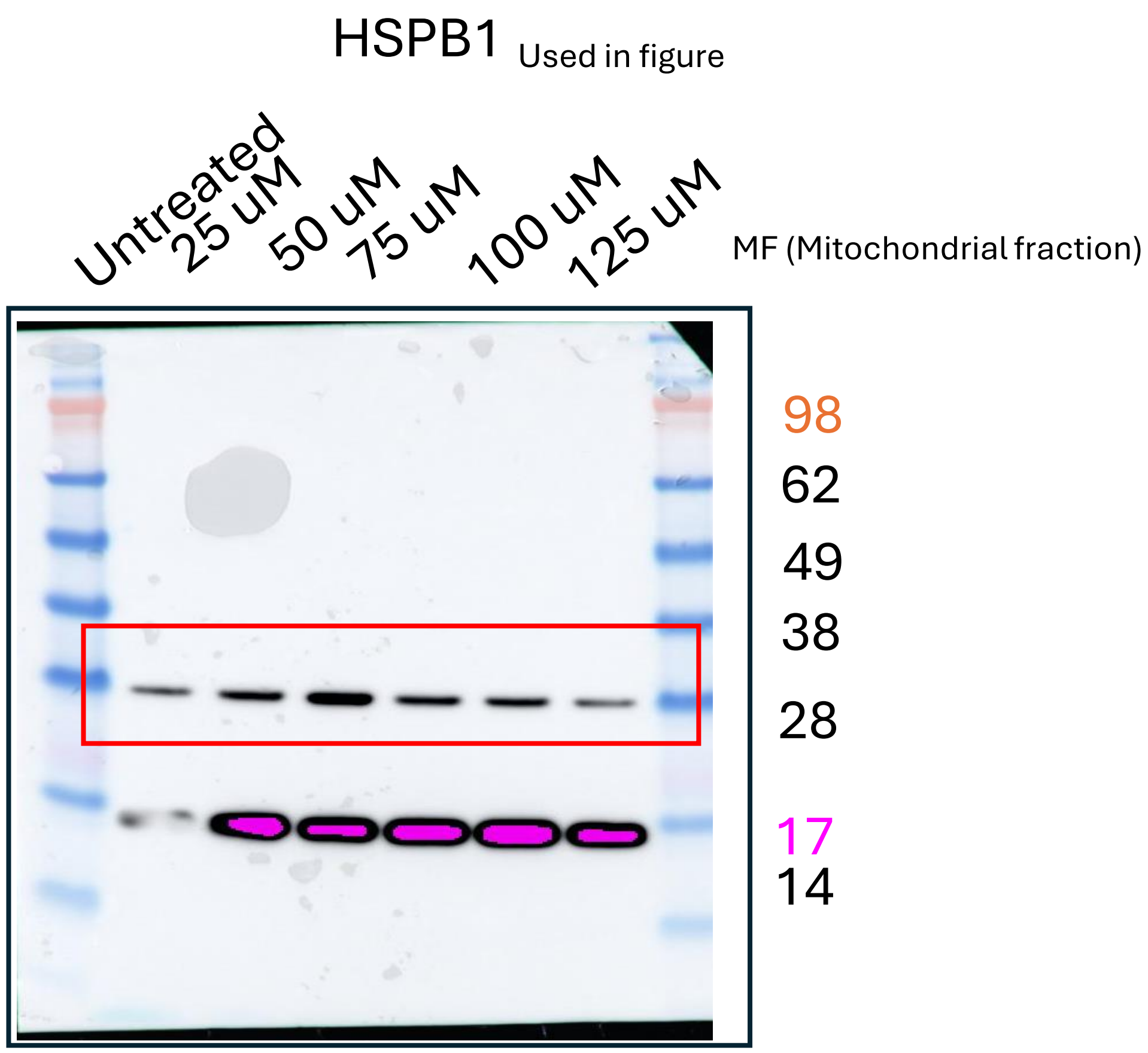

Bis-Tris 4-12% Gel
MES Buffer

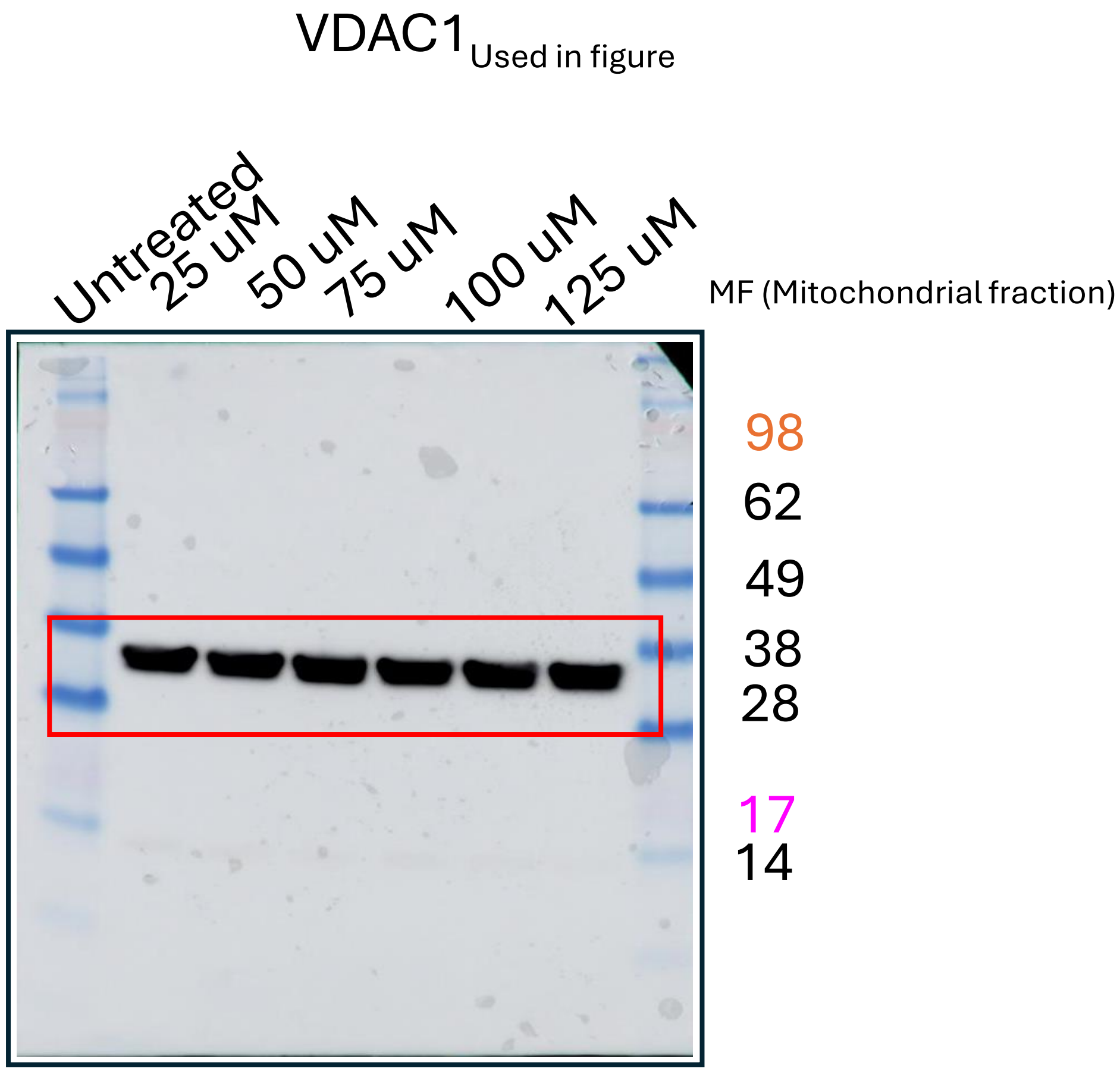

Bis-Tris 4-12% Gel
MES Buffer

Figure 2:
2F (Replicate 2)

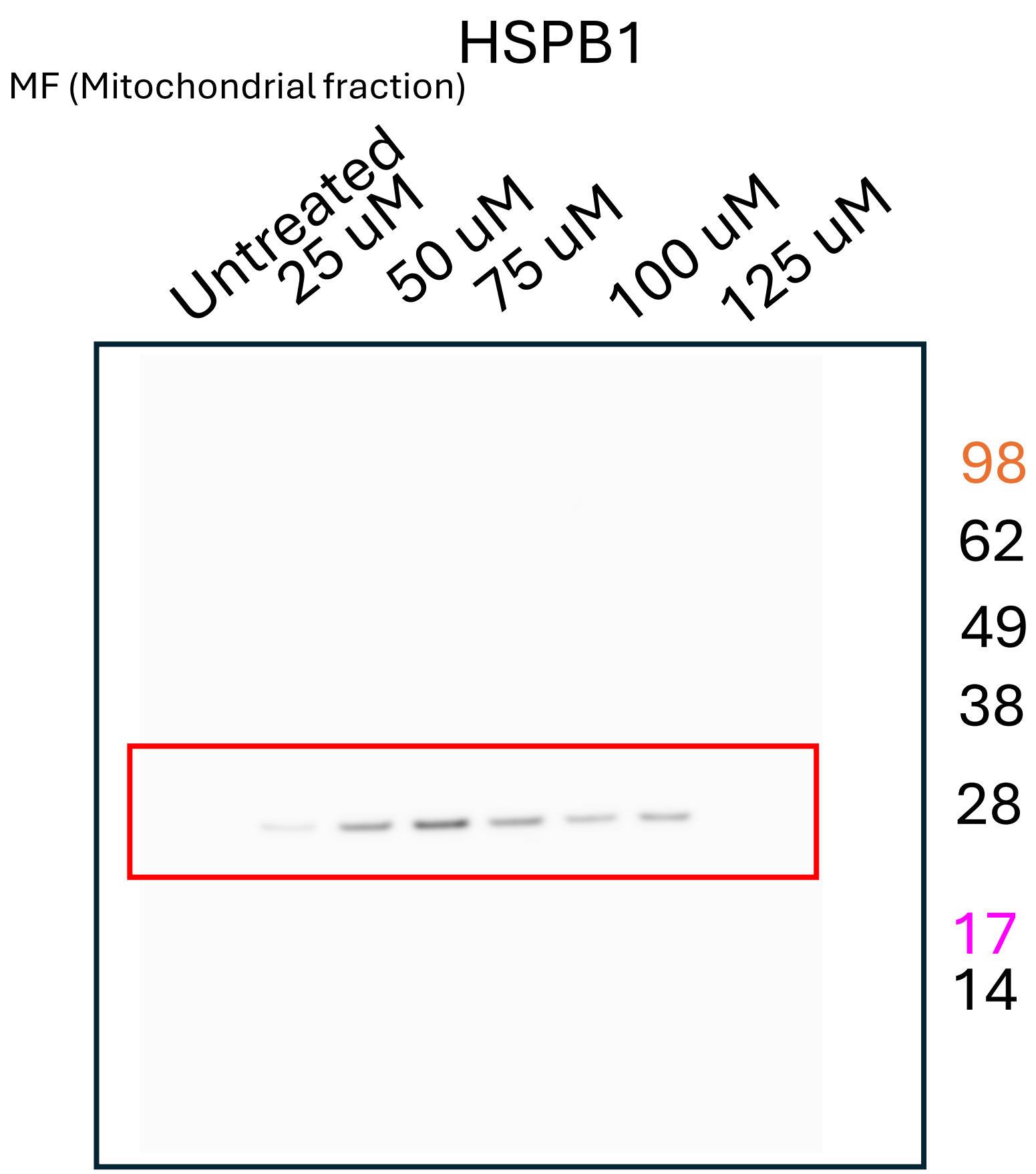

Bis-Tris 4-12% Gel
MES Buffer

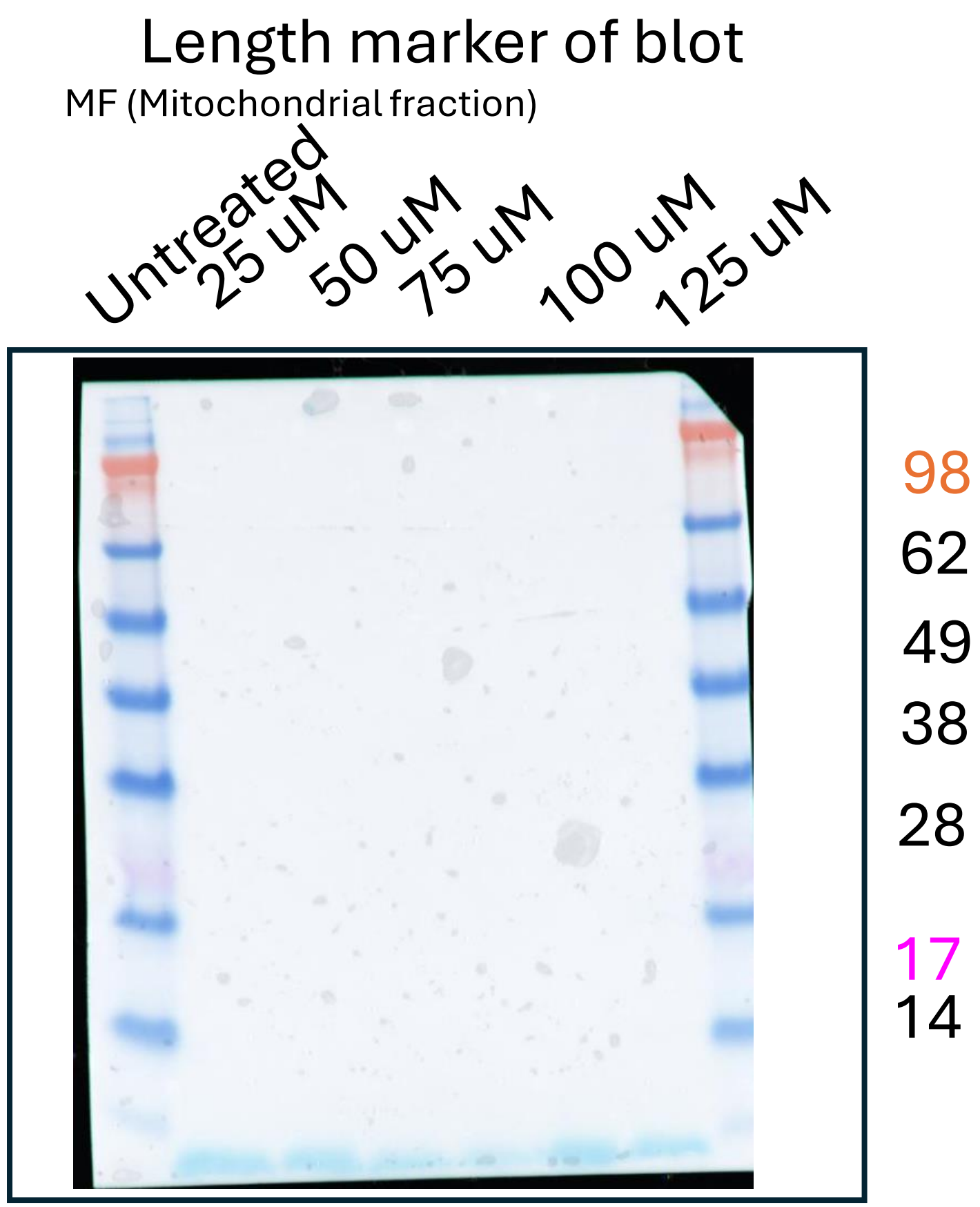

Bis-Tris 4-12% Gel
MES Buffer

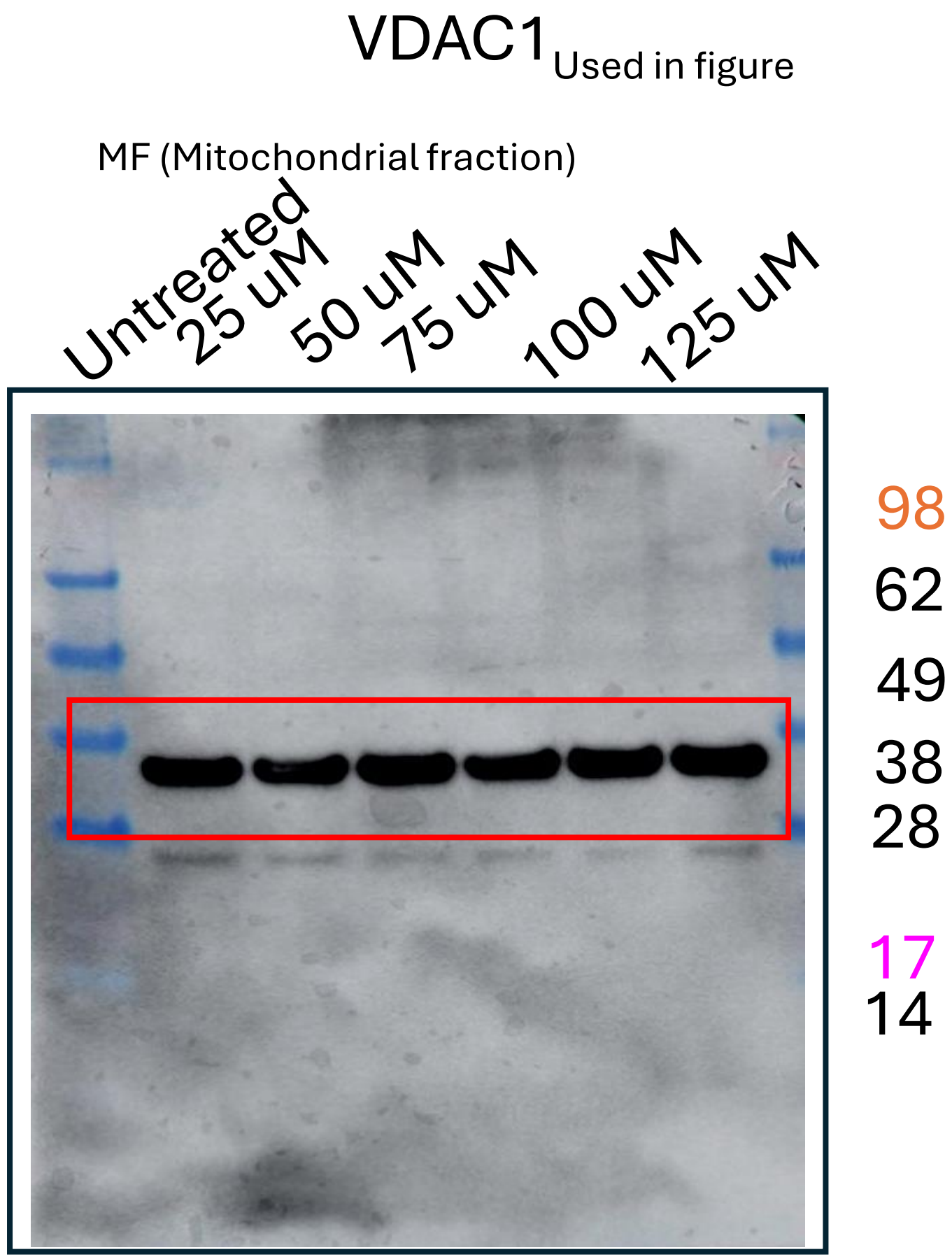

Bis-Tris 4-12% Gel
MES Buffer

Figure 2:
2F (Replicate 3)

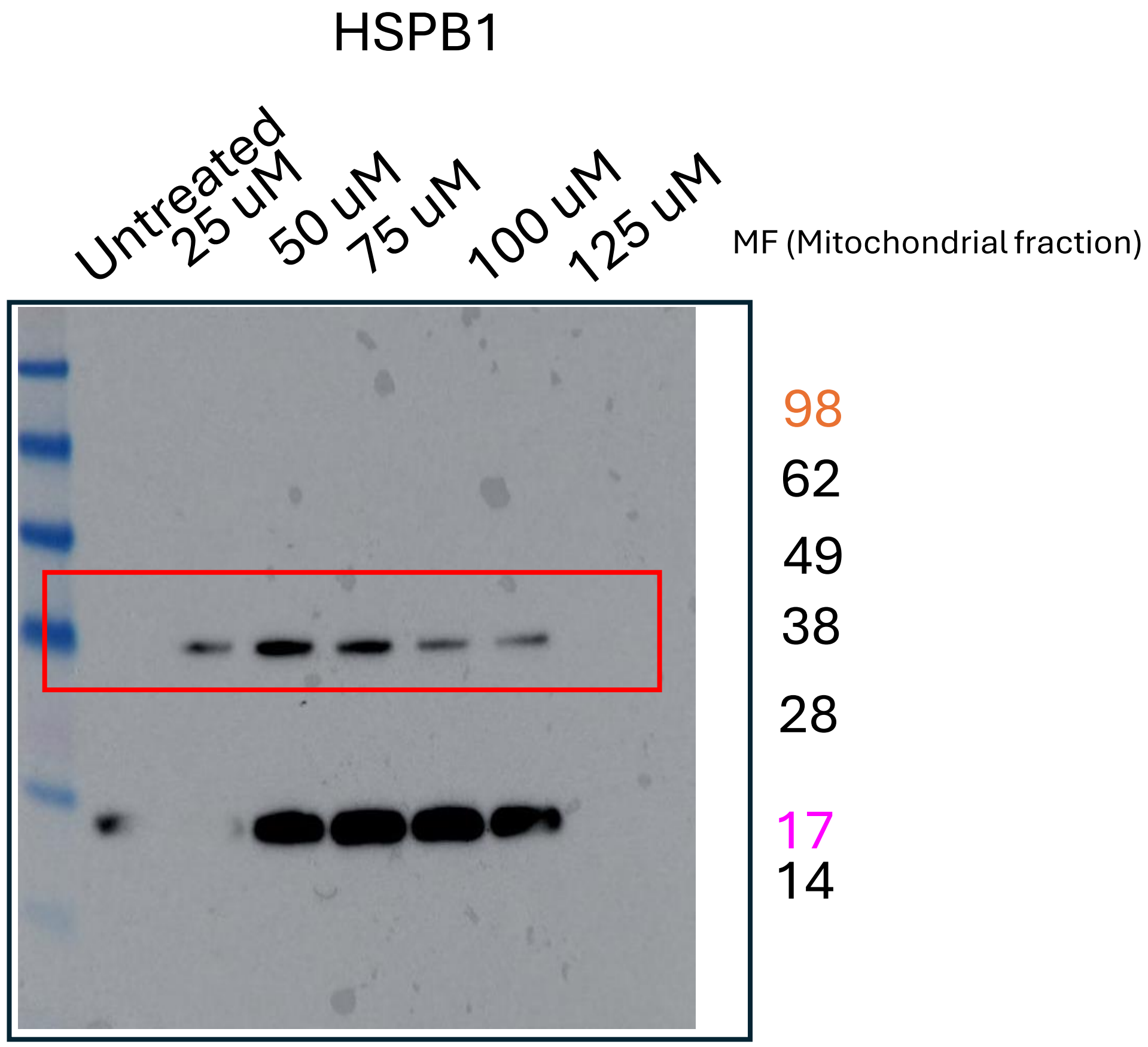

Bis-Tris 4-12% Gel
MES Buffer

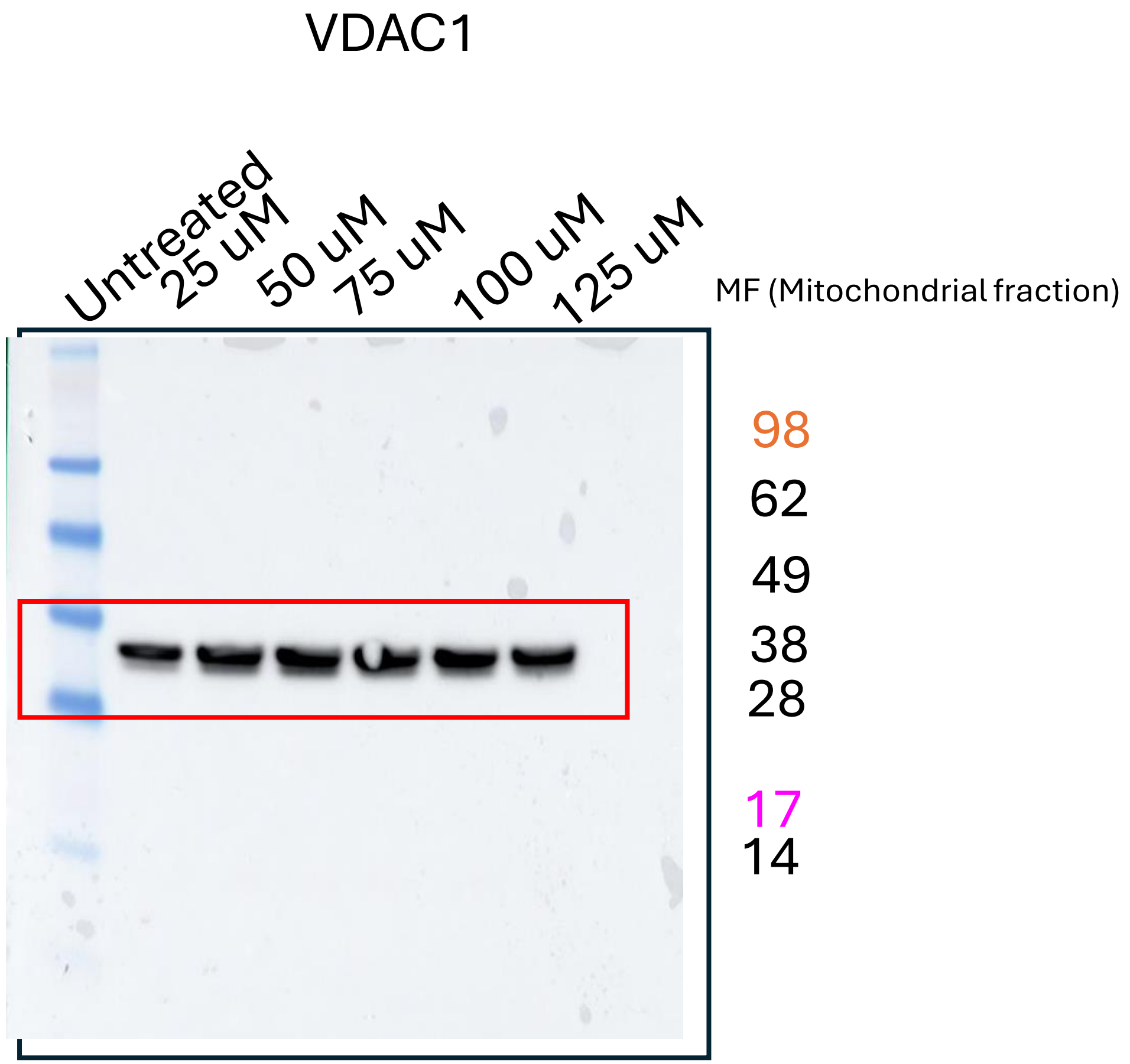

Bis-Tris 4-12% Gel
MES Buffer

Figure 2:
2F (Replicate 4)

HSPB5 Used in figure

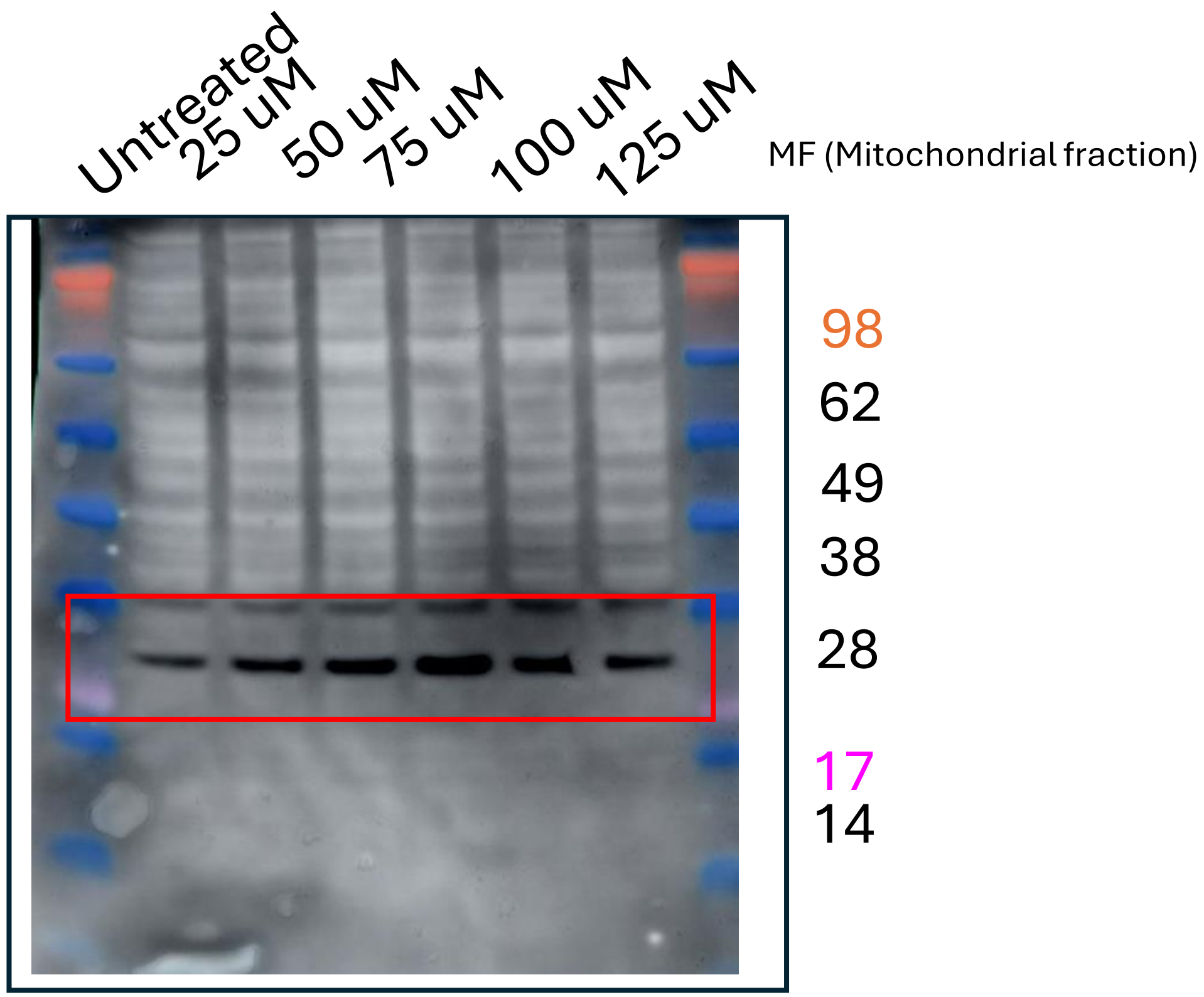

Bis-Tris 4-12% Gel
MES Buffer

VDAC1

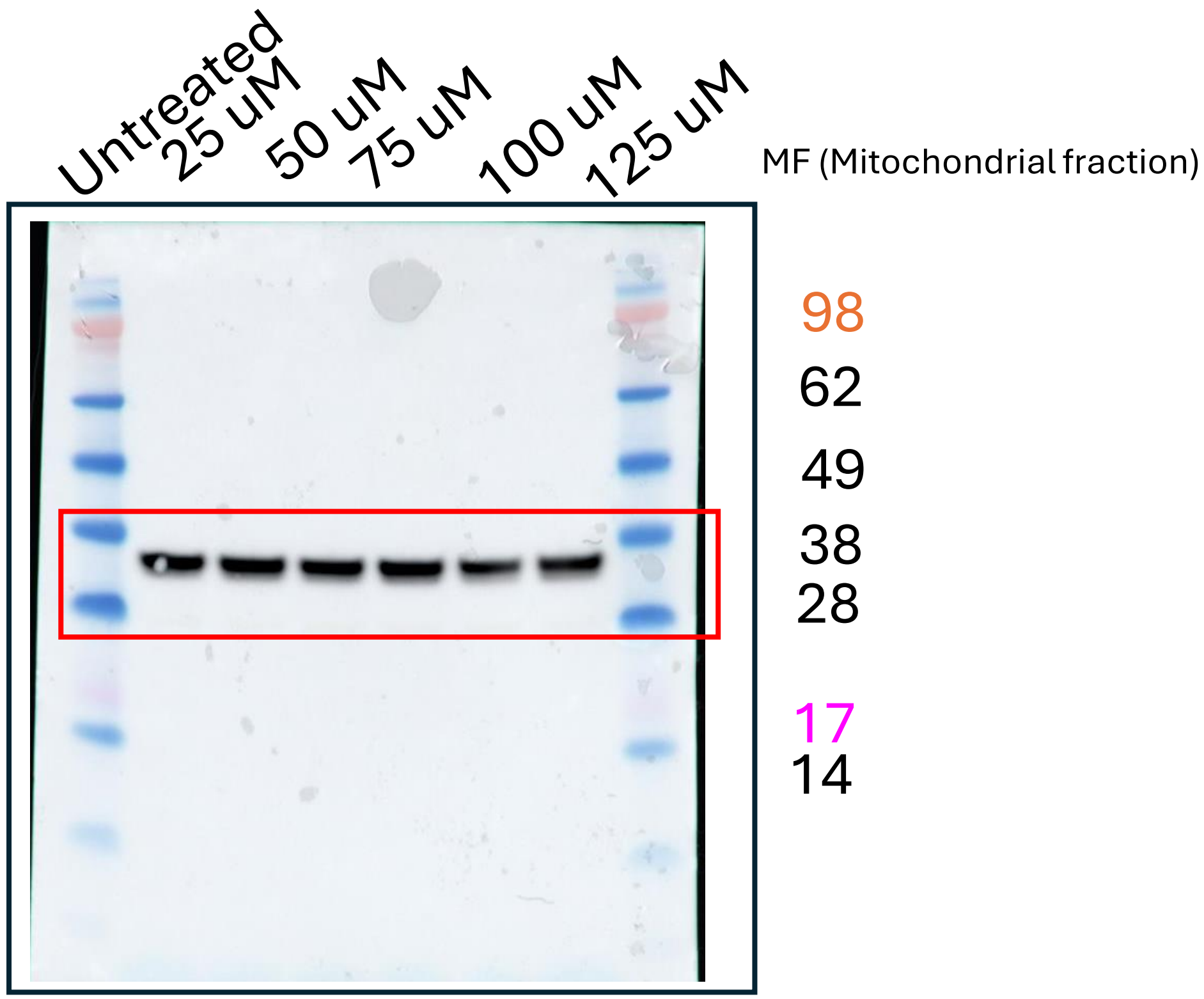

Bis-Tris 4-12% Gel
MES Buffer

Figure 2:
2F (Replicate 5)

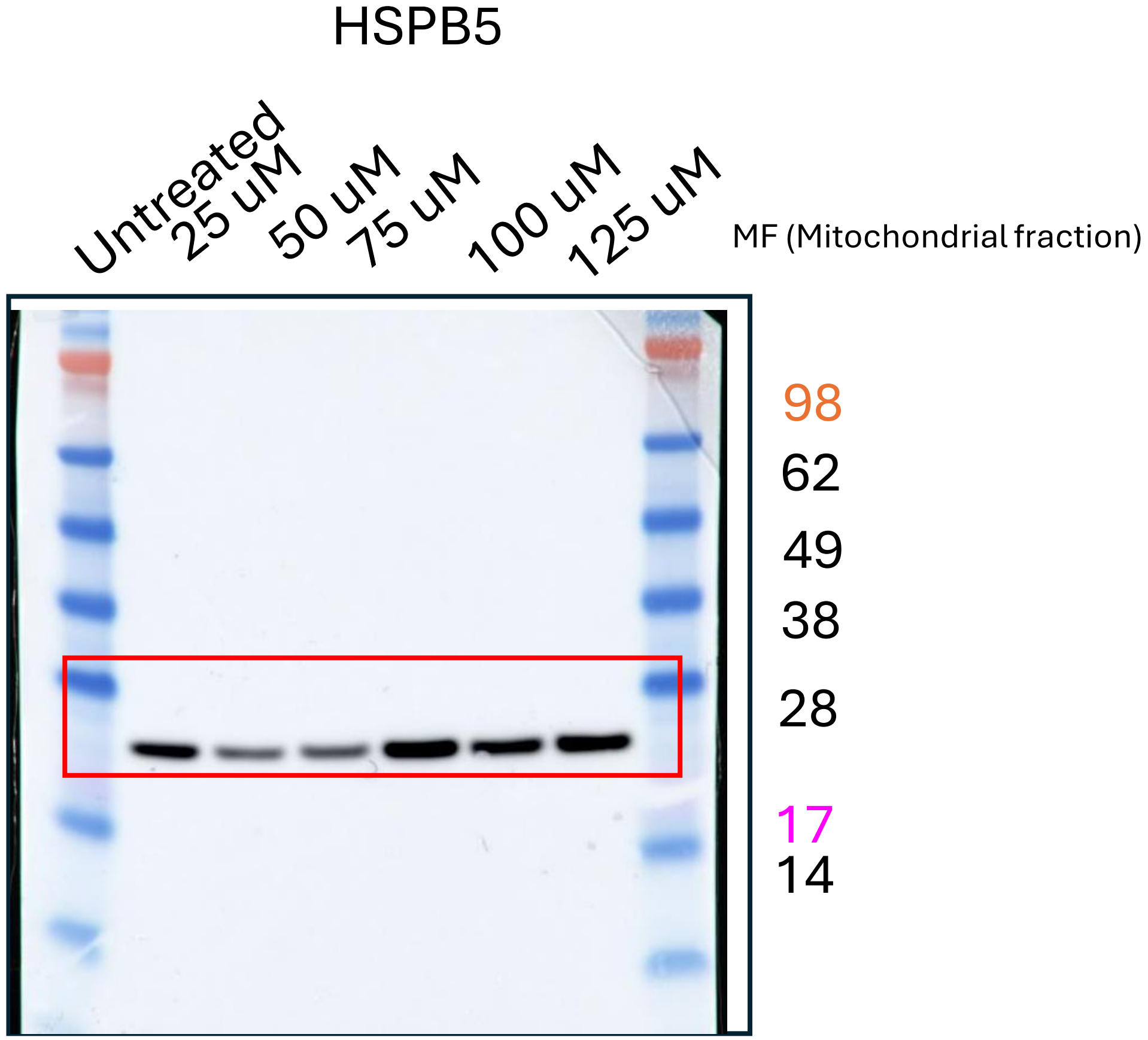

Bis-Tris 4-12% Gel
MES Buffer

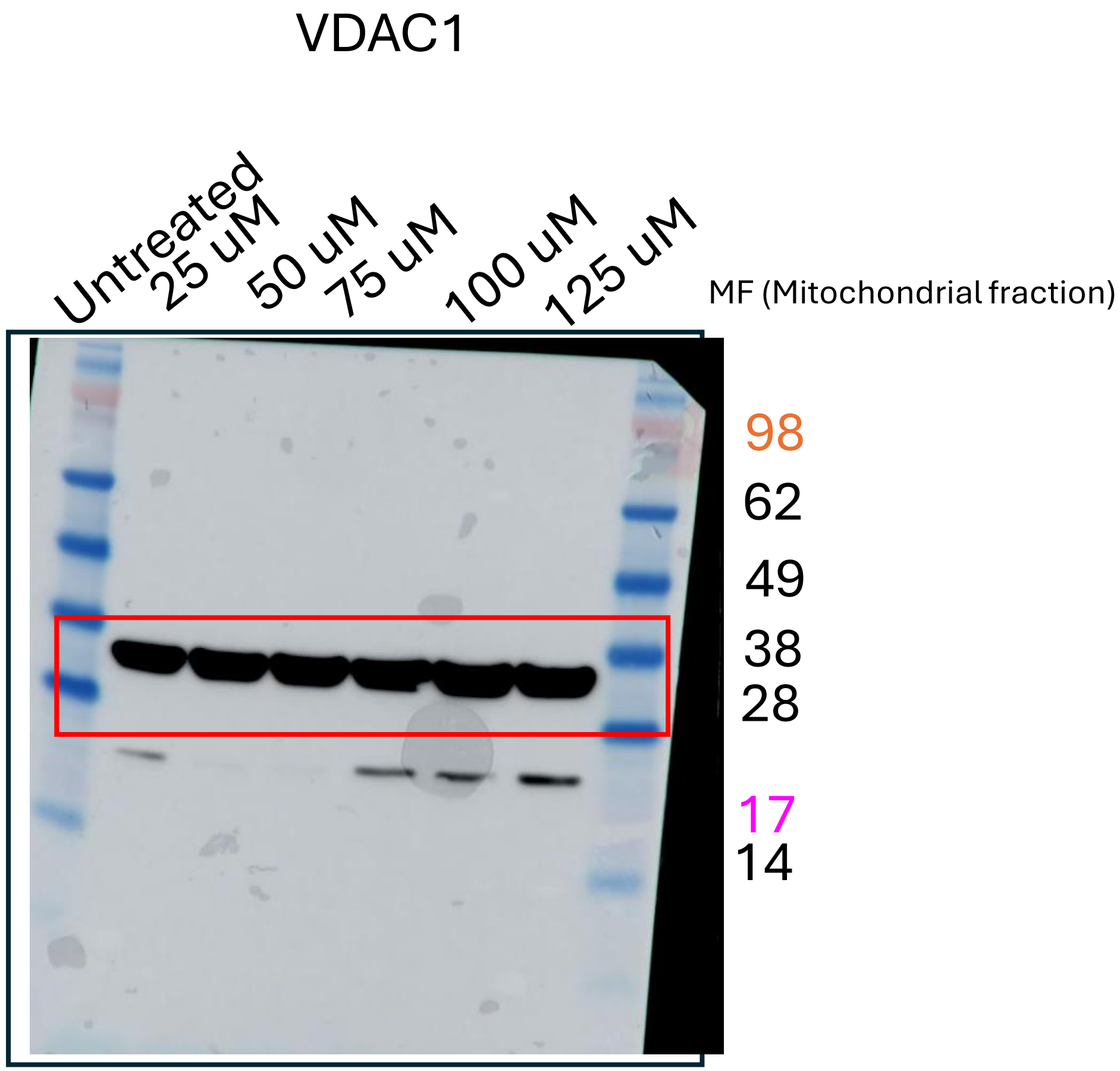

Bis-Tris 4-12% Gel
MES Buffer

Figure 2:
2F (Replicate 6)

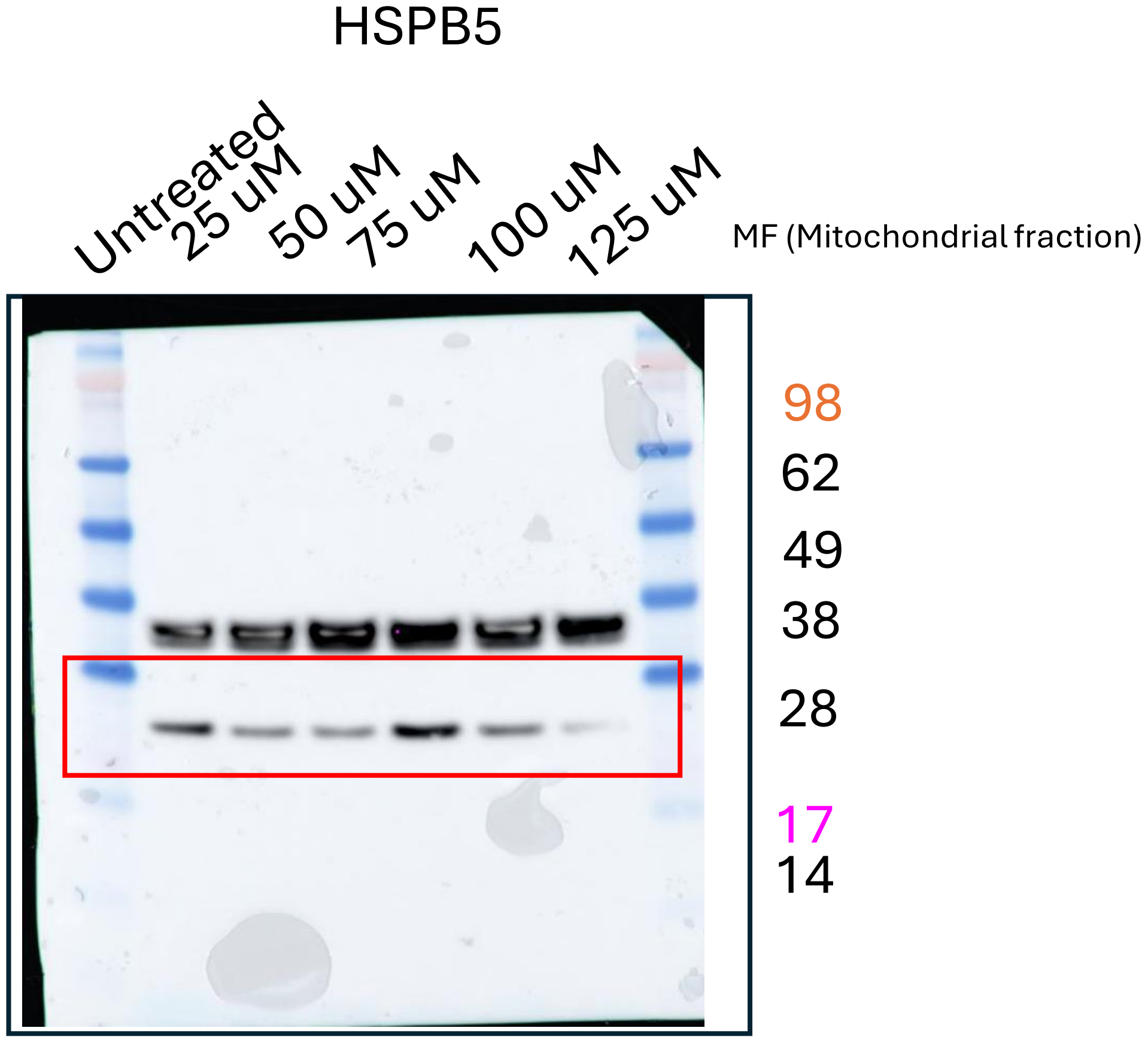

Bis-Tris 4-12% Gel
MES Buffer

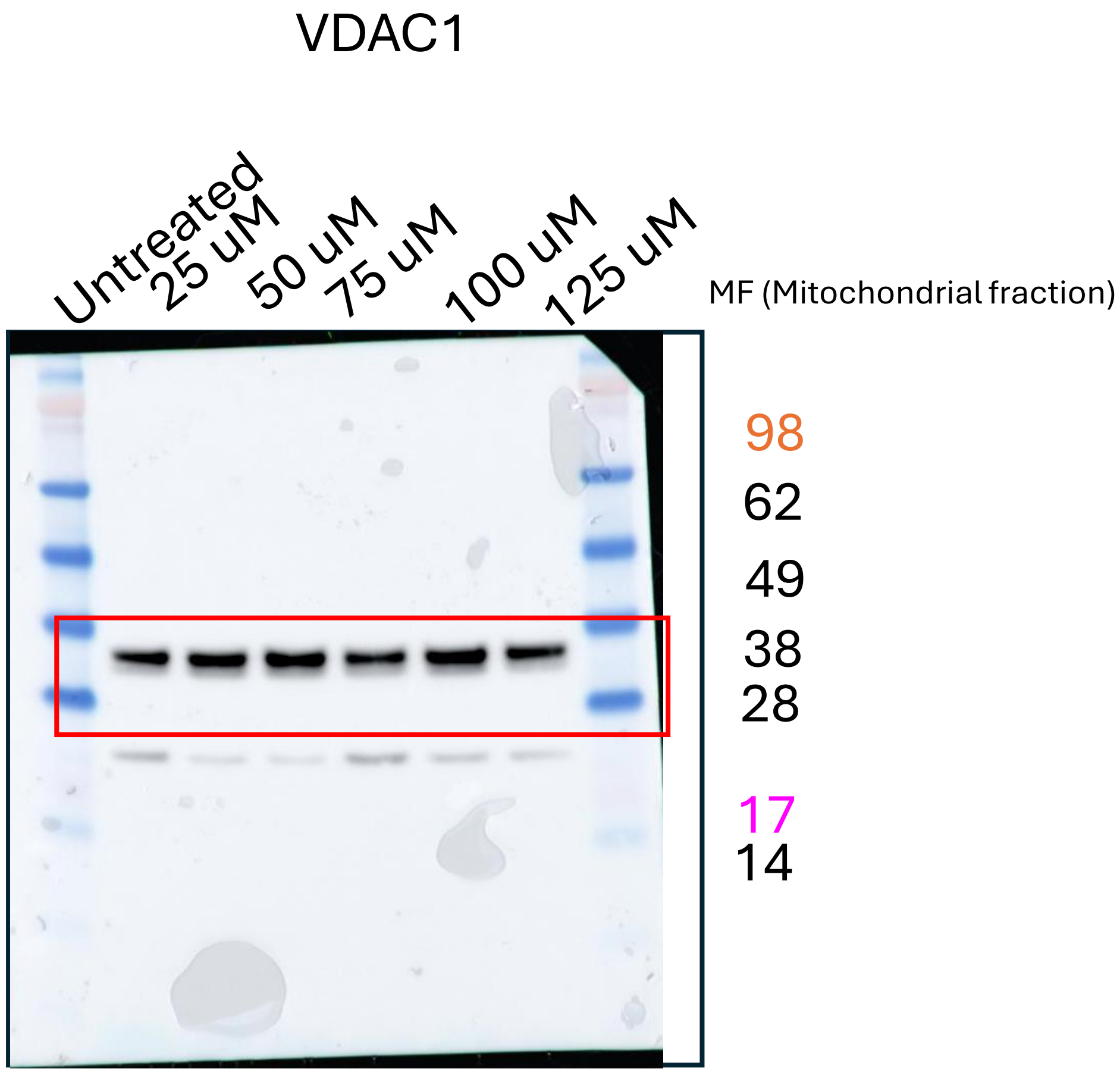

Bis-Tris 4-12% Gel
MES Buffer

Figure 2:
2F (Replicate 7)

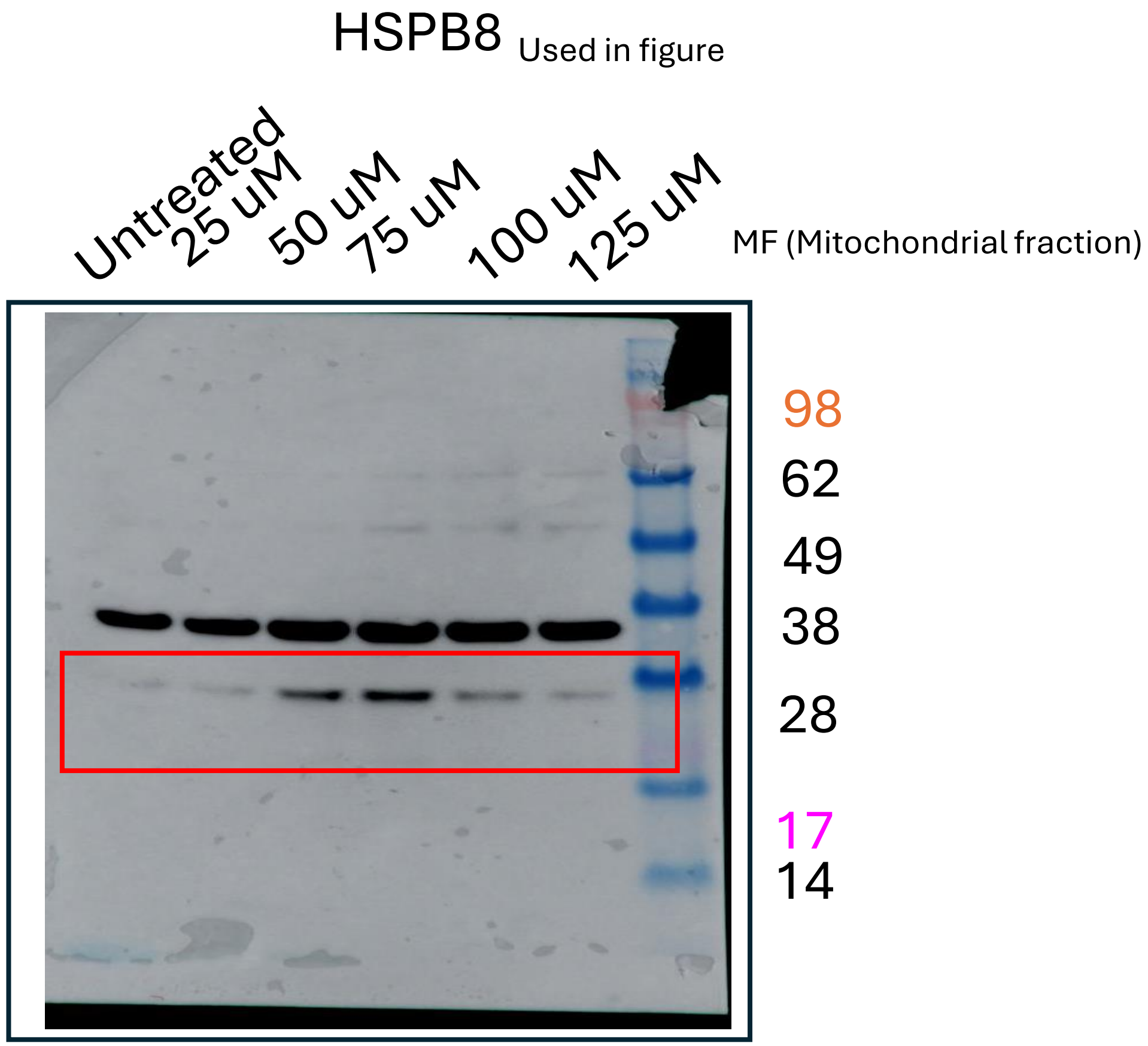

Bis-Tris 4-12% Gel
MES Buffer

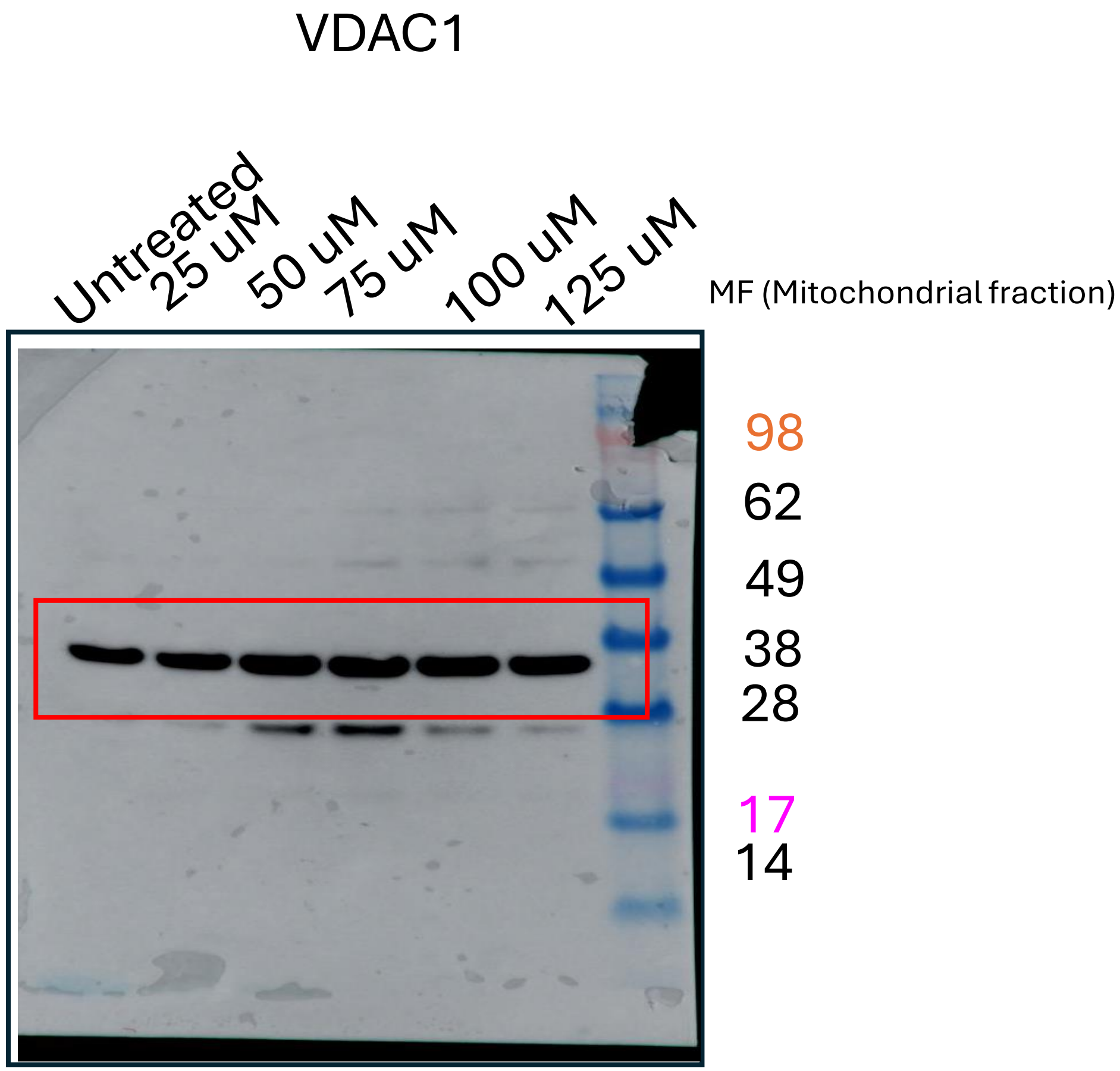

Bis-Tris 4-12% Gel
MES Buffer

Figure 2:
2F (Replicate 8)

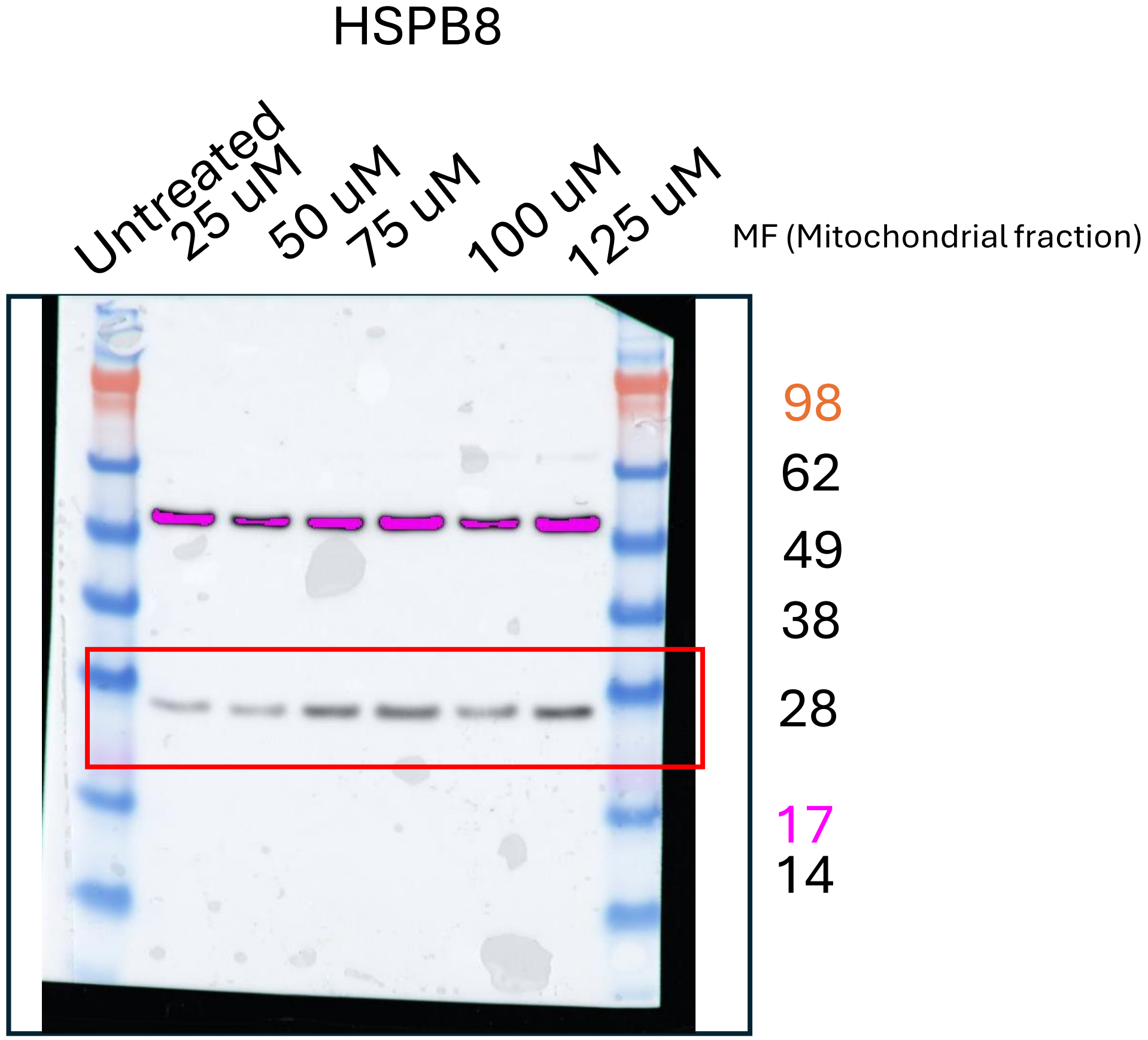

Bis-Tris 4-12% Gel
MES Buffer

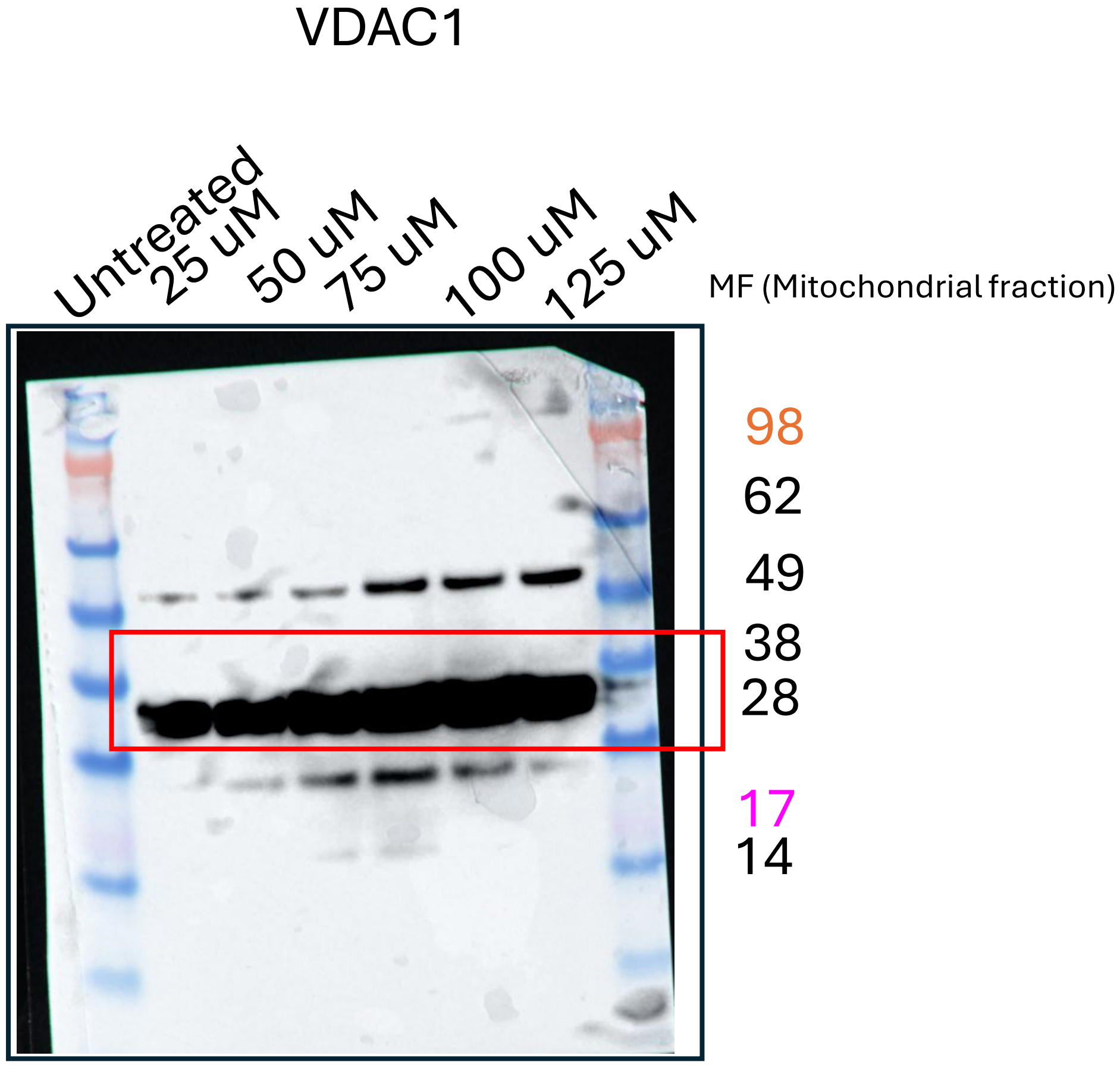

Bis-Tris 4-12% Gel
MES Buffer

Figure 2:

2F (Replicate 8)

HSPB8

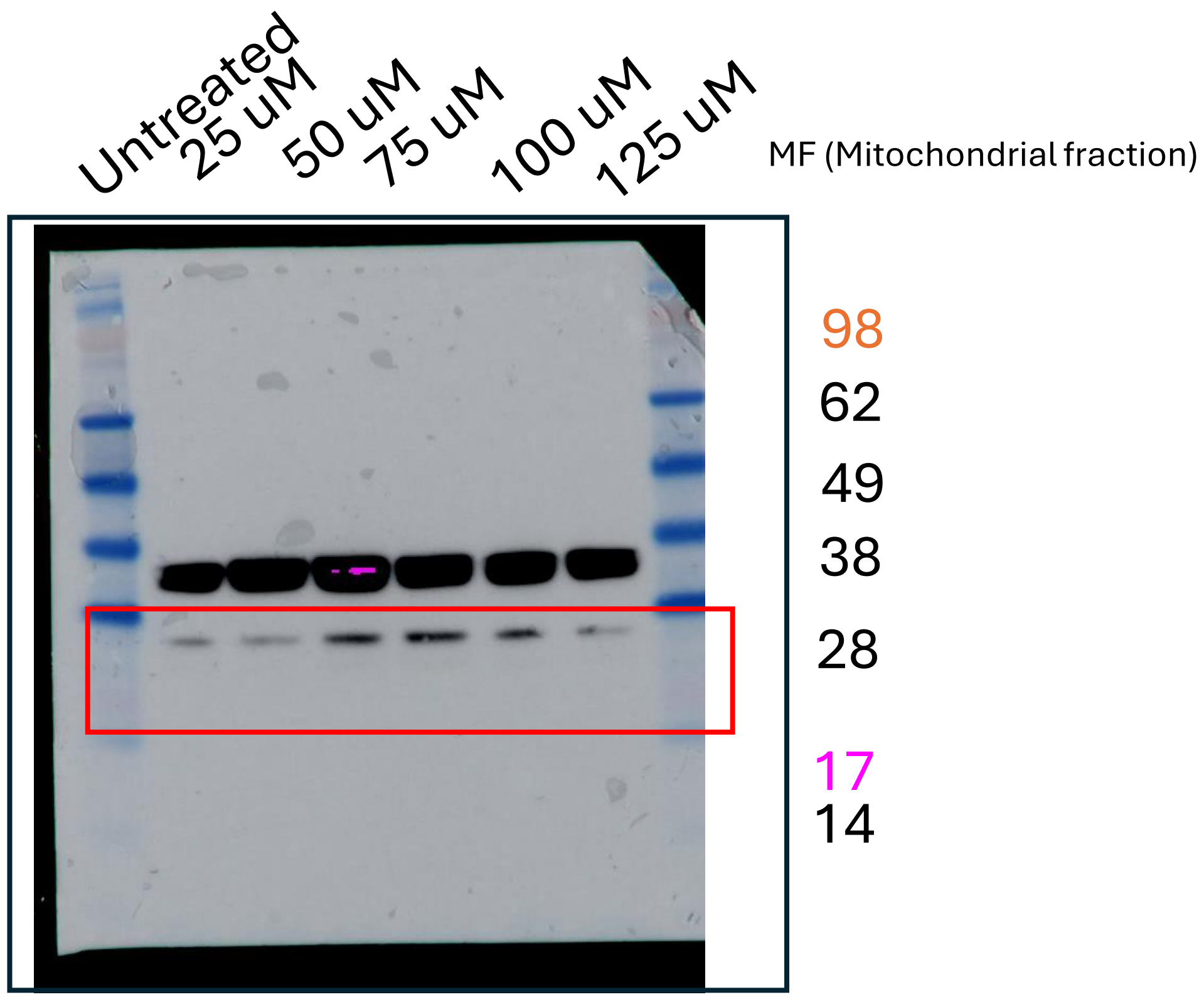

Bis-Tris 4-12% Gel

MES Buffer

VDAC1

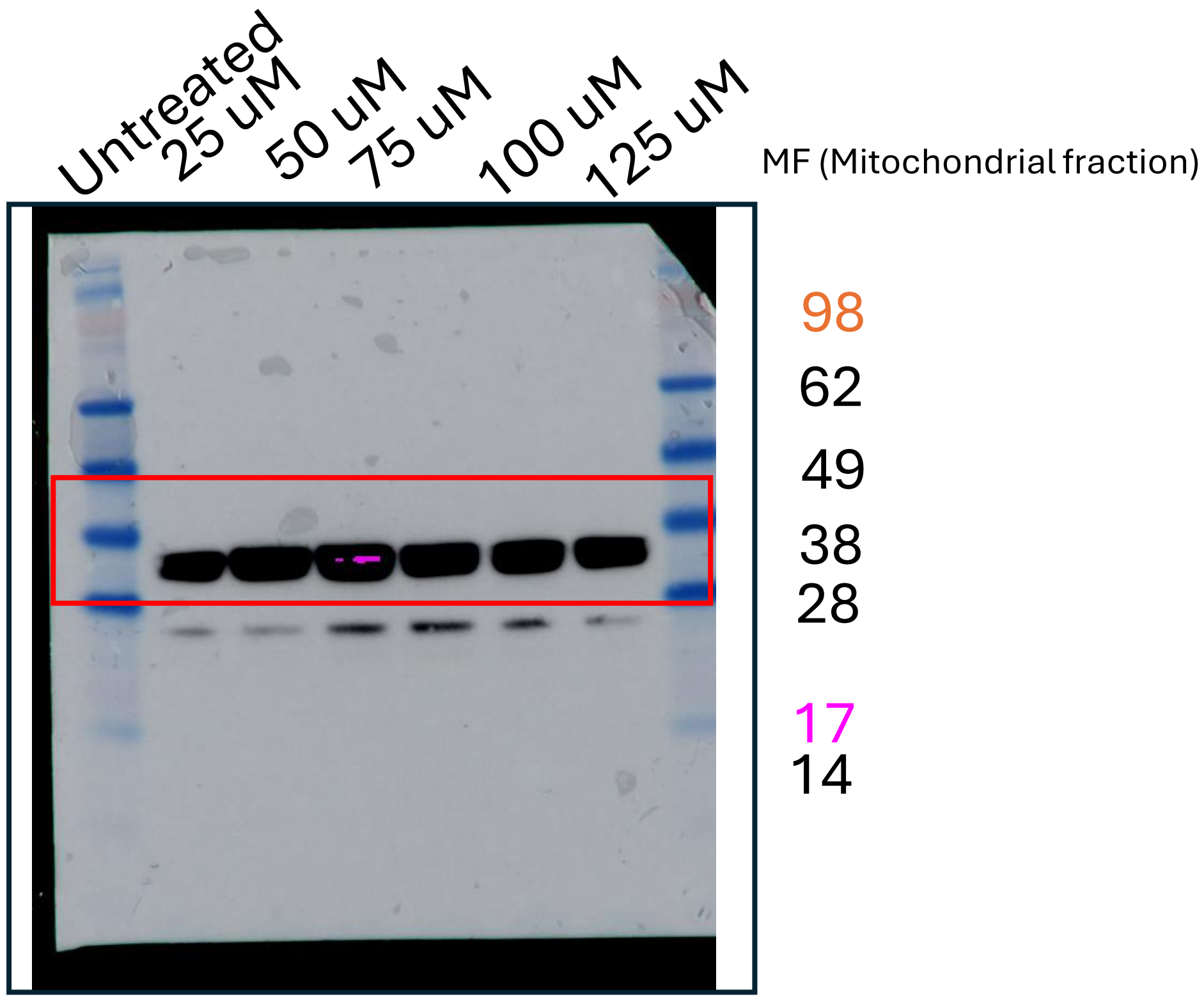

Bis-Tris 4-12% Gel

MES Buffer

Figure 2:
2J (Replicate 1)

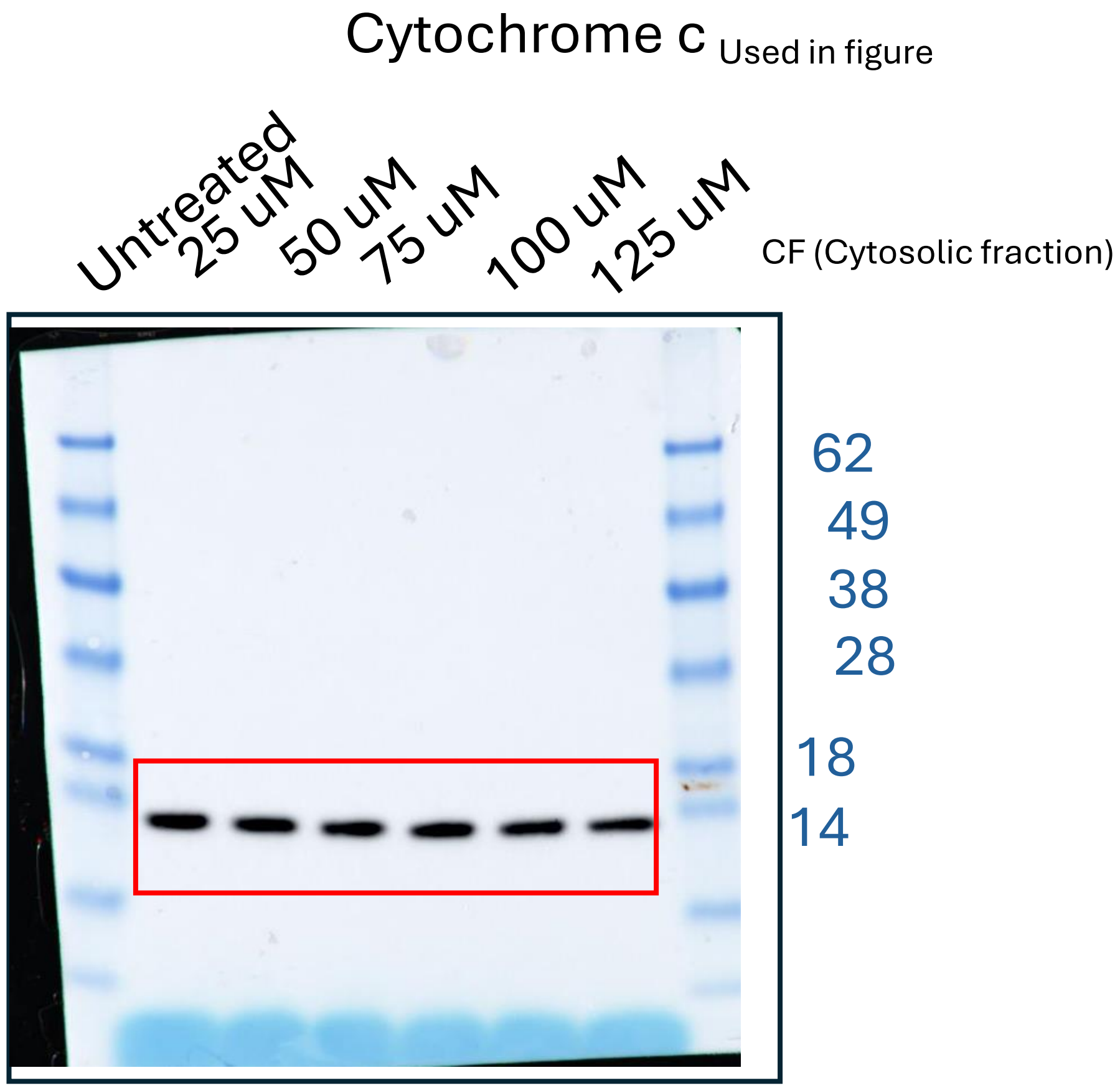

Bis-Tris 4-12% Gel
MES Buffer

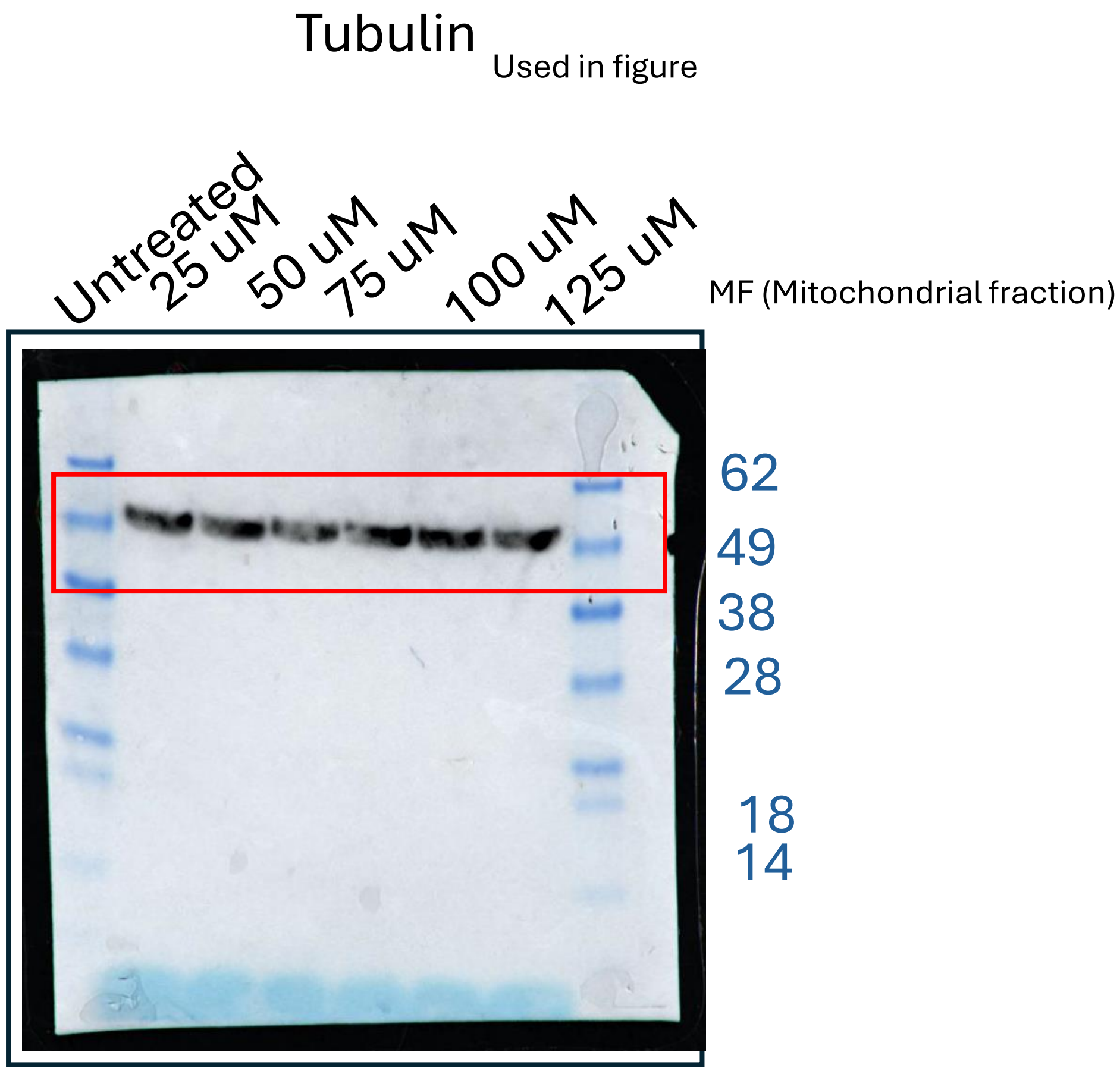

Bis-Tris 4-12% Gel
MES Buffer

Figure 2:
2J (Replicate 2)

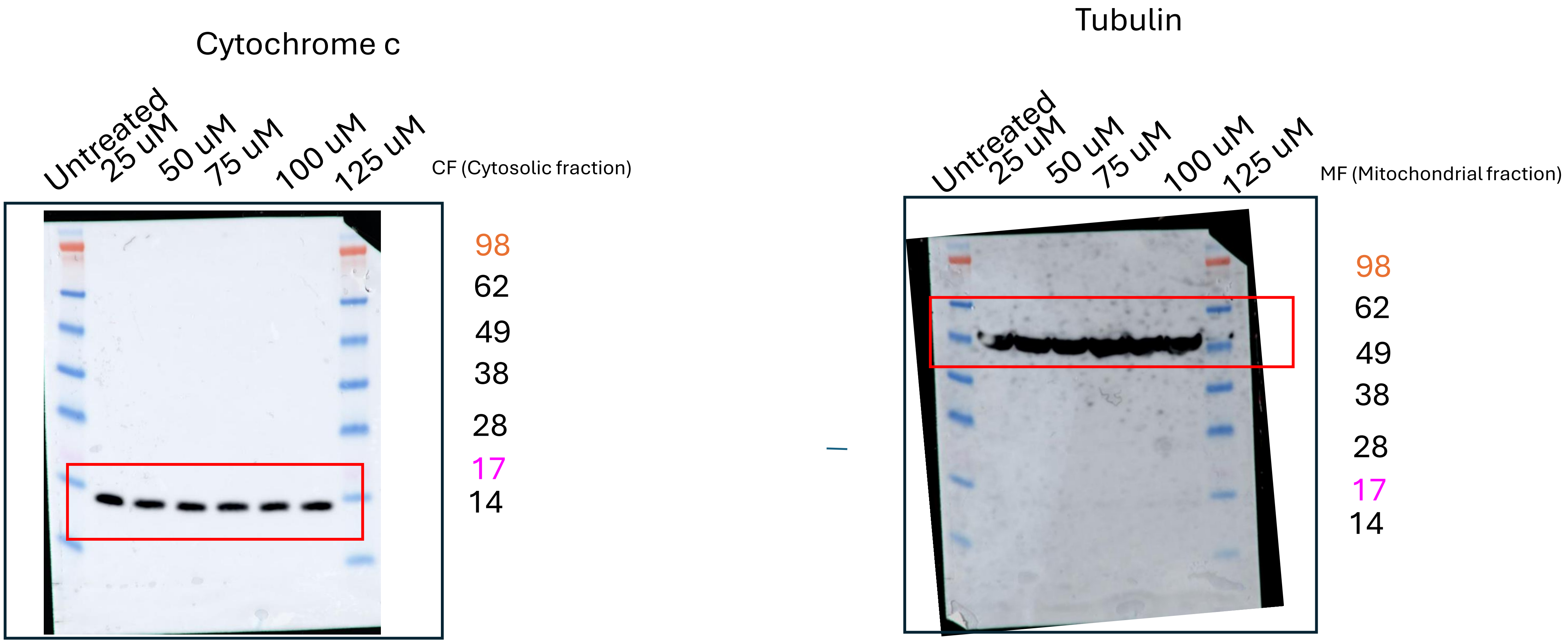

Bis-Tris 4-12% Gel
MES Buffer

Bis-Tris 4-12% Gel
MES Buffer

Figure 2:
2J (Replicate 3)

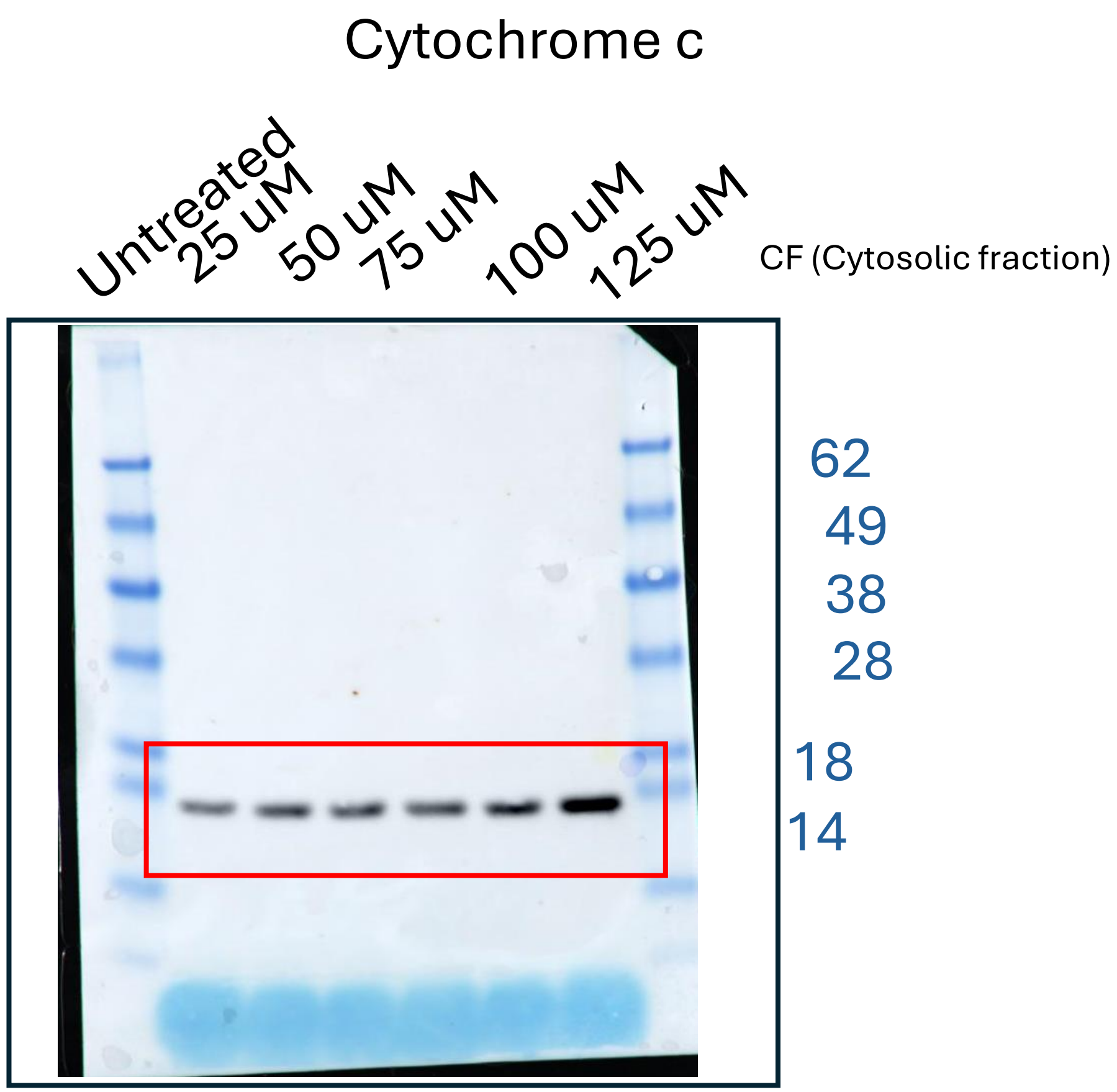

Bis-Tris 4-12% Gel
MES Buffer

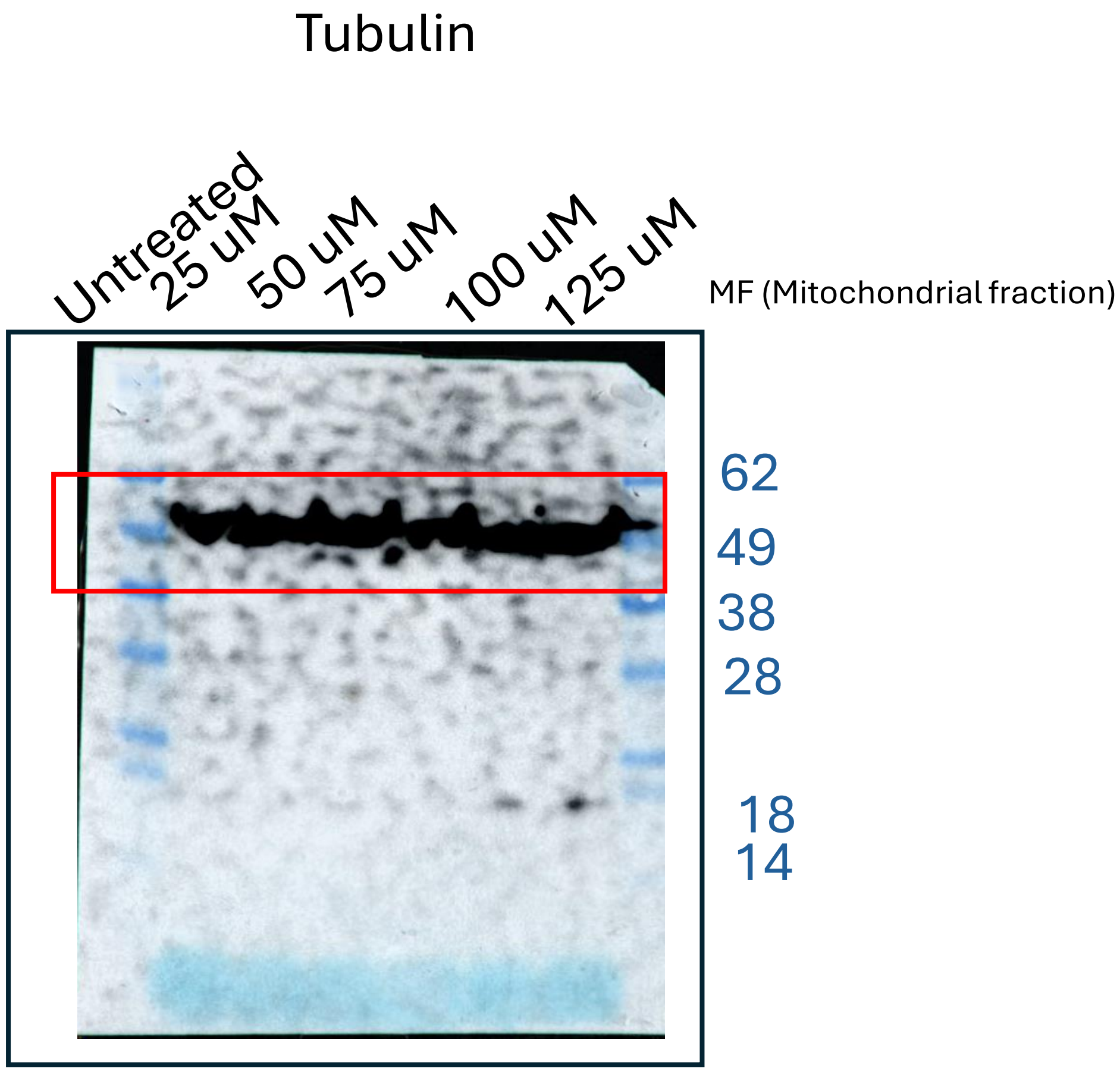

Bis-Tris 4-12% Gel
MES Buffer

Figure 3:
3C (Replicate 1)

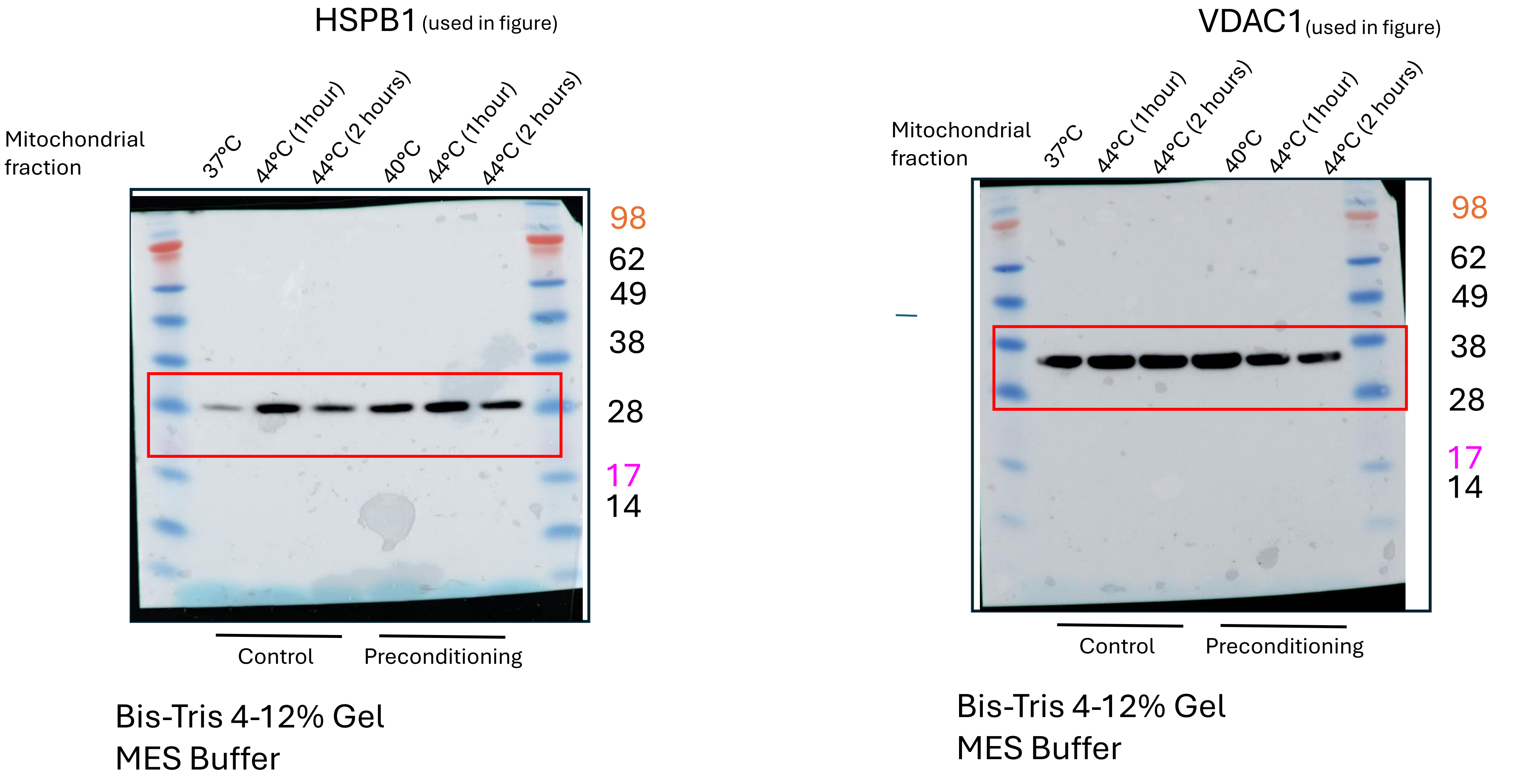

Figure 3:
3C (Replicate 2)

HSPB1 (used in figure)

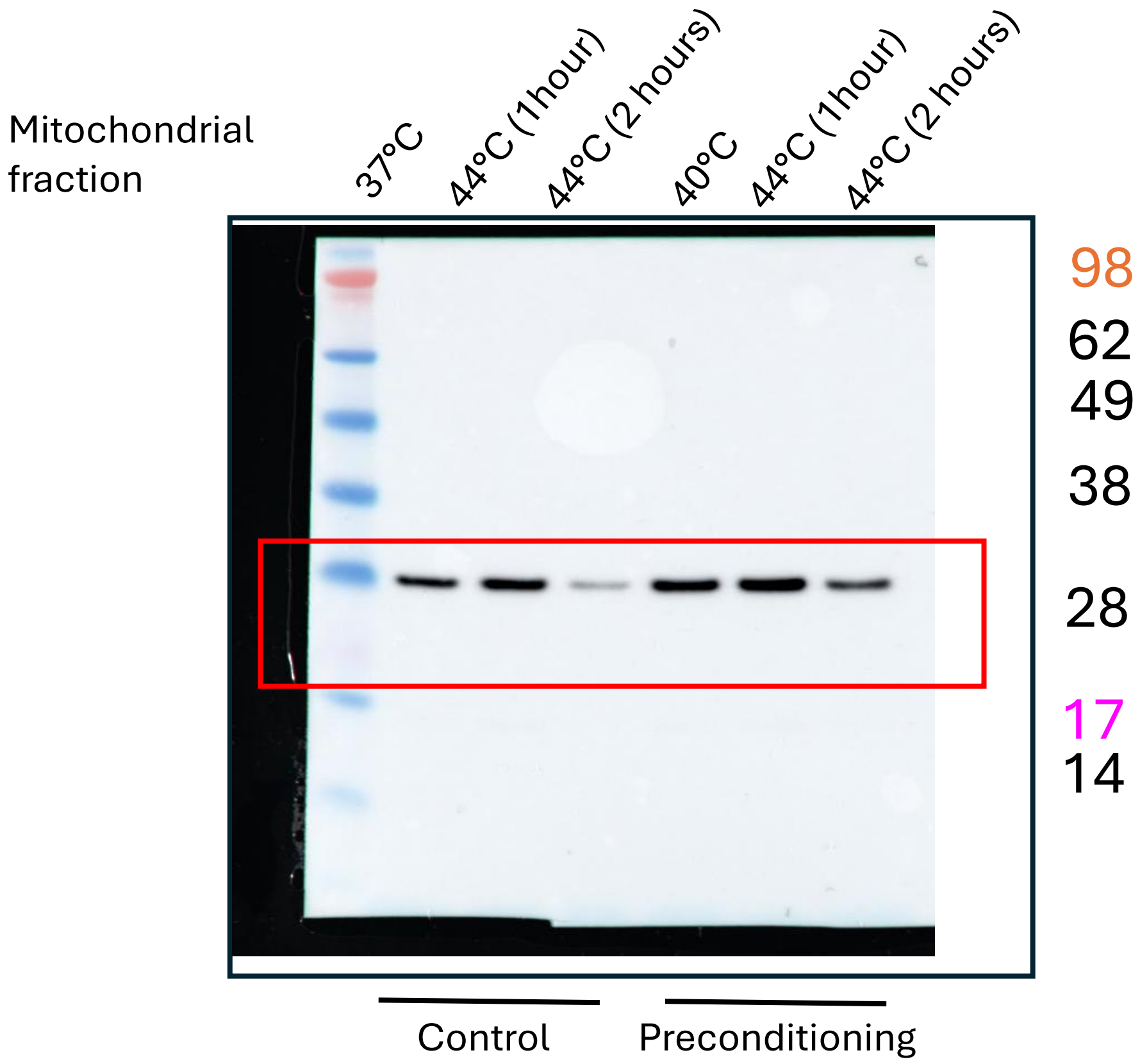

Bis-Tris 4-12% Gel
MES Buffer

VDAC1

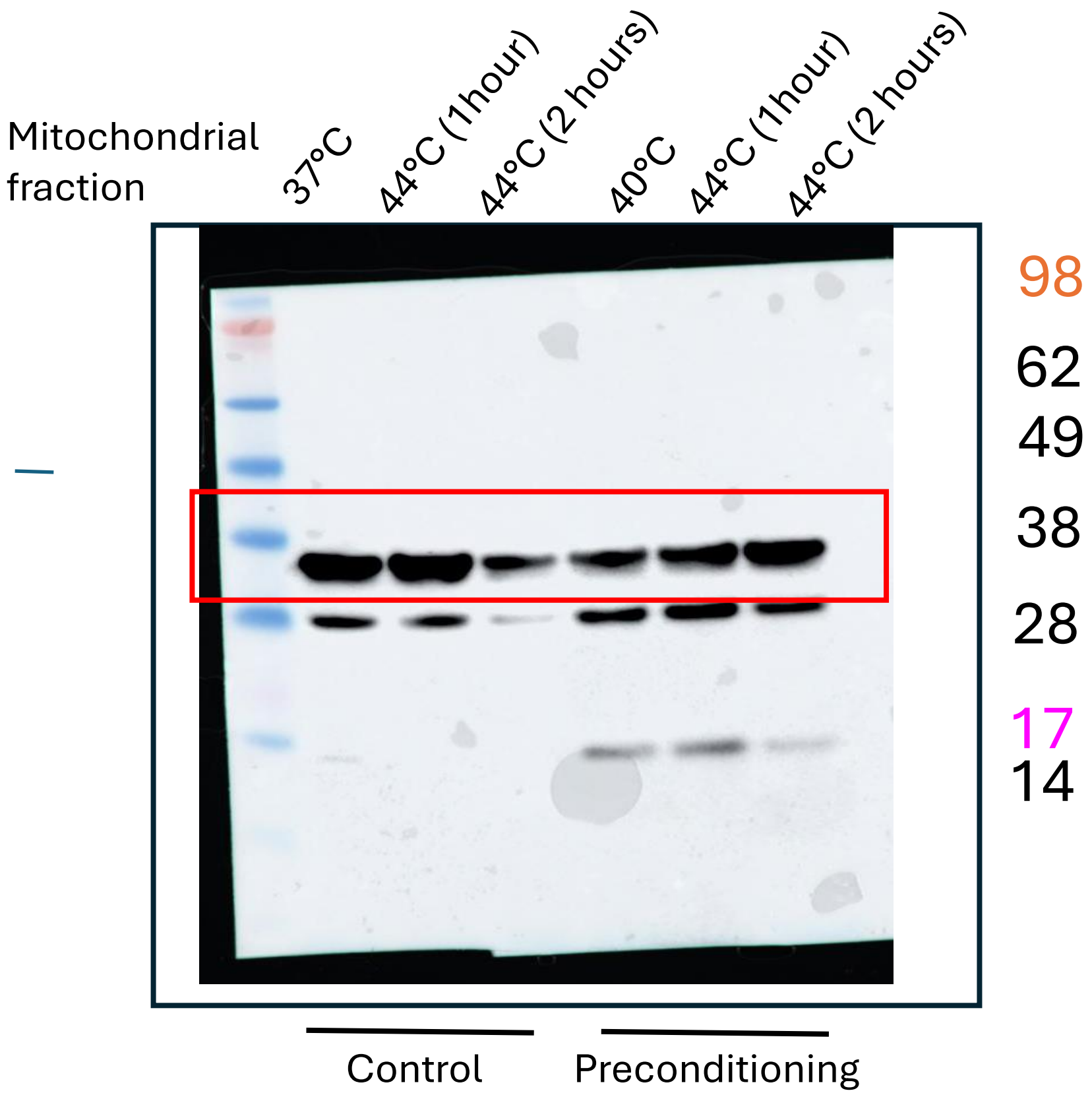

Bis-Tris 4-12% Gel
MES Buffer

Figure 3:
3C (Replicate 3)

HSPB1 (used in figure)

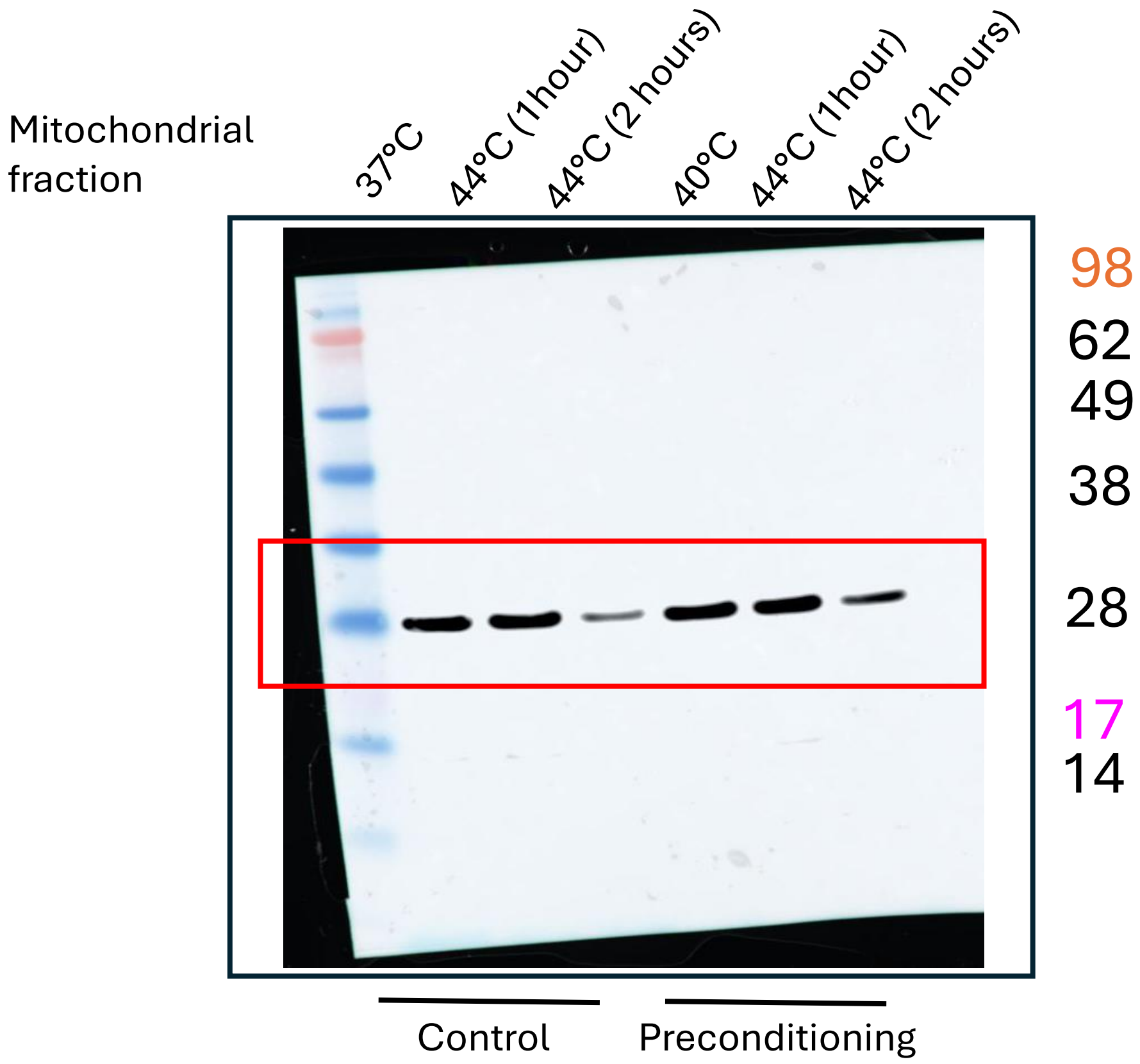

Bis-Tris 4-12% Gel
MES Buffer

VDAC1

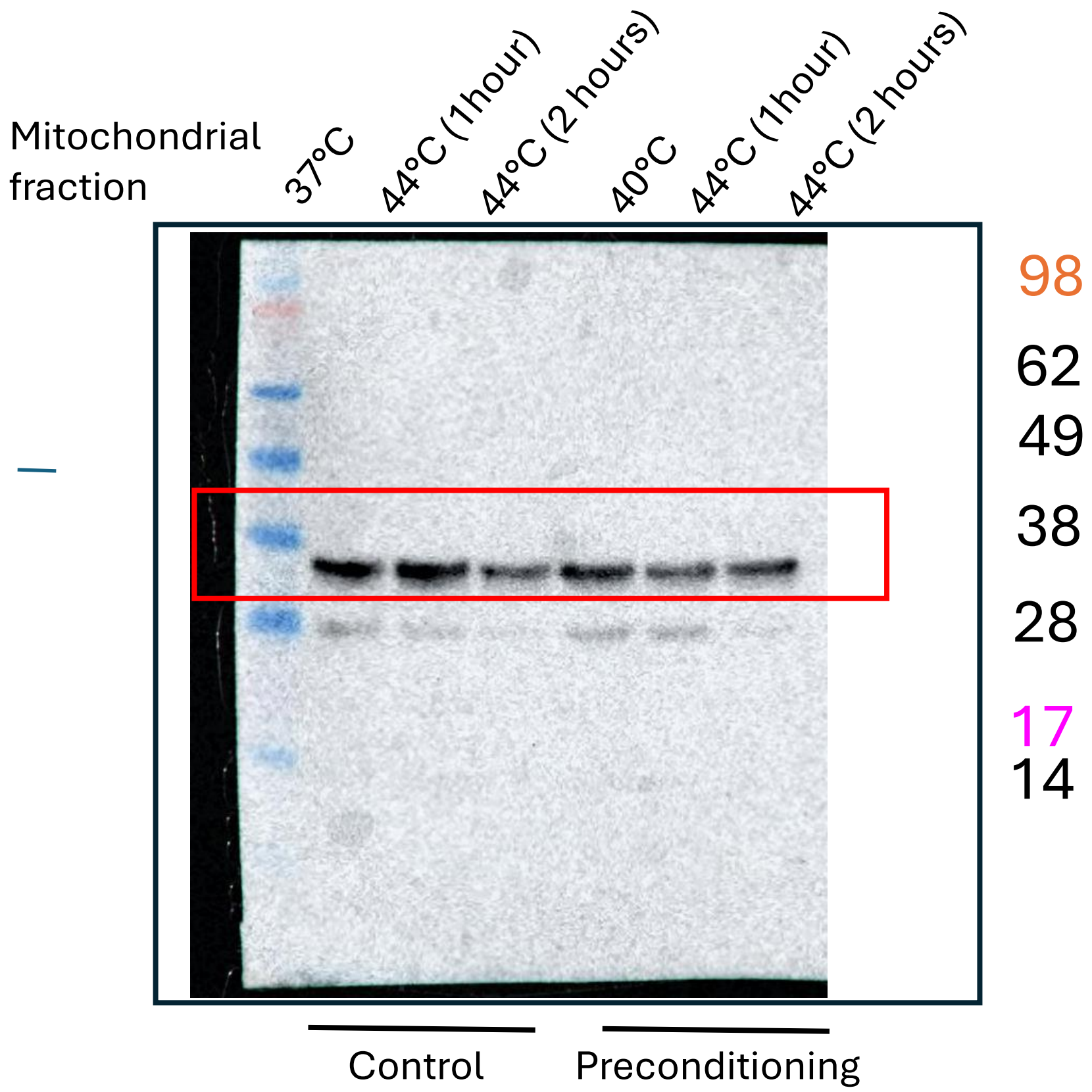

Bis-Tris 4-12% Gel
MES Buffer

Figure 3:
3C (Replicate 4)

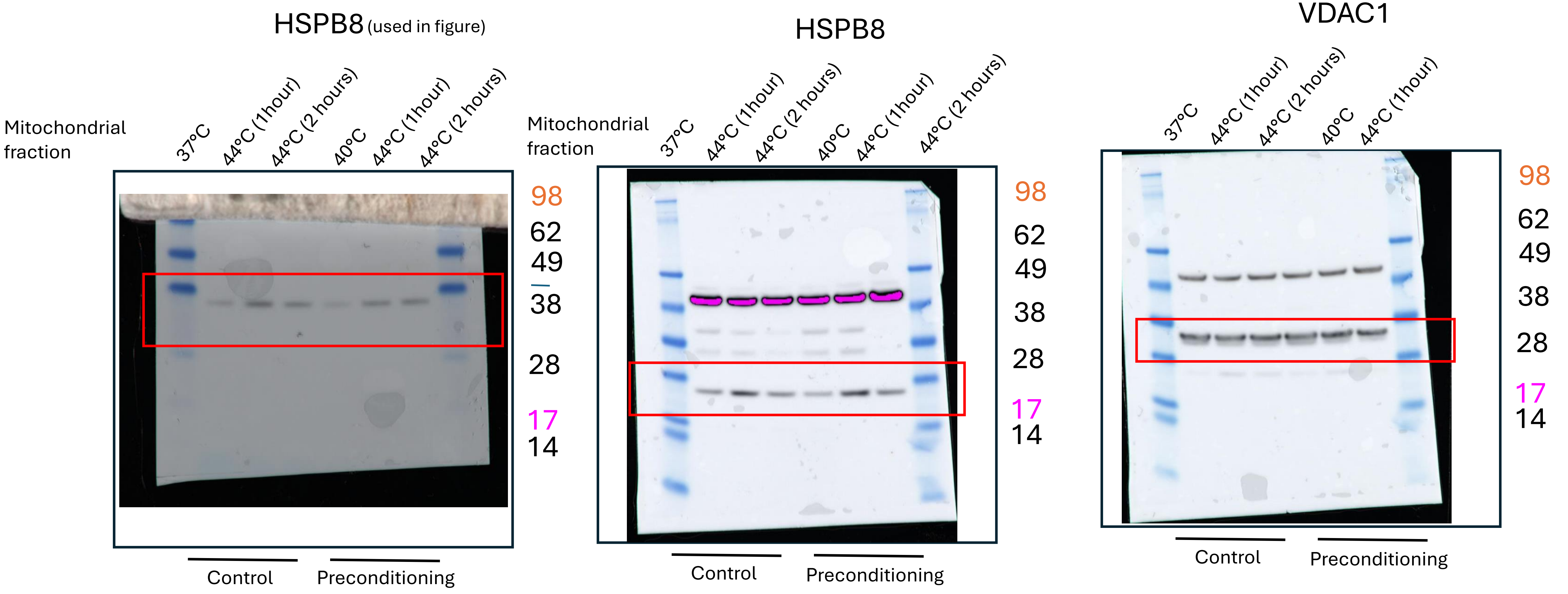

Bis-Tris 4-12% Gel
MES Buffer

Bis-Tris 4-12% Gel
MES Buffer

Same samples were run in two different blots

Figure 3:
3C (Replicate 5)

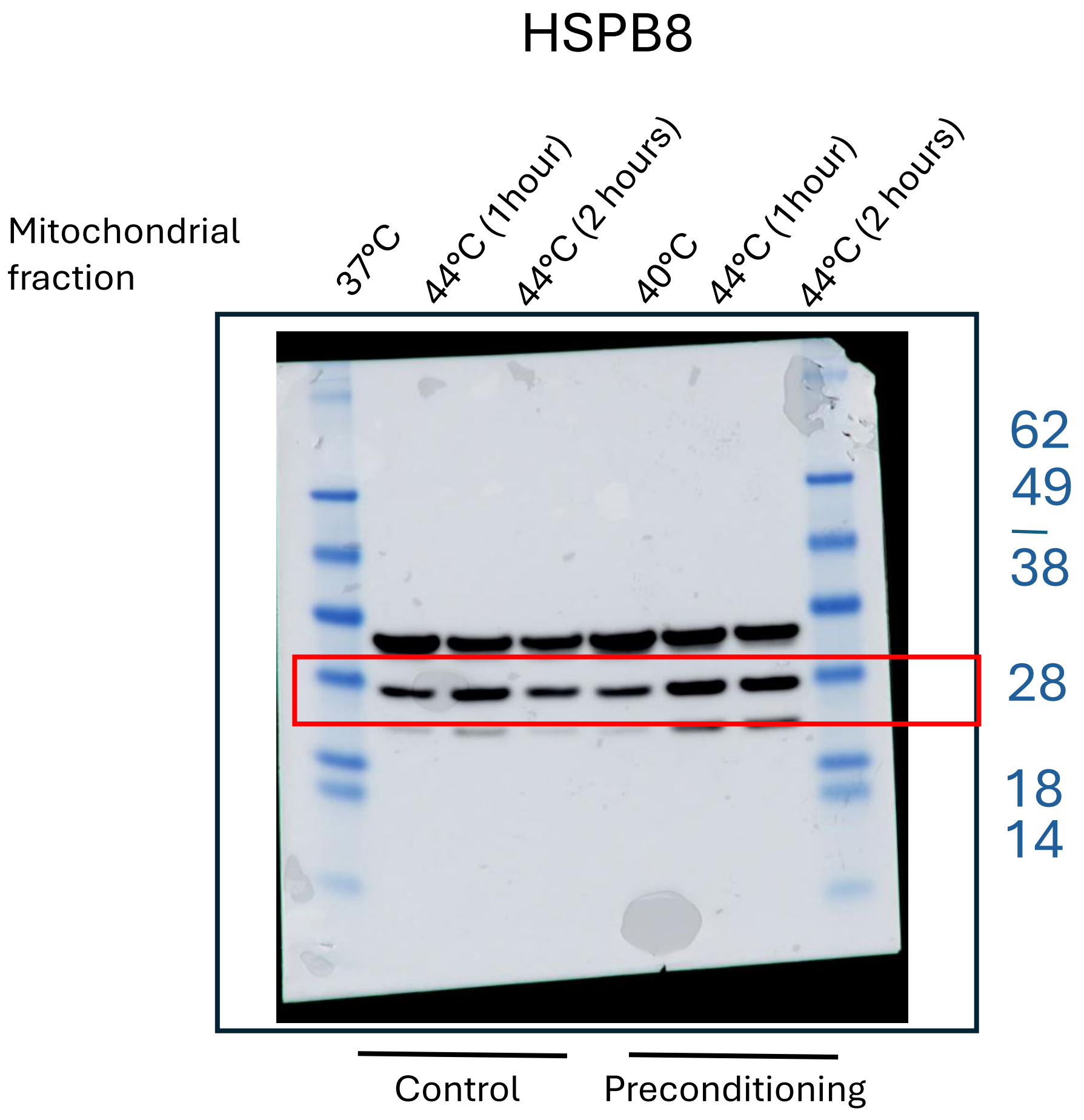

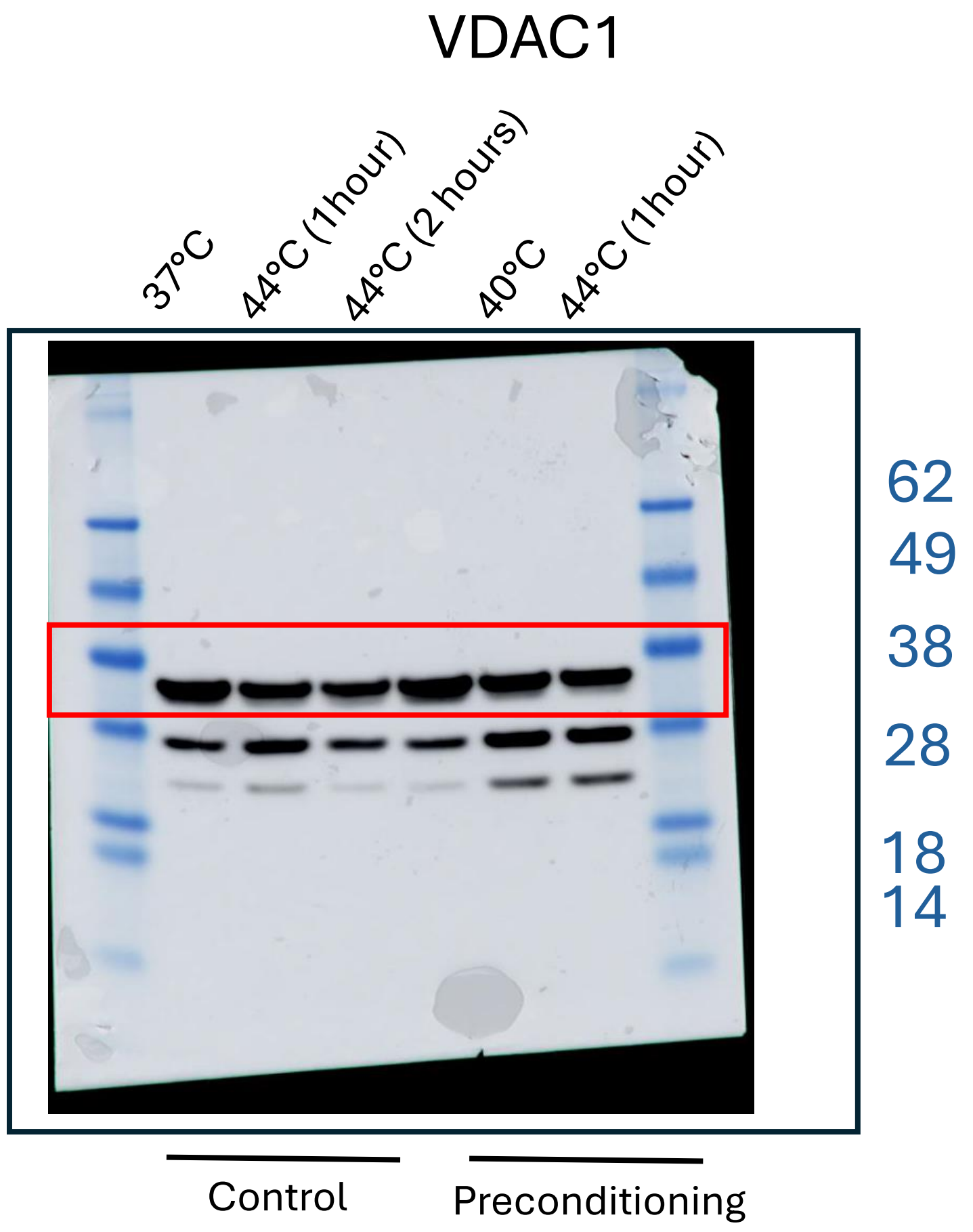

Bis-Tris 4-12% Gel
MES Buffer

Figure 3:
3C (Replicate 6)

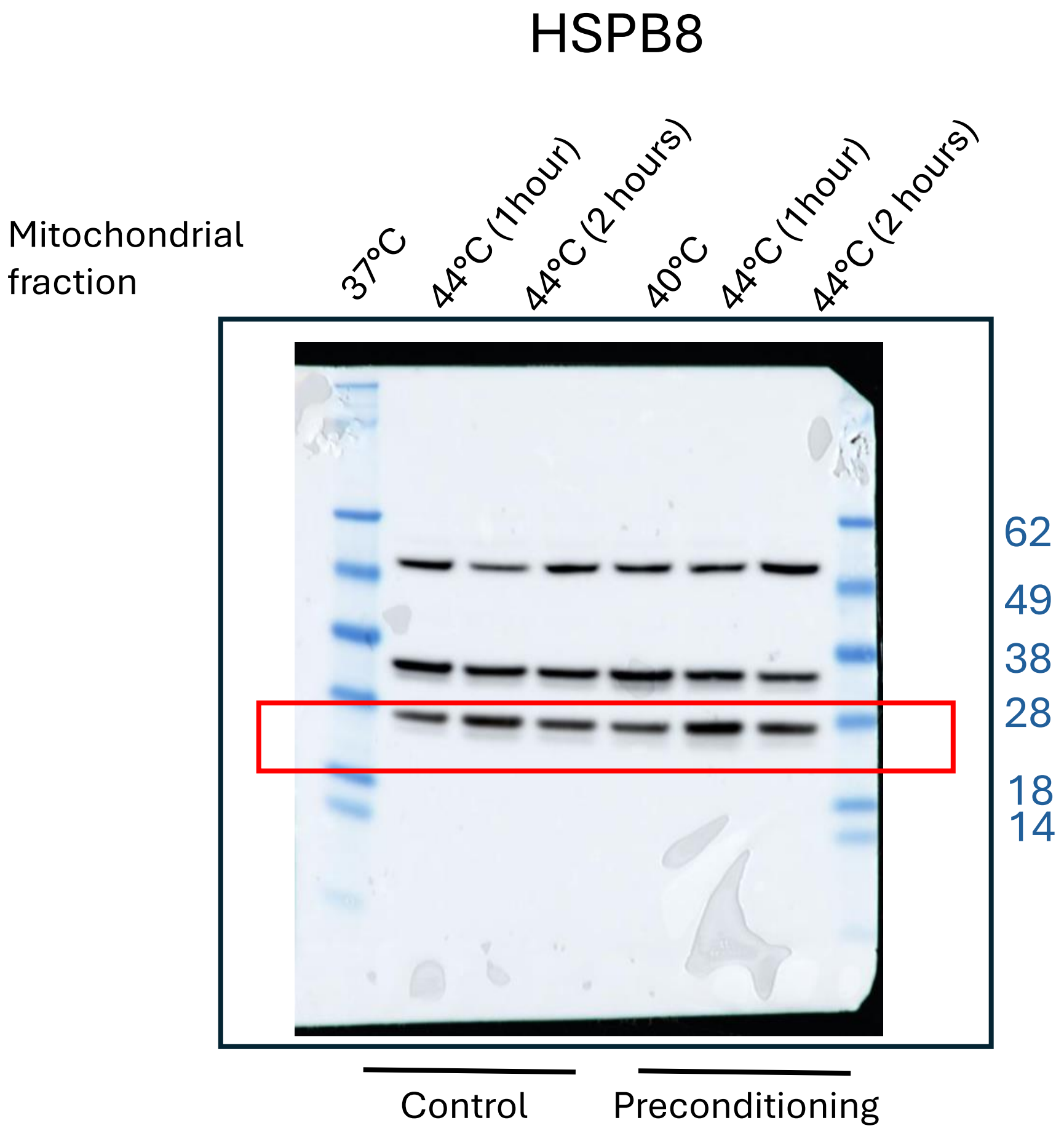

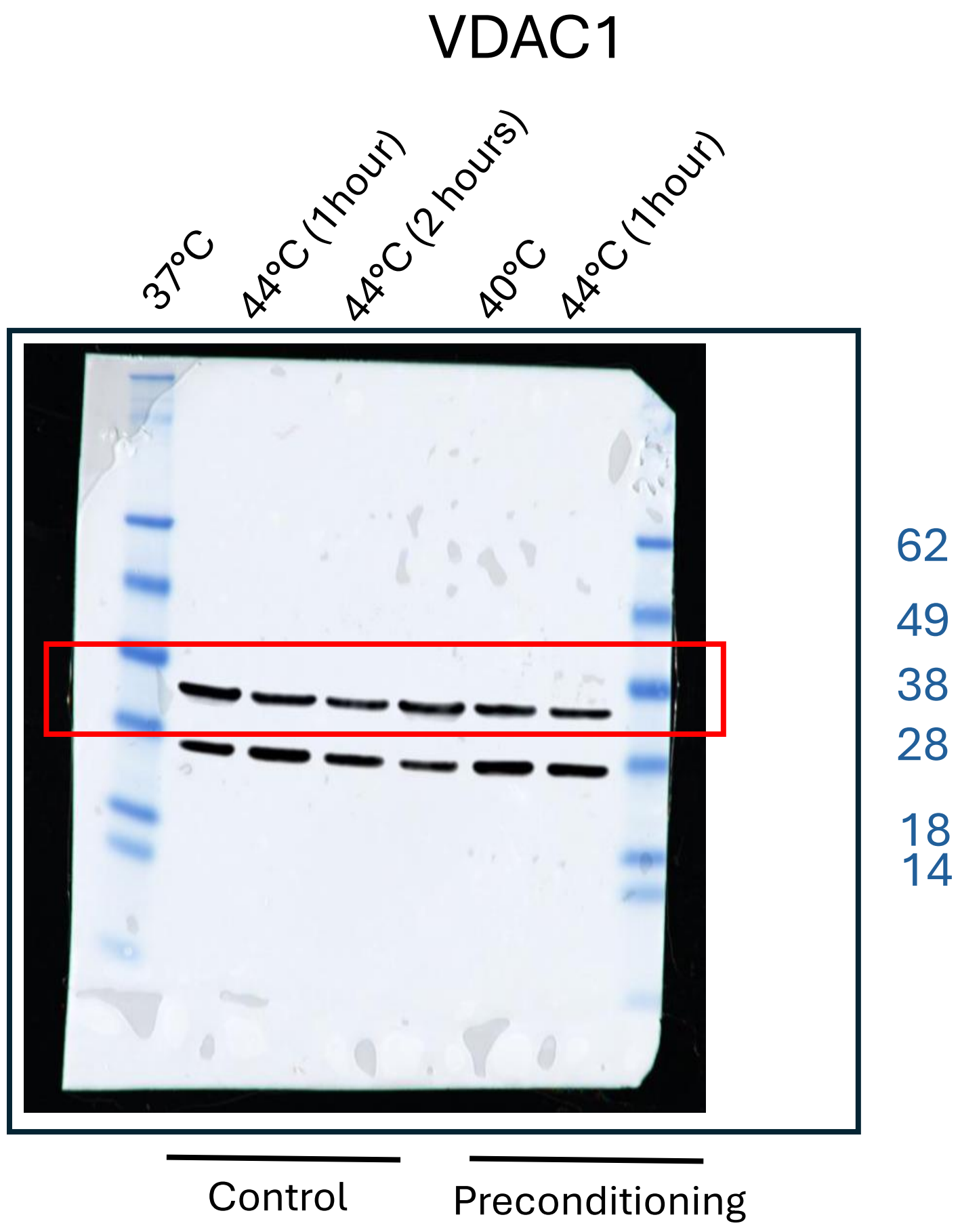

Bis-Tris 4-12% Gel
MES Buffer

Figure 3:
3C (Replicate 7)

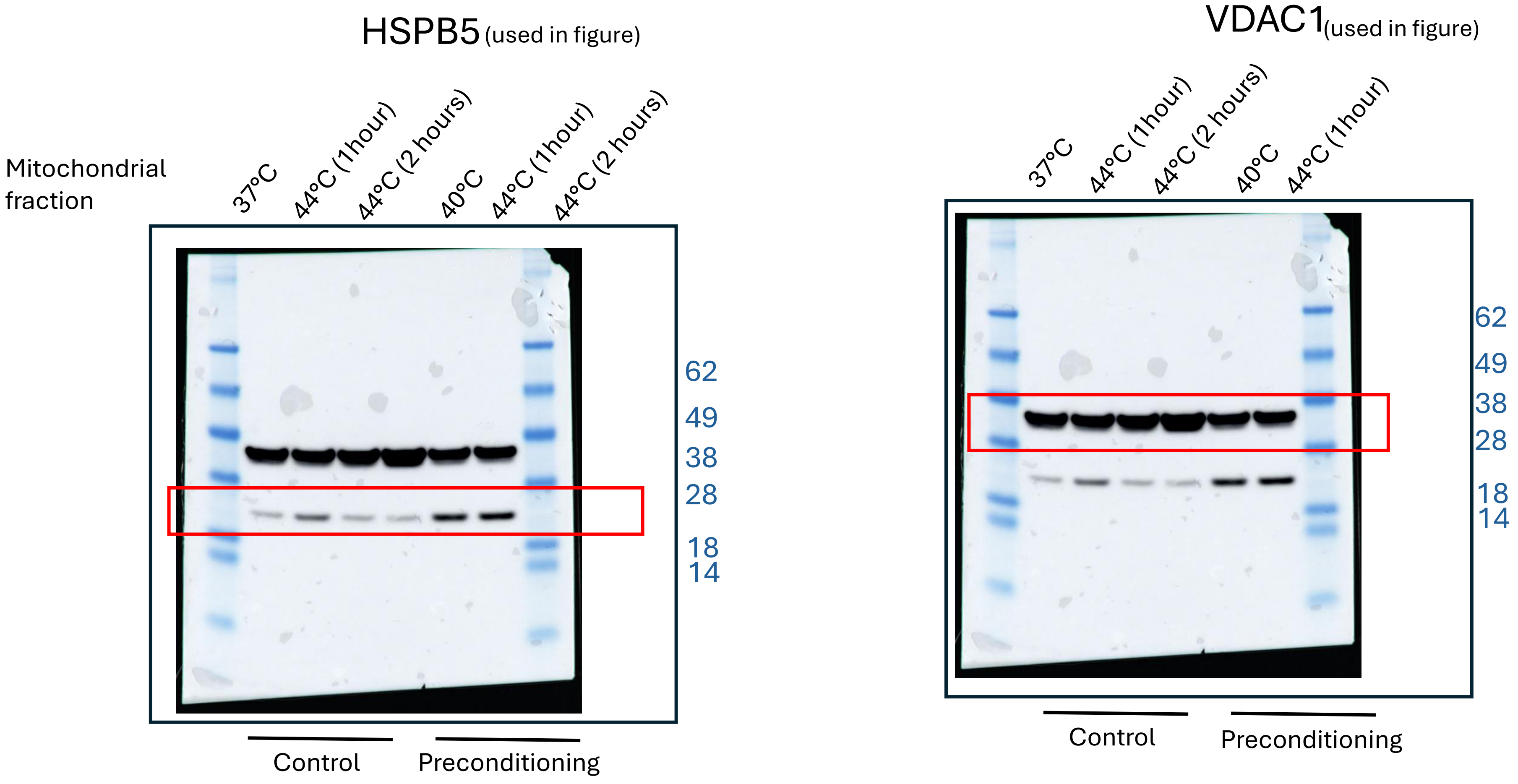

Bis-Tris 4-12% Gel
MES Buffer

Figure 3:
3C (Replicate 8)

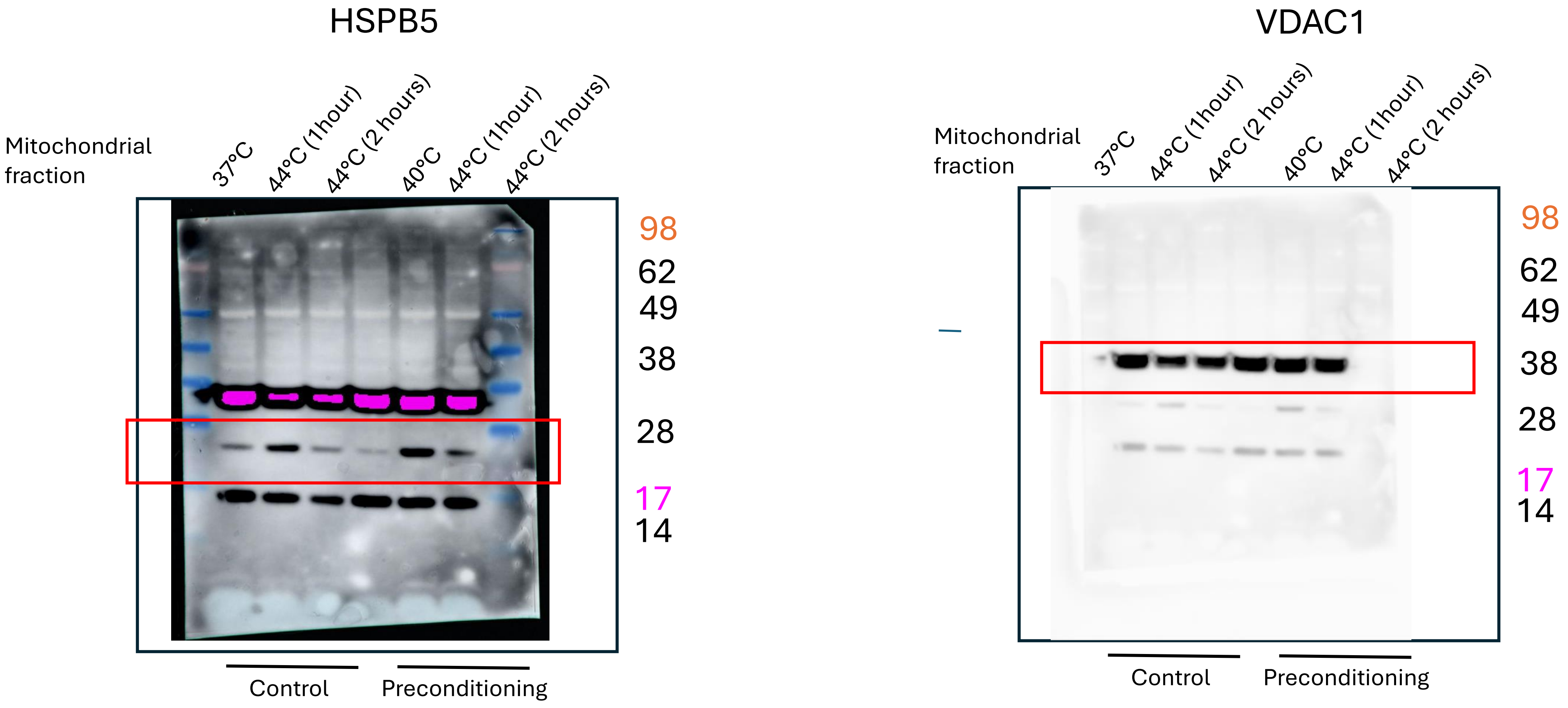

Bis-Tris 4-12% Gel
MES Buffer

Bis-Tris 4-12% Gel
MES Buffer

Figure 3:
3C (Replicate 9)

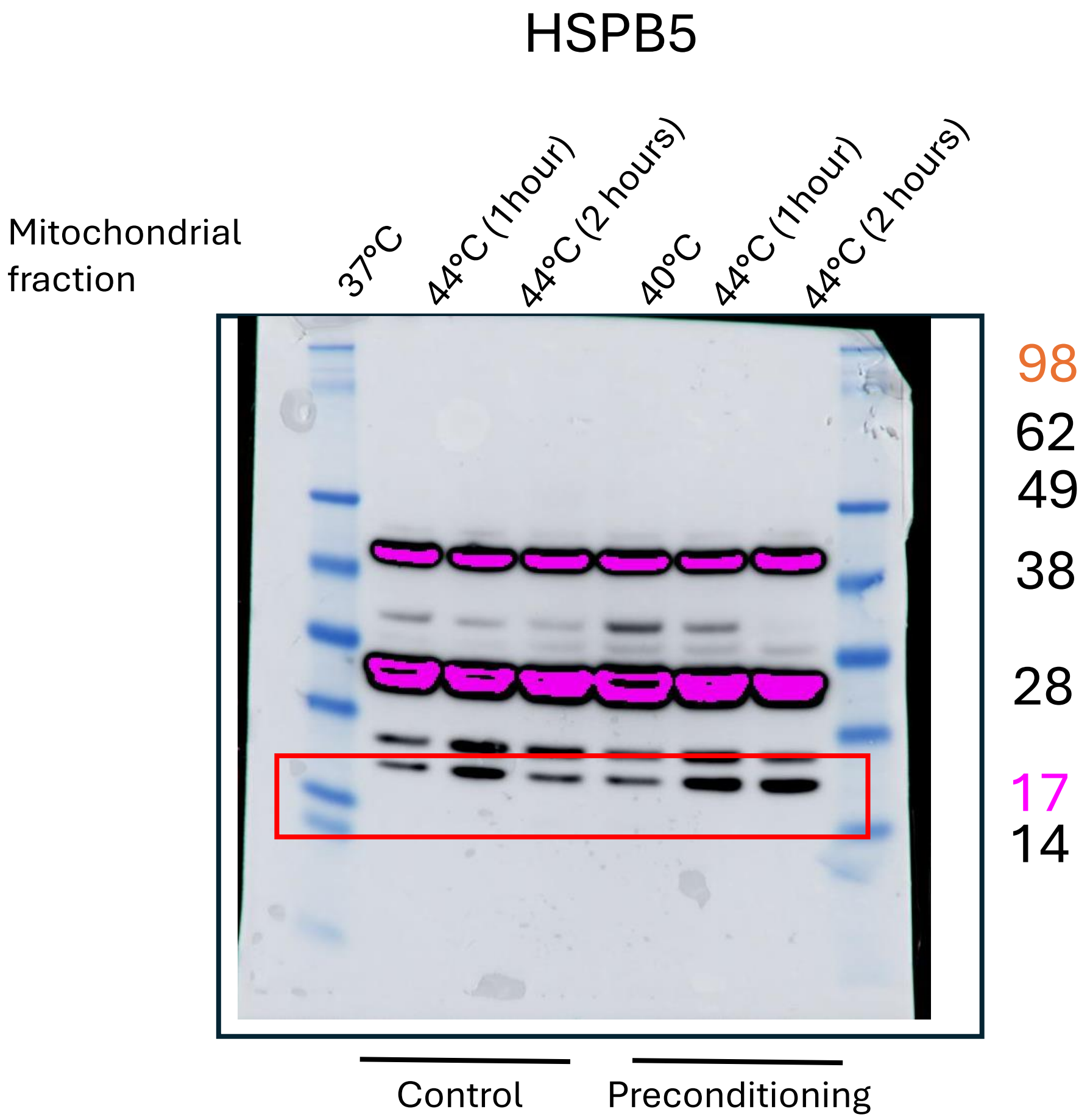

Bis-Tris 4-12% Gel
MES Buffer

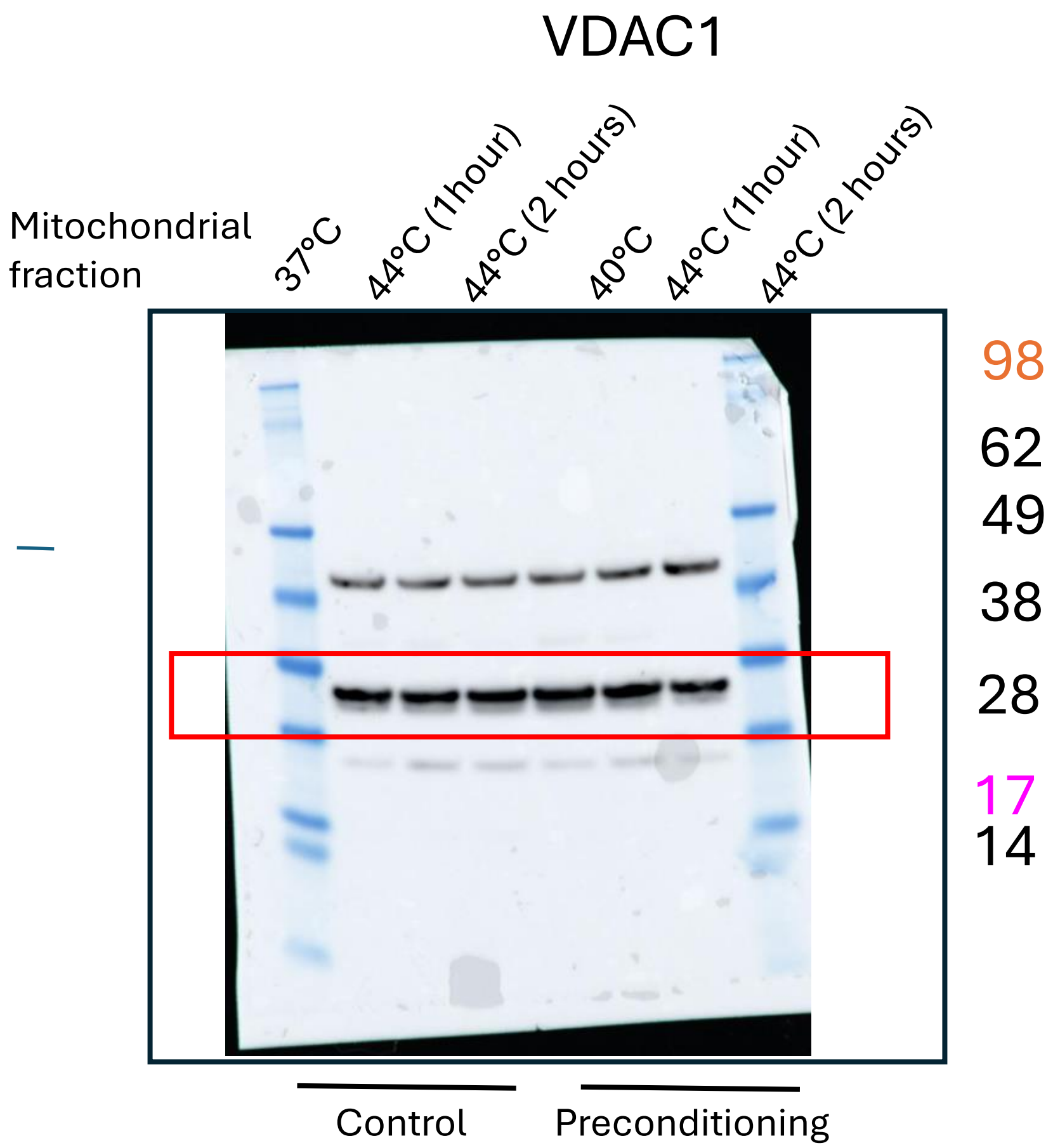

Bis-Tris 4-12% Gel
MES Buffer

Figure 3:
3E (Replicate 1)

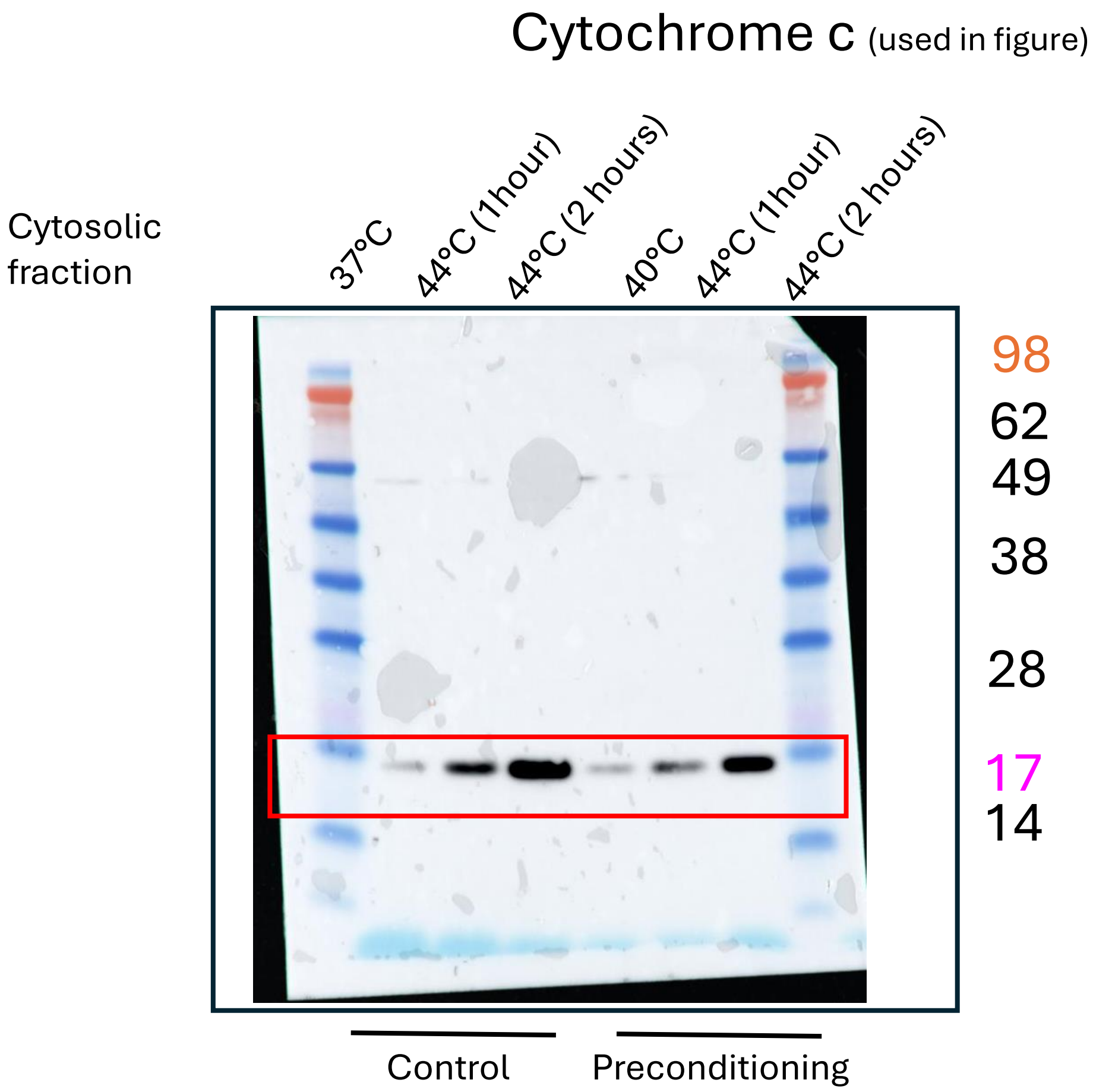

Bis-Tris 4-12% Gel
MES Buffer

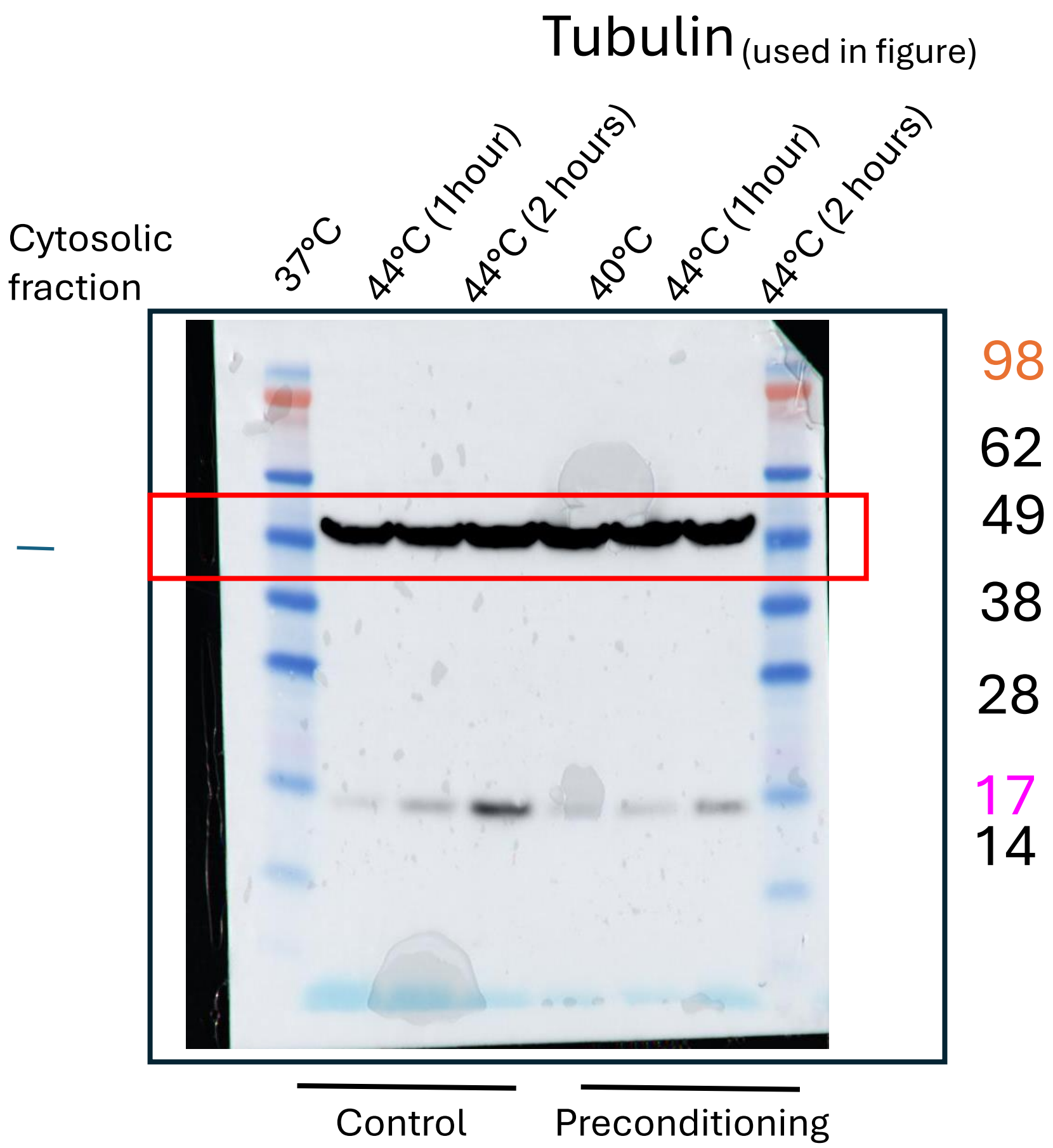

Bis-Tris 4-12% Gel
MES Buffer

Figure 3:
3E (Replicate 2)

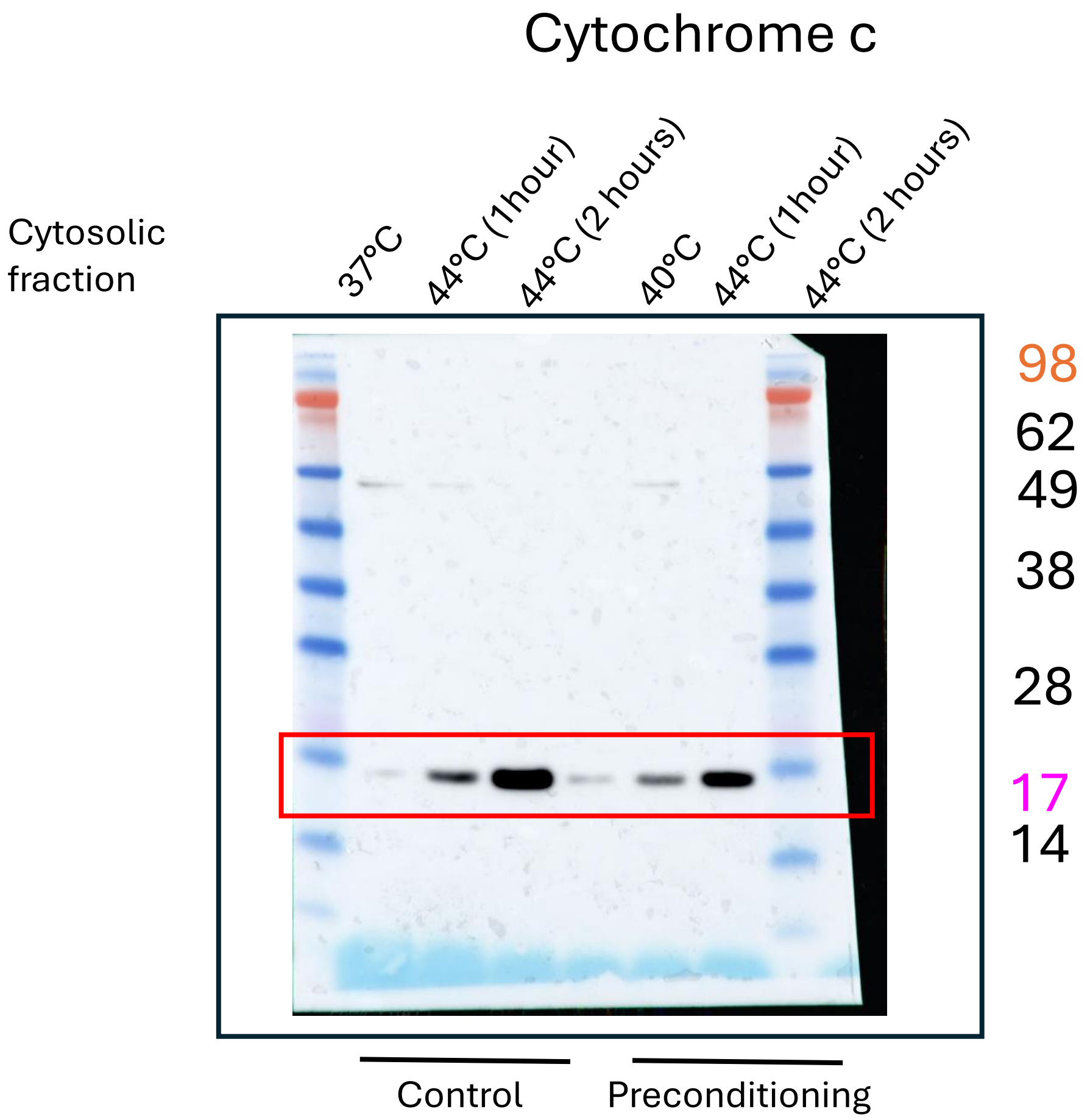

Bis-Tris 4-12% Gel
MES Buffer

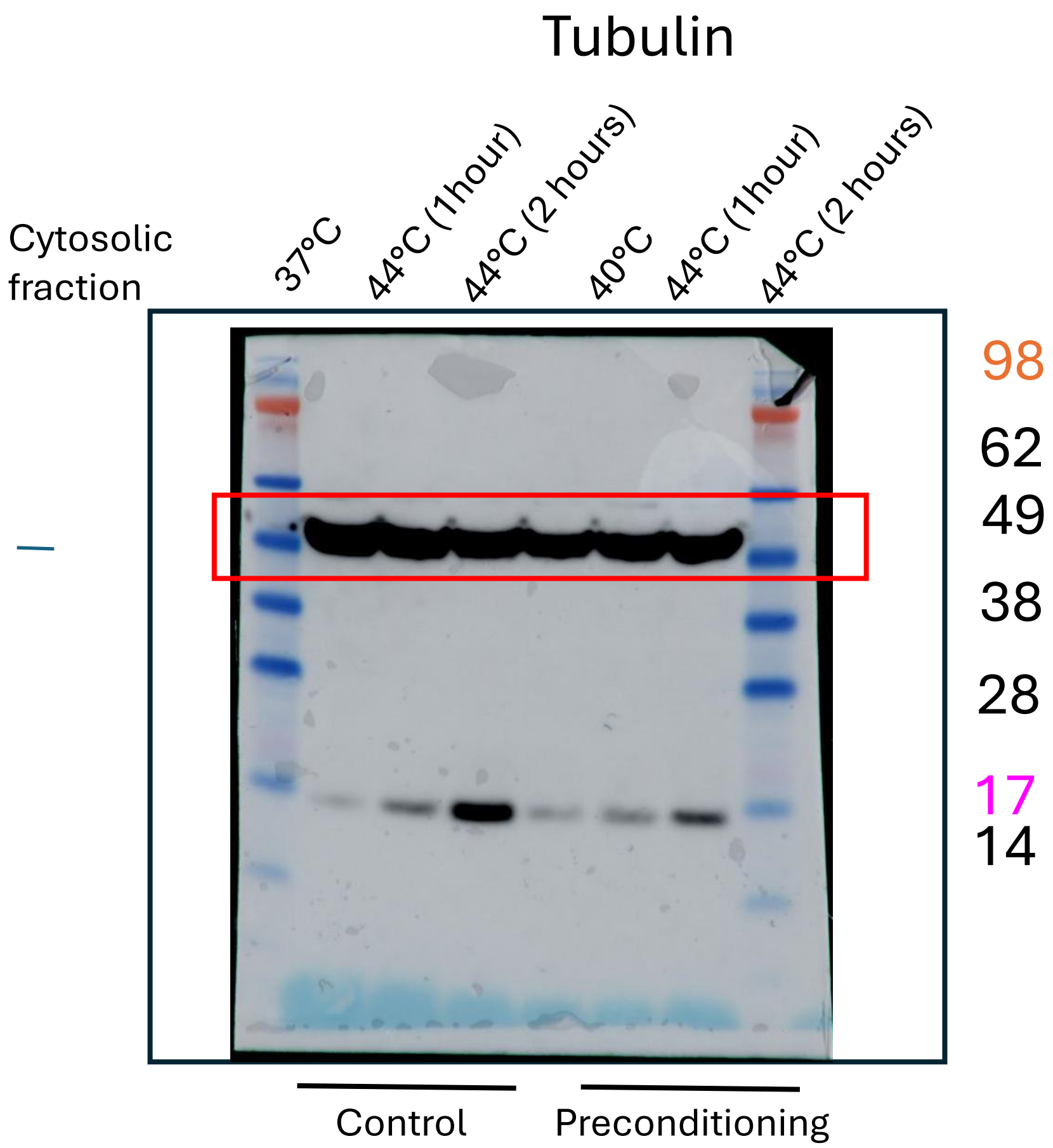

Bis-Tris 4-12% Gel
MES Buffer

Figure 3:
3E (Replicate 3)

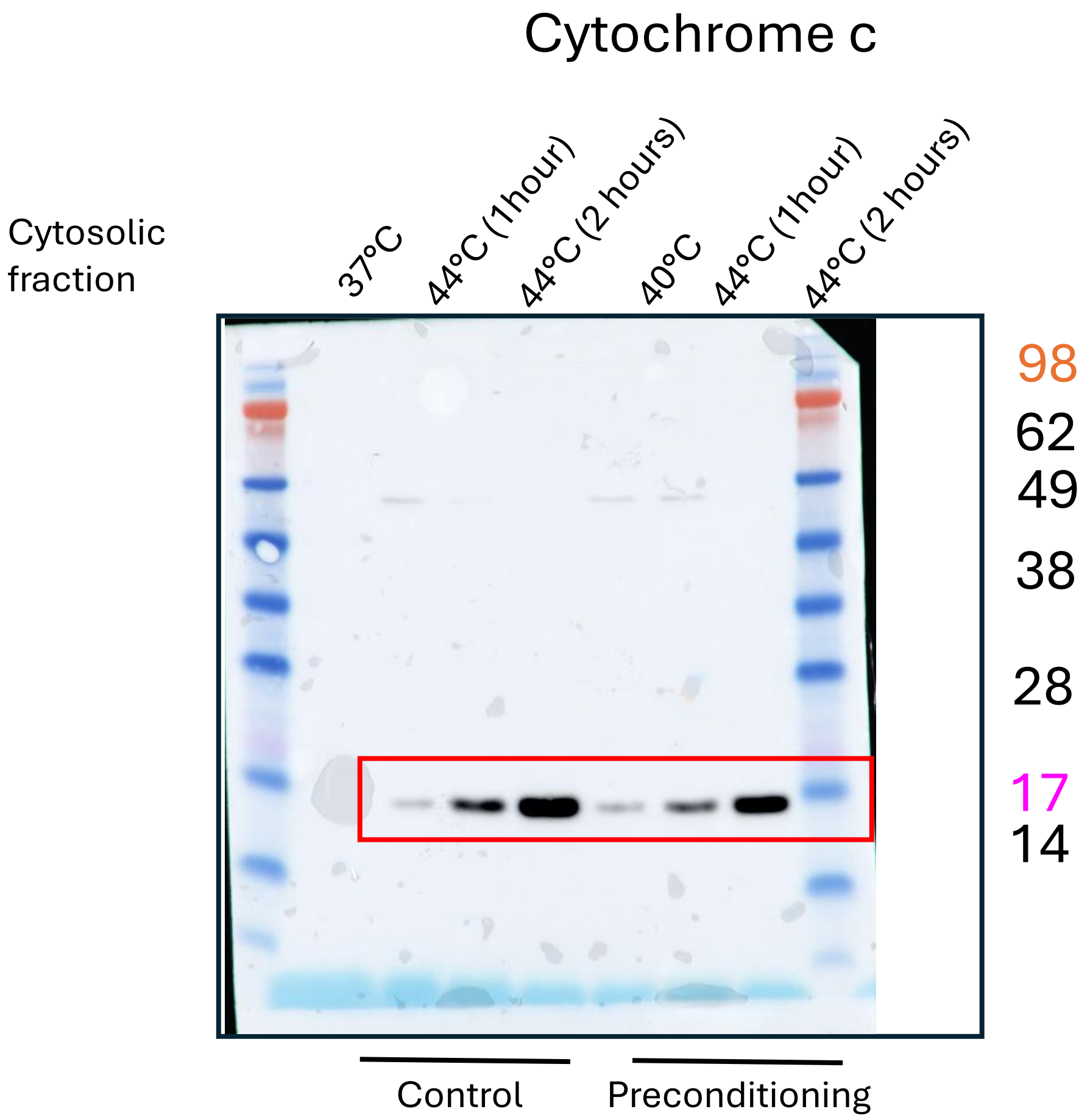

Bis-Tris 4-12% Gel
MES Buffer

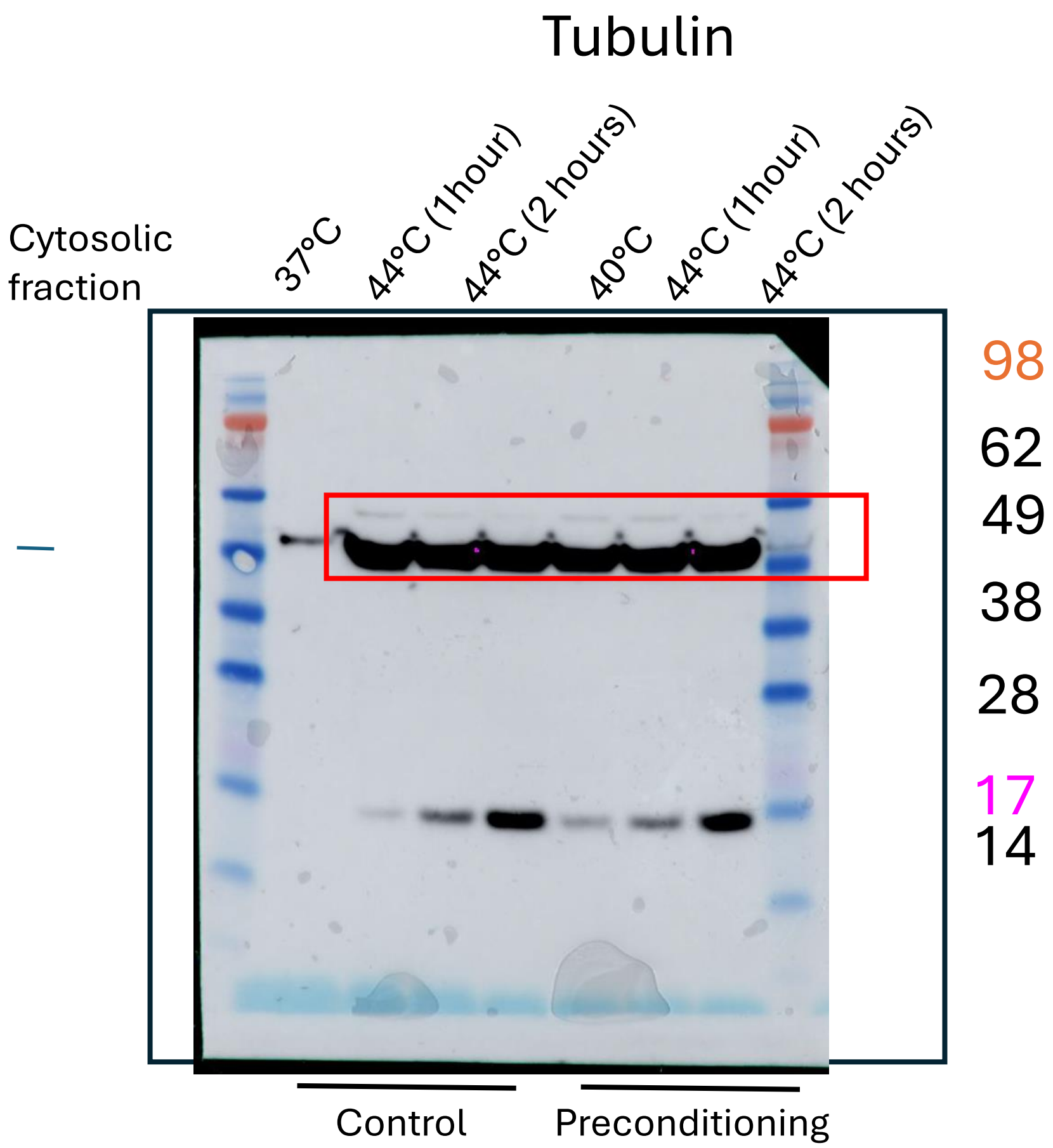

Bis-Tris 4-12% Gel
MES Buffer

Figure 3:
3G (Replicate 1)

Cytochrome c (used in figure)

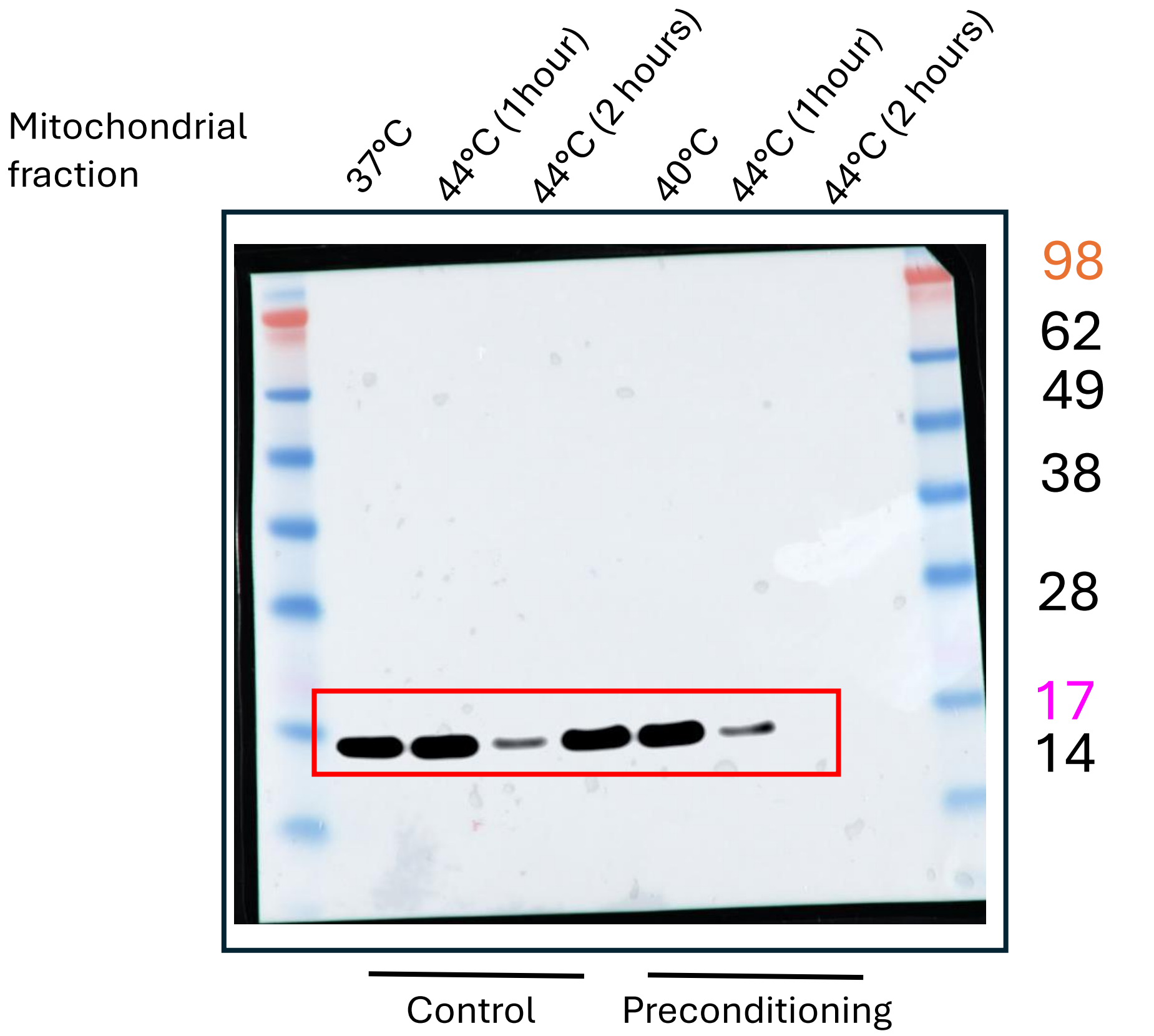

Bis-Tris 4-12% Gel
MES Buffer

VDAC1

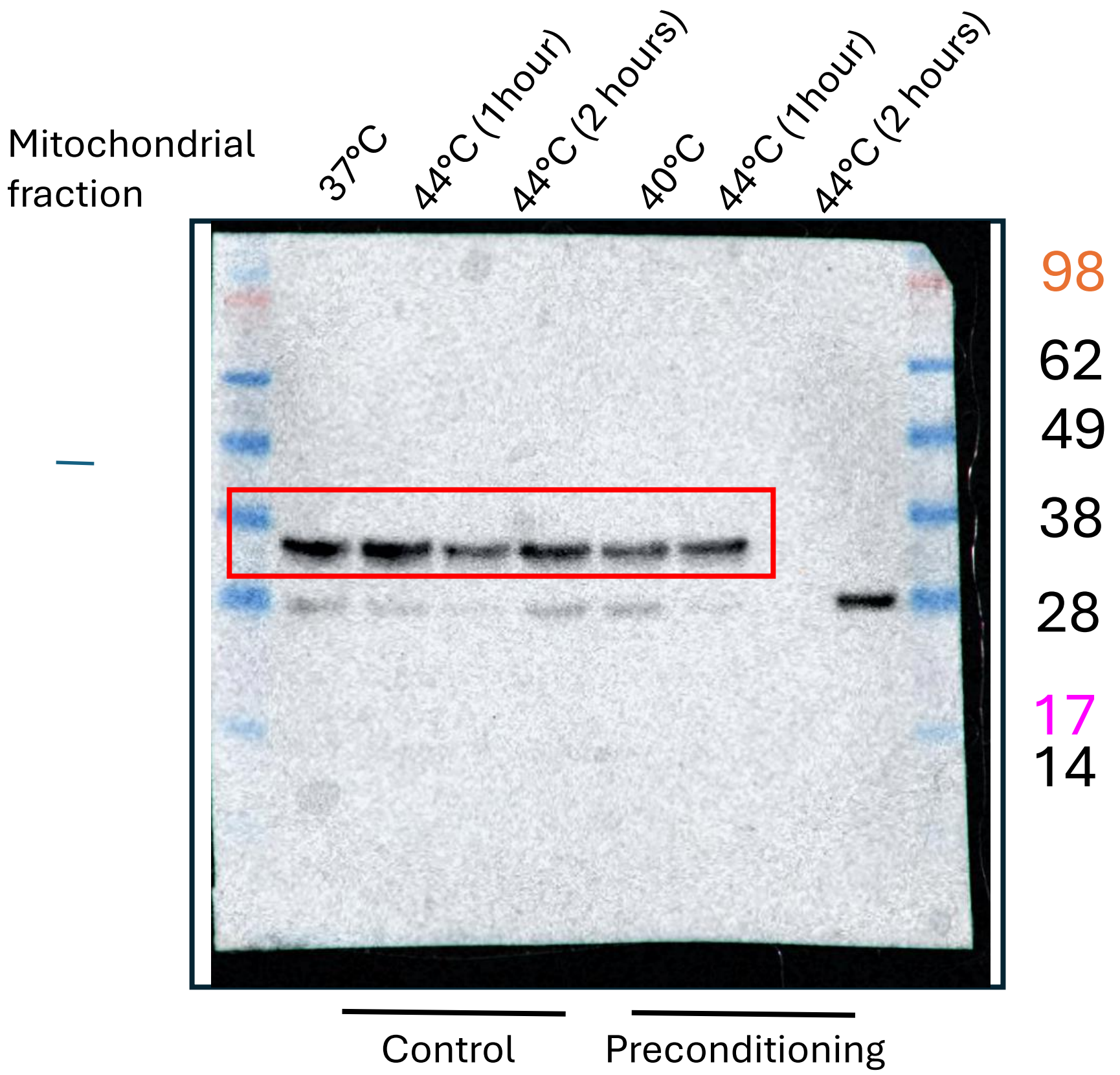

Bis-Tris 4-12% Gel
MES Buffer

Figure 3:
3G (Replicate 2)

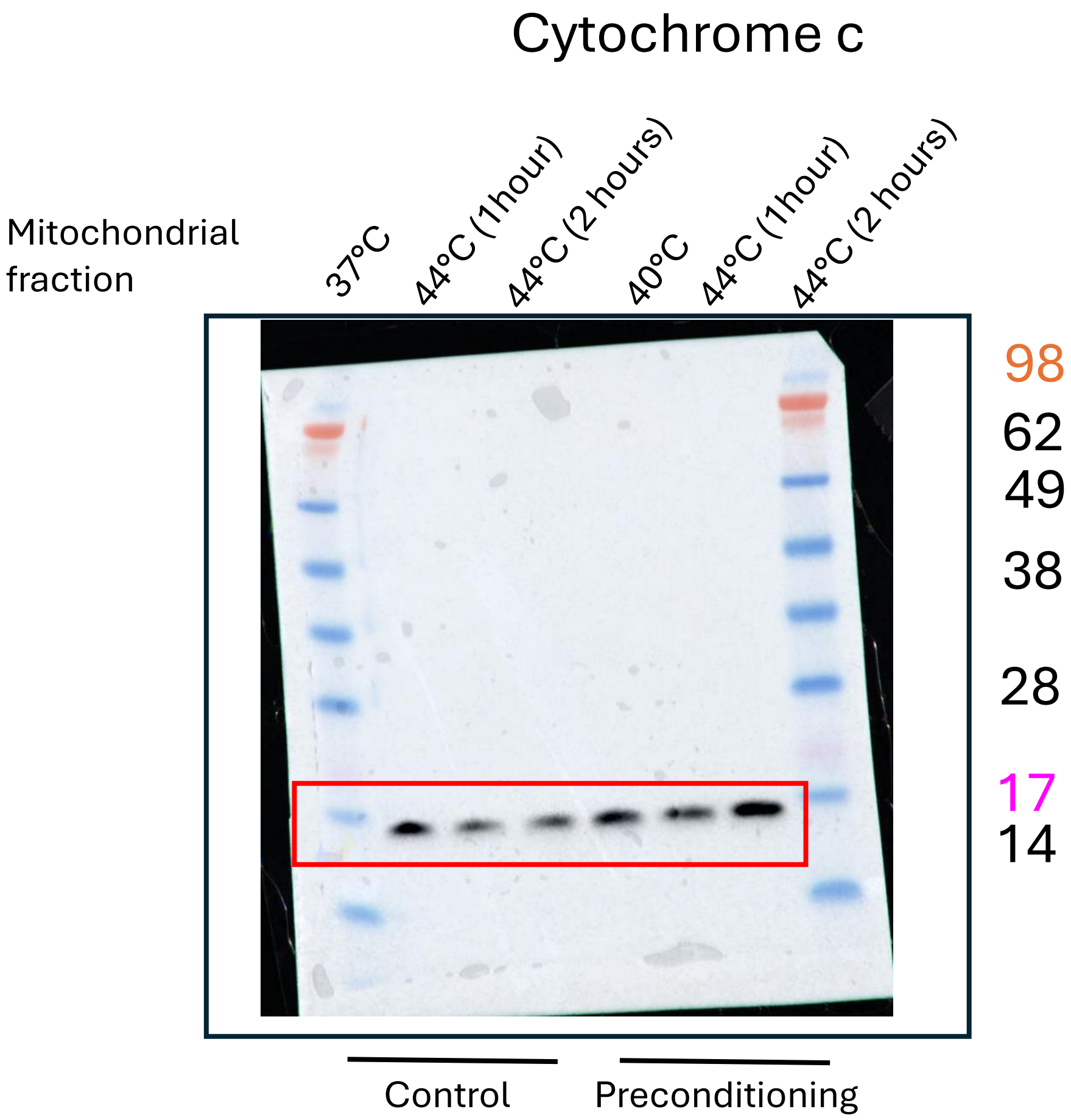

Bis-Tris 4-12% Gel
MES Buffer

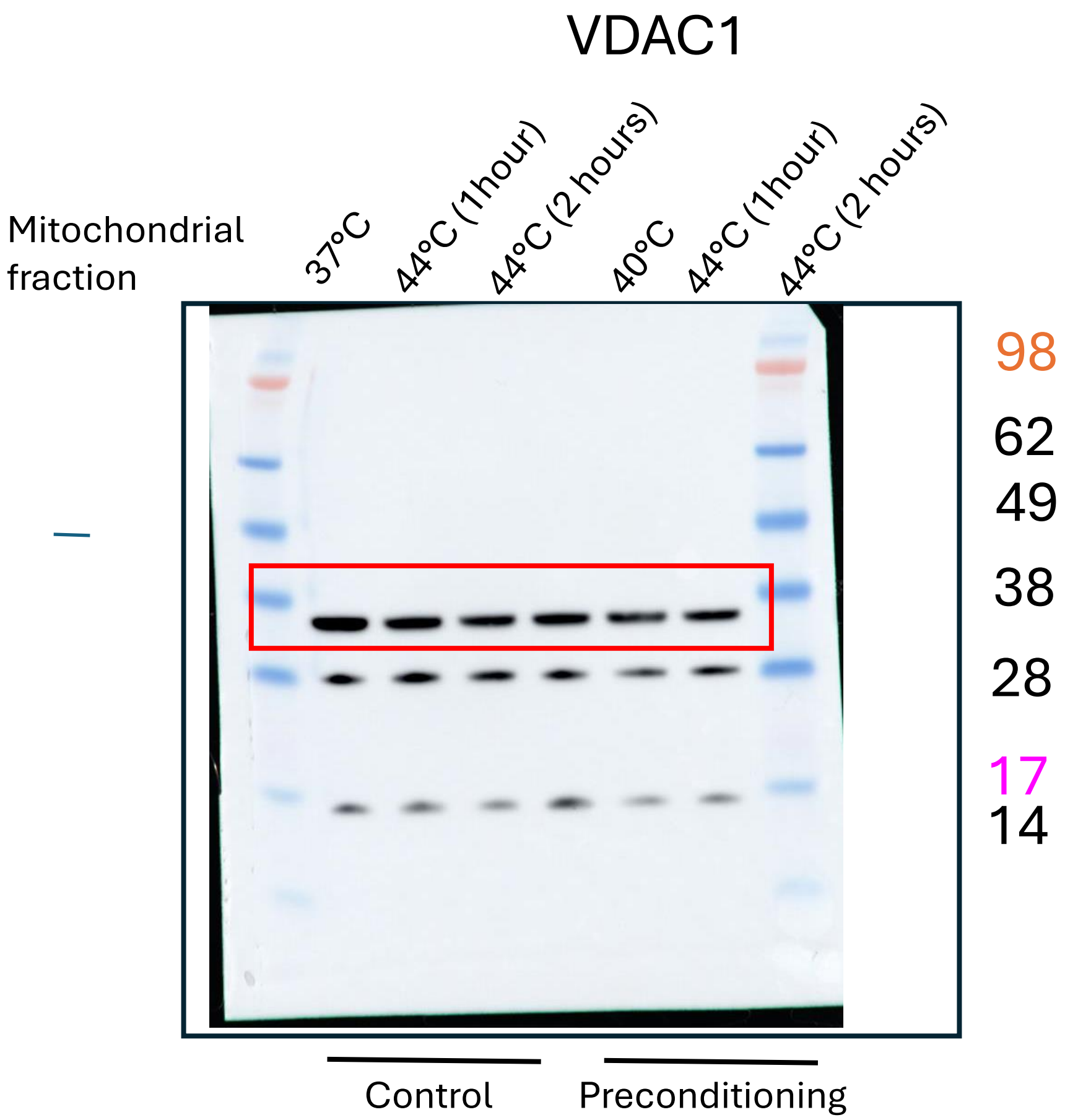

Bis-Tris 4-12% Gel
MES Buffer

Figure 3:
3G (Replicate 3)

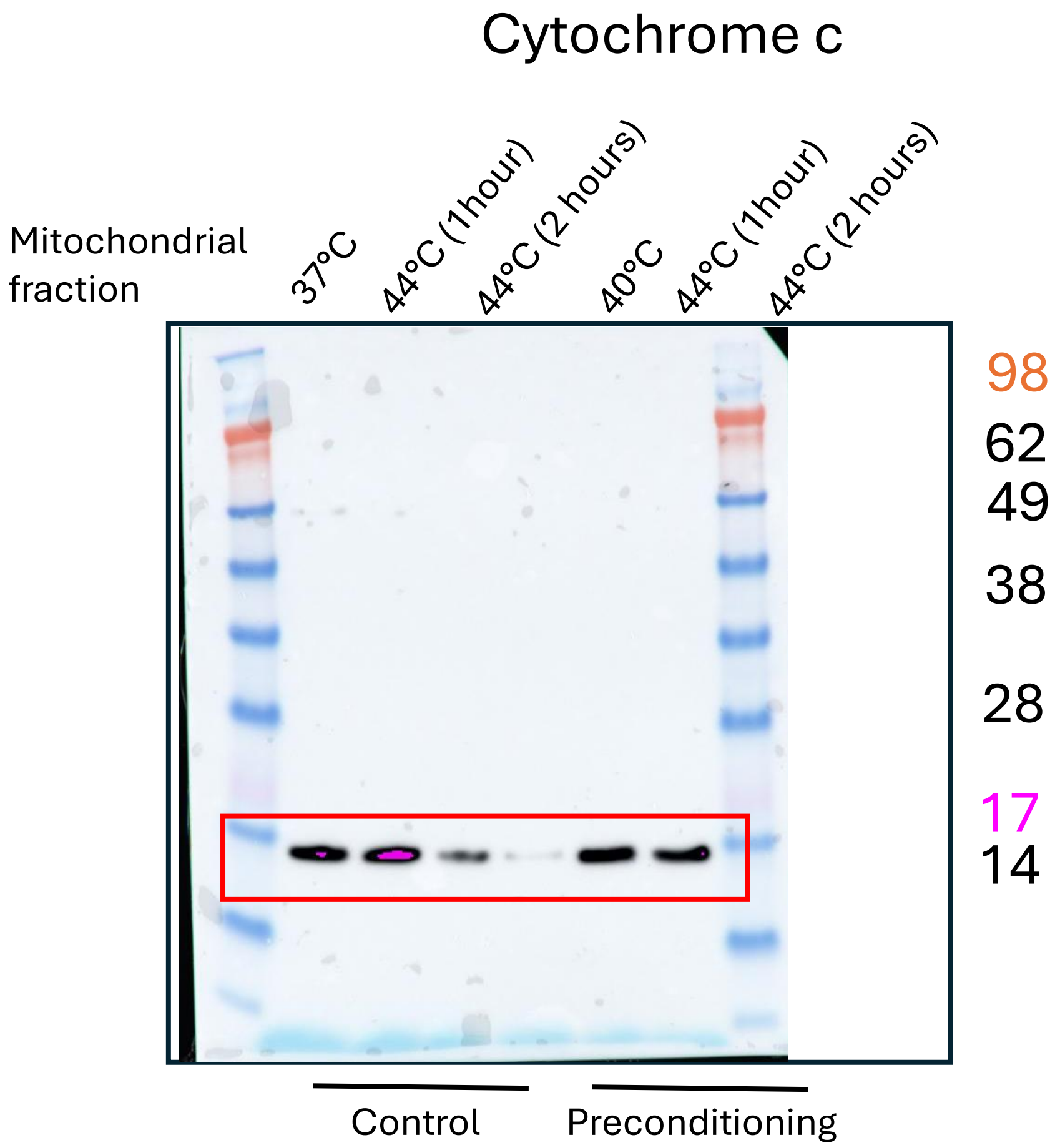

Bis-Tris 4-12% Gel
MES Buffer

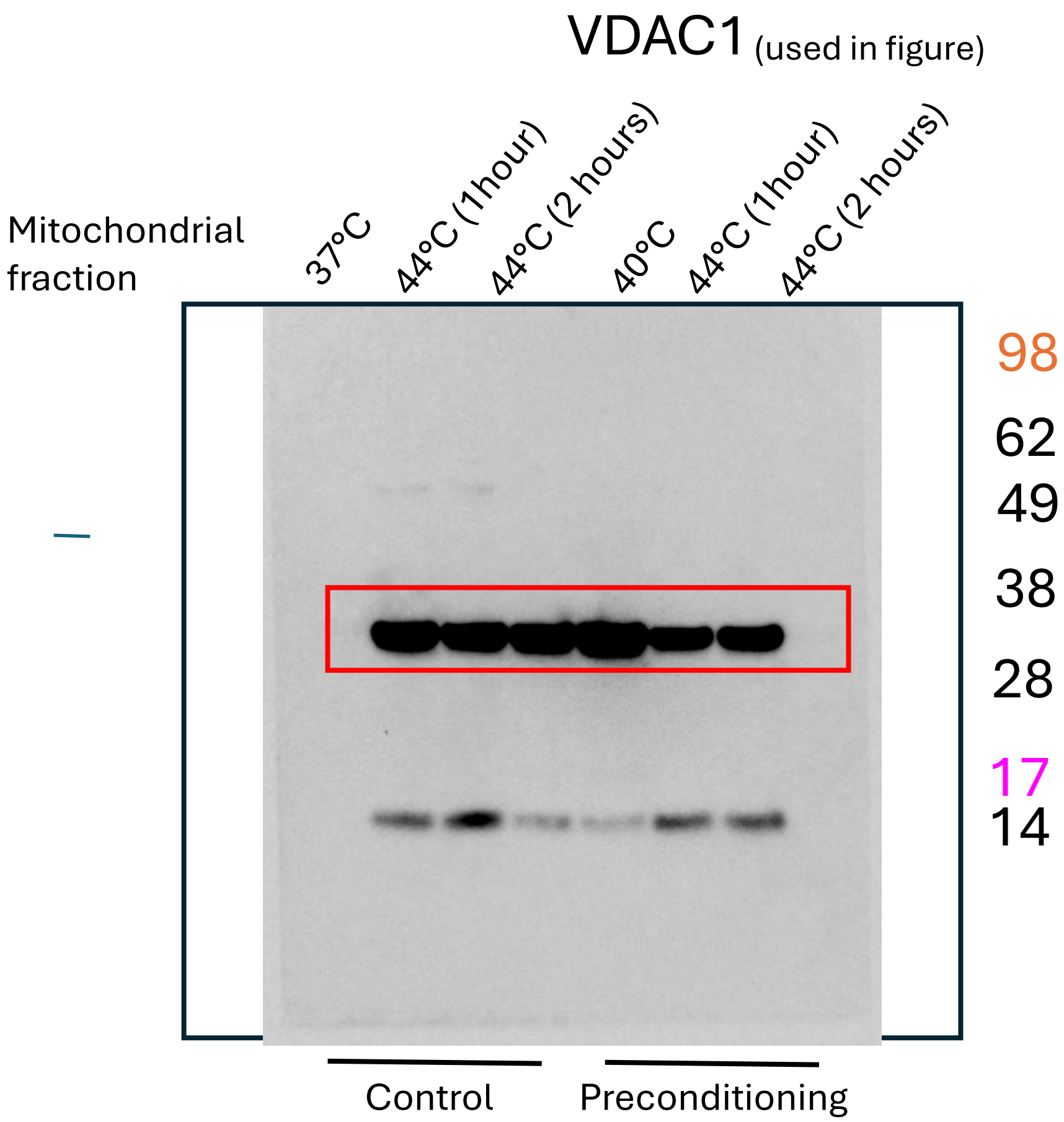

Bis-Tris 4-12% Gel
MES Buffer

Figure 3:
3I (Replicate 1)

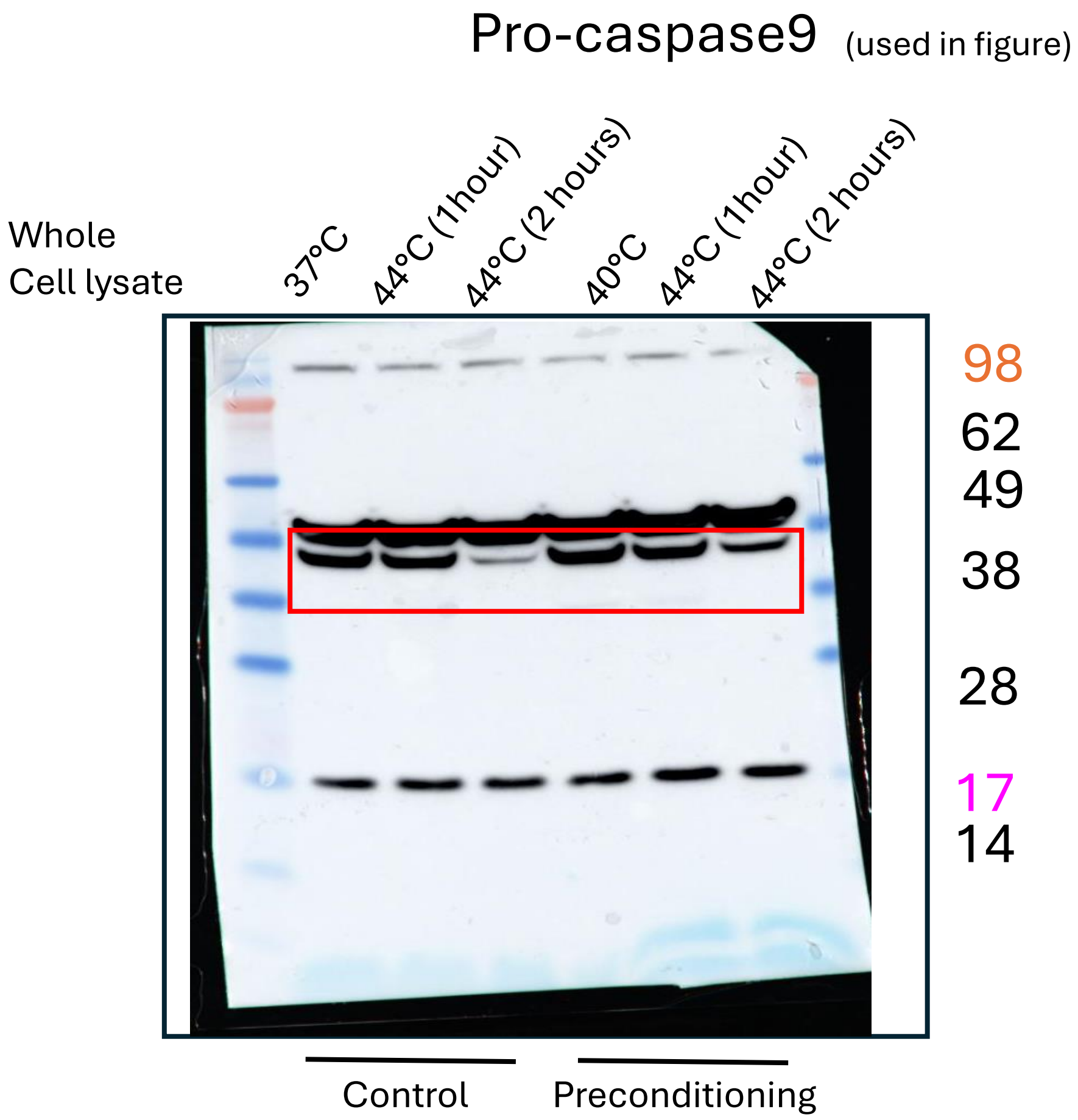

Bis-Tris 4-12% Gel
MES Buffer

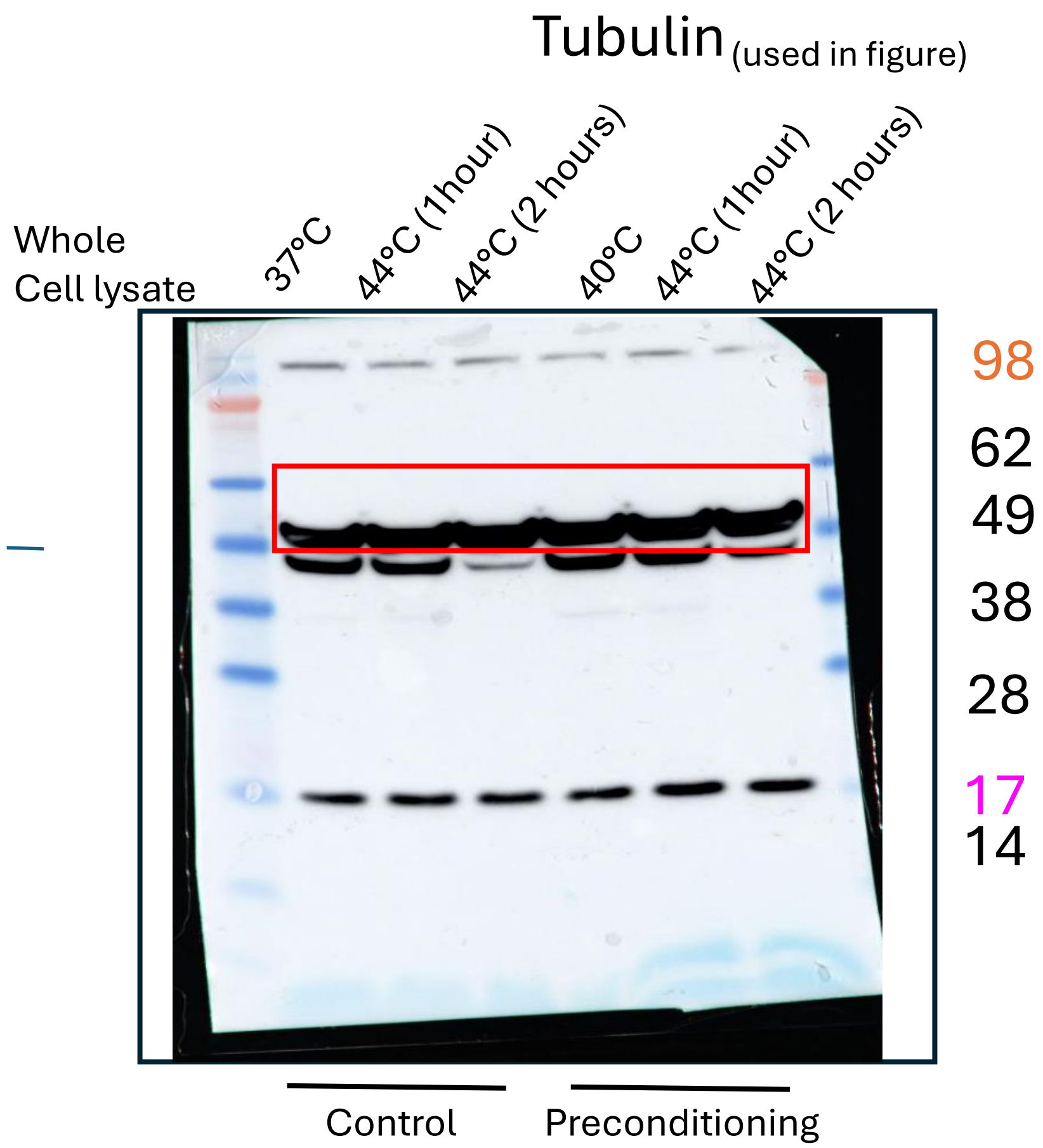

Bis-Tris 4-12% Gel
MES Buffer

Figure 3:
3I (Replicate 2)

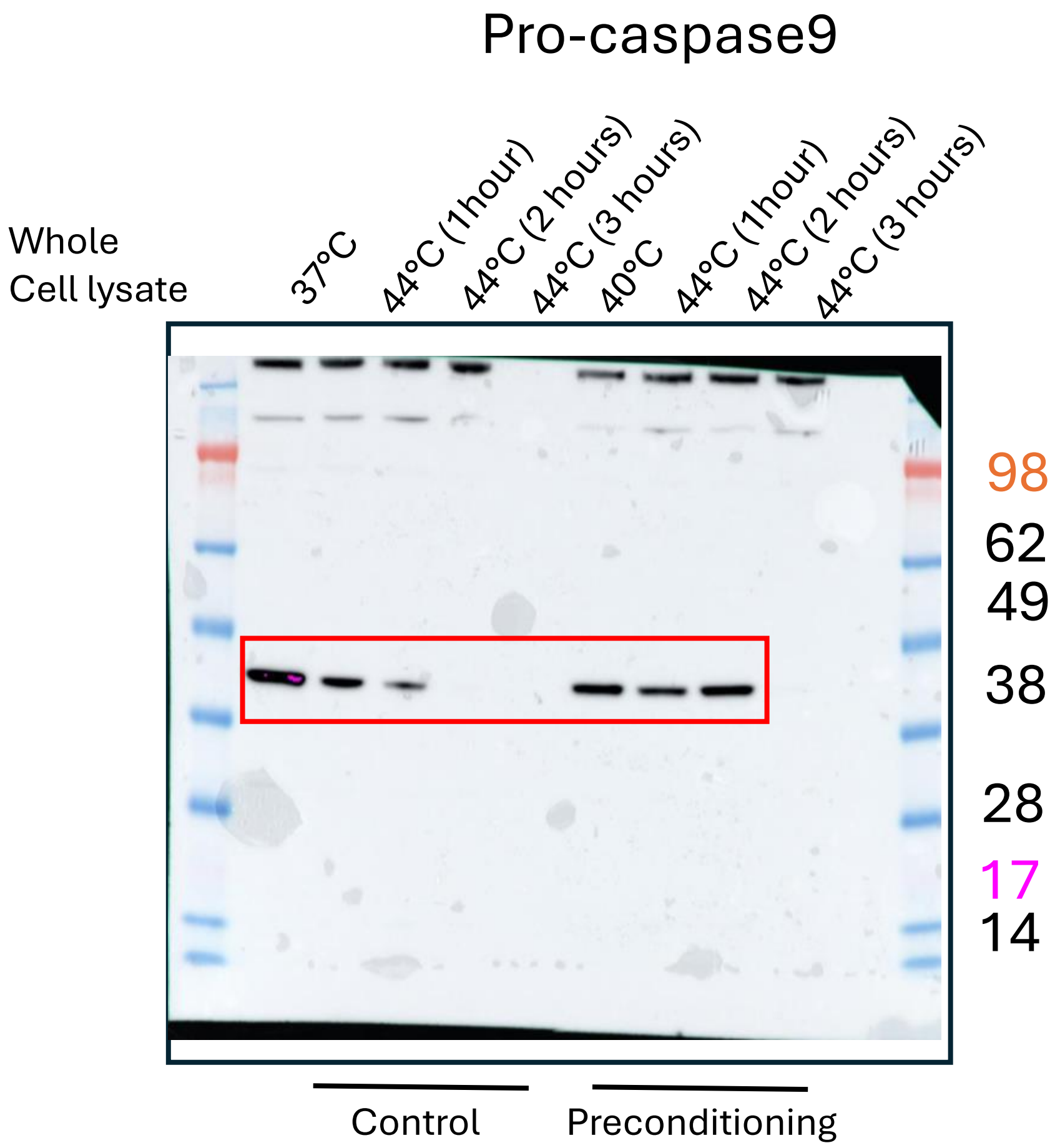

Bis-Tris 4-12% Gel
MES Buffer

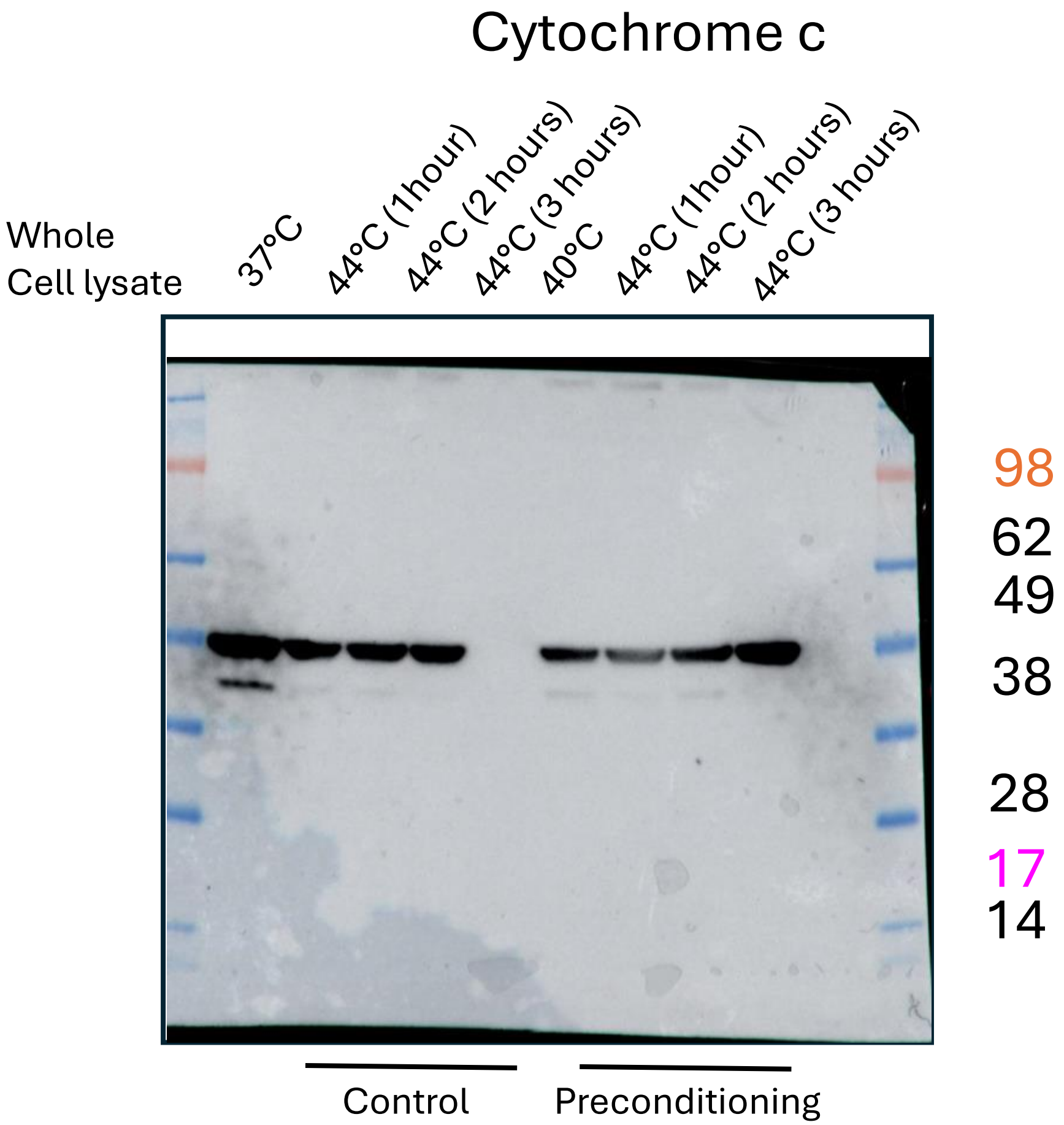

Bis-Tris 4-12% Gel
MES Buffer

Figure 3:
3I (Replicate 3)

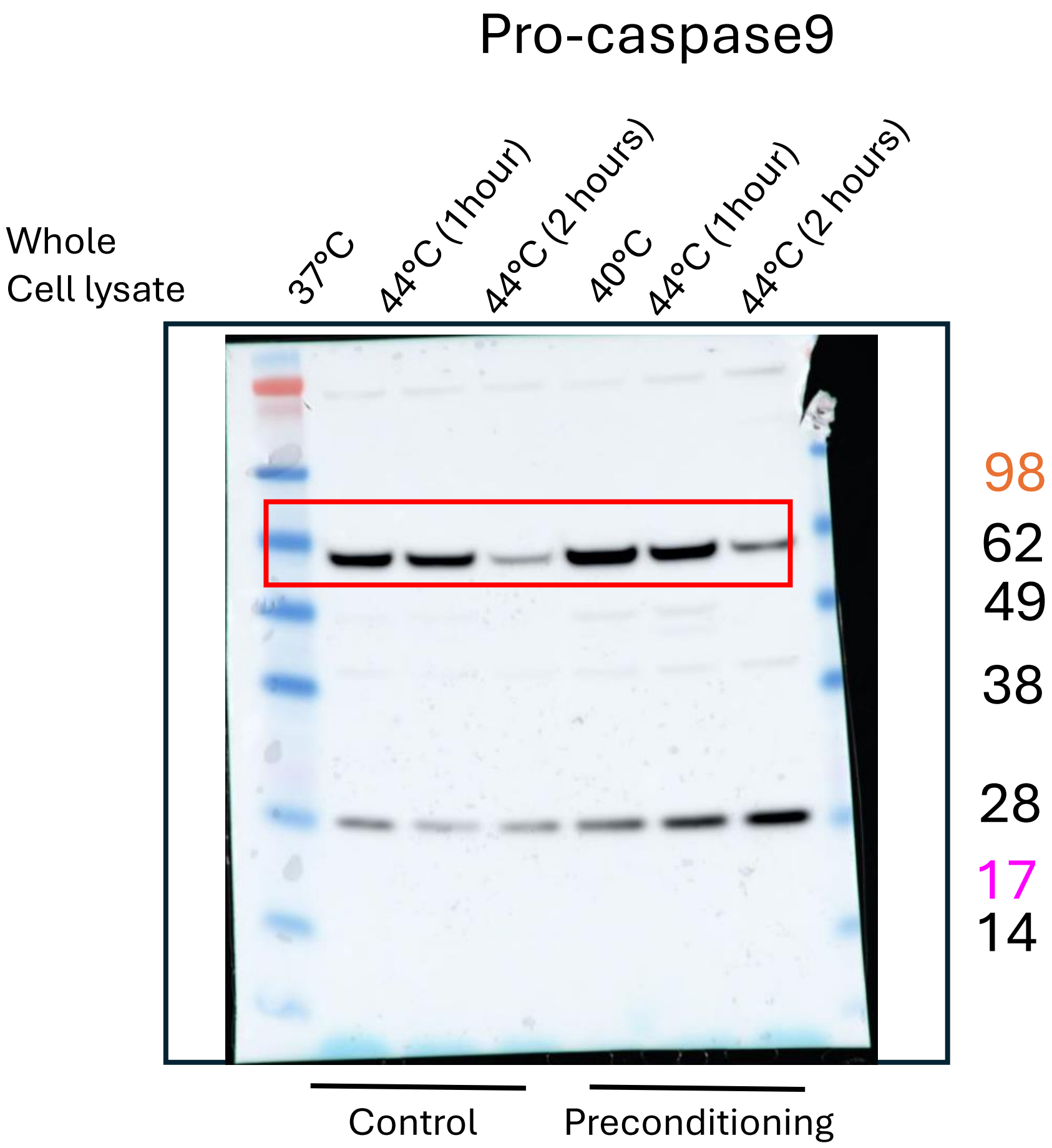

Bis-Tris 4-12% Gel
MES Buffer

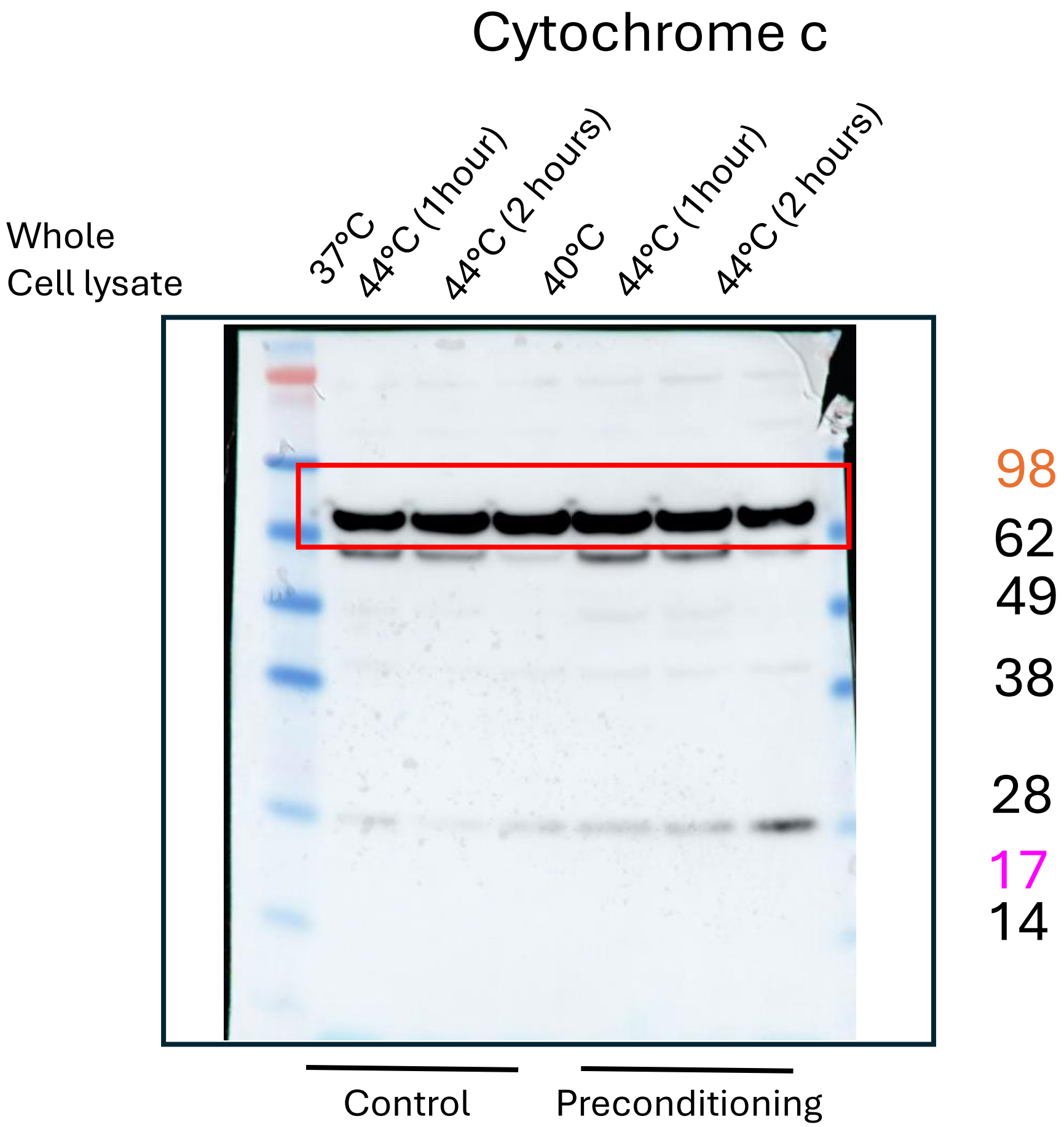

Bis-Tris 4-12% Gel
MES Buffer

Figure 3:
3J (Replicate 1)

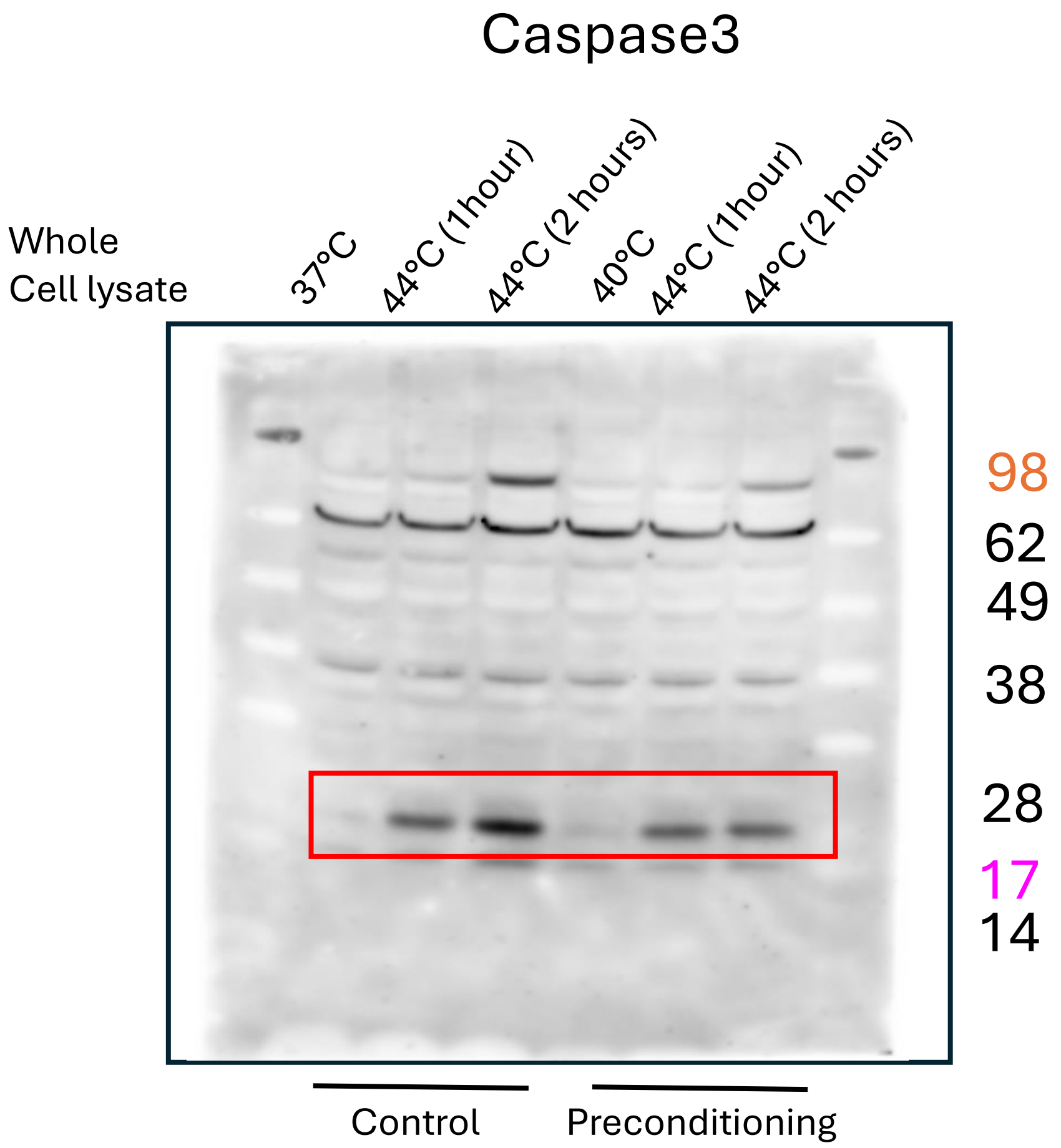

Bis-Tris 4-12% Gel
MES Buffer

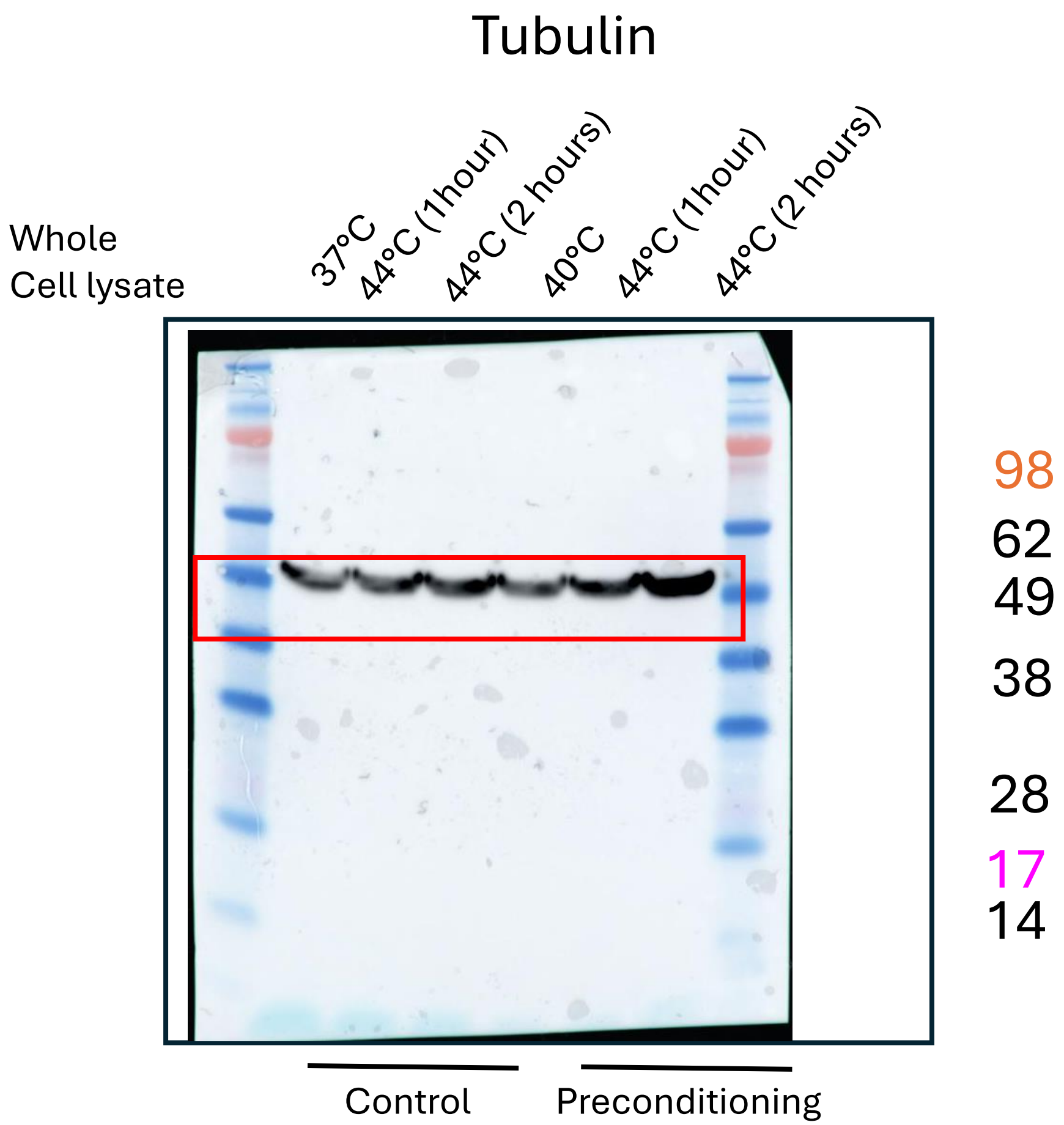

Bis-Tris 4-12% Gel
MES Buffer

Figure 3:
3J (Replicate 2)

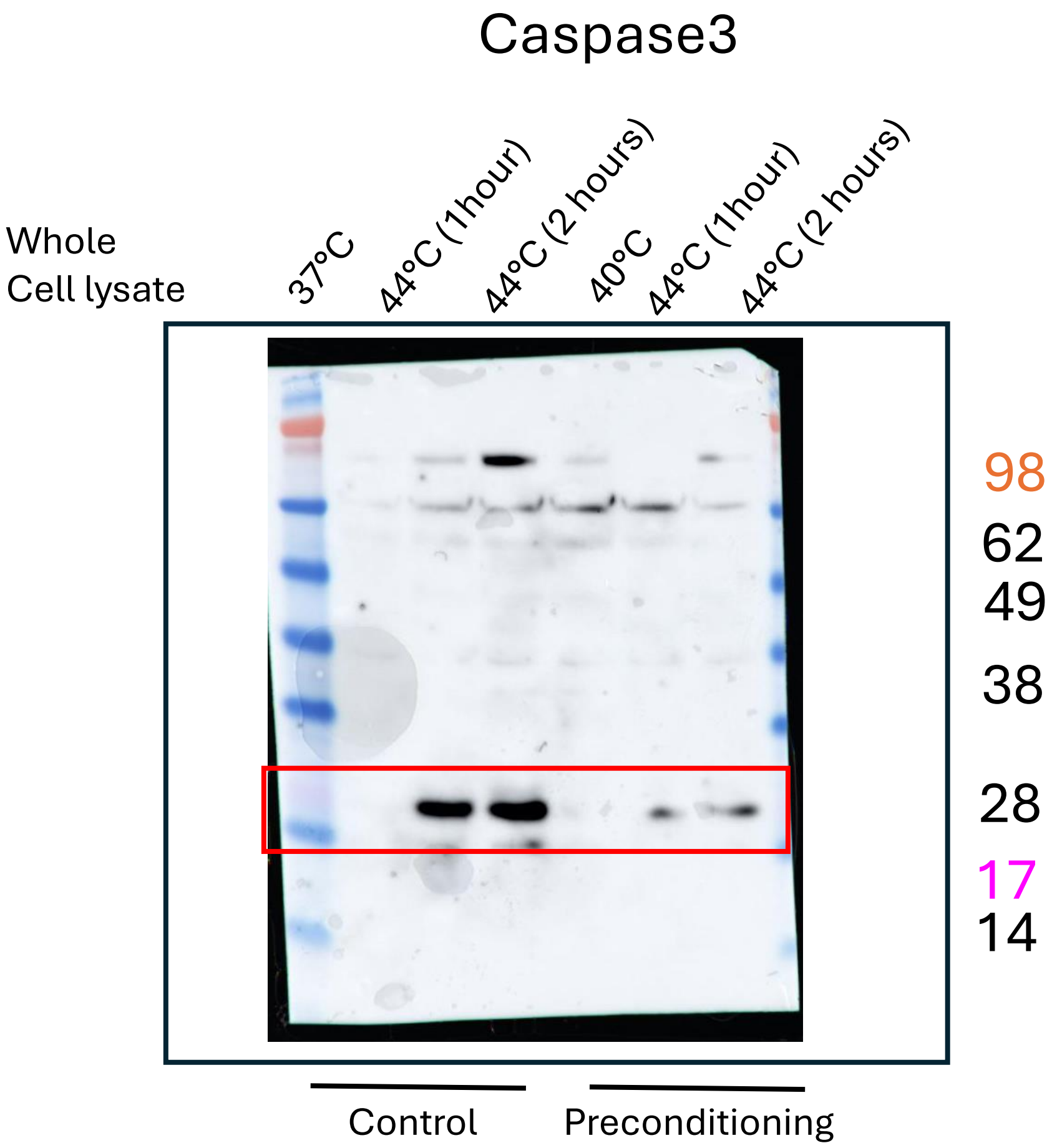

Bis-Tris 4-12% Gel
MES Buffer

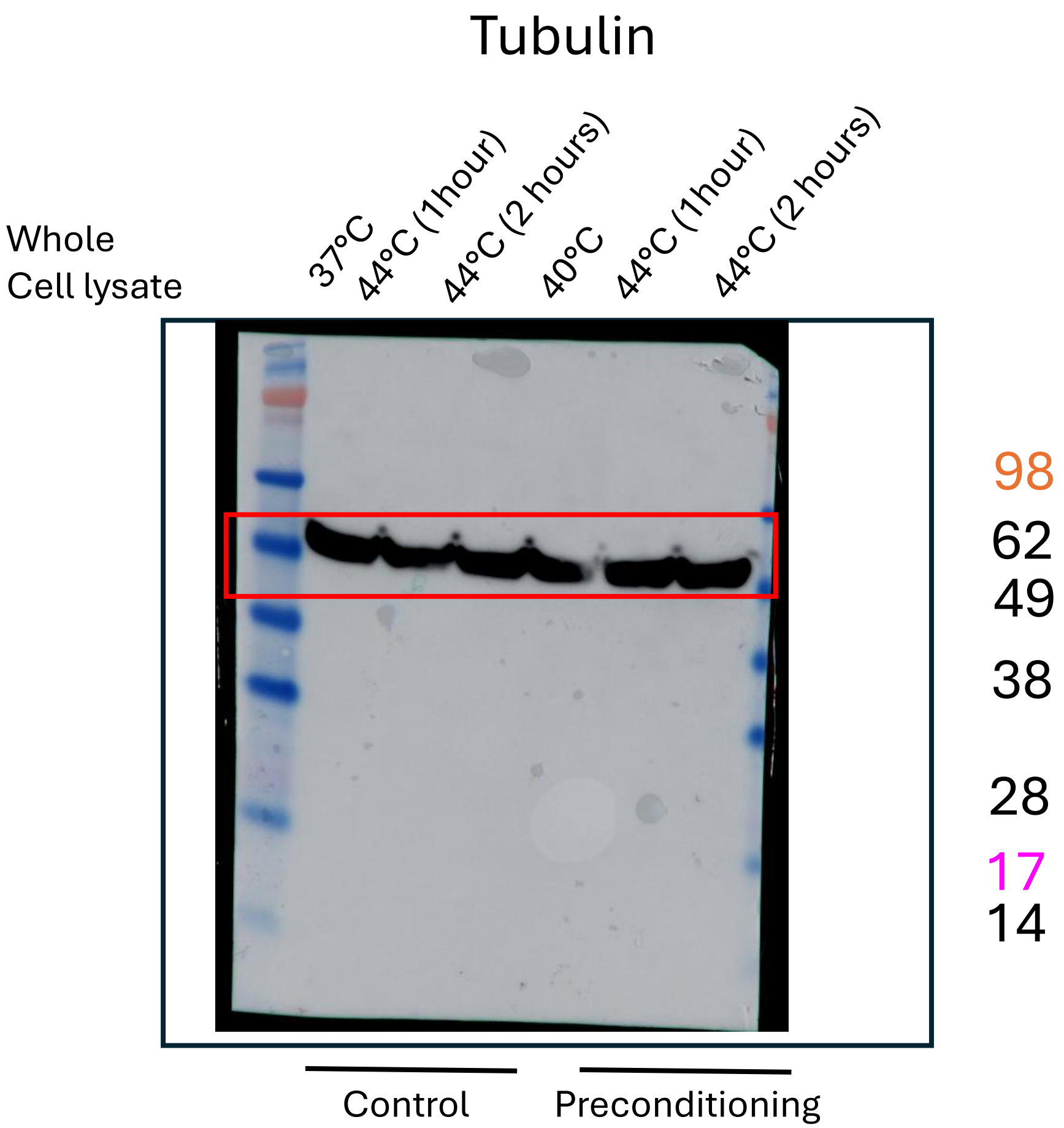

Bis-Tris 4-12% Gel
MES Buffer

Figure 3:
3J (Replicate 3)

Caspase3

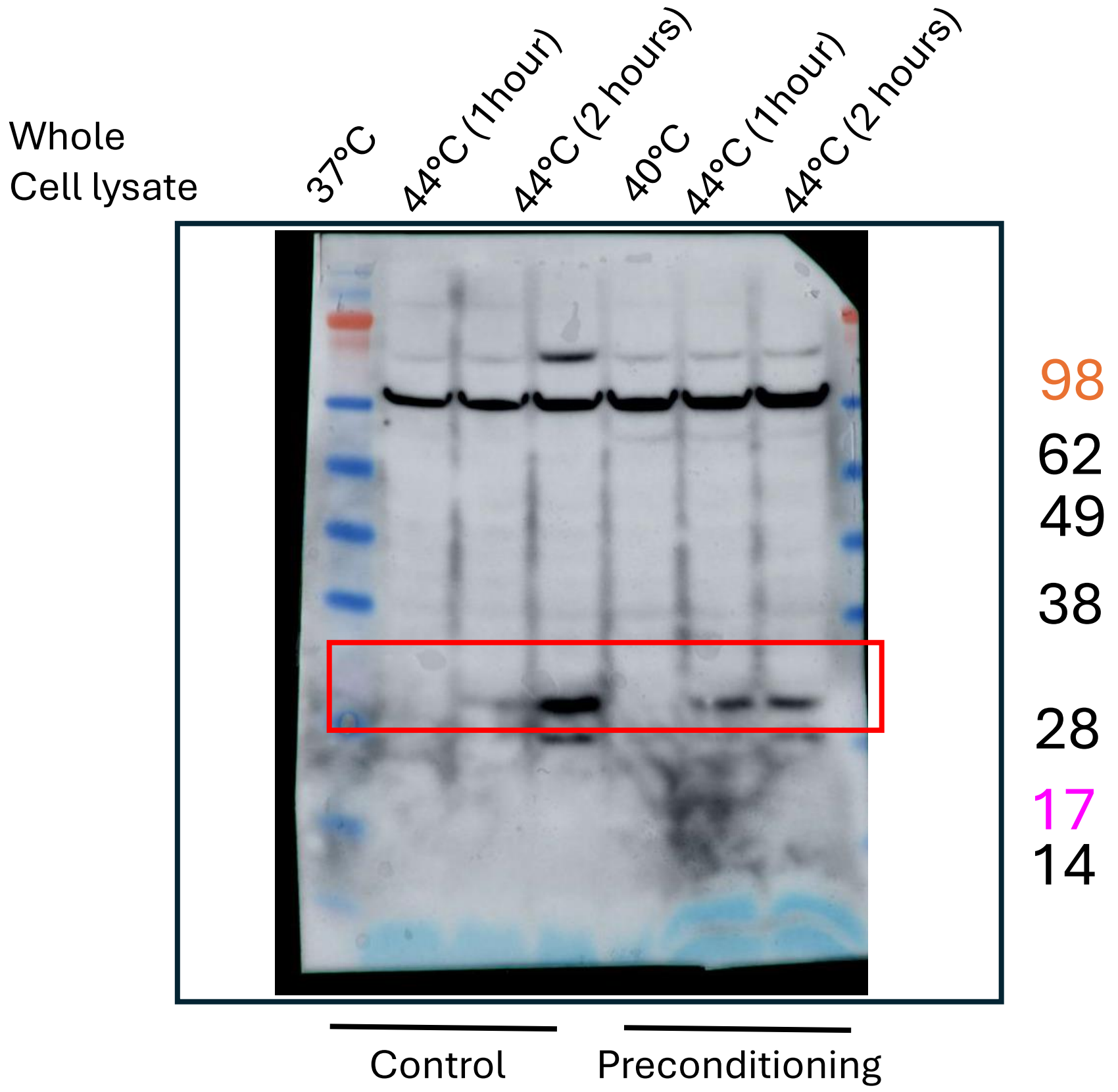

Bis-Tris 4-12% Gel
MES Buffer

Tubulin(used in figure)

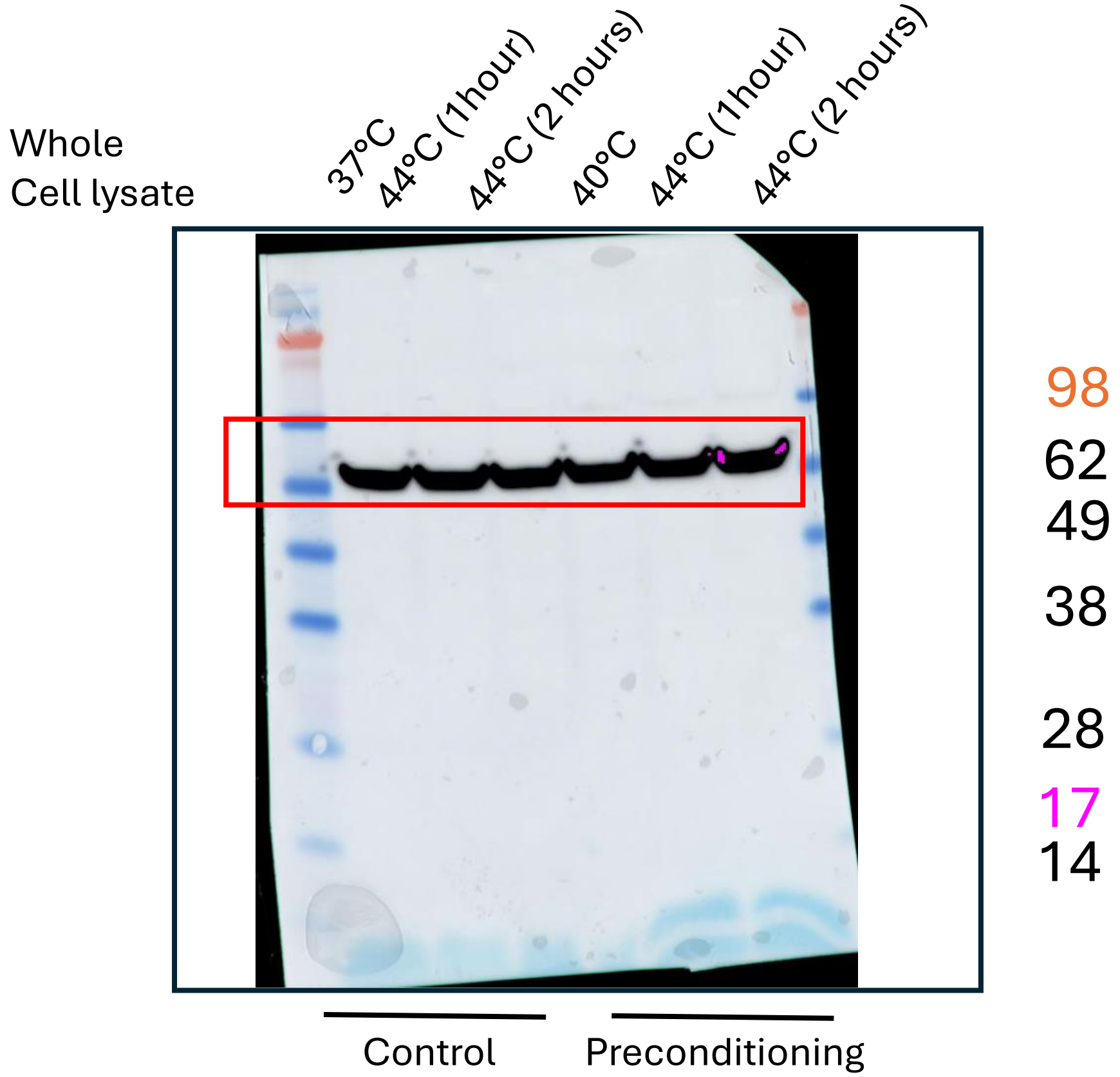

Bis-Tris 4-12% Gel
MES Buffer

Figure 3:
3K (Replicate 1)

PARP/ Cleaved PARP (used in figure)

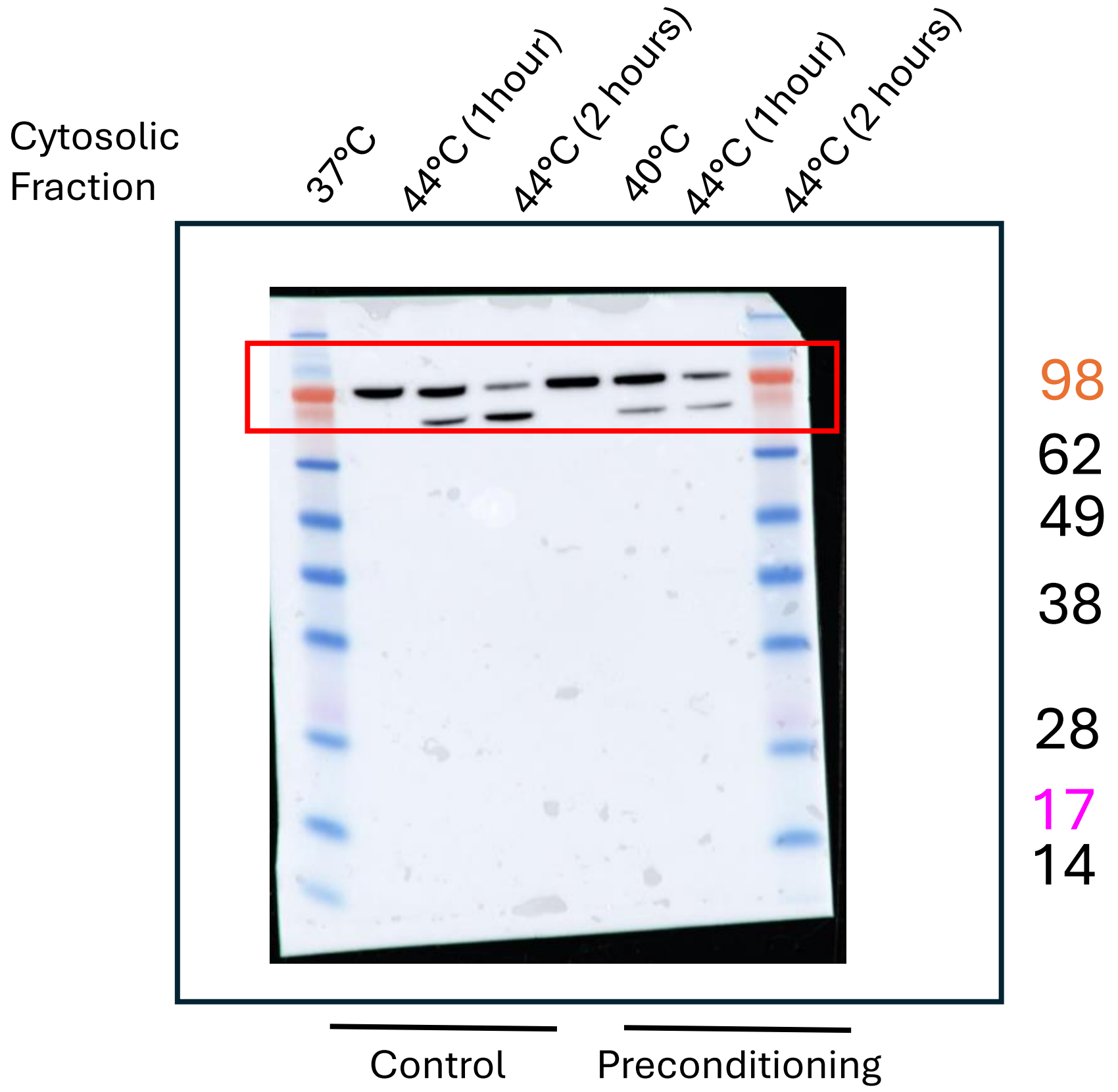

Bis-Tris 4-12% Gel
MES Buffer

Tubulin(used in figure)

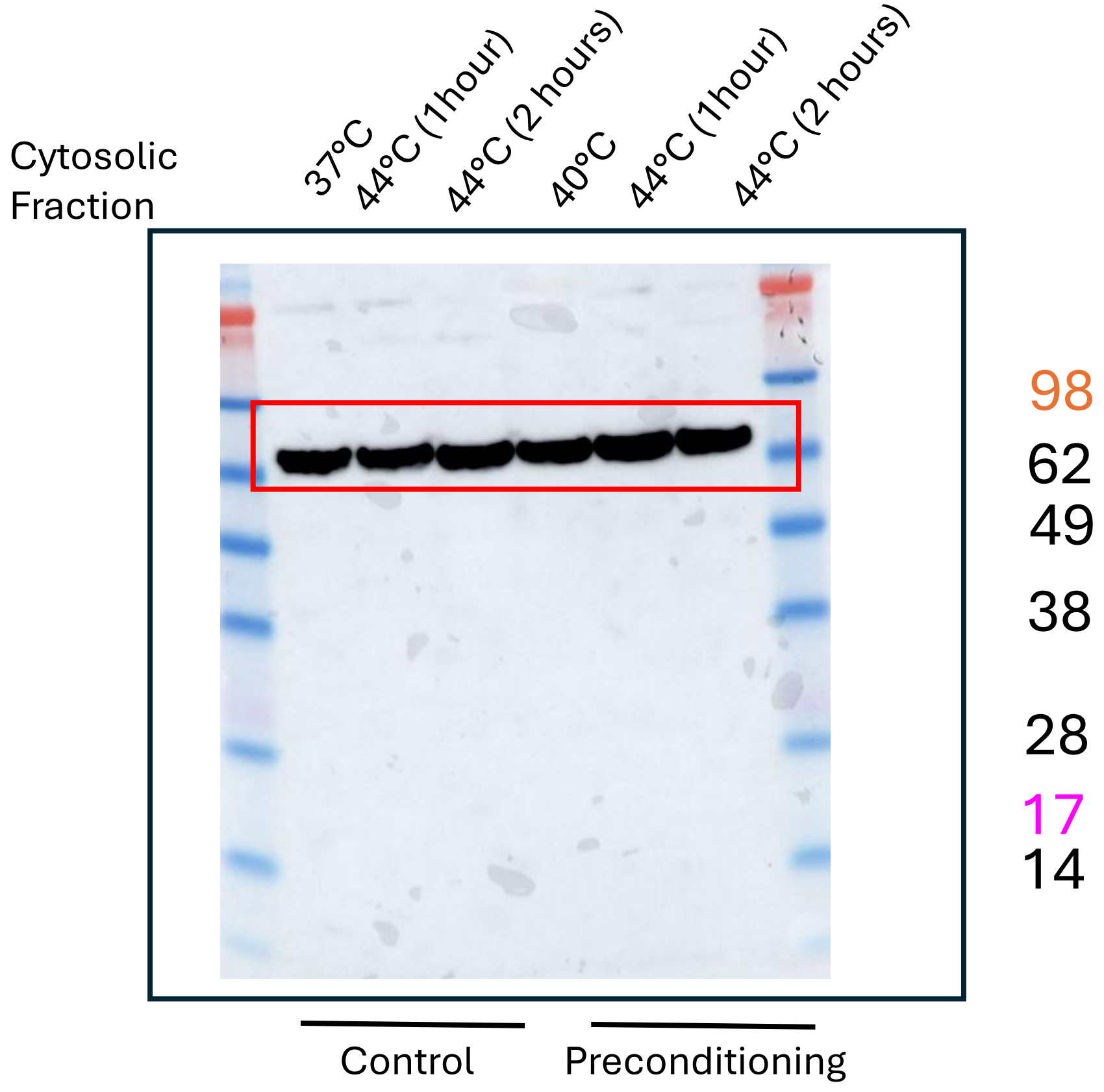

Bis-Tris 4-12% Gel
MES Buffer

Figure 3:
3K (Replicate 2)

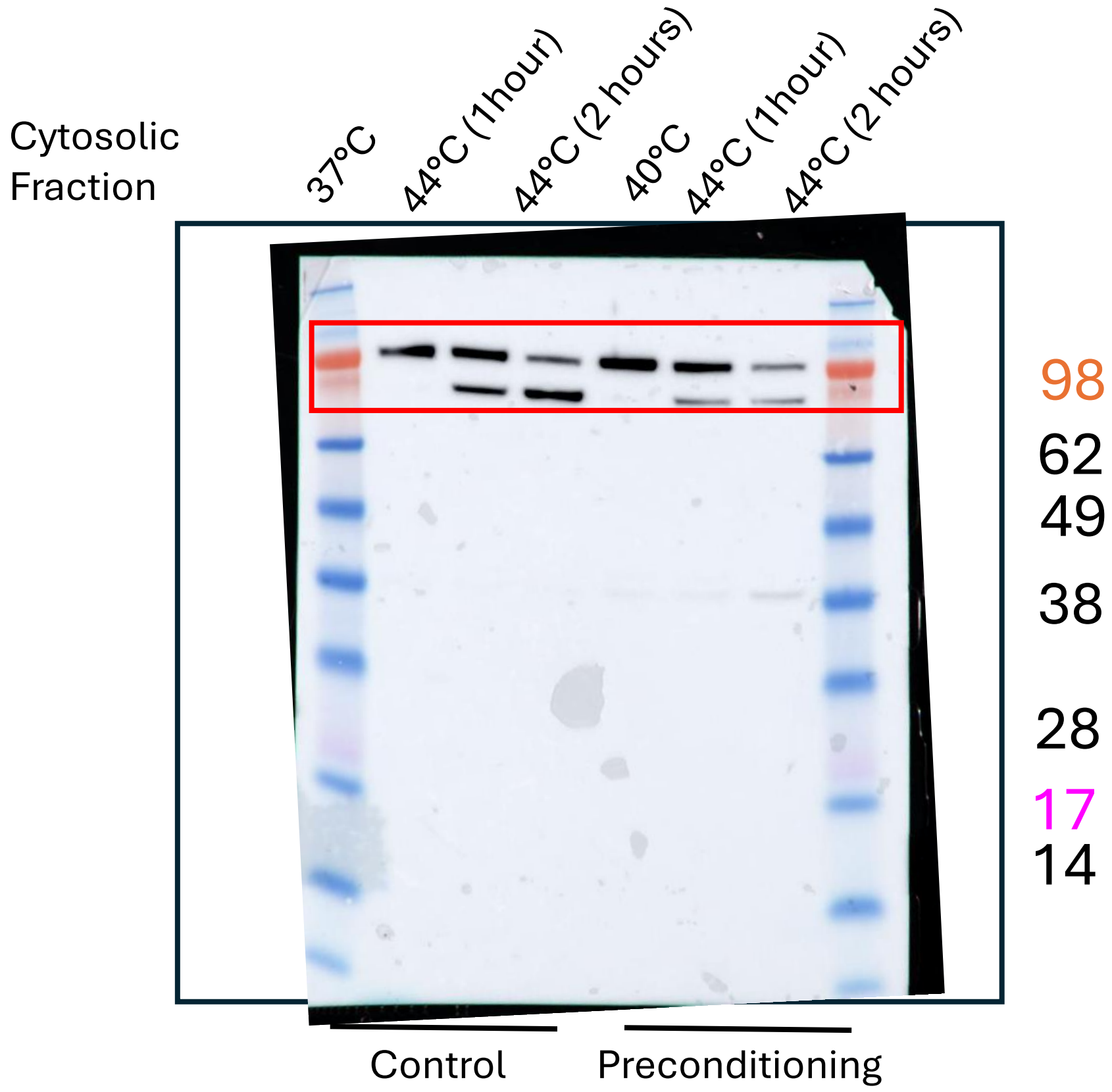

Bis-Tris 4-12% Gel
MES Buffer

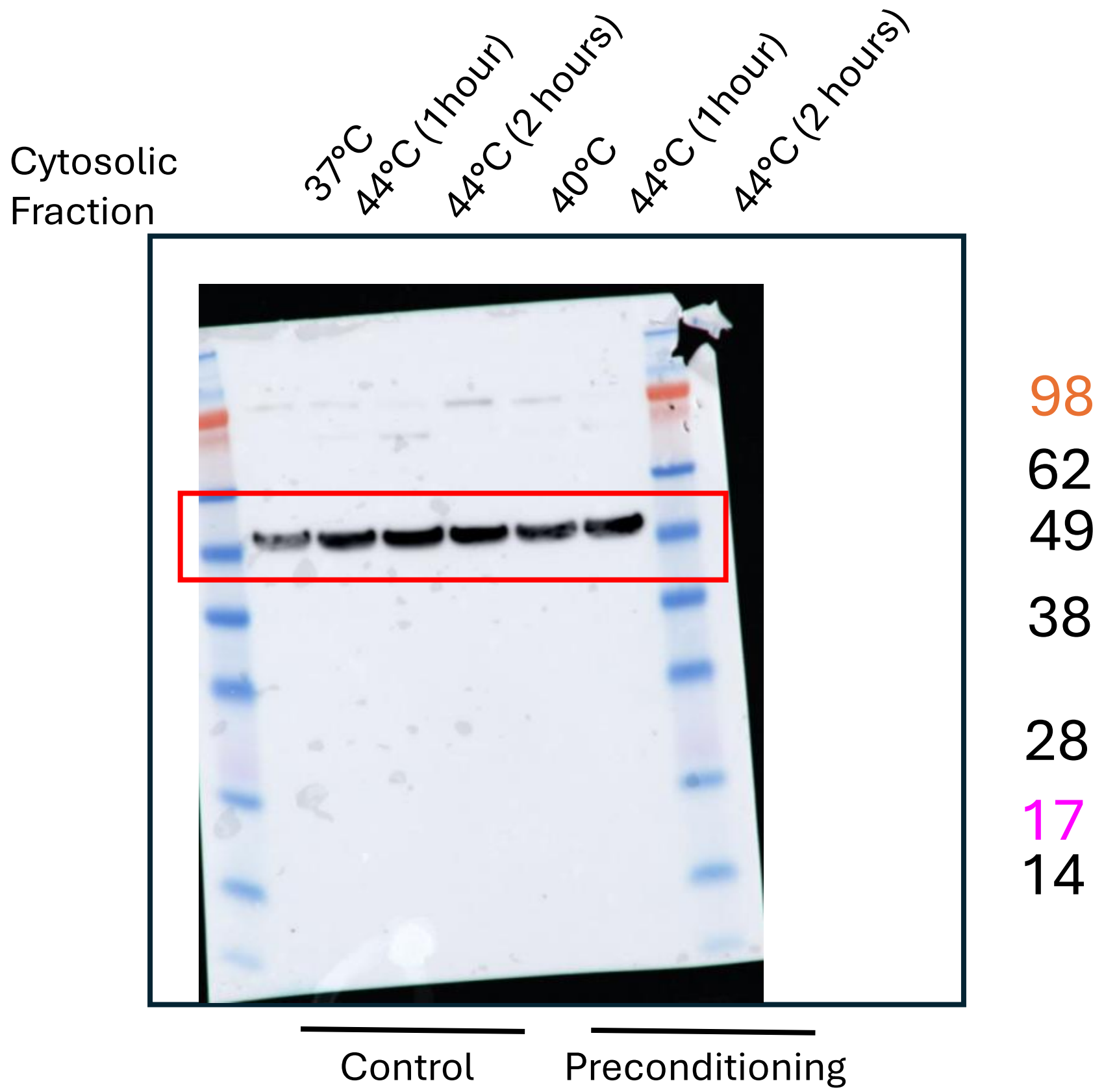

Bis-Tris 4-12% Gel
MES Buffer

Figure 3:
3K (Replicate 3)

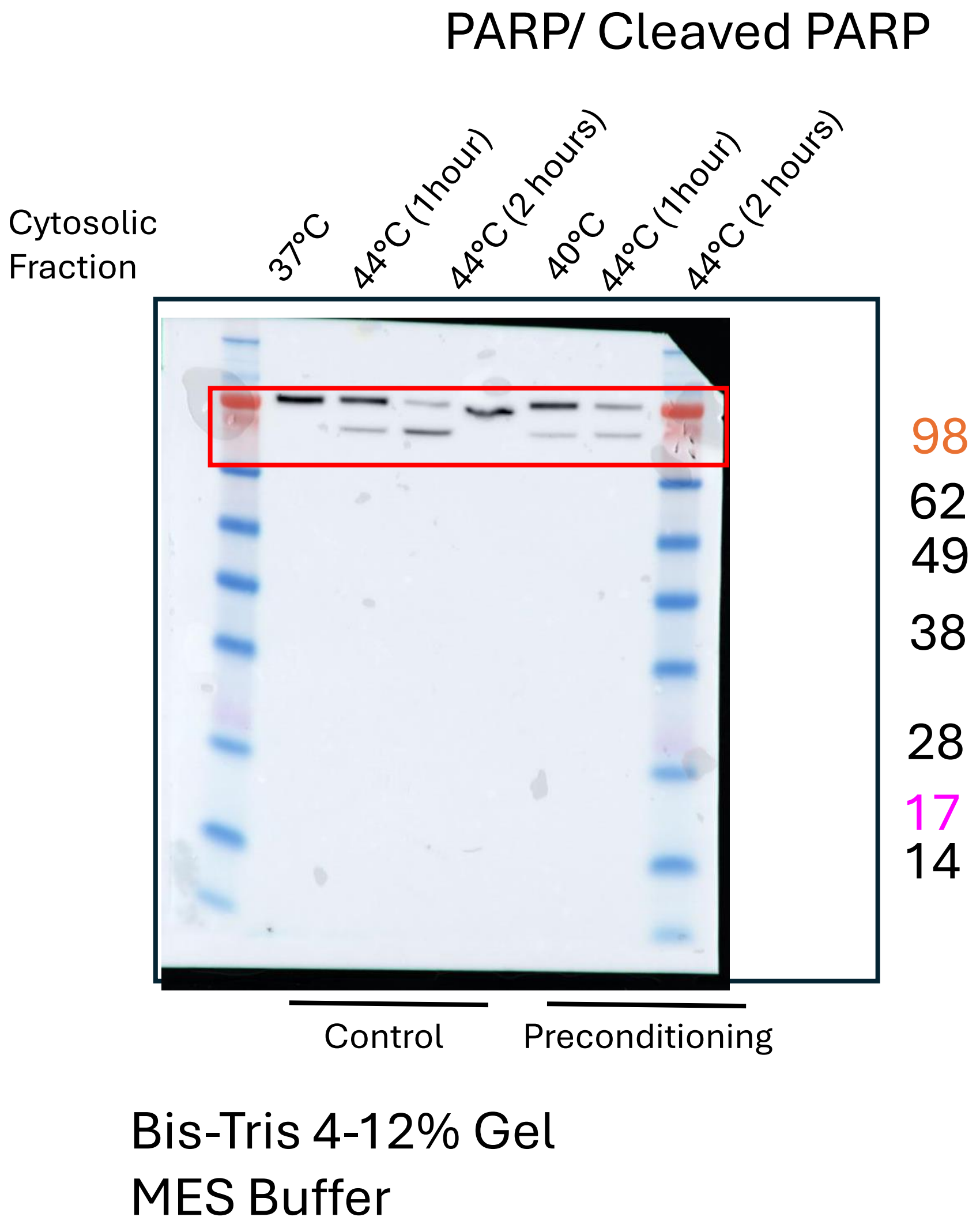

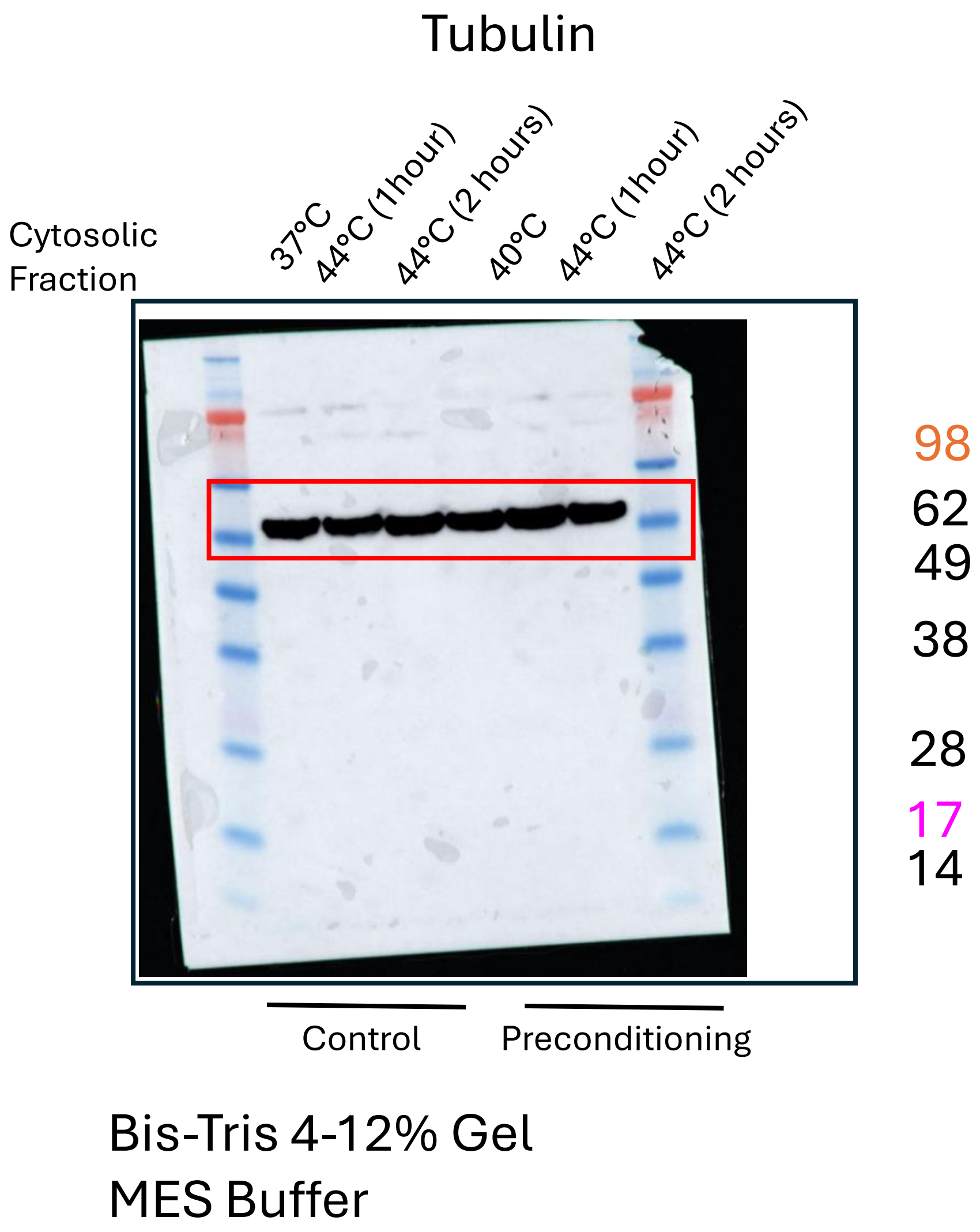

Figure 4:
4A (Replicate1)

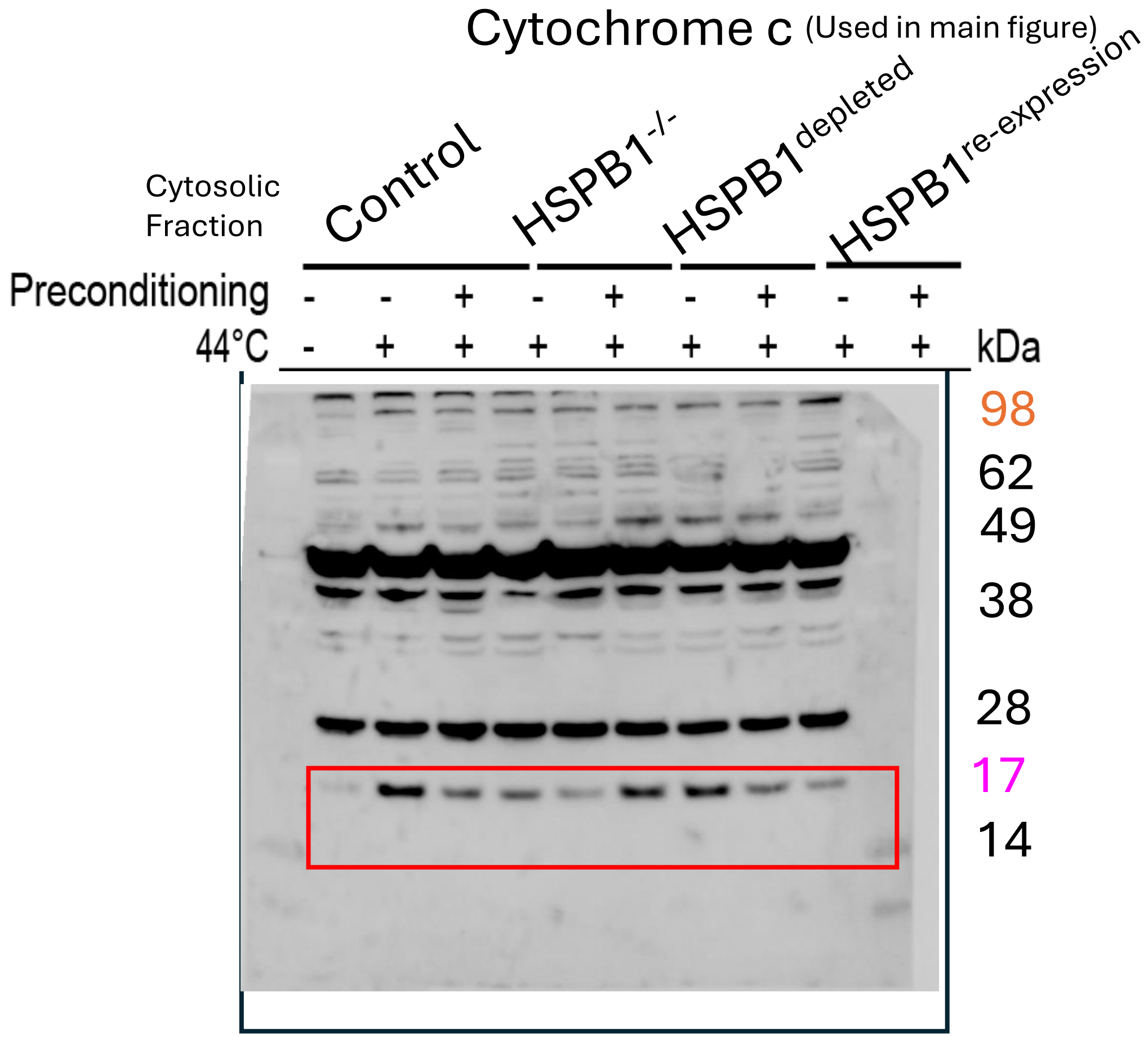

Bis-Tris 4-12% Gel
MES Buffer

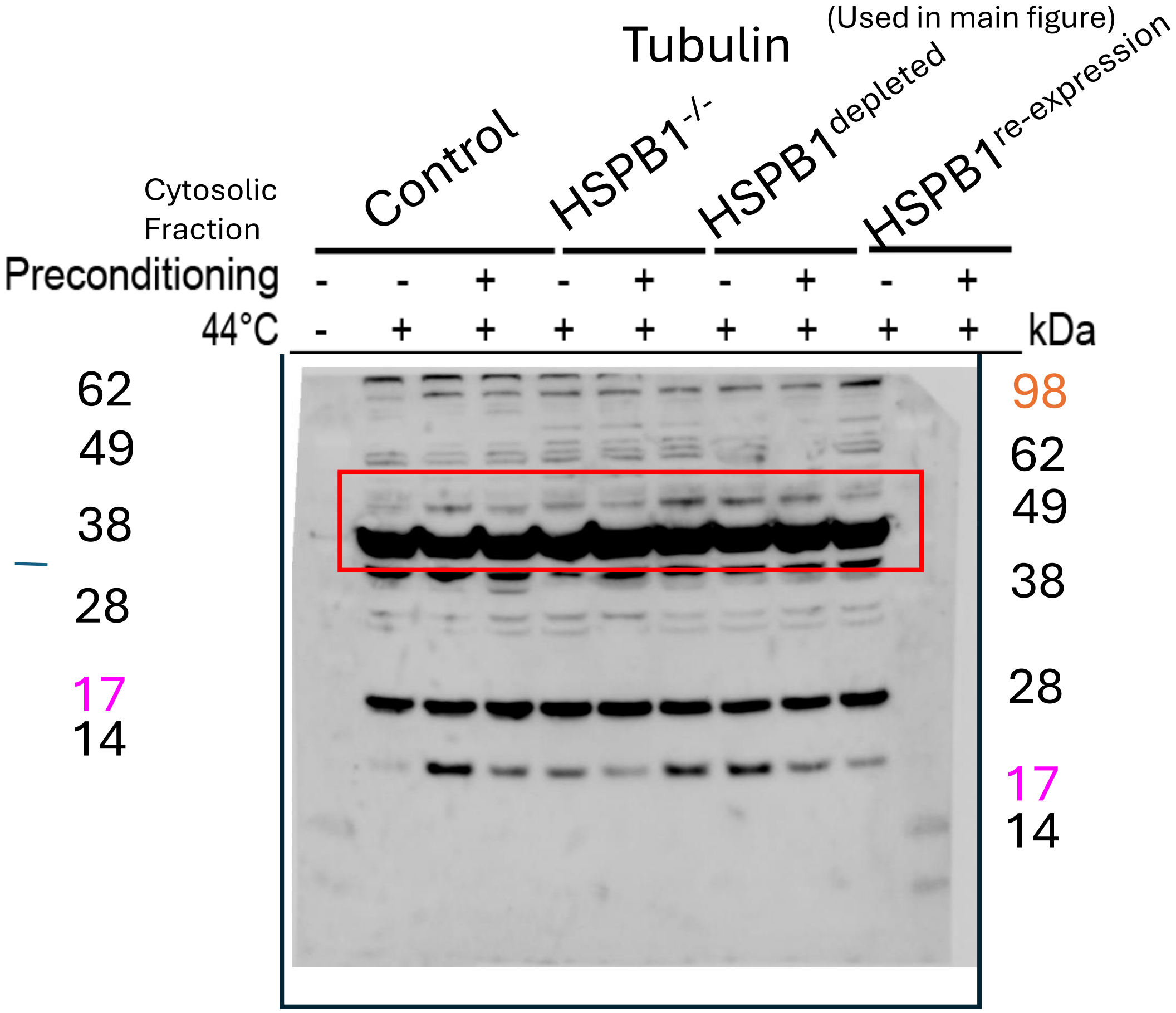

Bis-Tris 4-12% Gel
MES Buffer

Figure 4:
4A (Replicate 2)

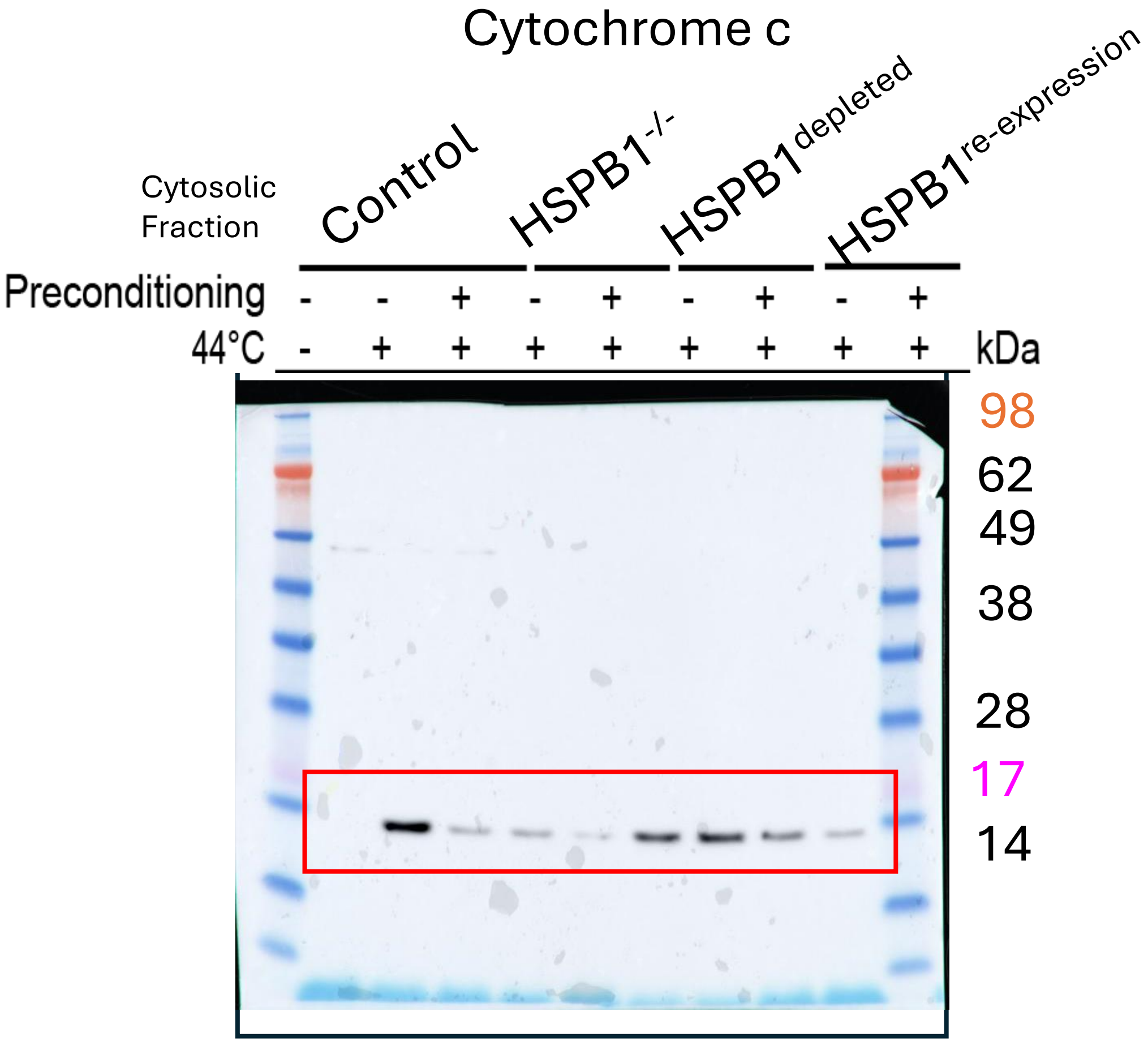

Bis-Tris 4-12% Gel
MES Buffer

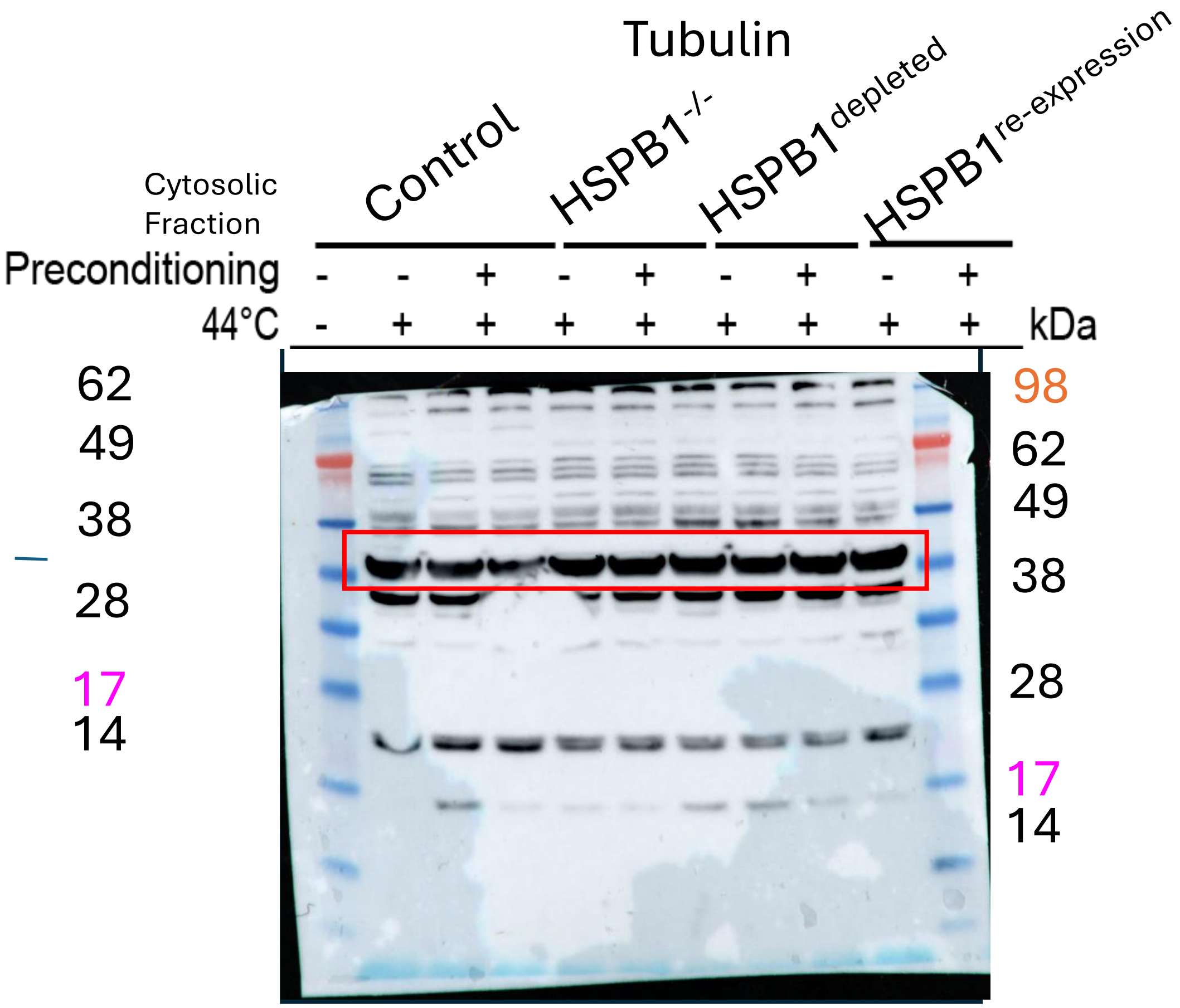

Bis-Tris 4-12% Gel
MES Buffer

Figure 4:
4A (Replicate 3)

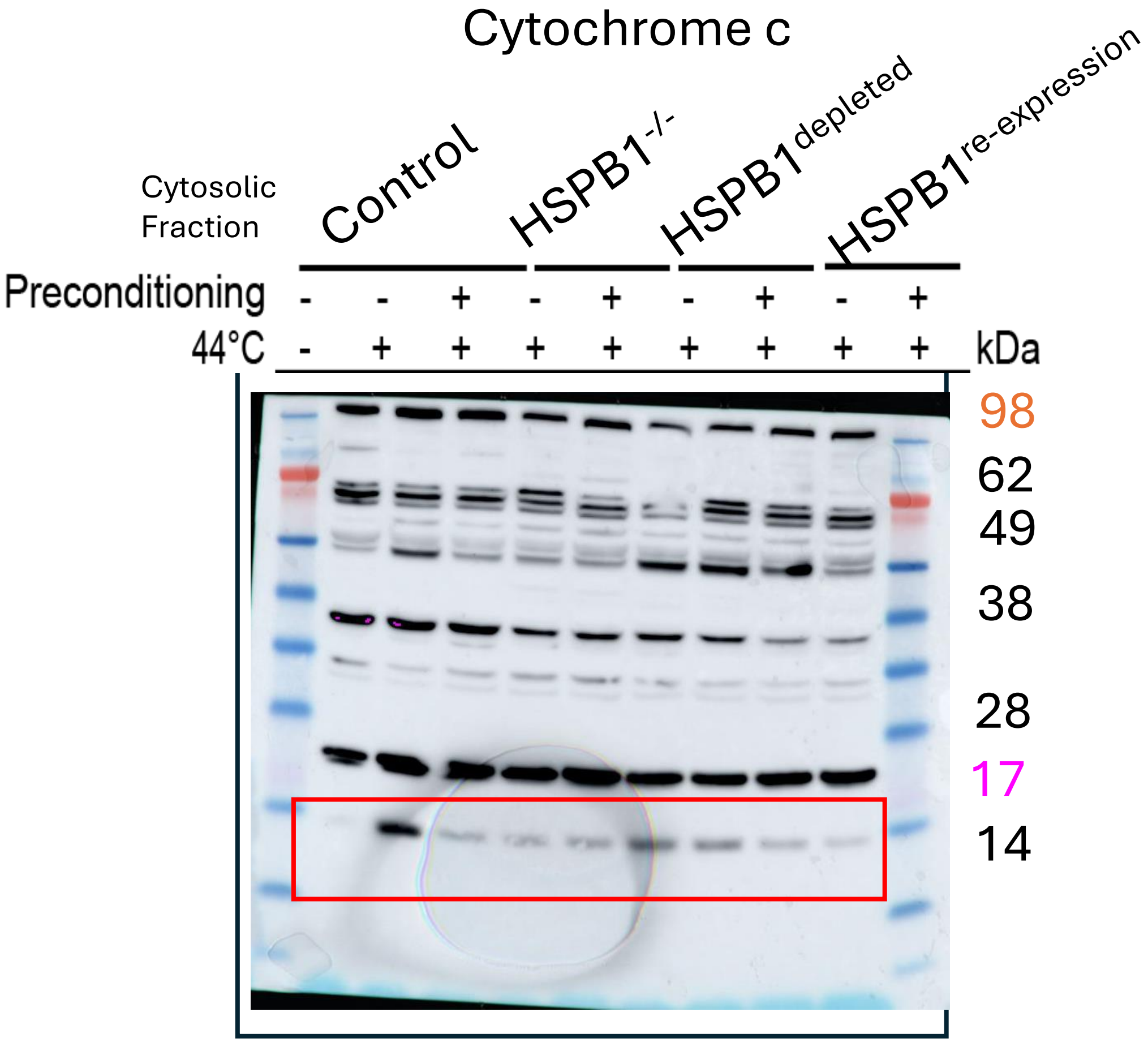

Bis-Tris 4-12% Gel
MES Buffer

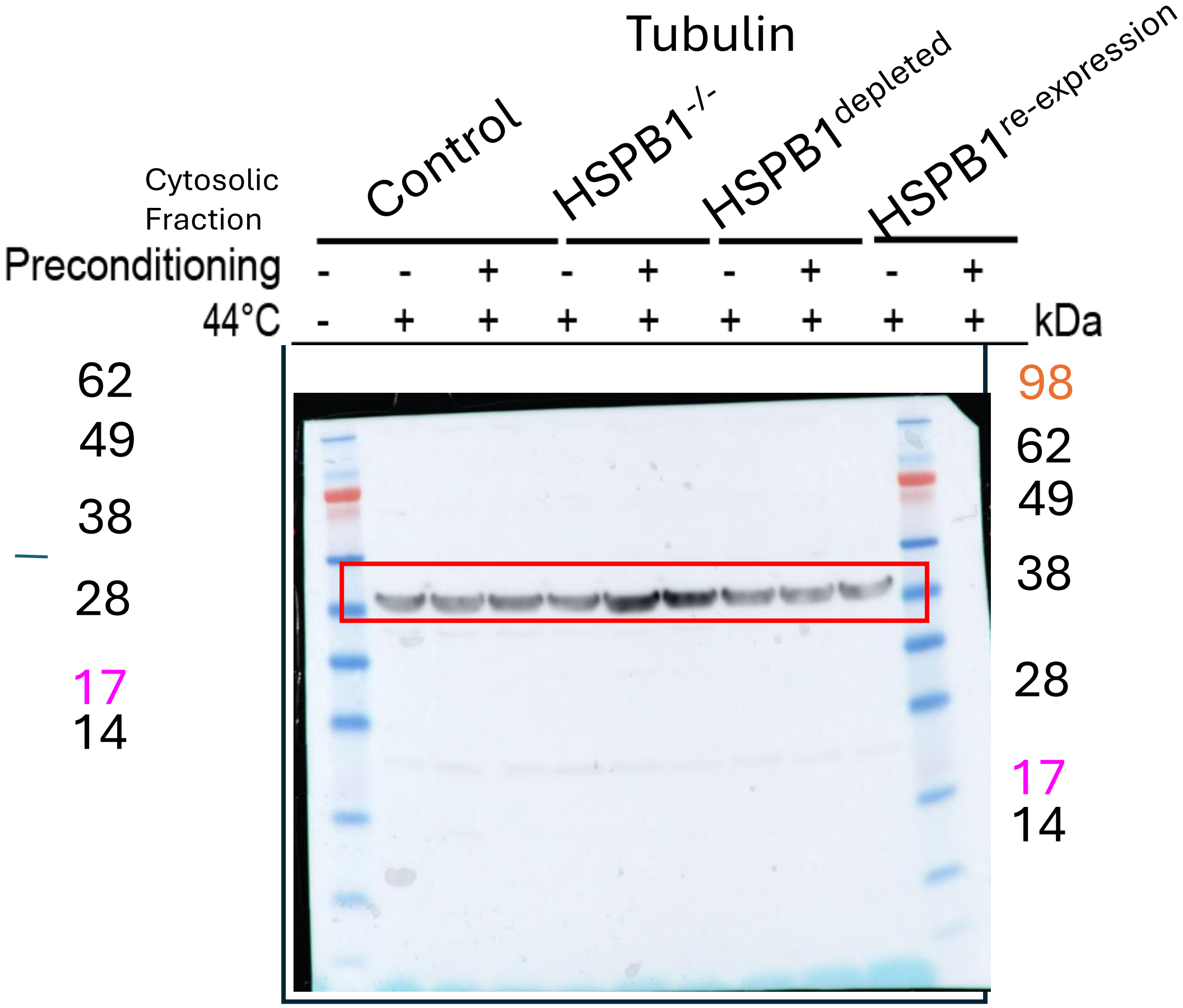

Bis-Tris 4-12% Gel
MES Buffer

Figure 4:
4B (Replicate 1)

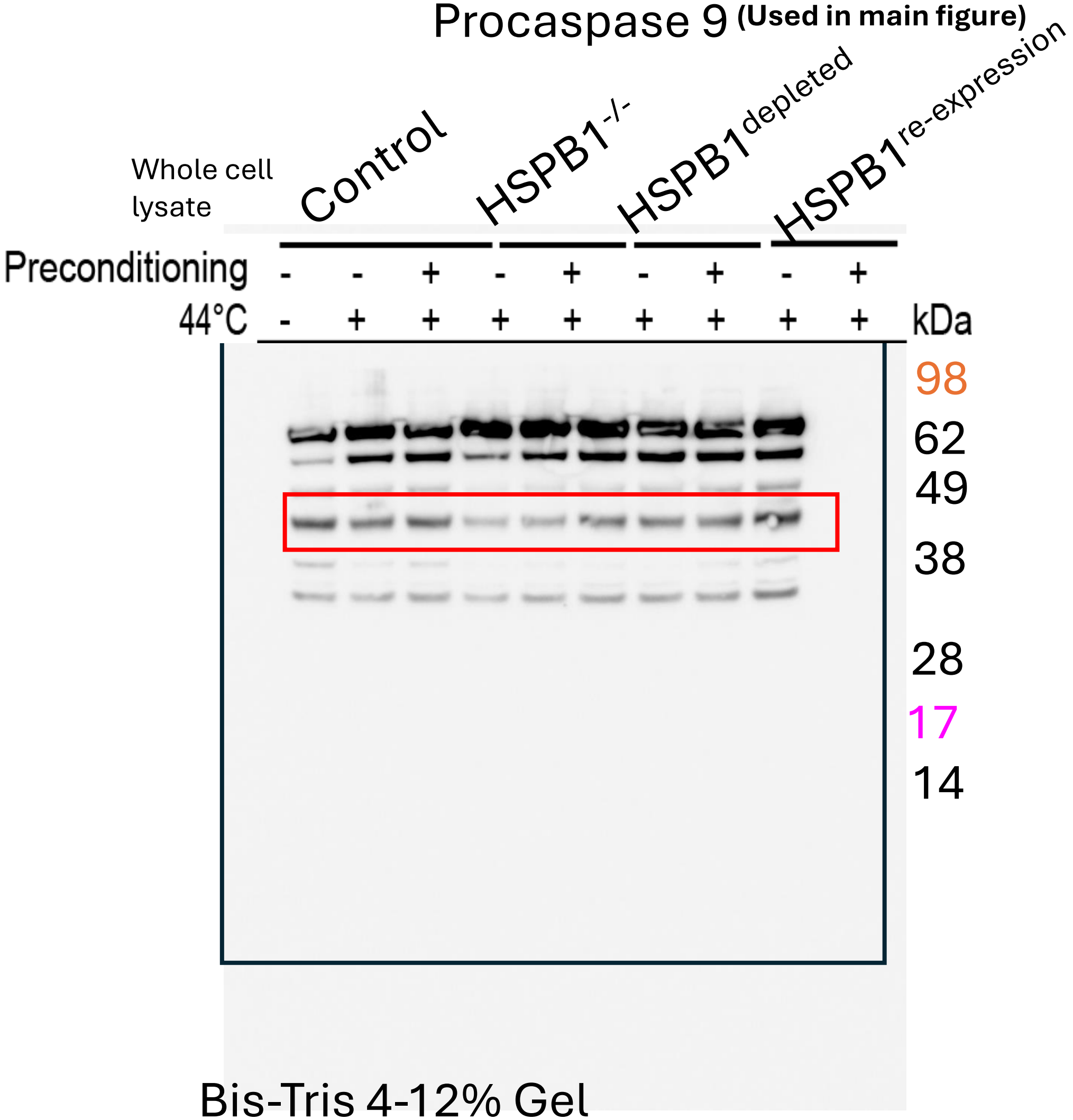

Bis-Tris 4-12% Gel
MES Buffer

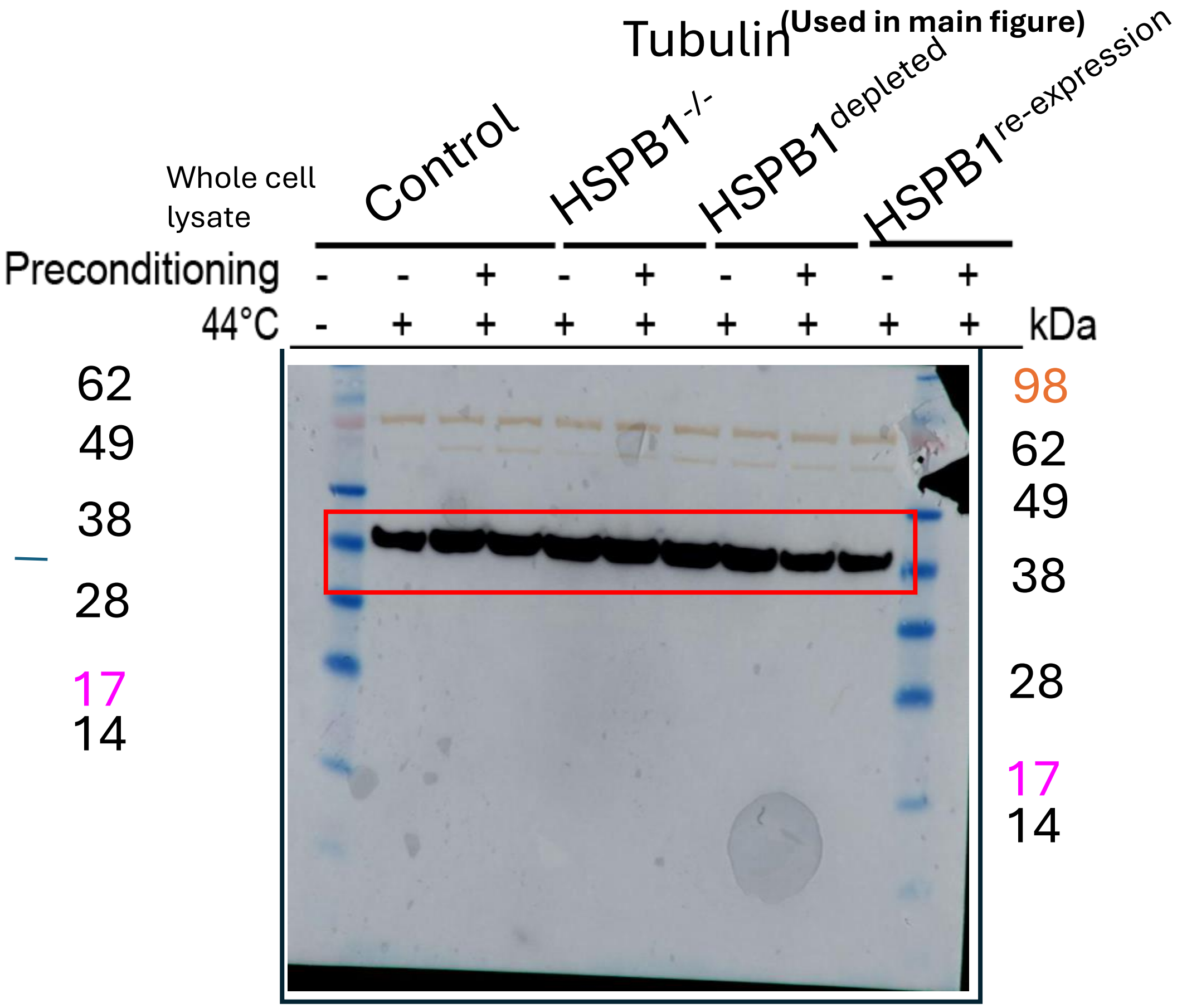

Bis-Tris 4-12% Gel
MES Buffer

Figure 4:
4B (Replicate 2)

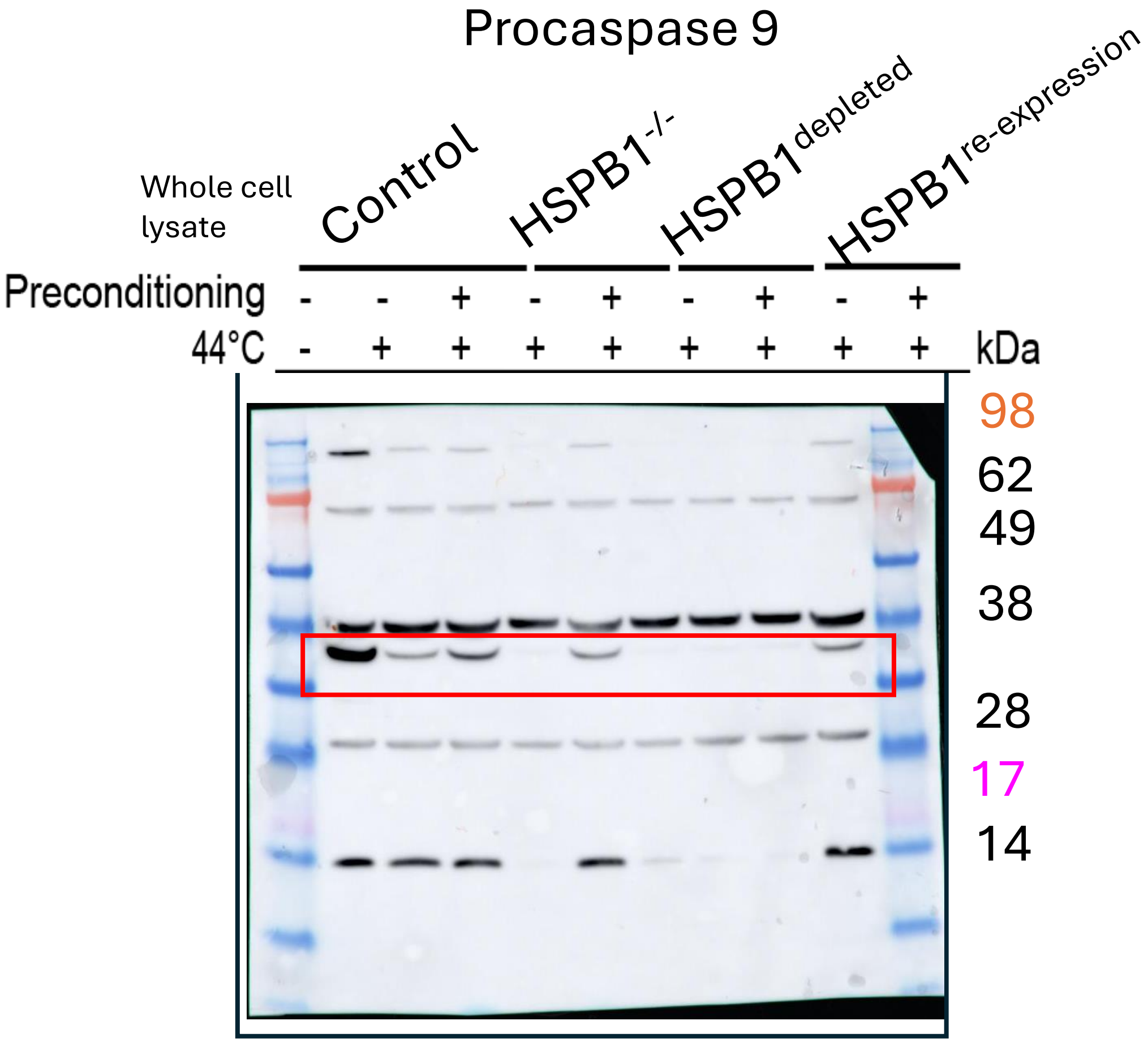

Bis-Tris 4-12% Gel
MES Buffer

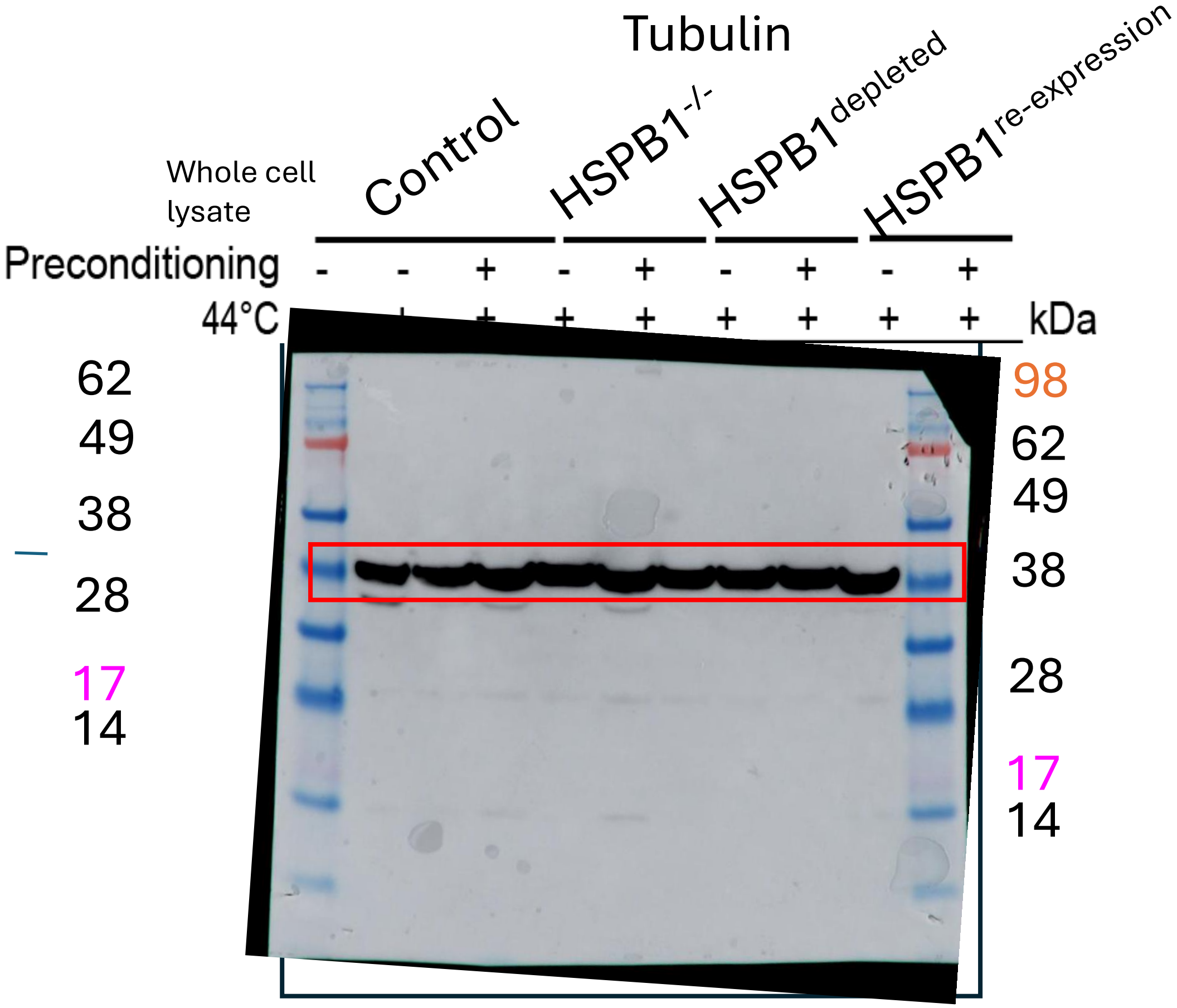

Bis-Tris 4-12% Gel
MES Buffer

Figure 4:
4B (Replicate 3)

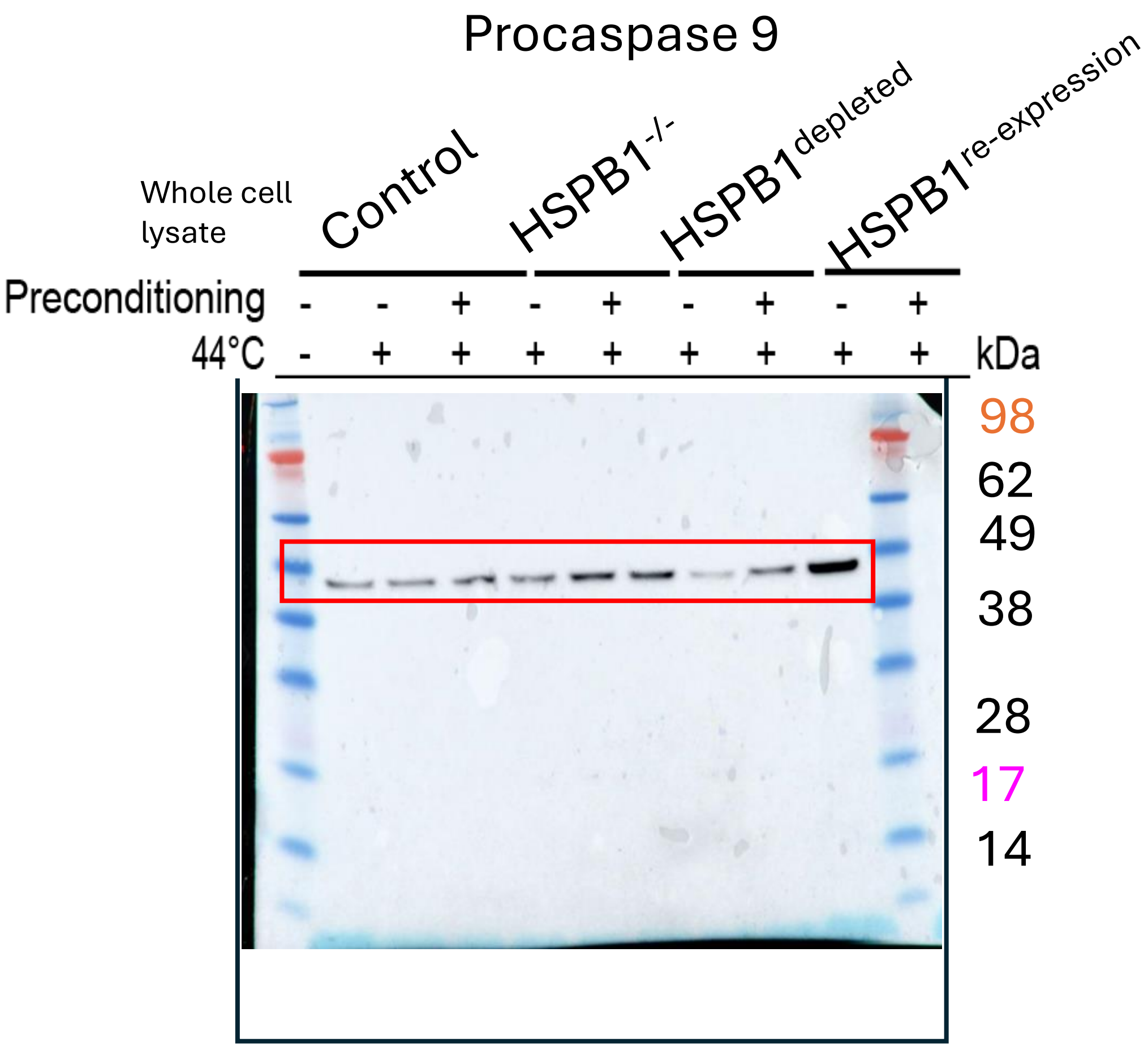

Bis-Tris 4-12% Gel
MES Buffer

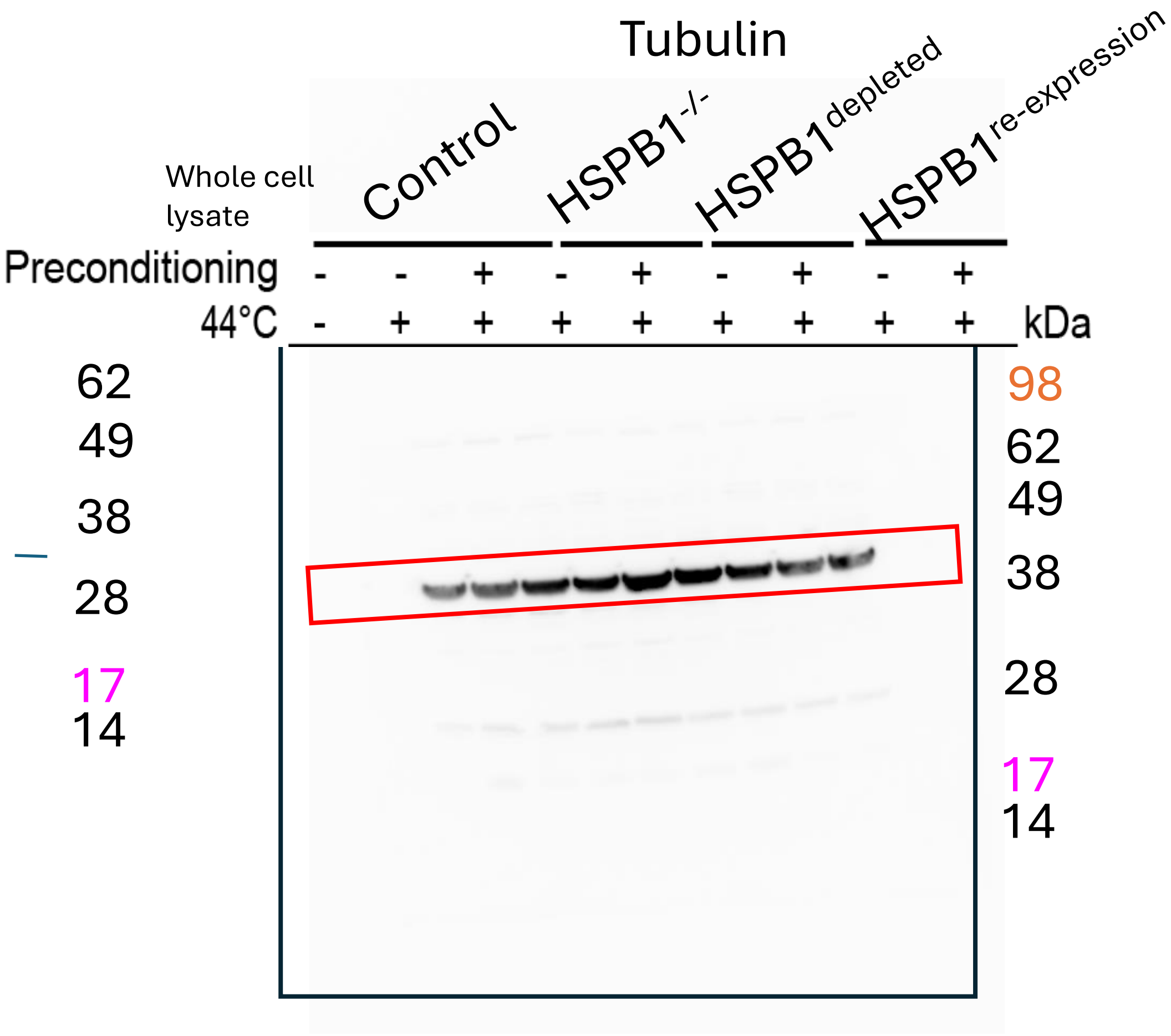

Bis-Tris 4-12% Gel
MES Buffer

Figure 4:
4C (Replicate 1)

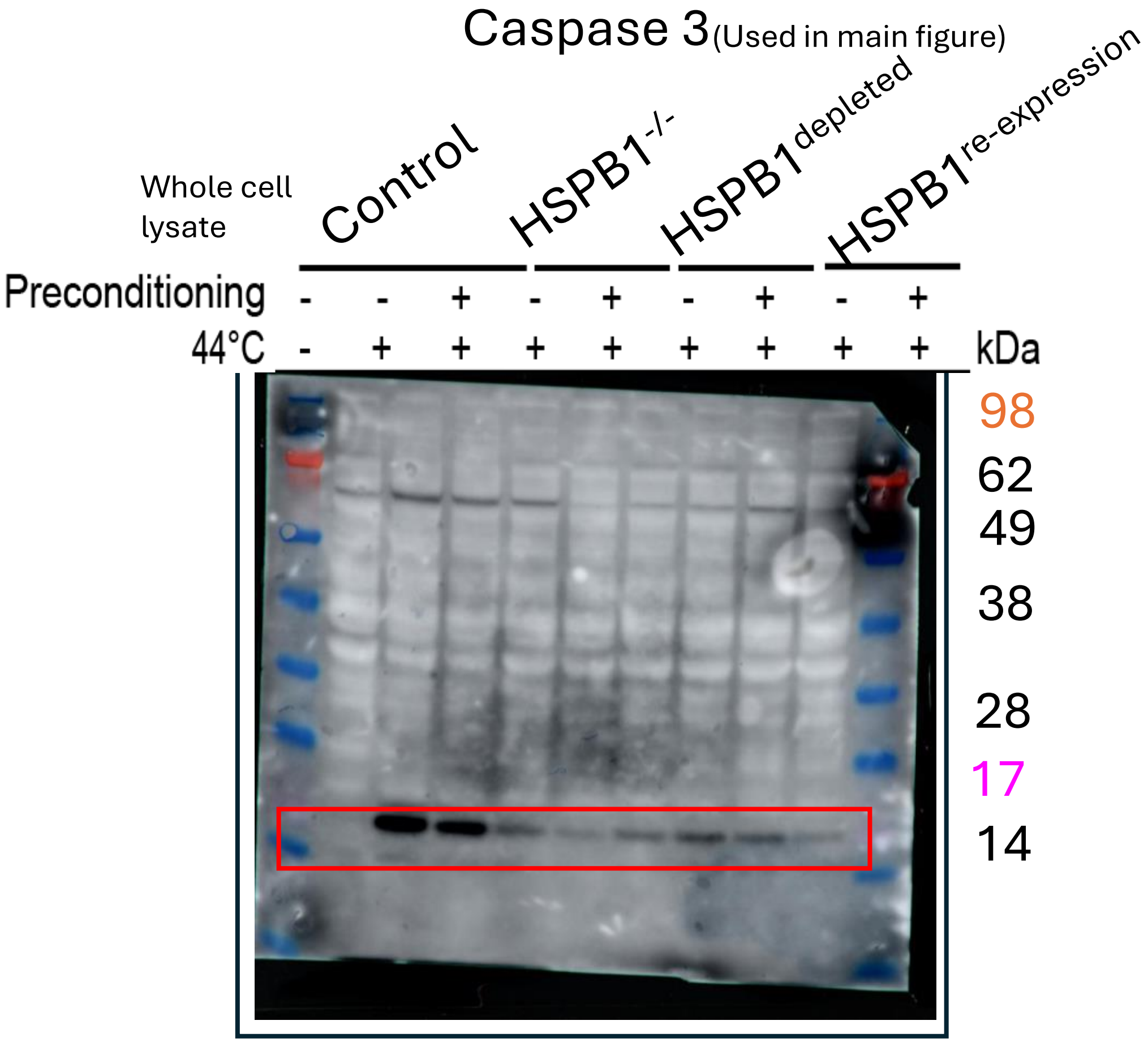

Bis-Tris 4-12% Gel
MES Buffer

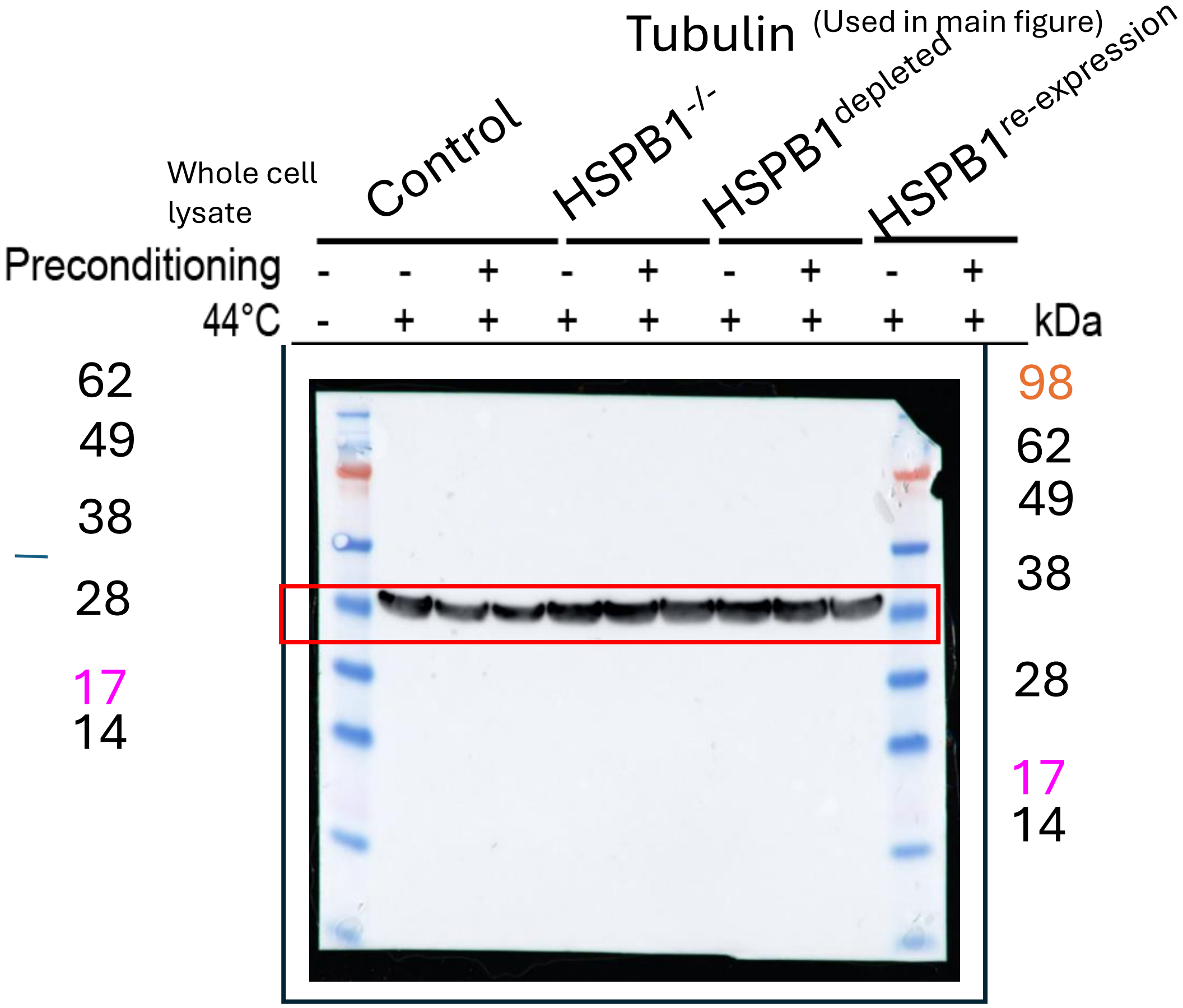

Bis-Tris 4-12% Gel
MES Buffer

Figure 4:
4C (Replicate 2)

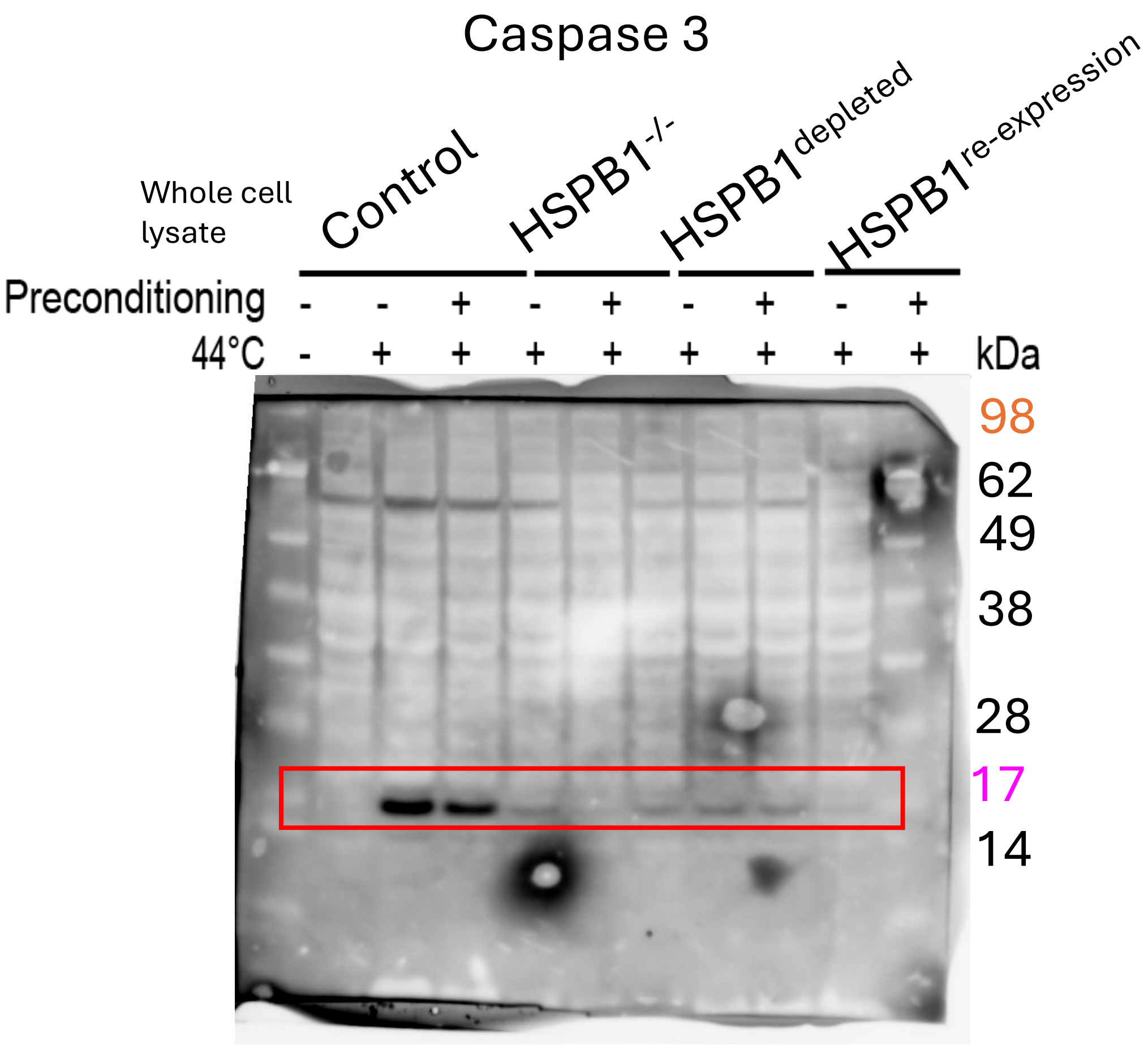

Bis-Tris 4-12% Gel
MES Buffer

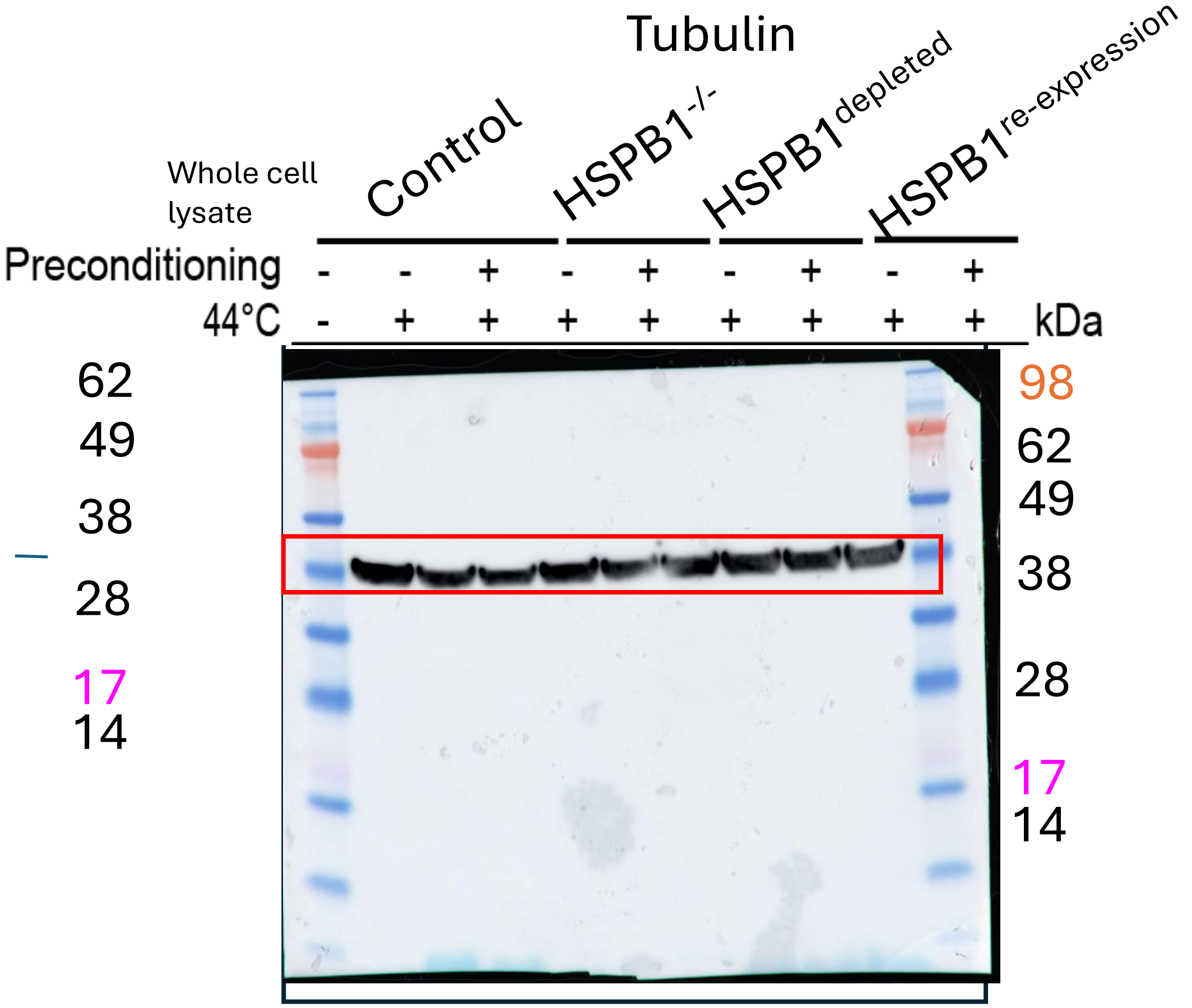

Bis-Tris 4-12% Gel
MES Buffer

Figure 4:
4C (Replicate 3)

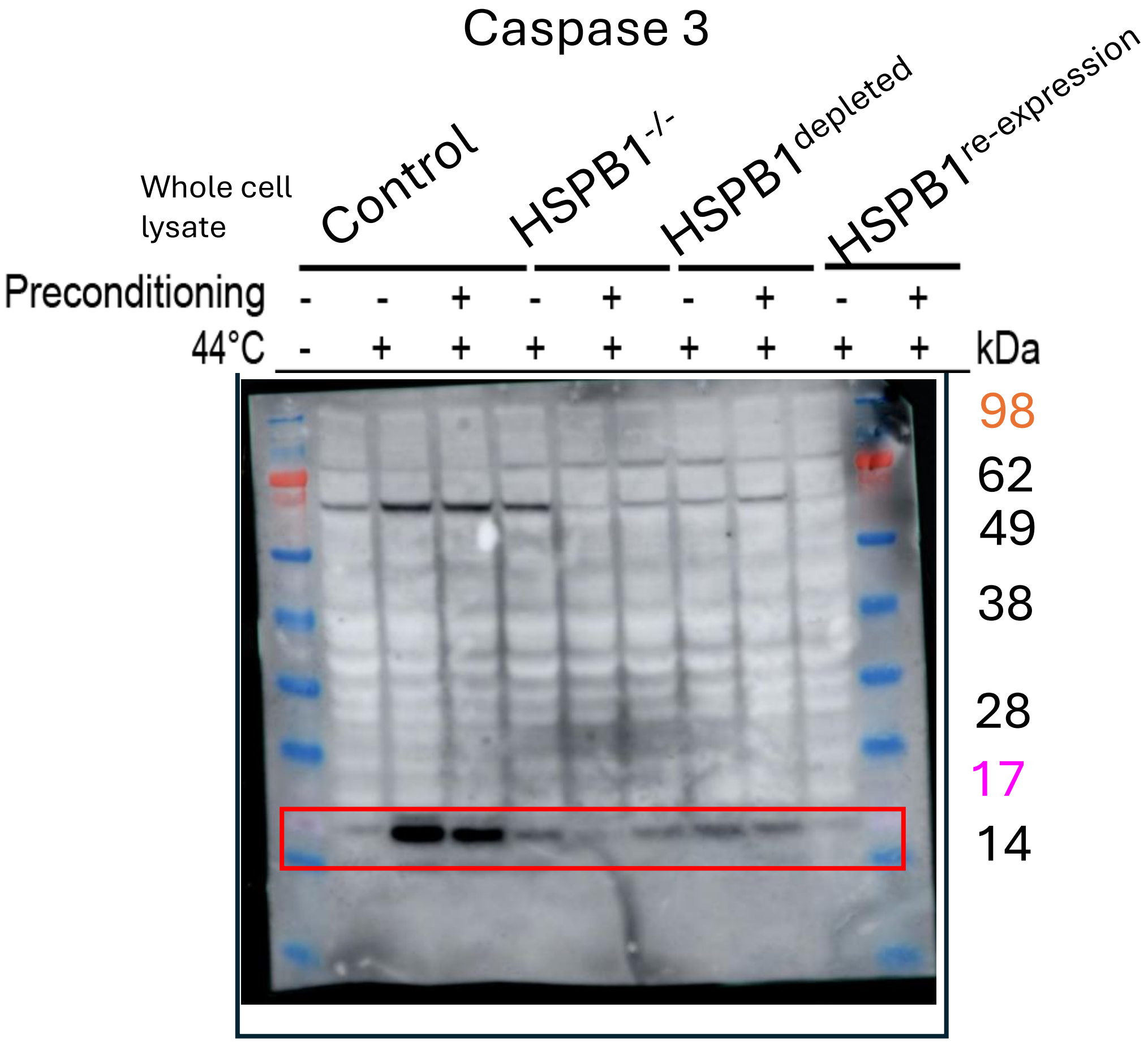

Bis-Tris 4-12% Gel
MES Buffer

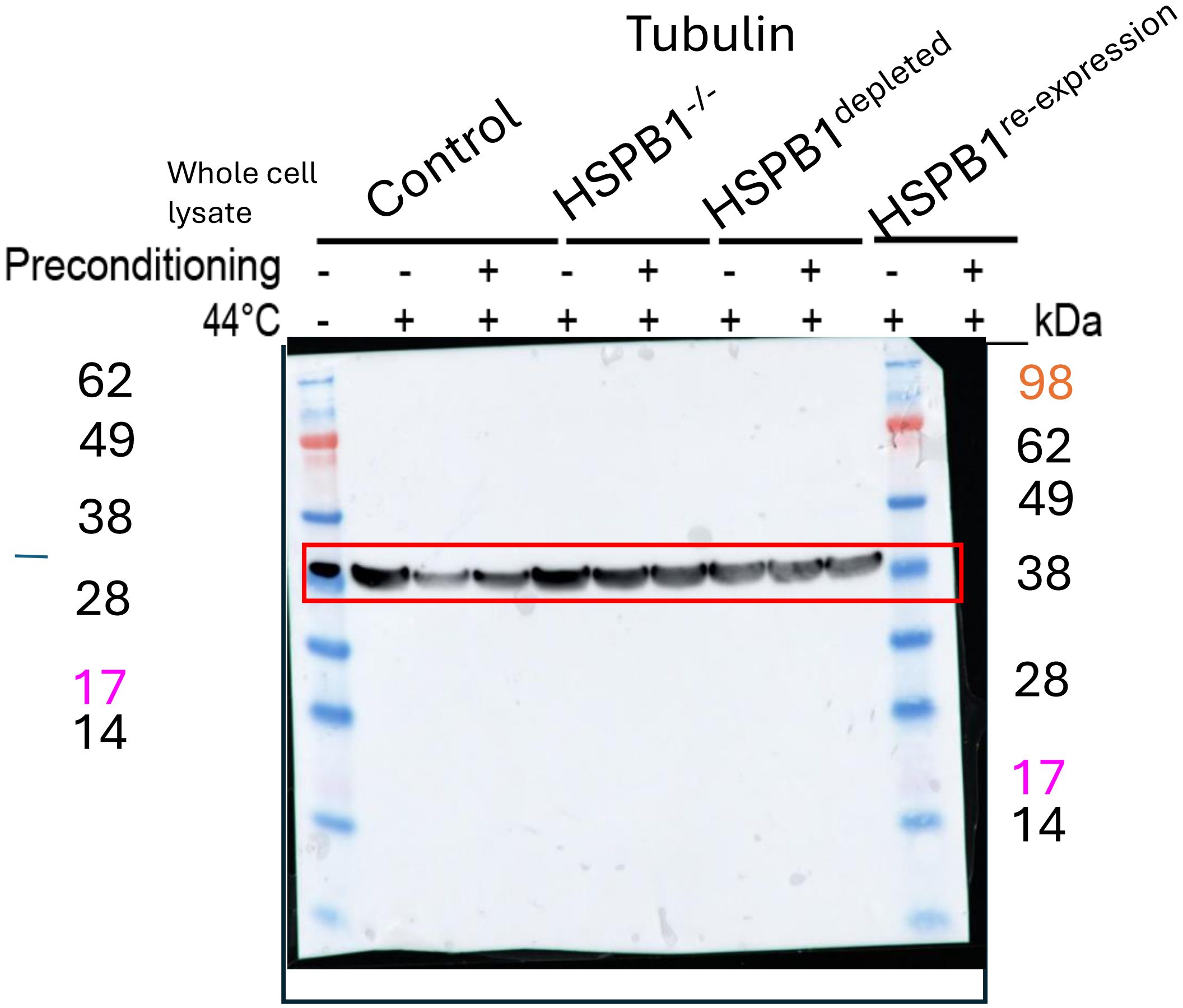

Bis-Tris 4-12% Gel
MES Buffer

Figure 4:
4D (Replicate 1)

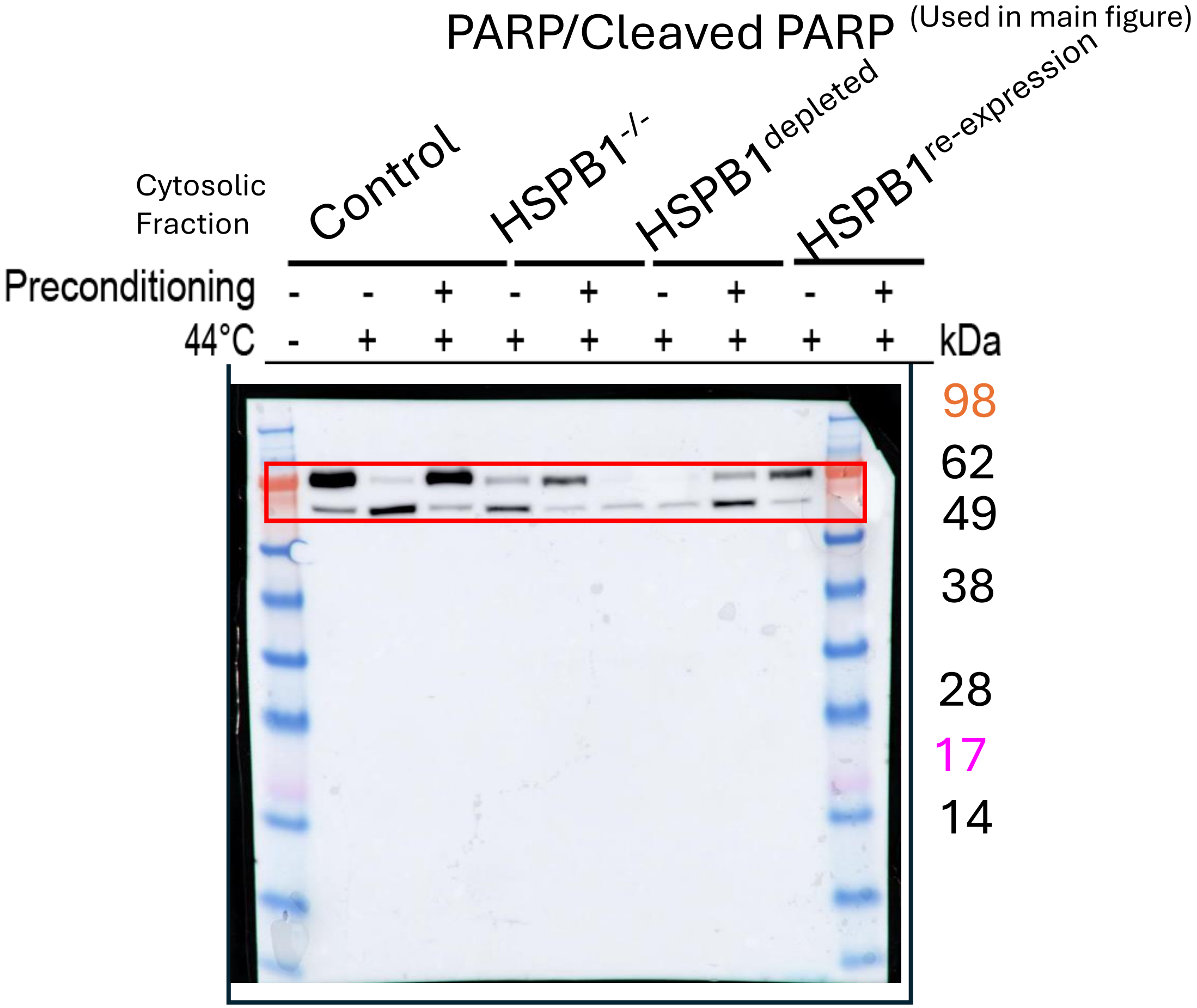

Bis-Tris 4-12% Gel
MES Buffer

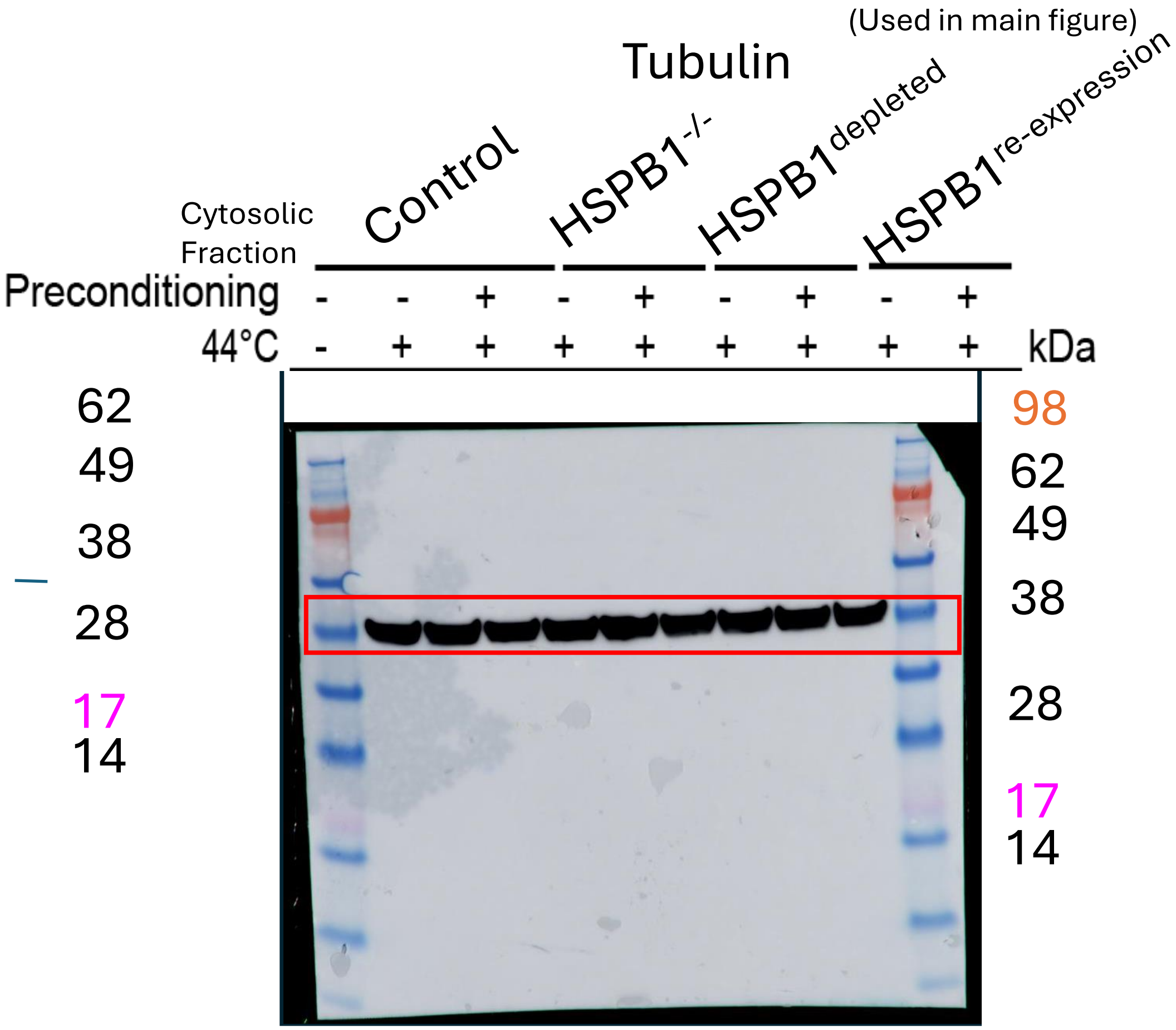

Bis-Tris 4-12% Gel
MES Buffer

Figure 4:
4D (Replicate 2)

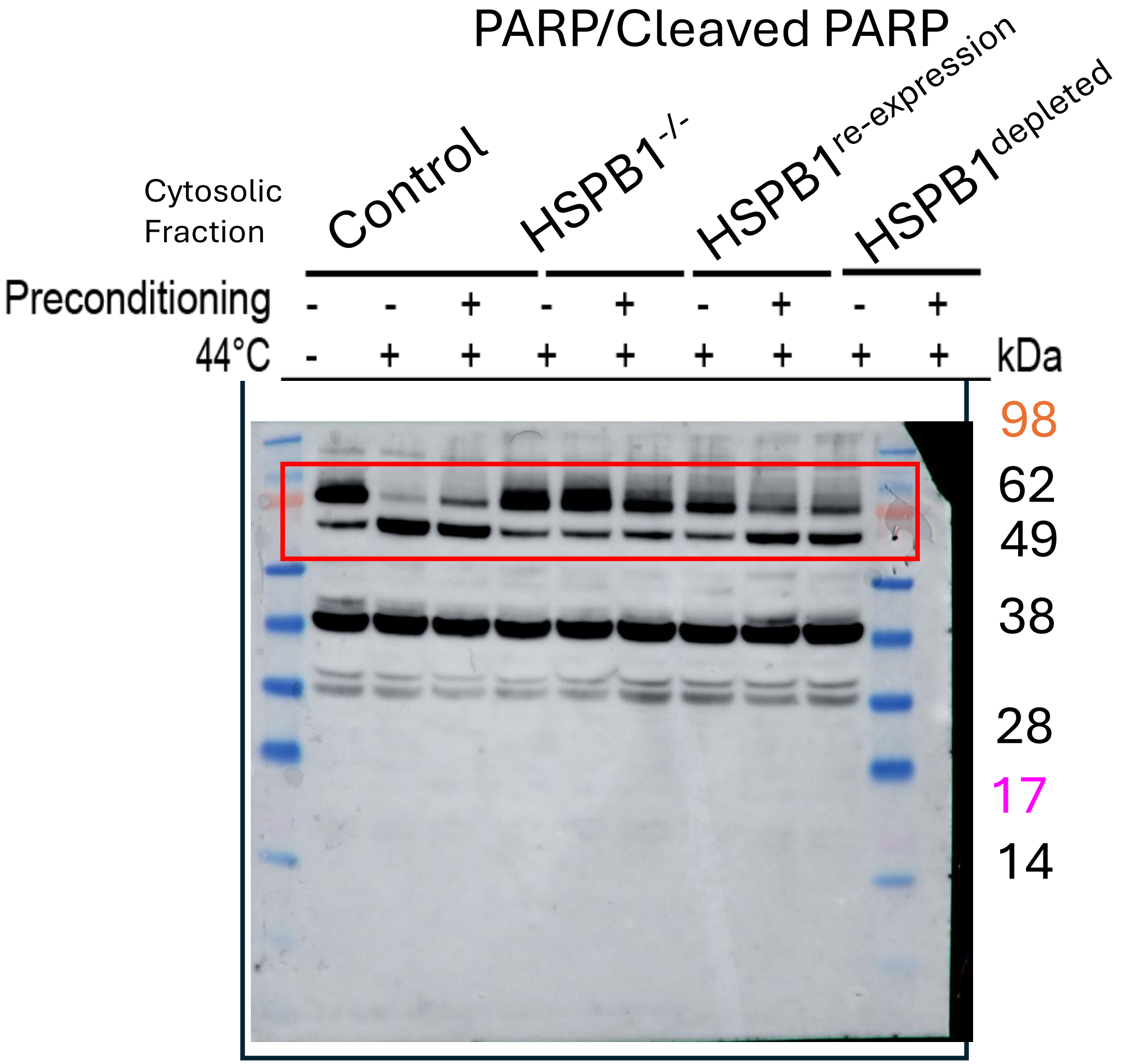

Bis-Tris 4-12% Gel
MES Buffer

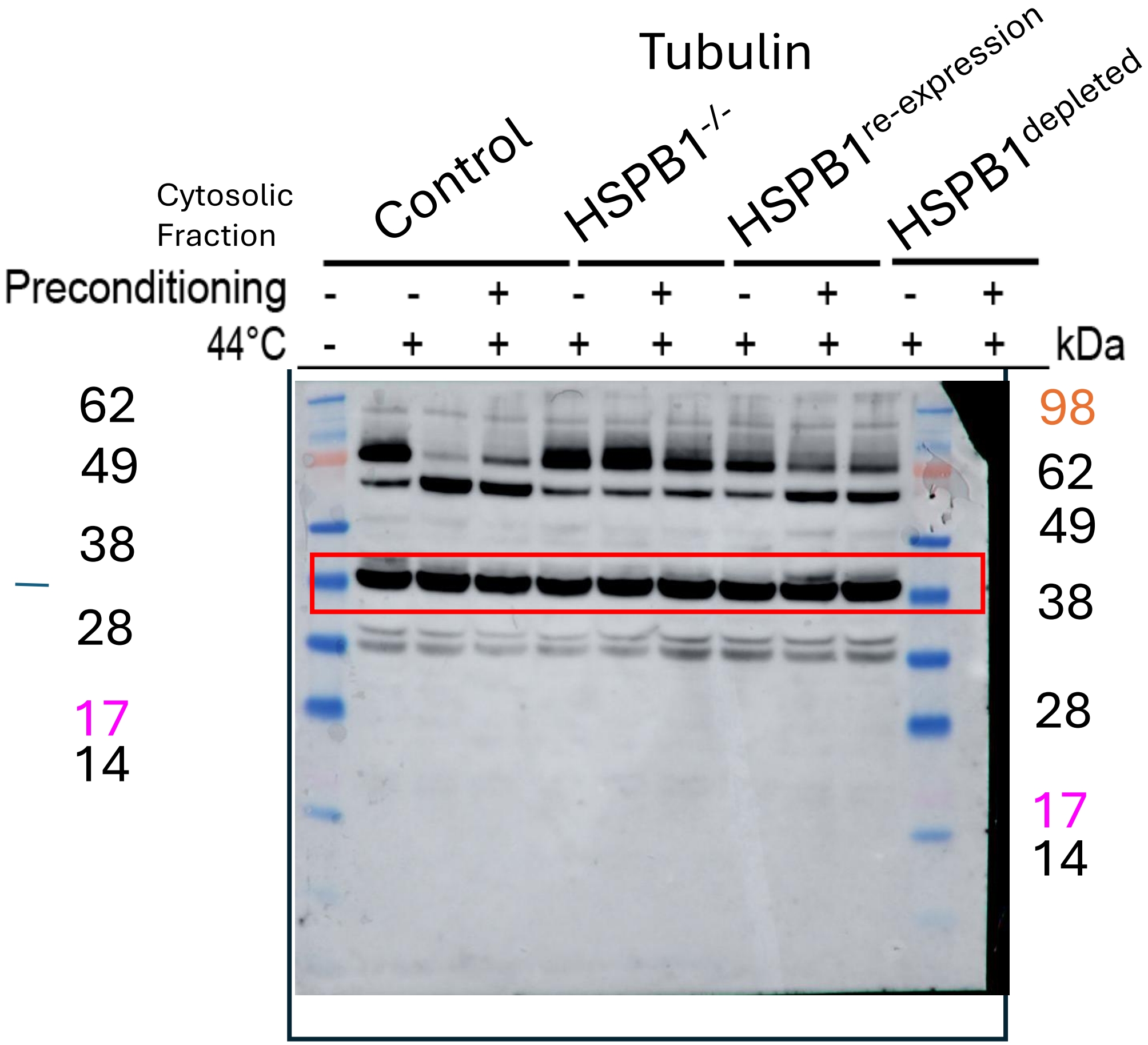

Bis-Tris 4-12% Gel
MES Buffer

Figure 4:
4D (Replicate 3)

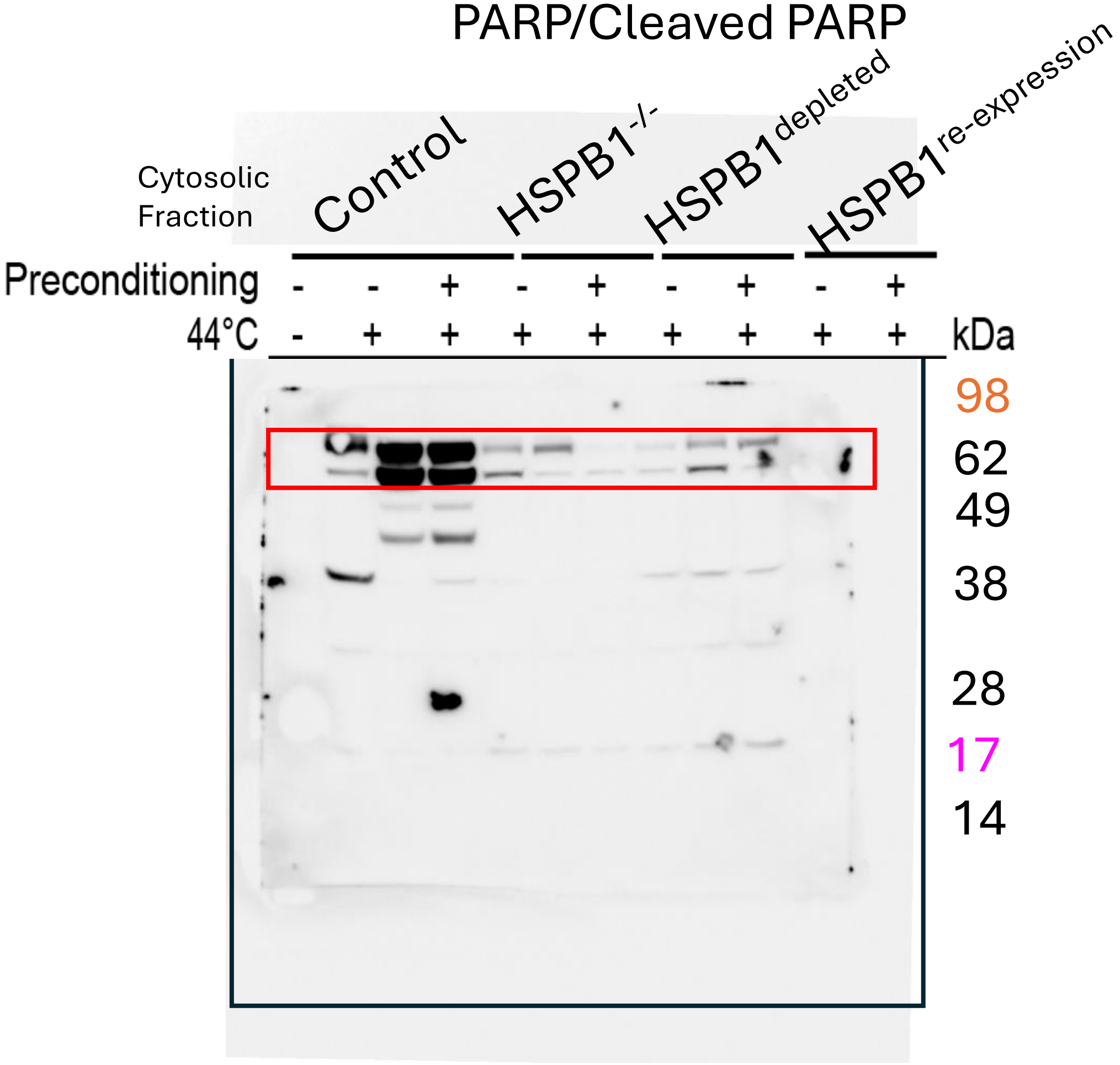

Bis-Tris 4-12% Gel
MES Buffer

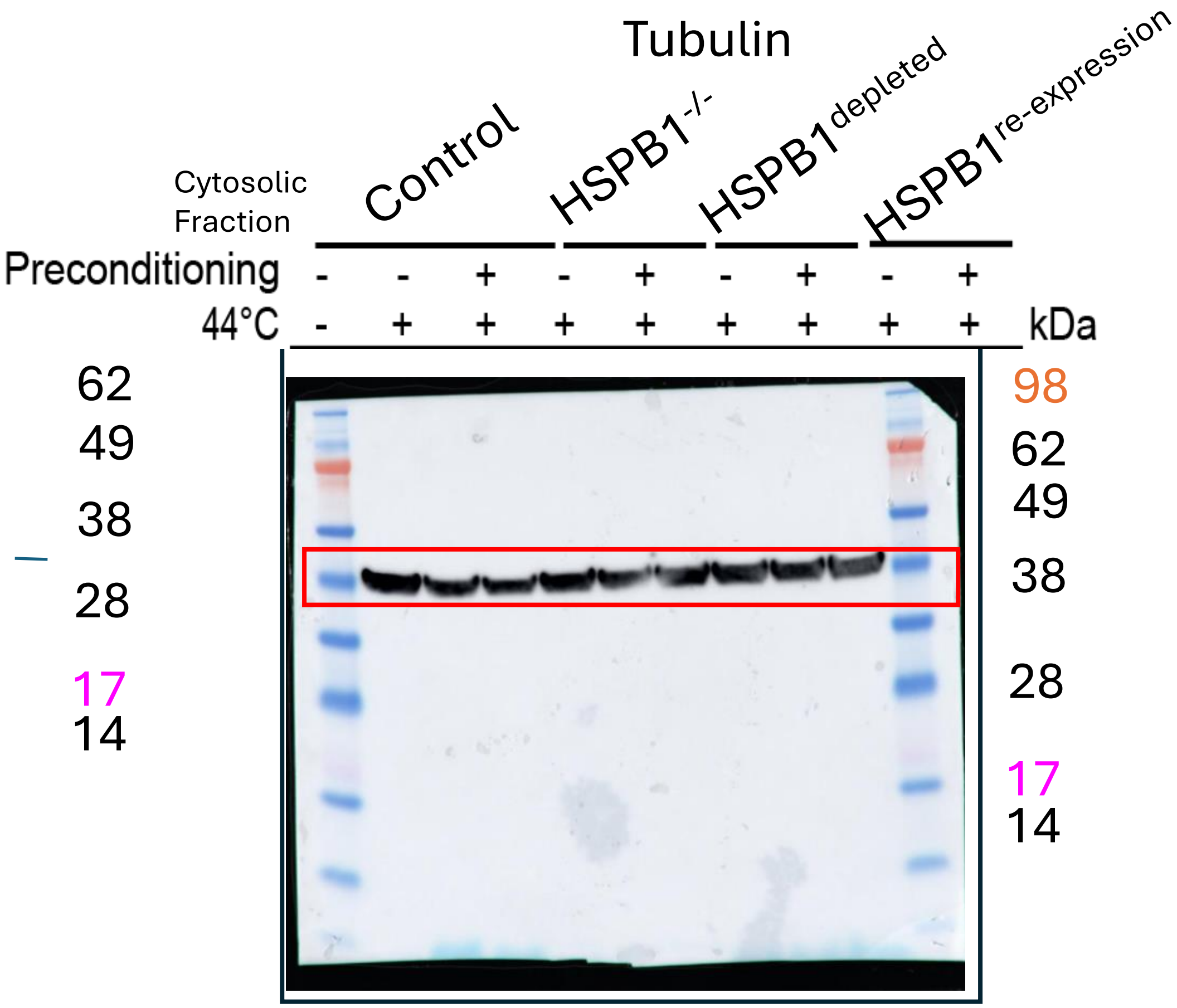

Bis-Tris 4-12% Gel
MES Buffer

Figure 4:
4D (Replicate 4)

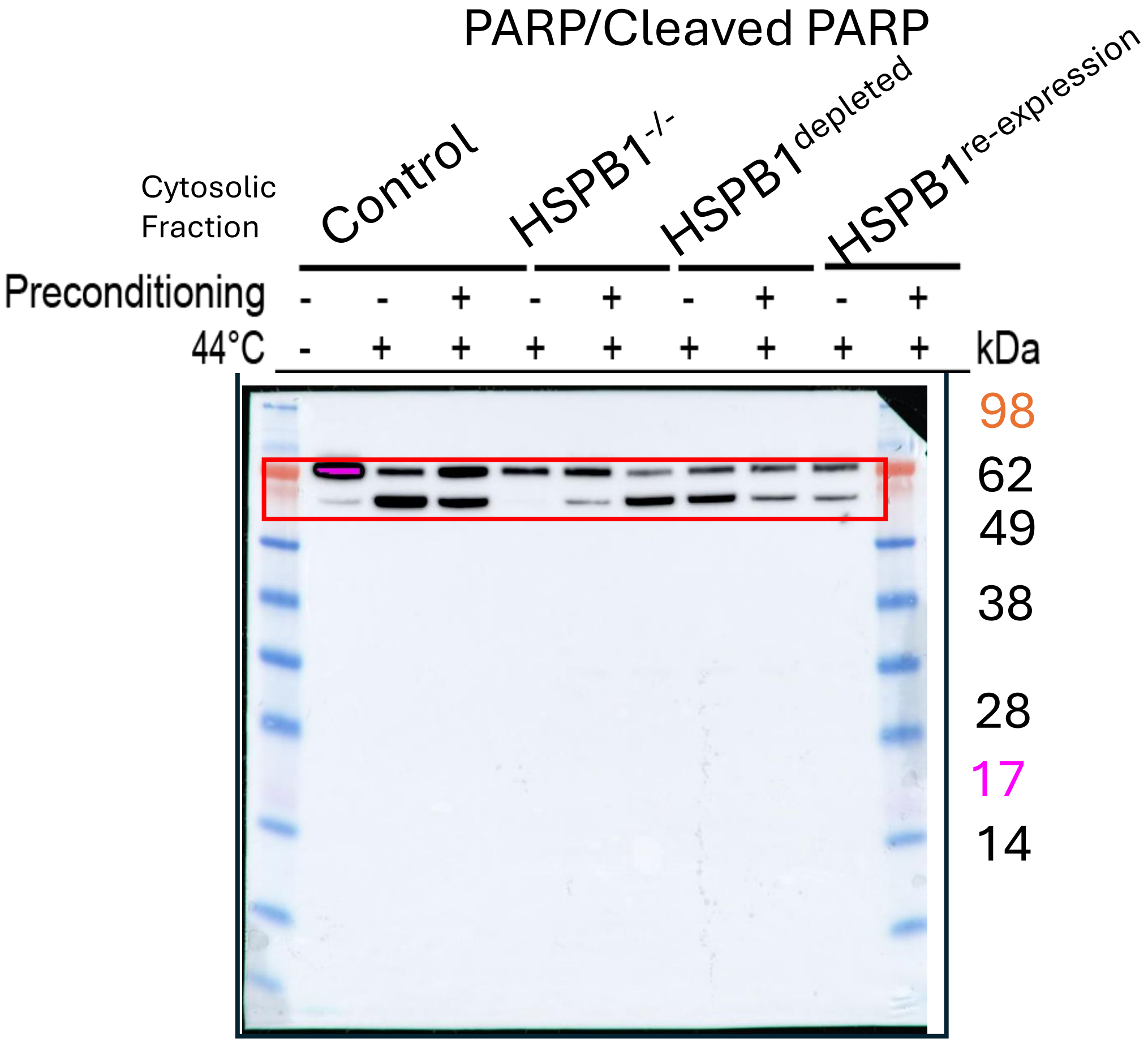

Bis-Tris 4-12% Gel
MES Buffer

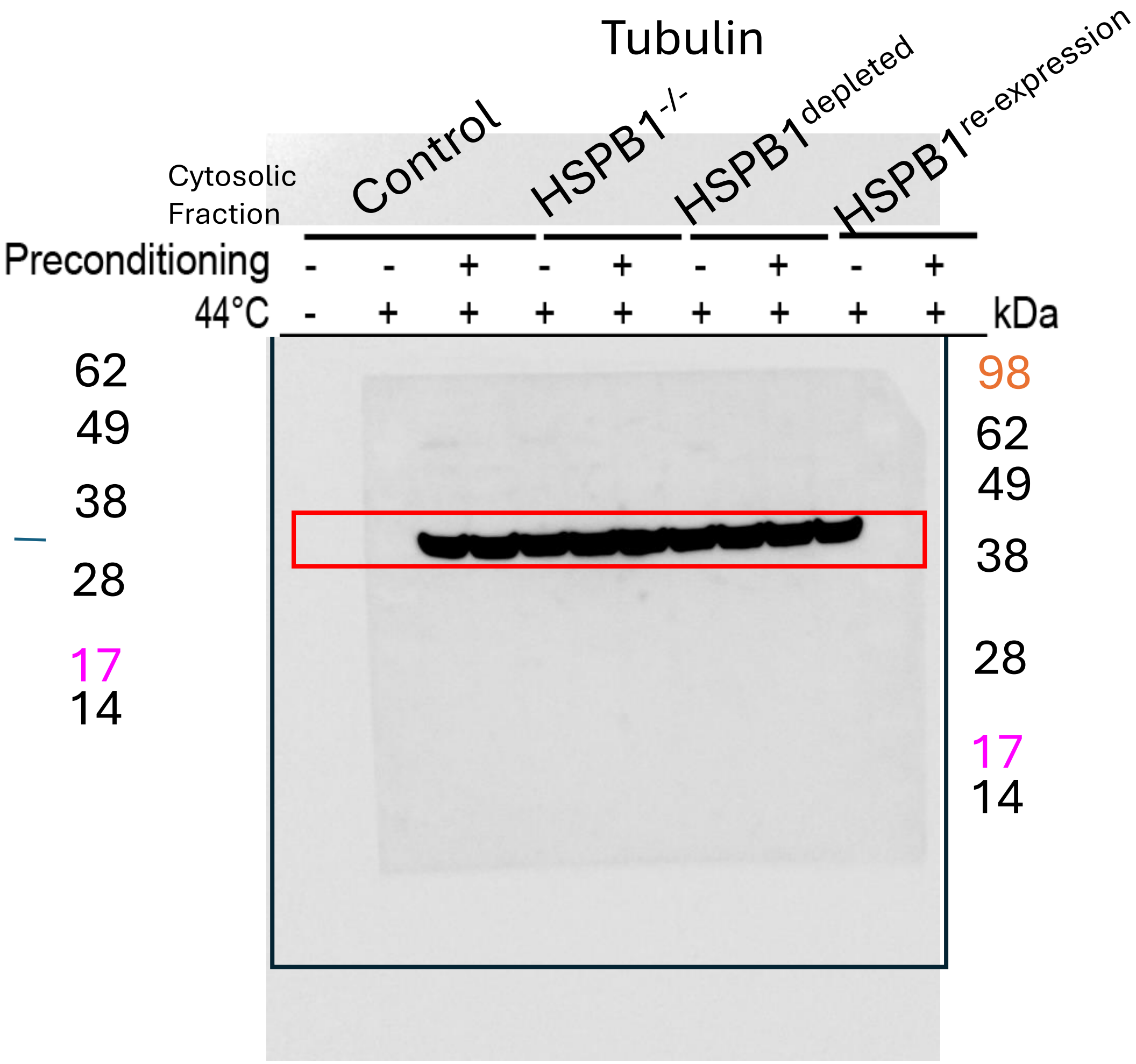

Bis-Tris 4-12% Gel
MES Buffer

Figure 5:

5A (Replicate 1)

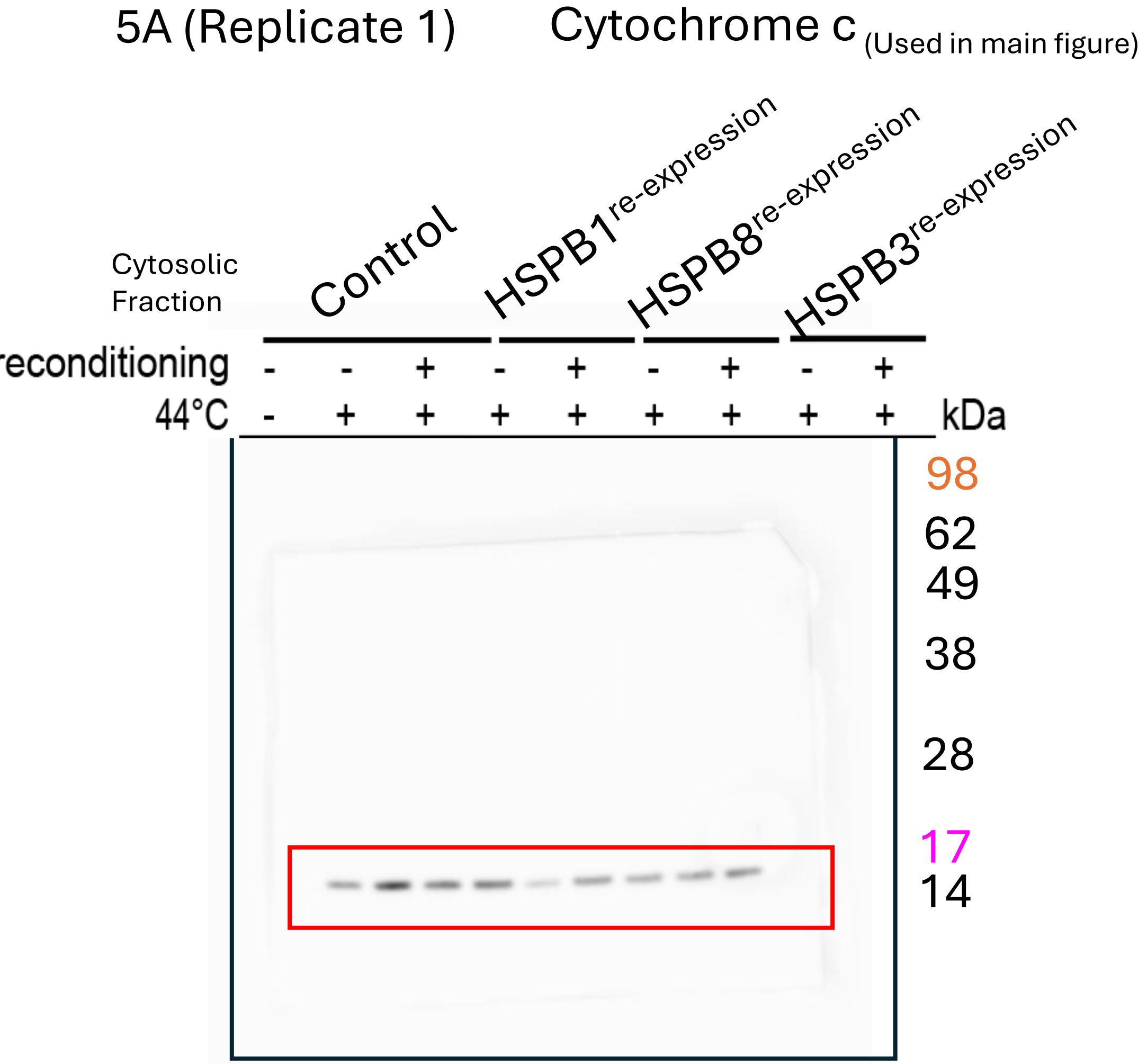

Bis-Tris 4-12% Gel
MES Buffer

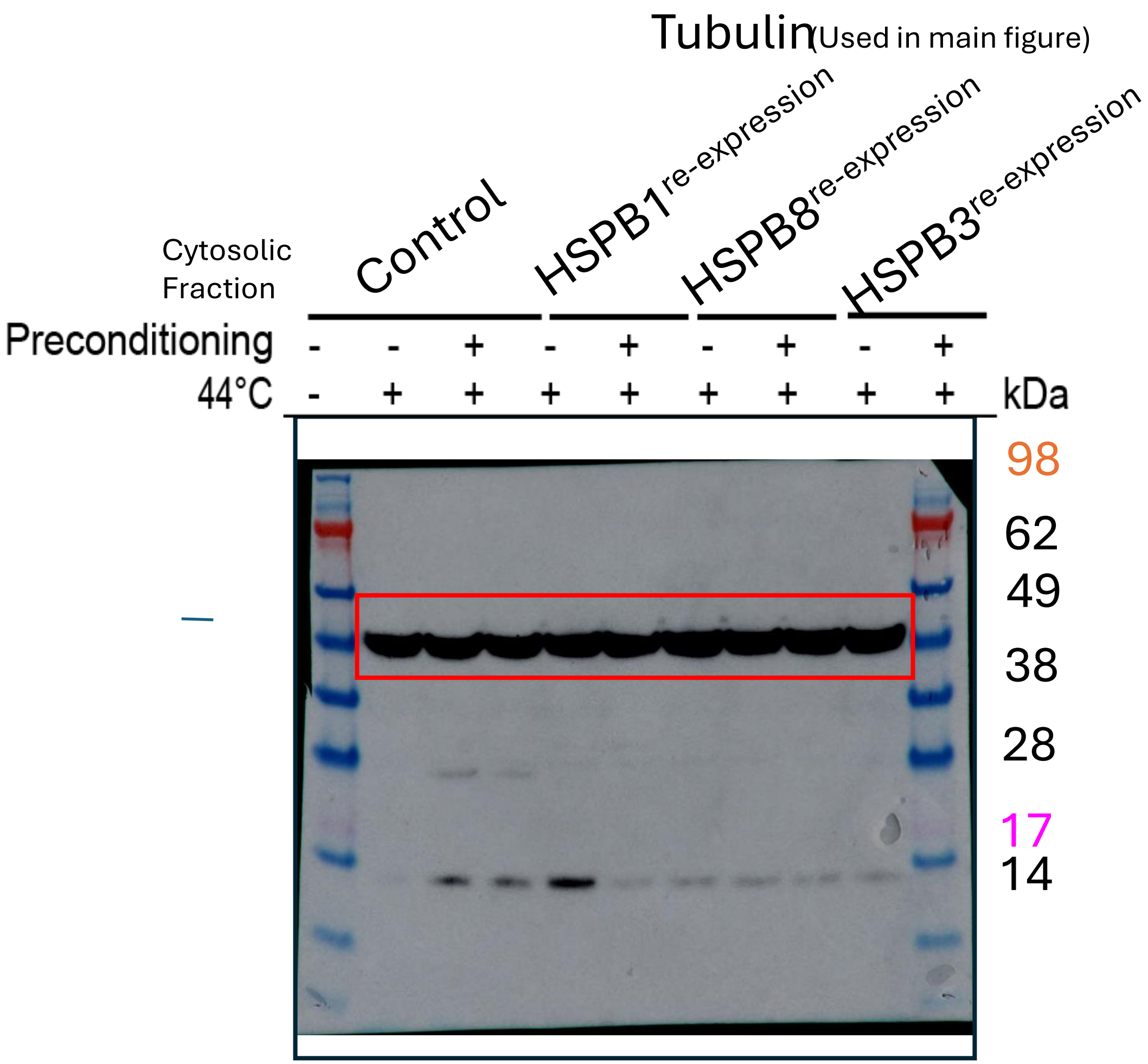

Bis-Tris 4-12% Gel
MES Buffer

Figure 5:
5A (Replicate 2)

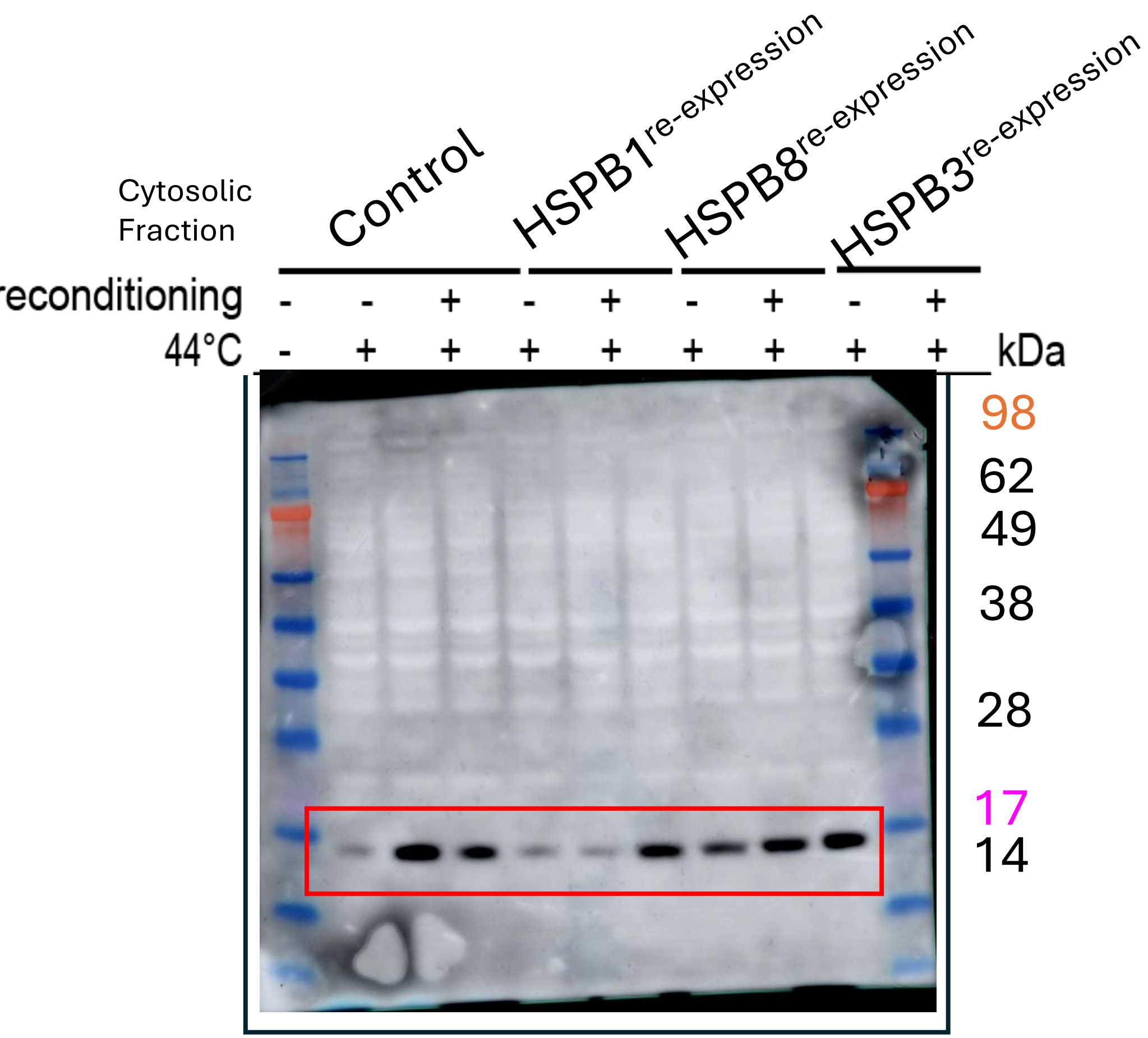

Bis-Tris 4-12% Gel
MES Buffer

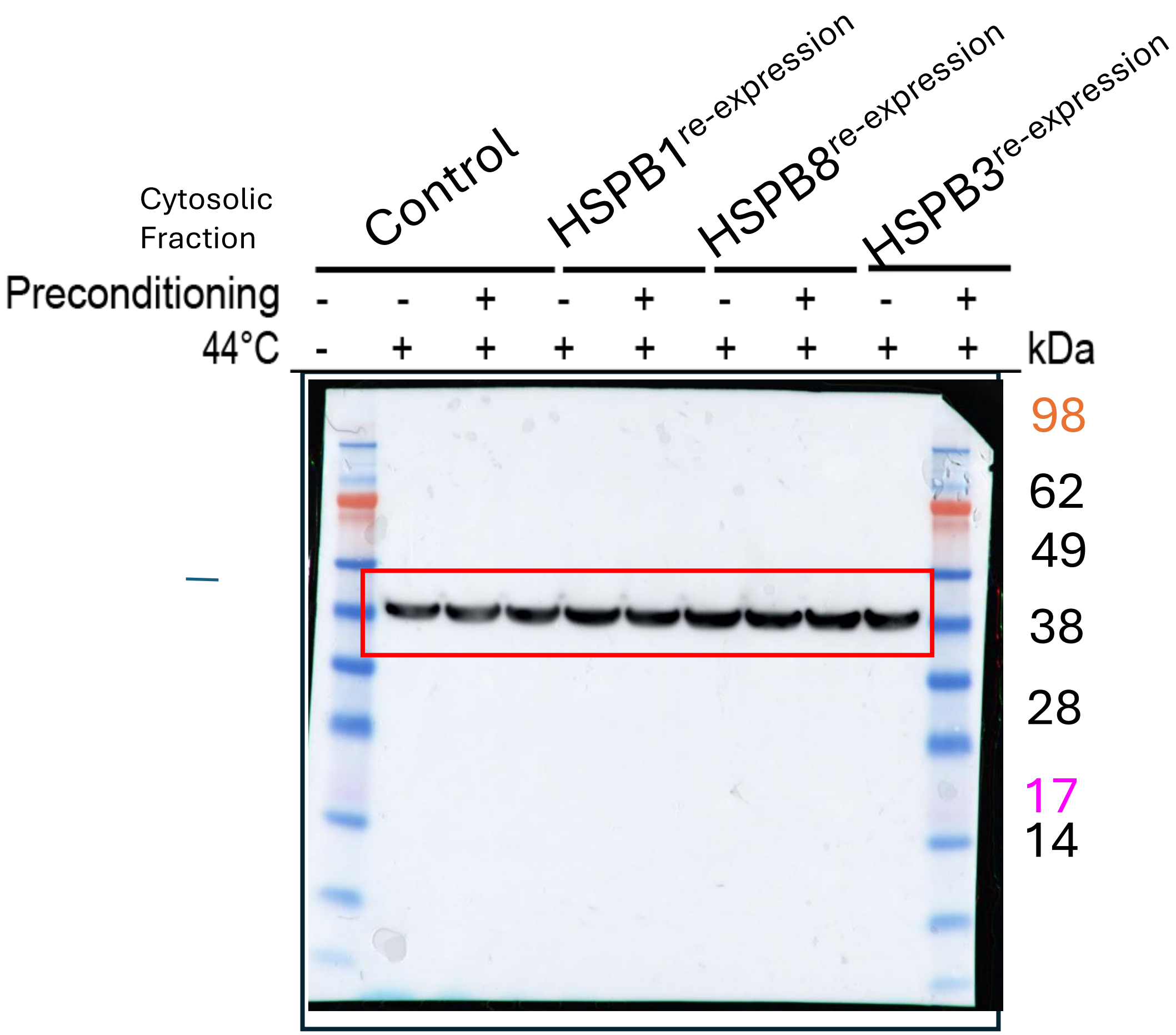

Bis-Tris 4-12% Gel
MES Buffer

Figure 5:
5A (Replicate 3)

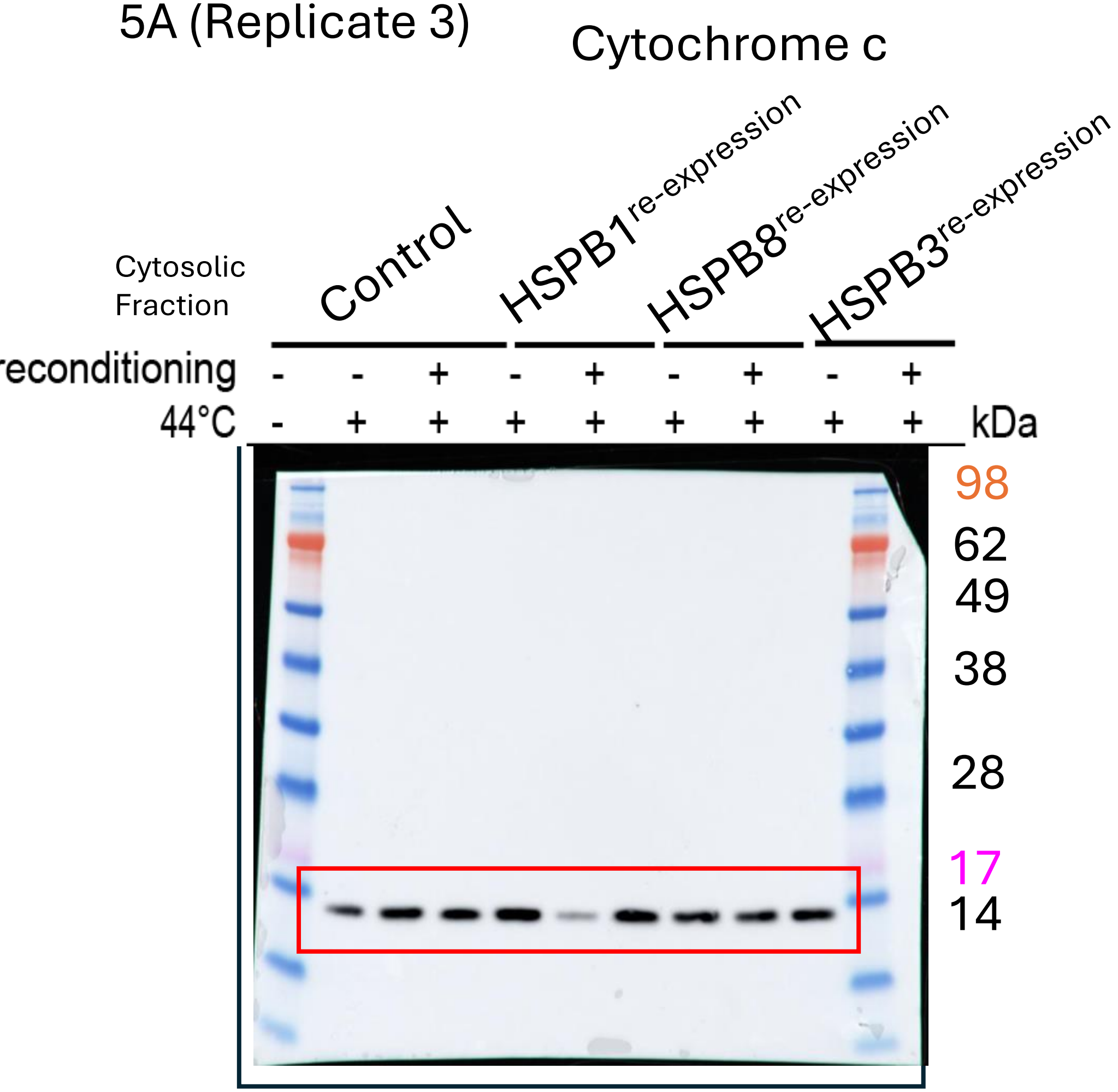

Bis-Tris 4-12% Gel
MES Buffer

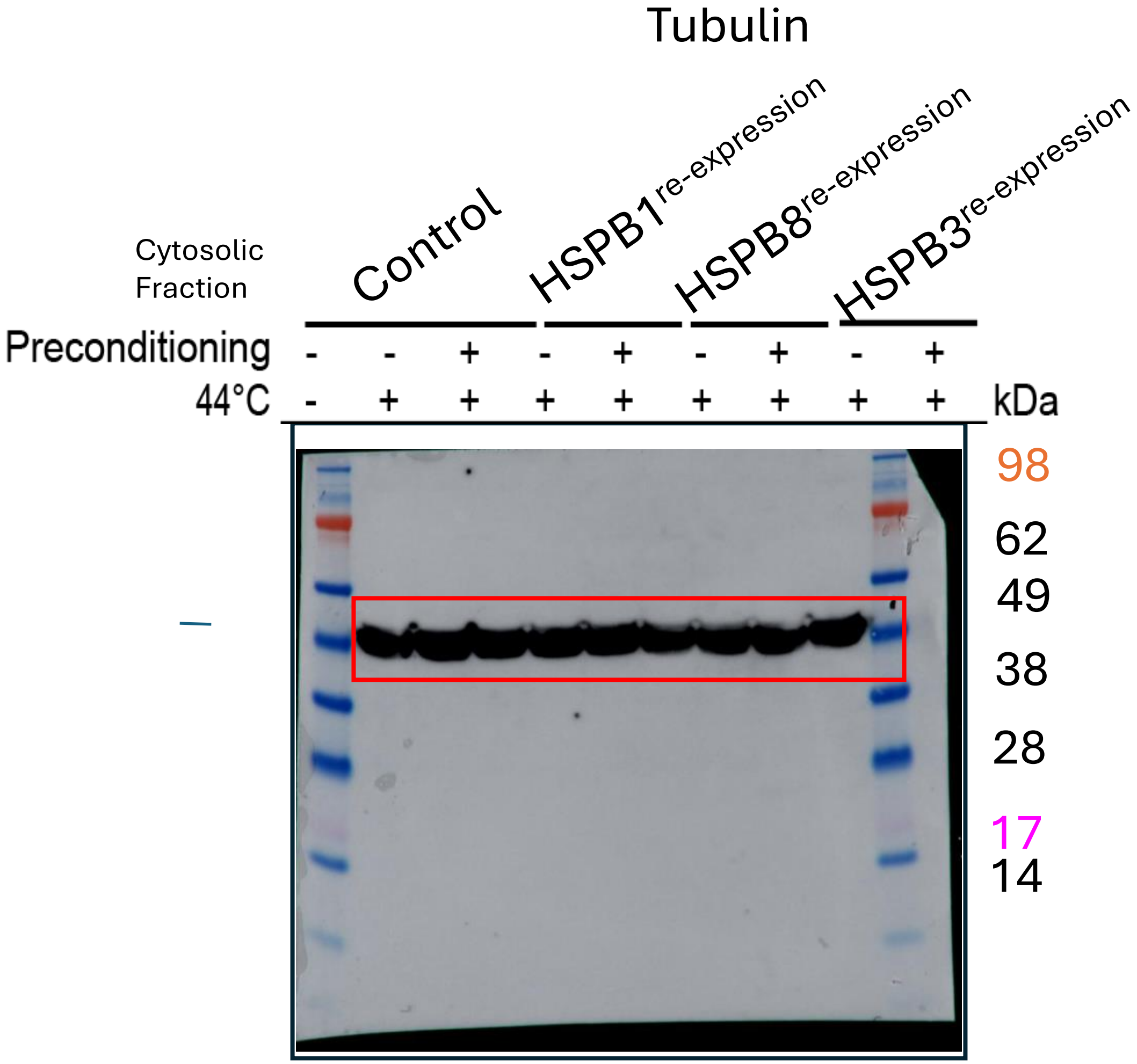

Bis-Tris 4-12% Gel
MES Buffer

Figure 5:
5B (Replicate 1)

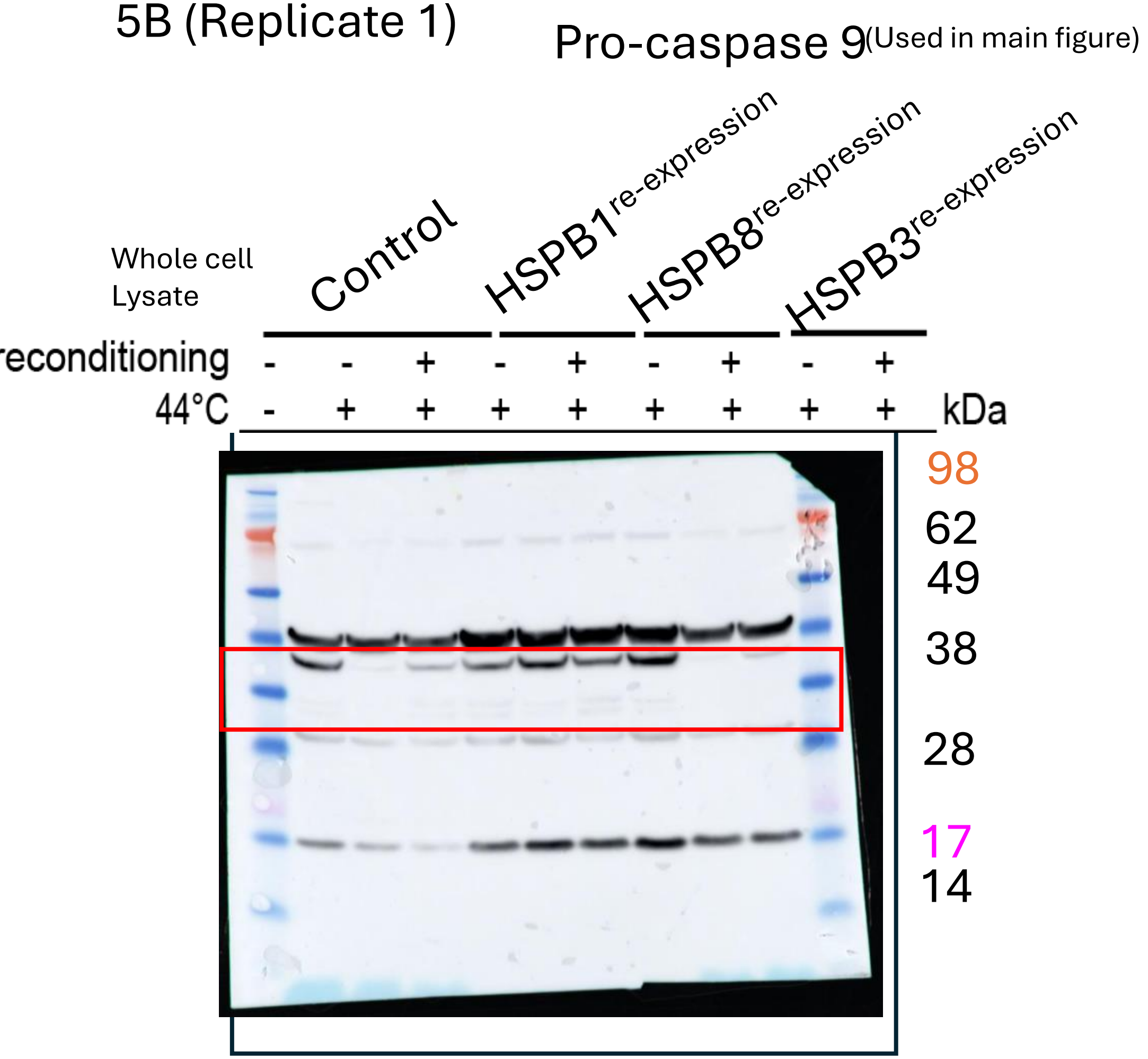

Bis-Tris 4-12% Gel
MES Buffer

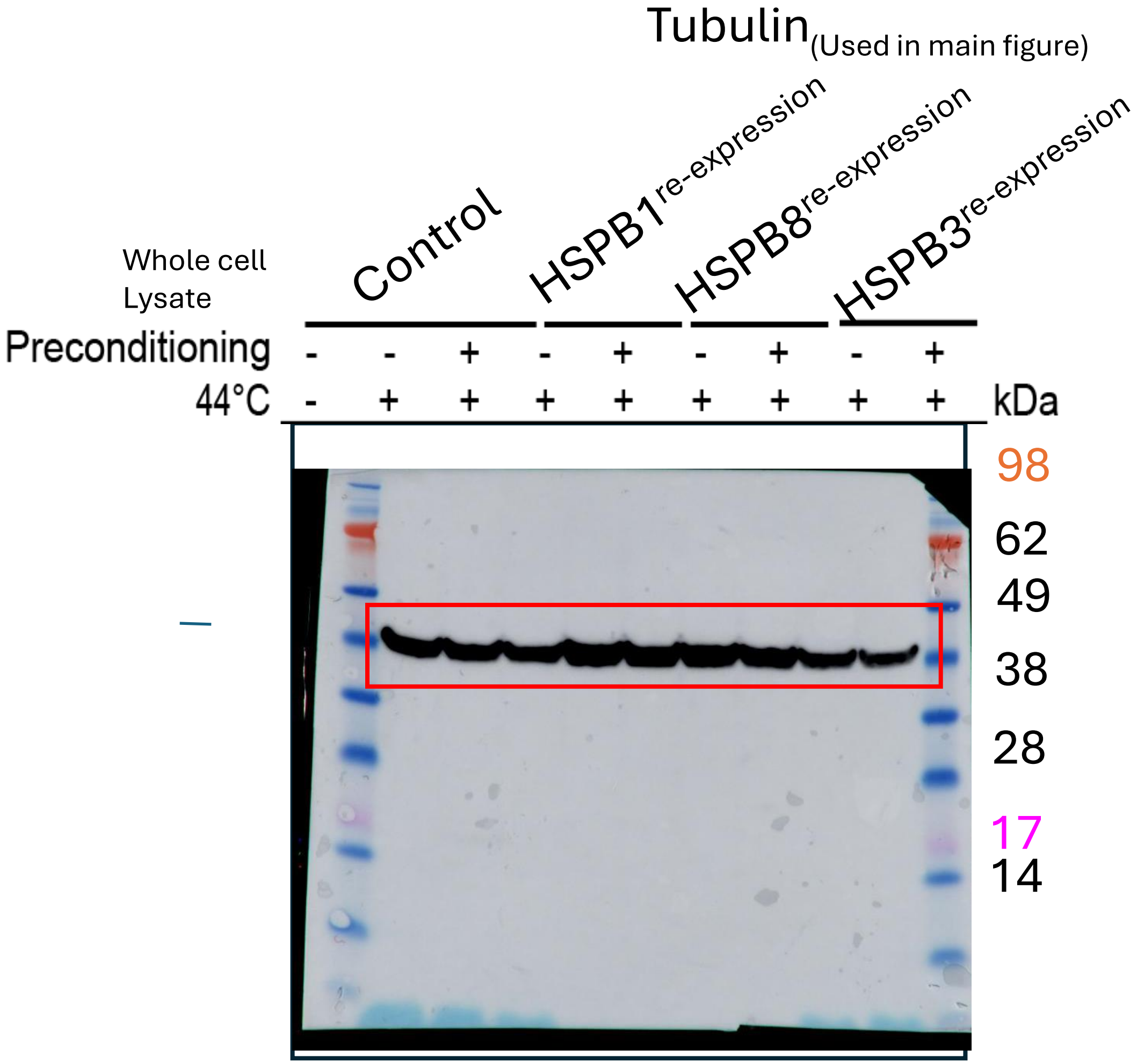

Bis-Tris 4-12% Gel
MES Buffer

Figure 5:
5B (Replicate 2)

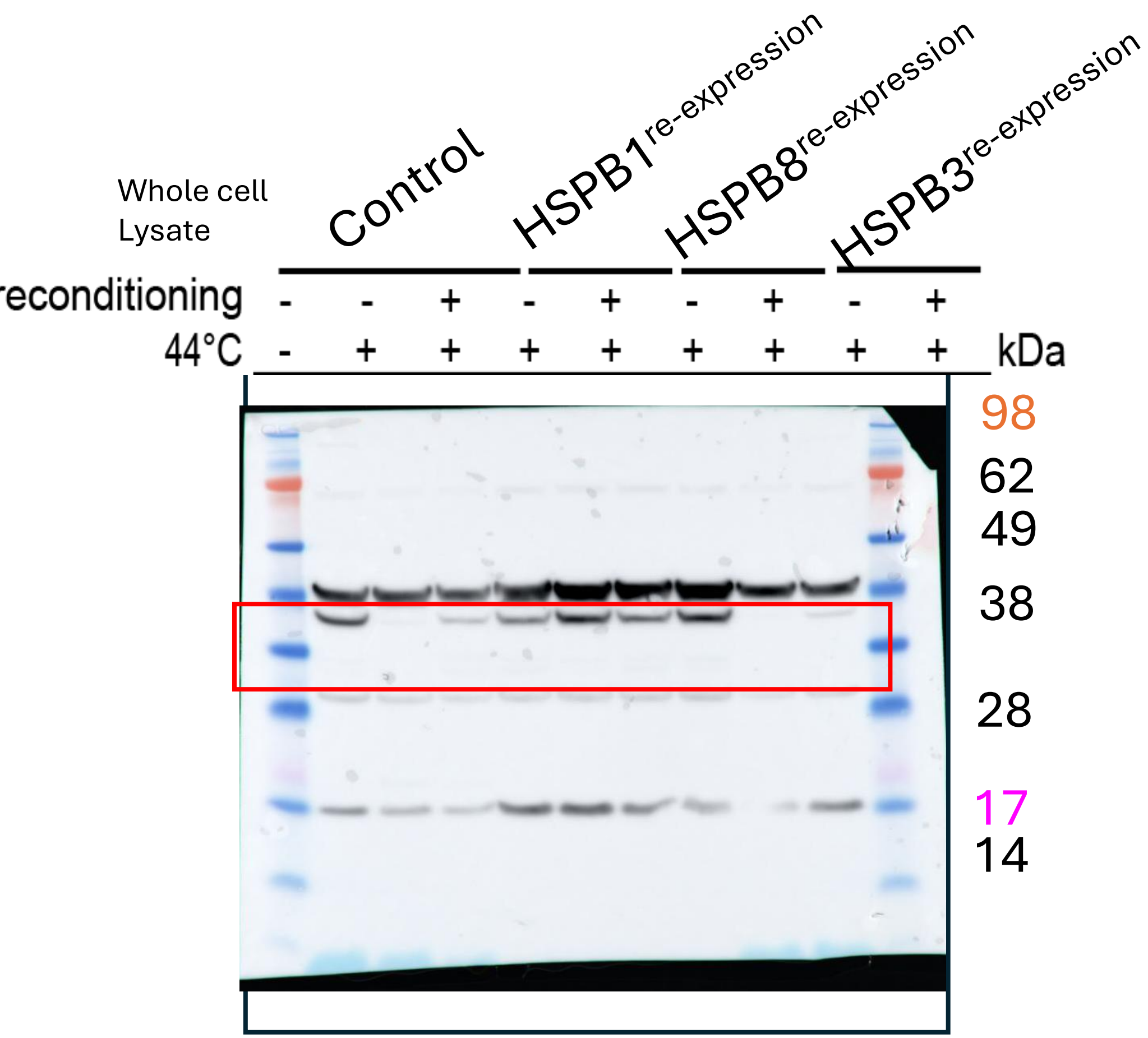

Bis-Tris 4-12% Gel
MES Buffer

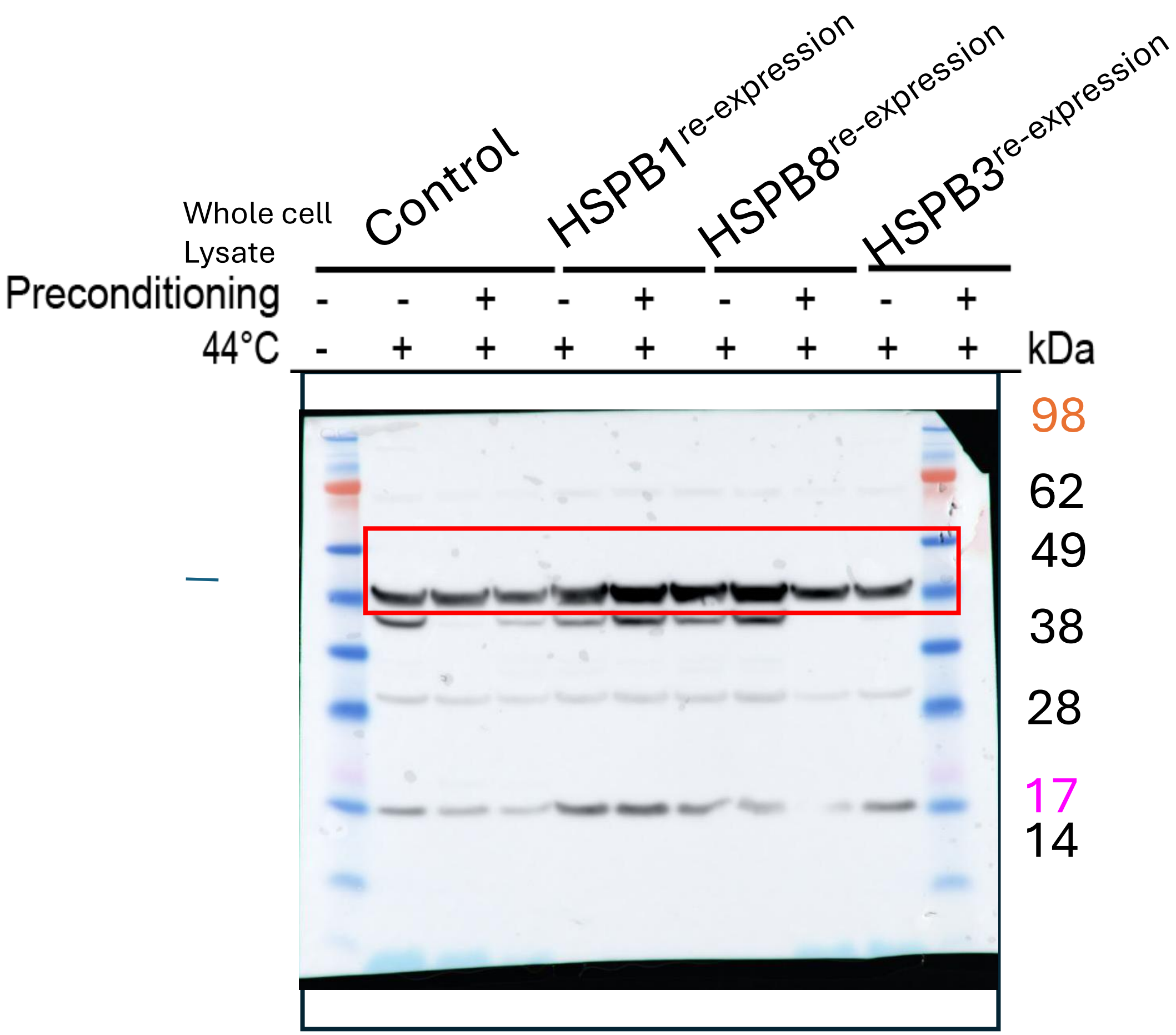

Bis-Tris 4-12% Gel
MES Buffer

Figure 5:
5B (Replicate 3)

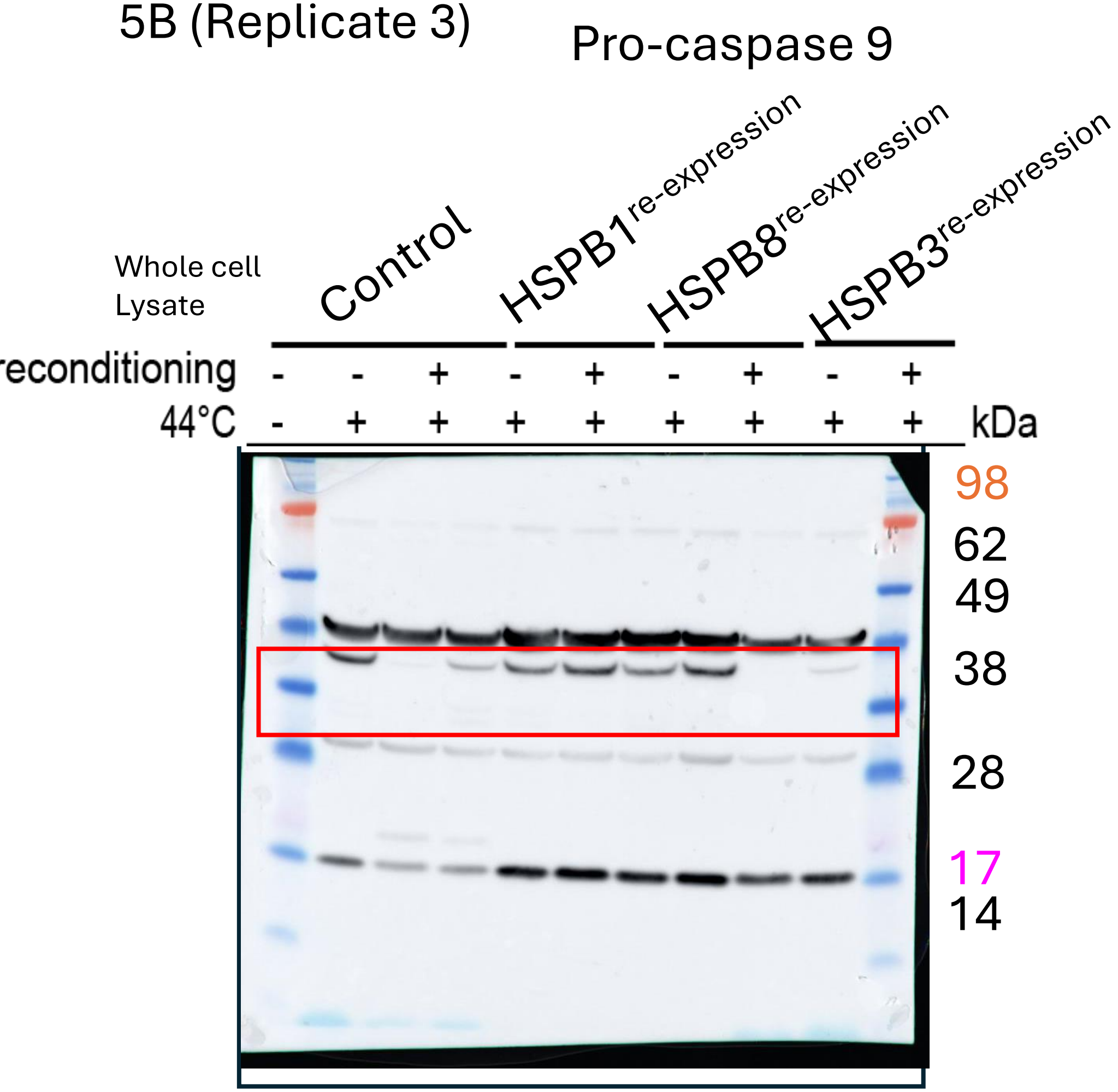

Bis-Tris 4-12% Gel
MES Buffer

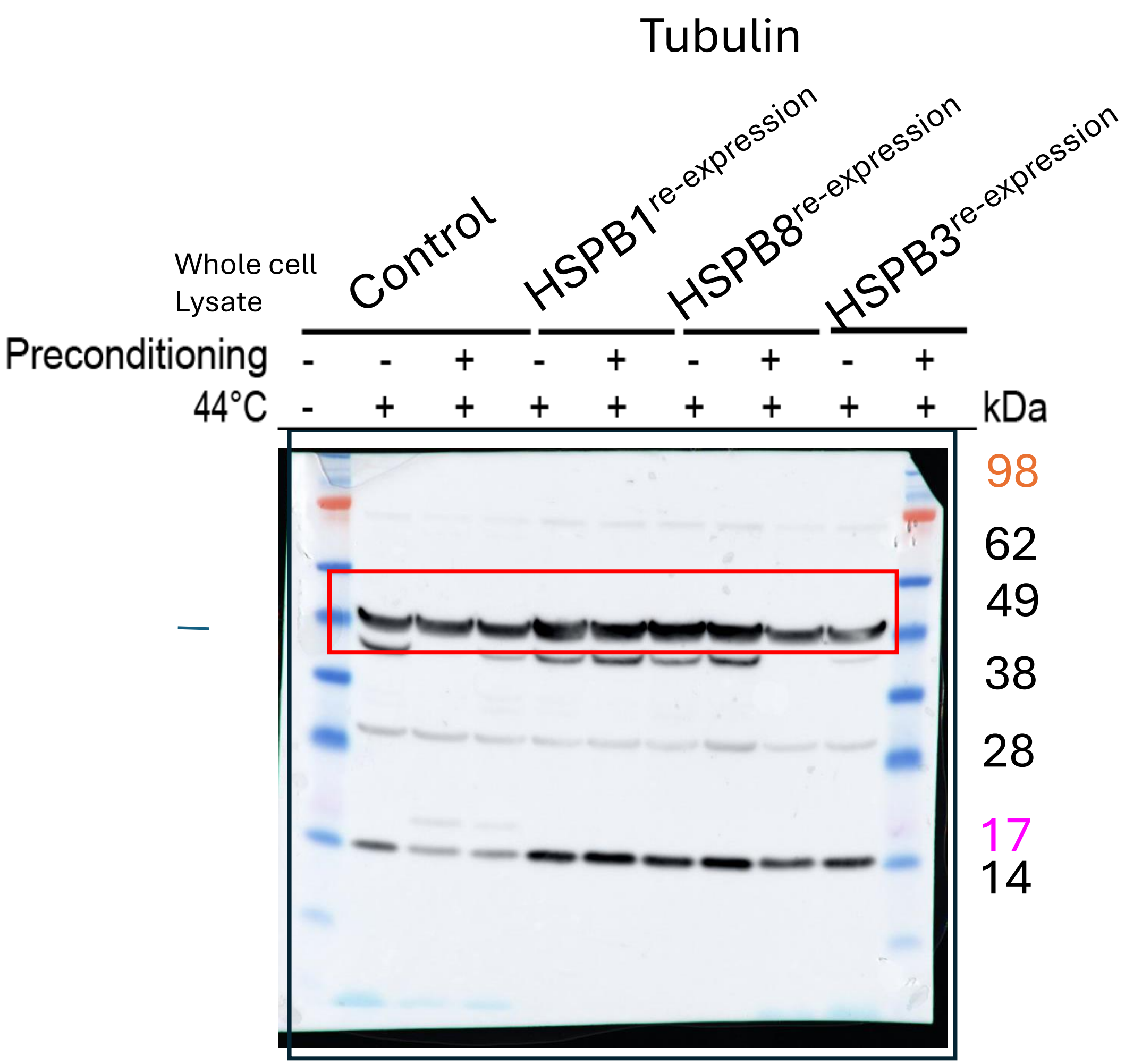

Bis-Tris 4-12% Gel
MES Buffer

Figure 5:
5C (Replicate 1)

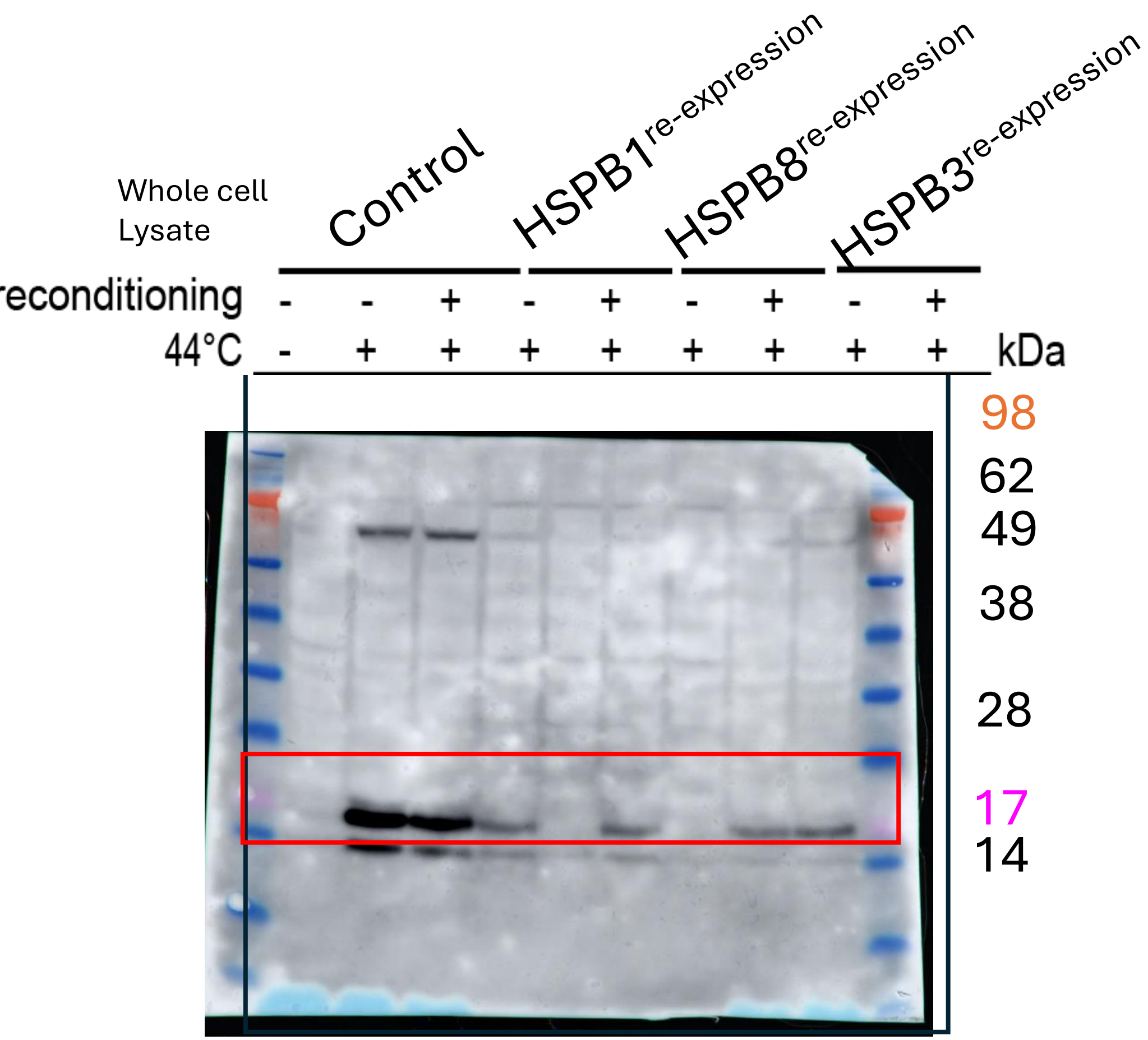

Bis-Tris 4-12% Gel
MES Buffer

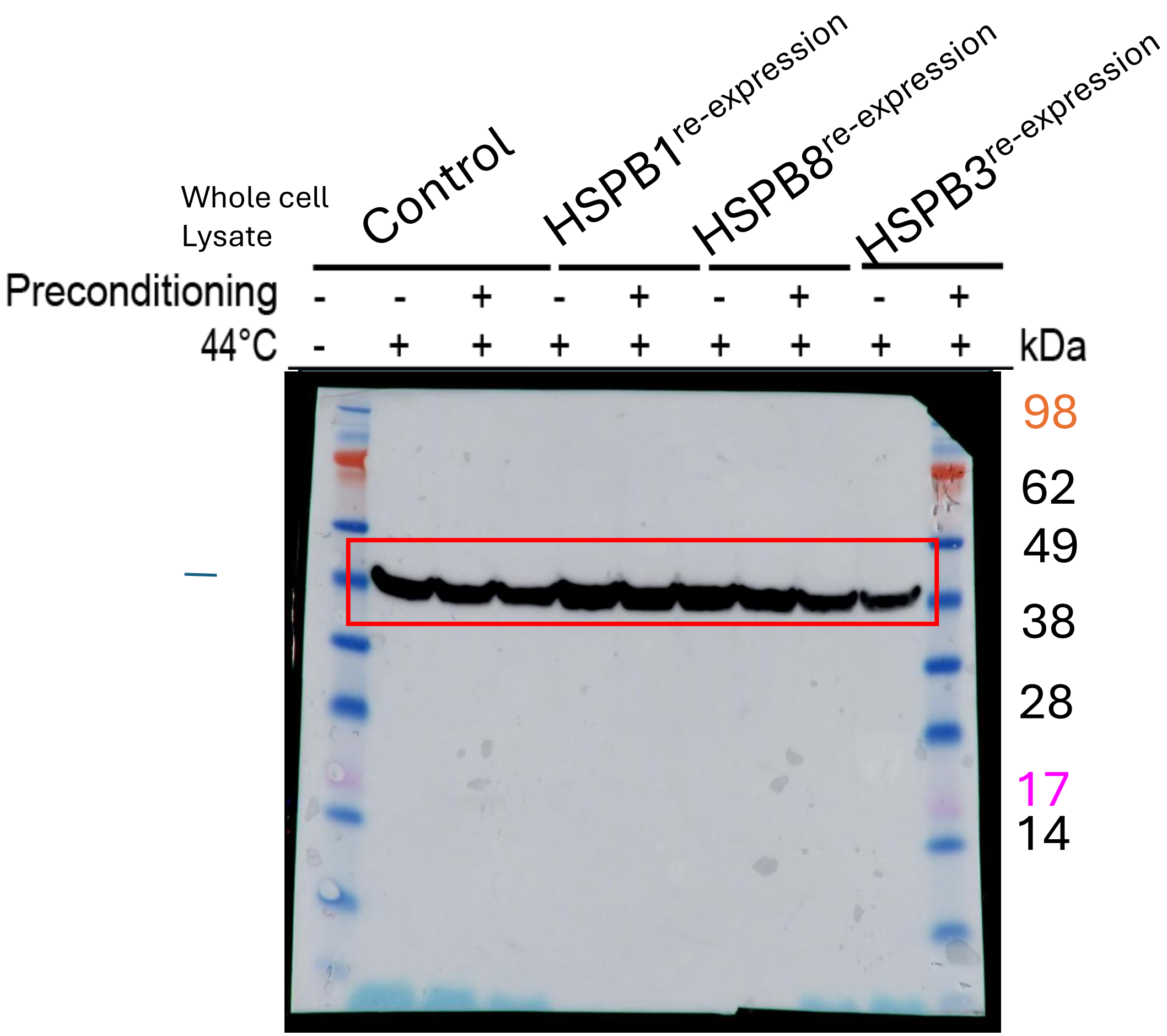

Bis-Tris 4-12% Gel
MES Buffer

Figure 5:
5C (Replicate 2)

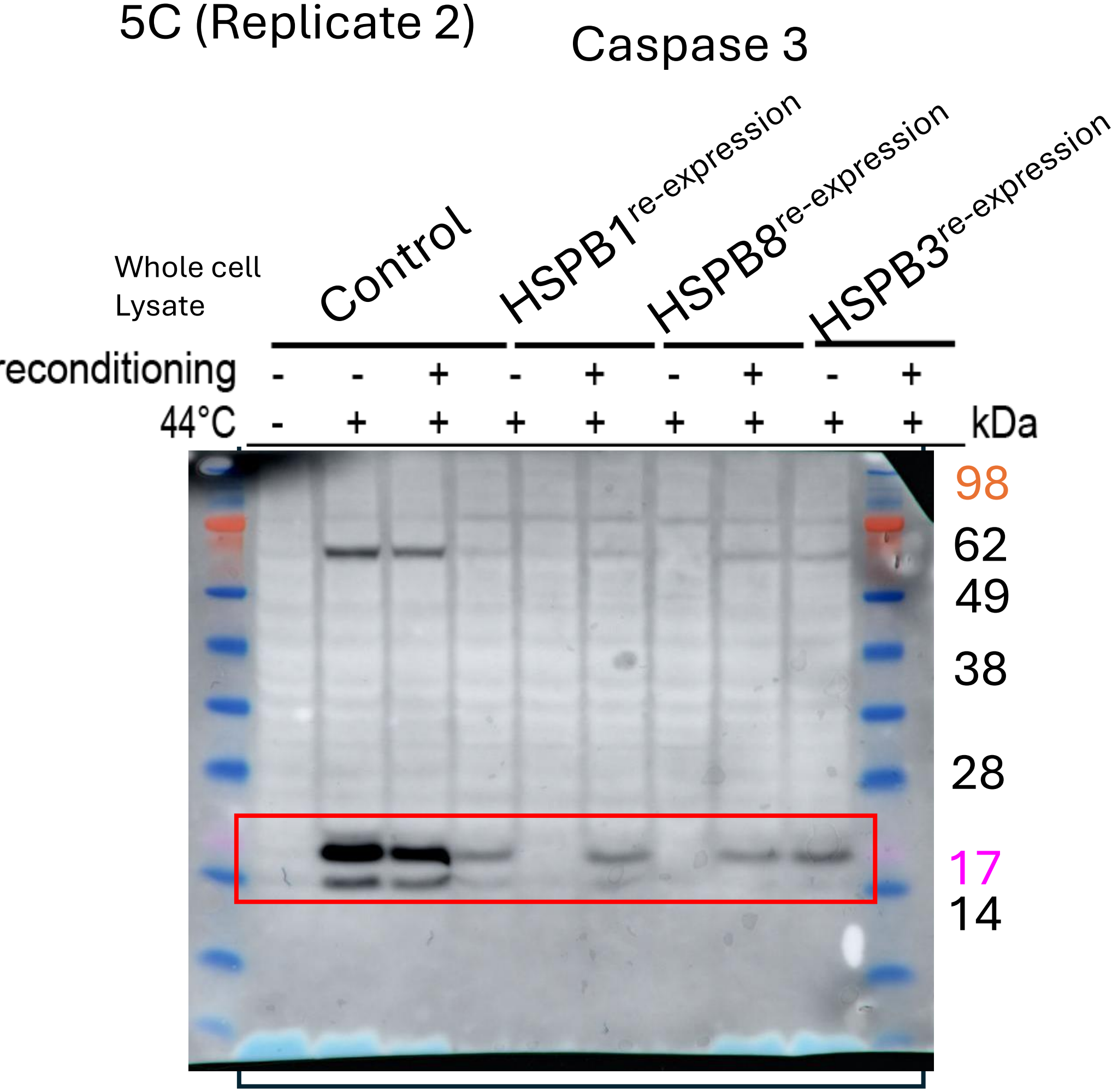

Bis-Tris 4-12% Gel
MES Buffer

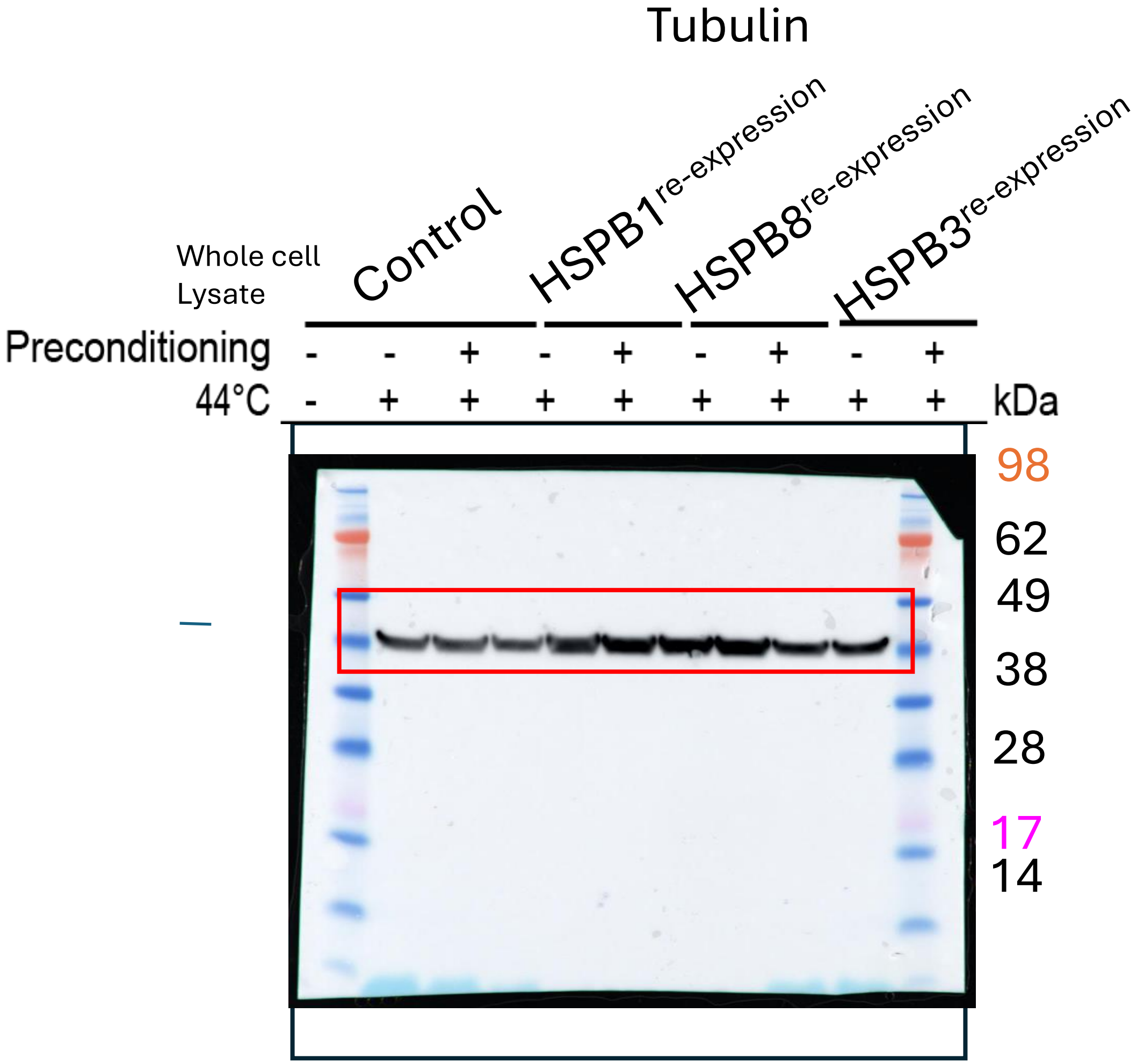

Bis-Tris 4-12% Gel
MES Buffer

Figure 5:
5C (Replicate 3)

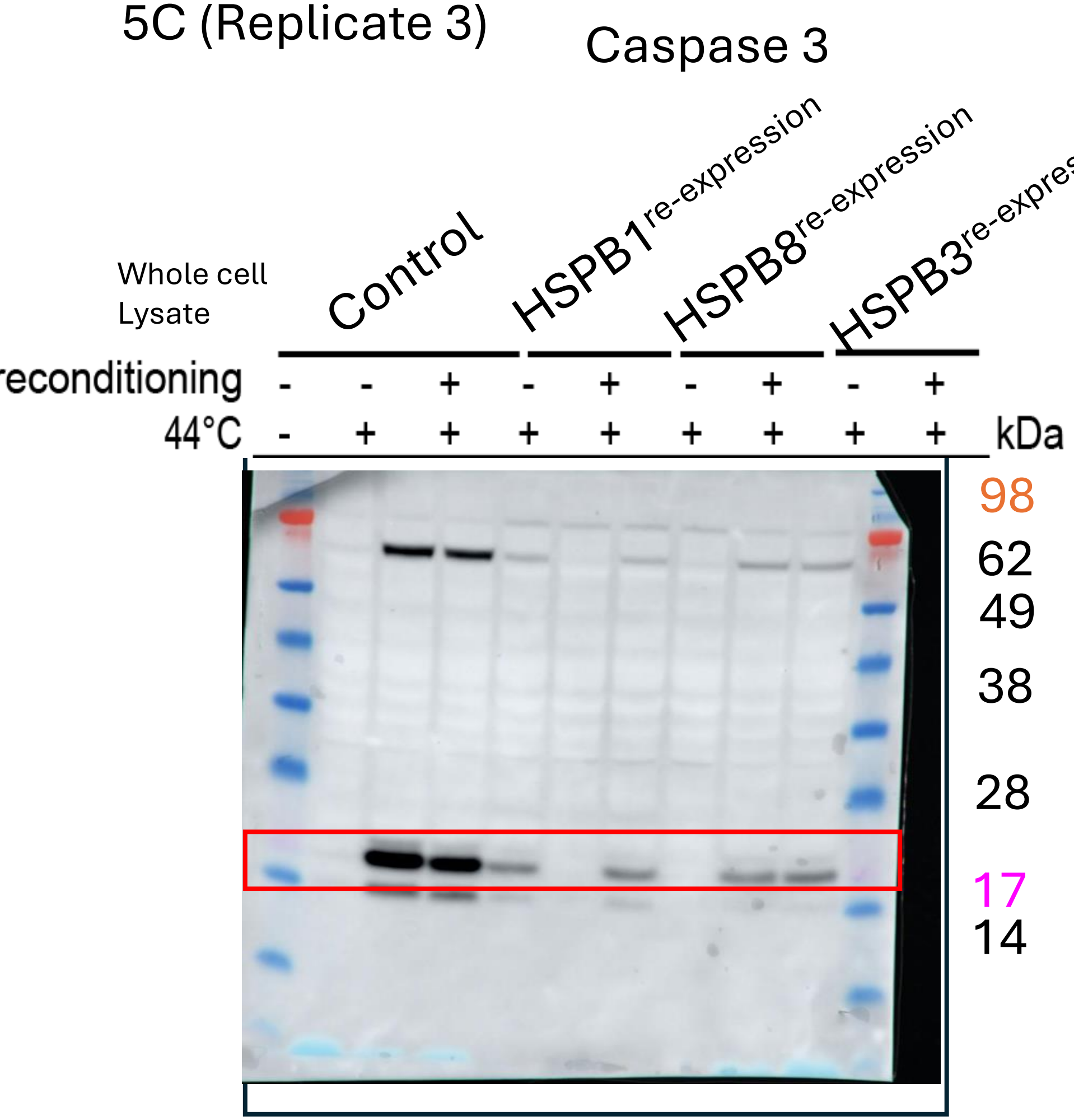

Bis-Tris 4-12% Gel
MES Buffer

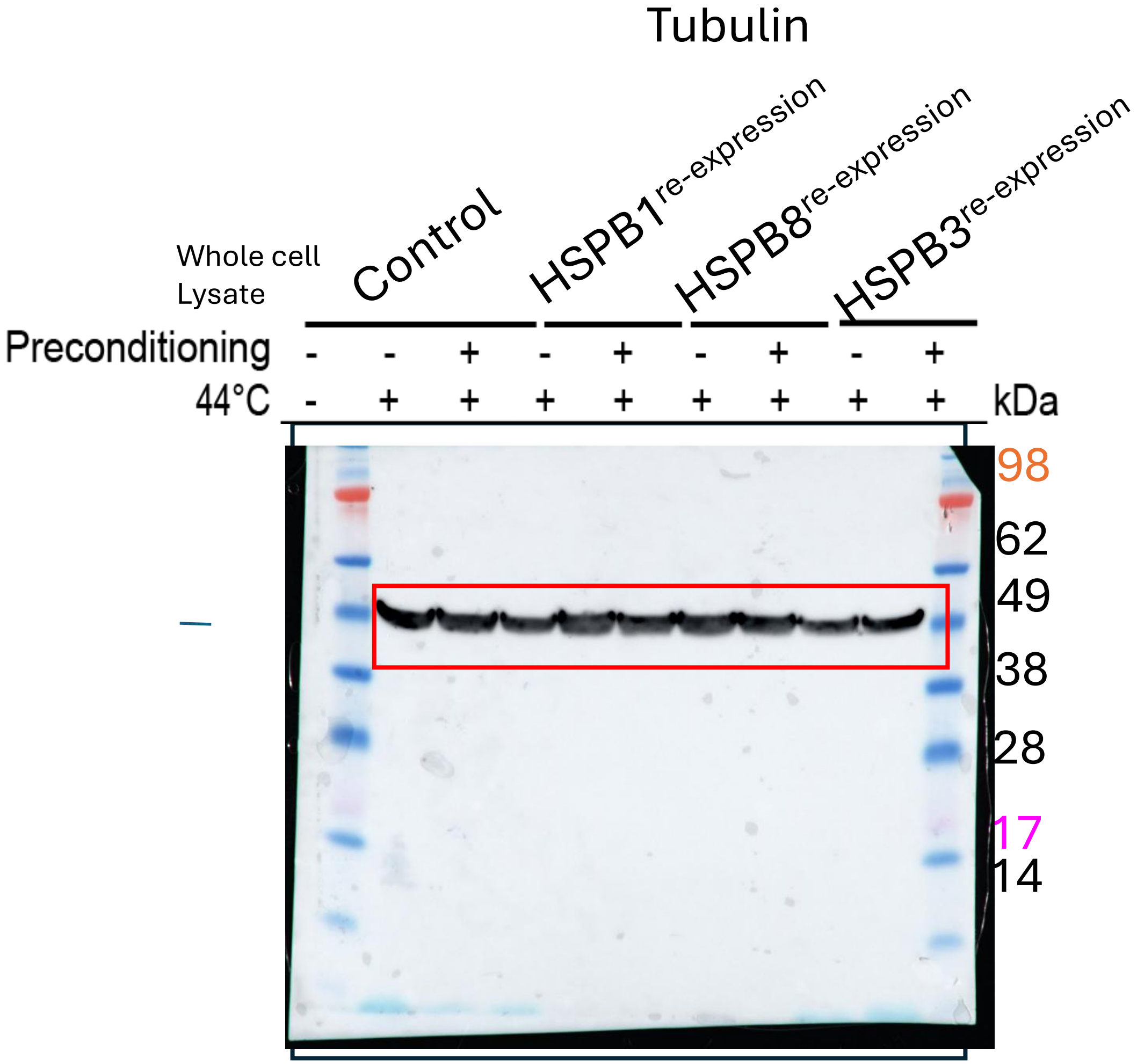

Bis-Tris 4-12% Gel
MES Buffer

Figure 5:

5D (Replicate 1)

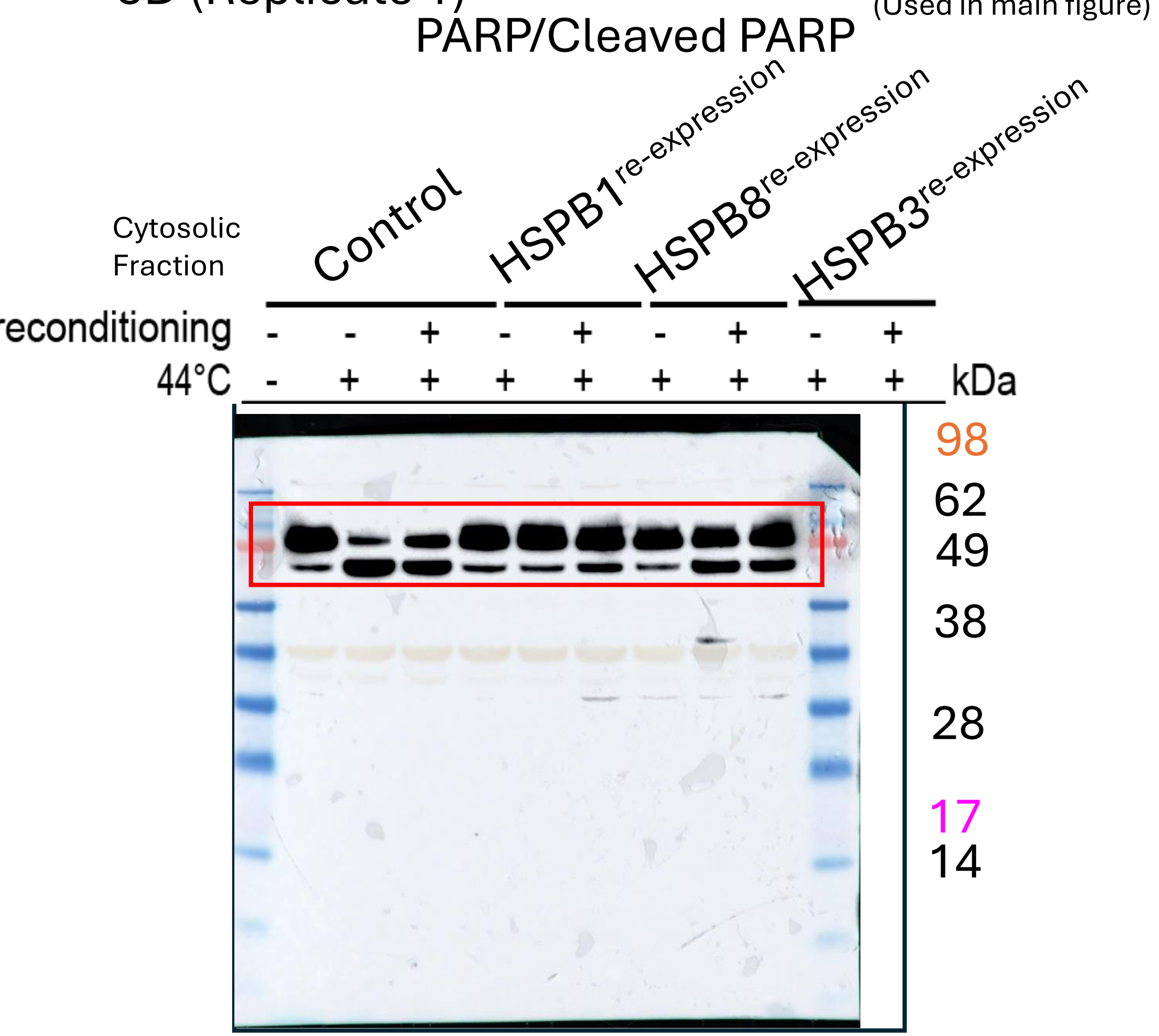

Bis-Tris 4-12% Gel
MES Buffer

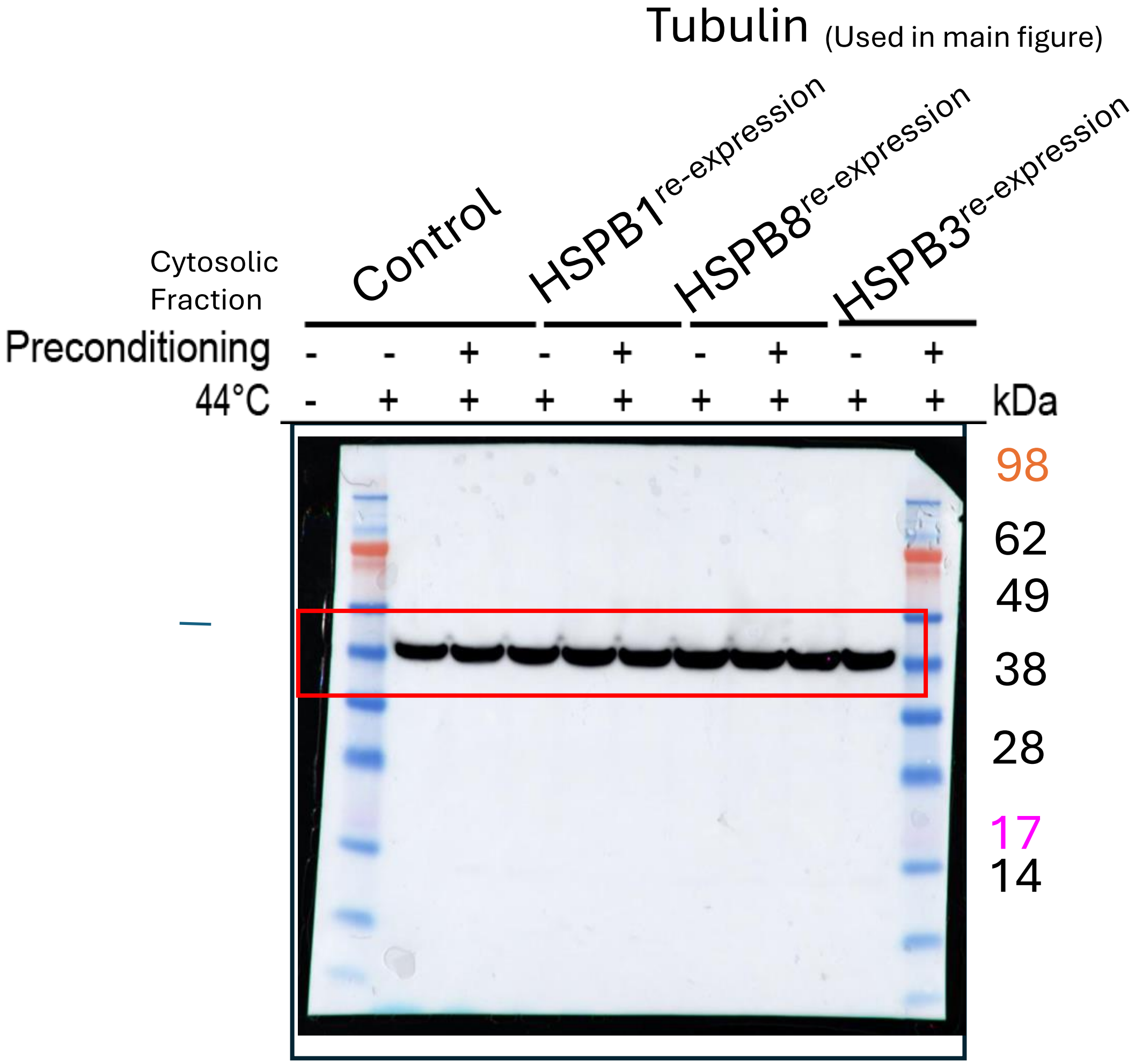

Bis-Tris 4-12% Gel
MES Buffer

Figure 5:
5D (Replicate 2)

PARP/Cleaved PARP

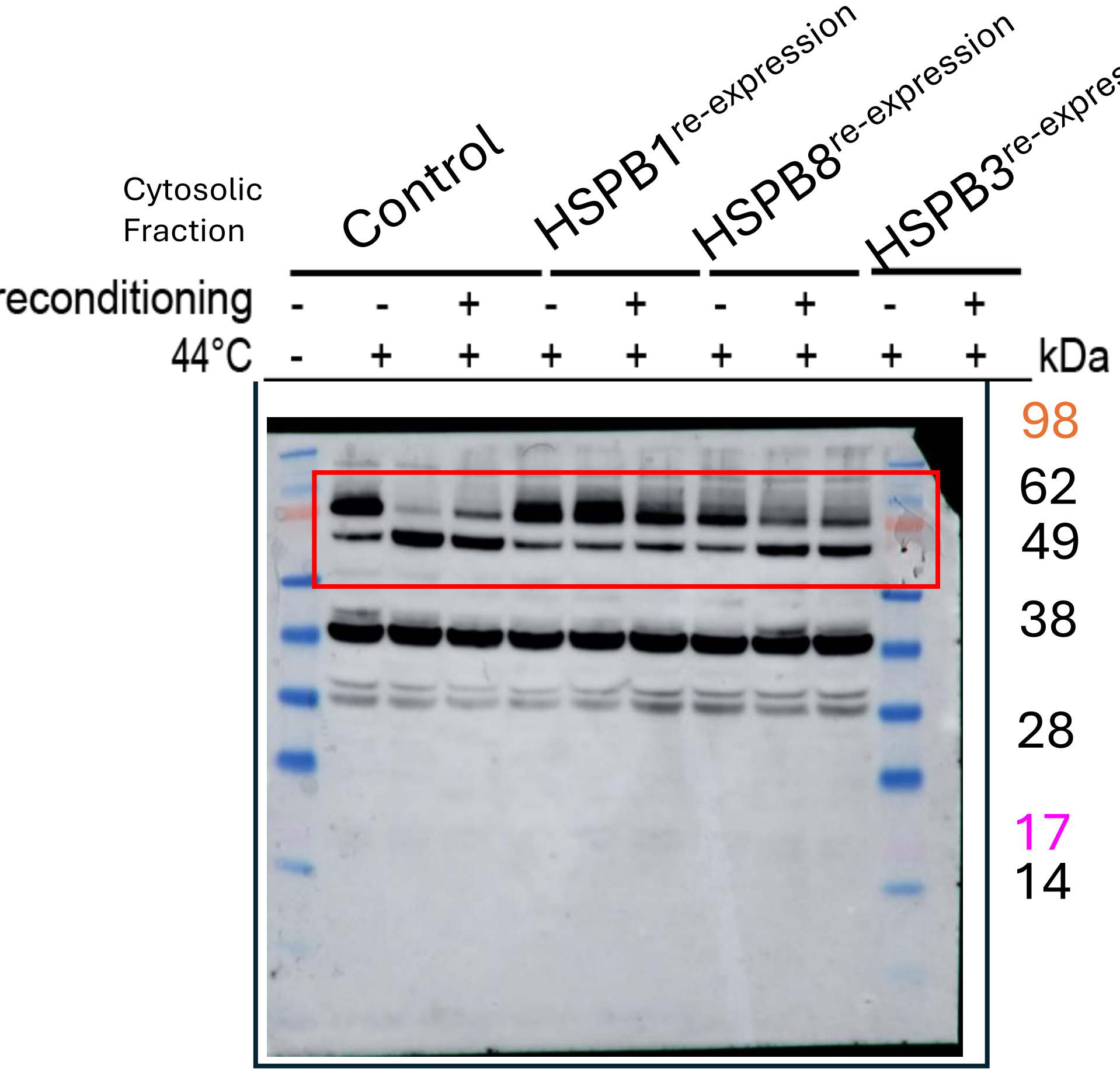

Bis-Tris 4-12% Gel
MES Buffer

Tubulin

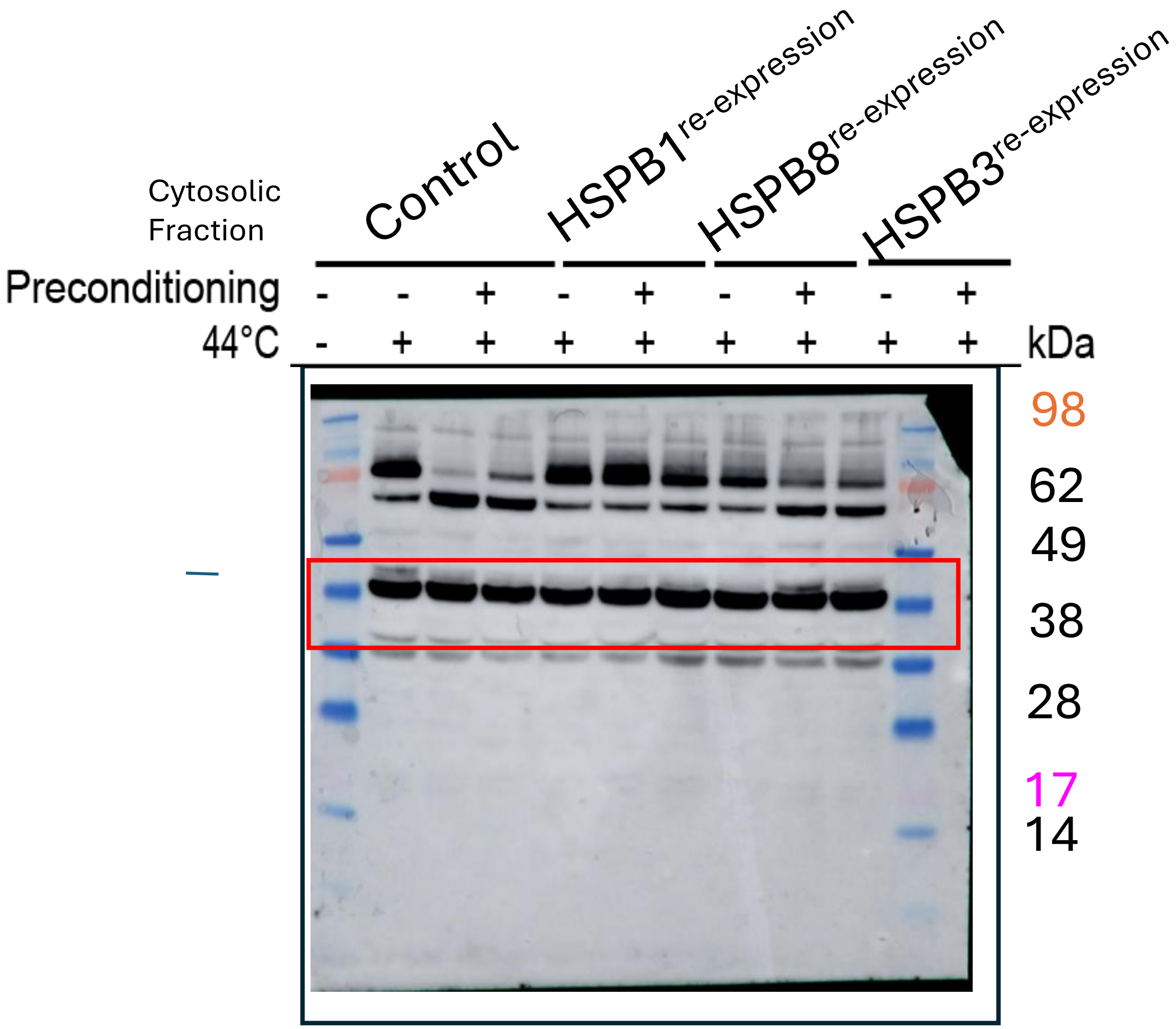

Bis-Tris 4-12% Gel
MES Buffer

Figure 5:
5D (Replicate 3)

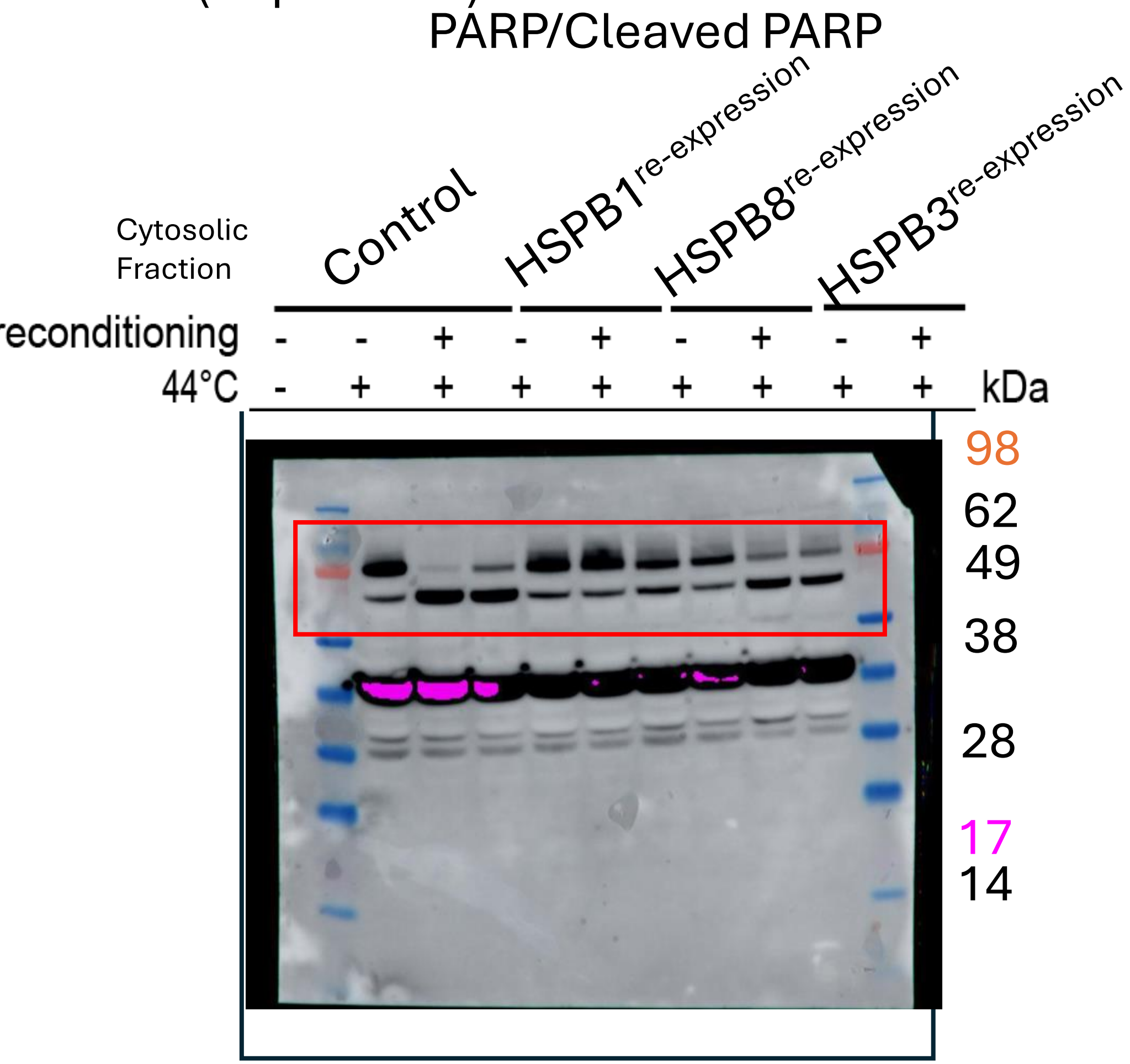

Bis-Tris 4-12% Gel
MES Buffer

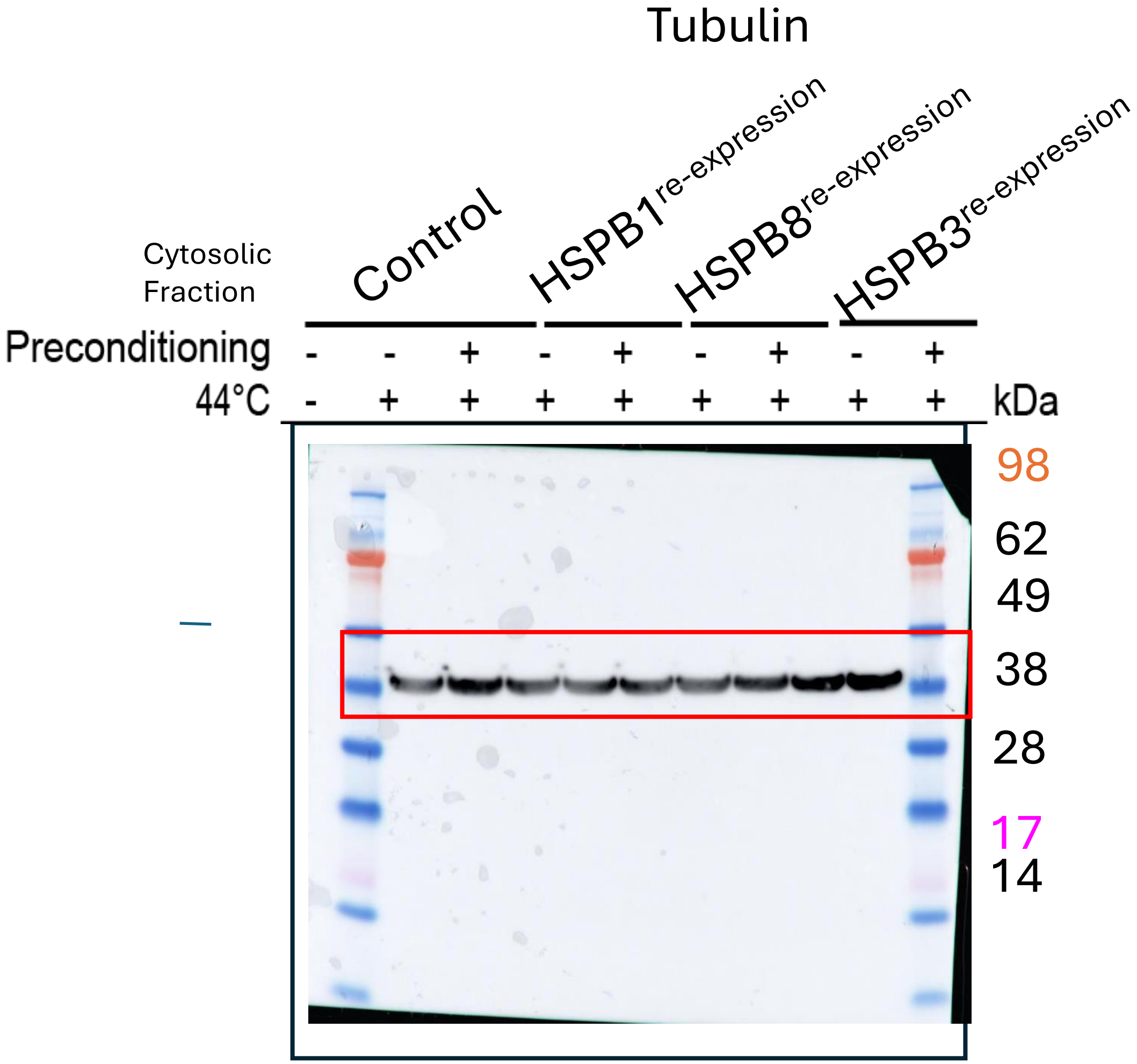

Bis-Tris 4-12% Gel
MES Buffer

Figure 5:
5D (Replicate 4)

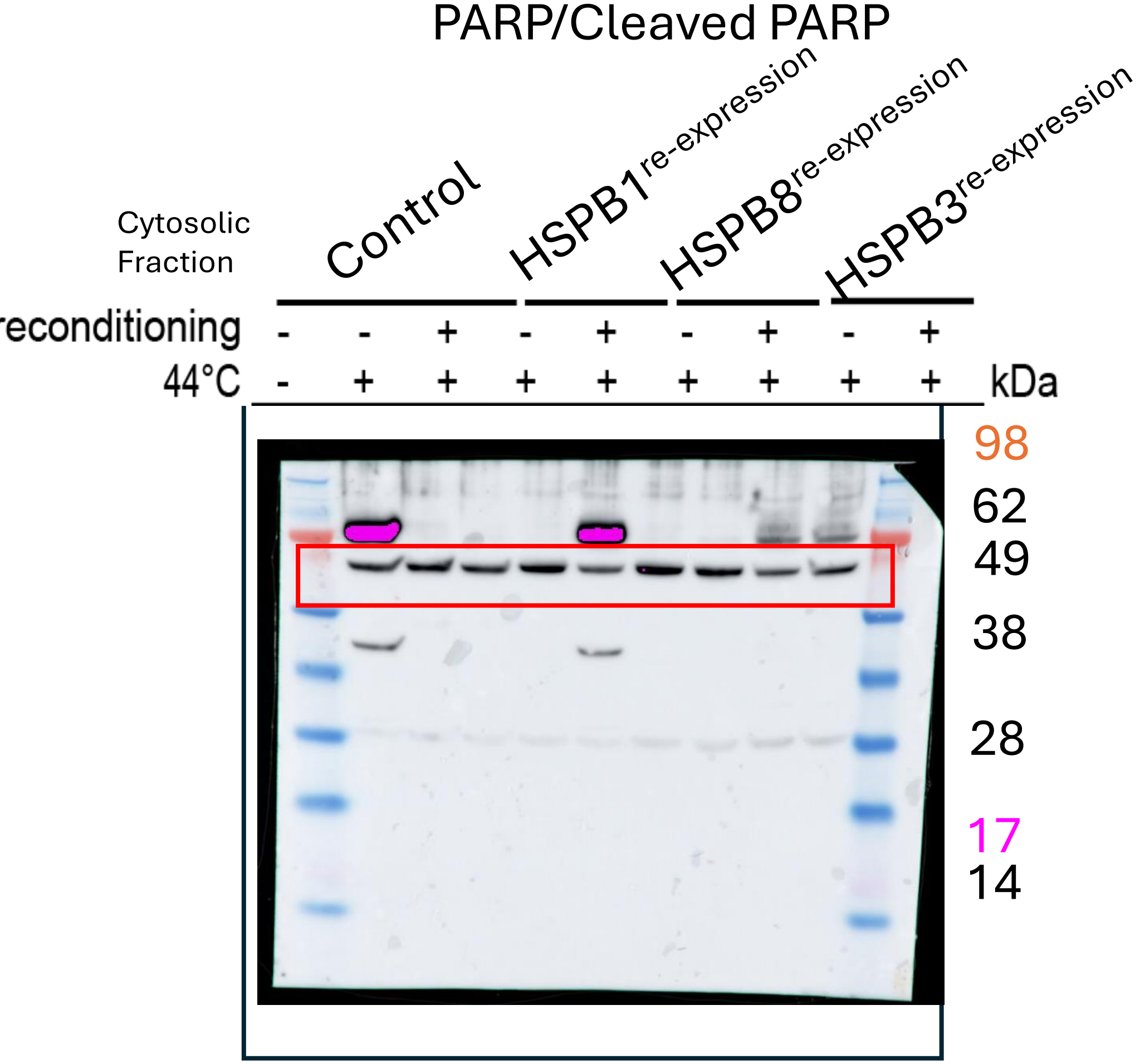

Bis-Tris 4-12% Gel
MES Buffer

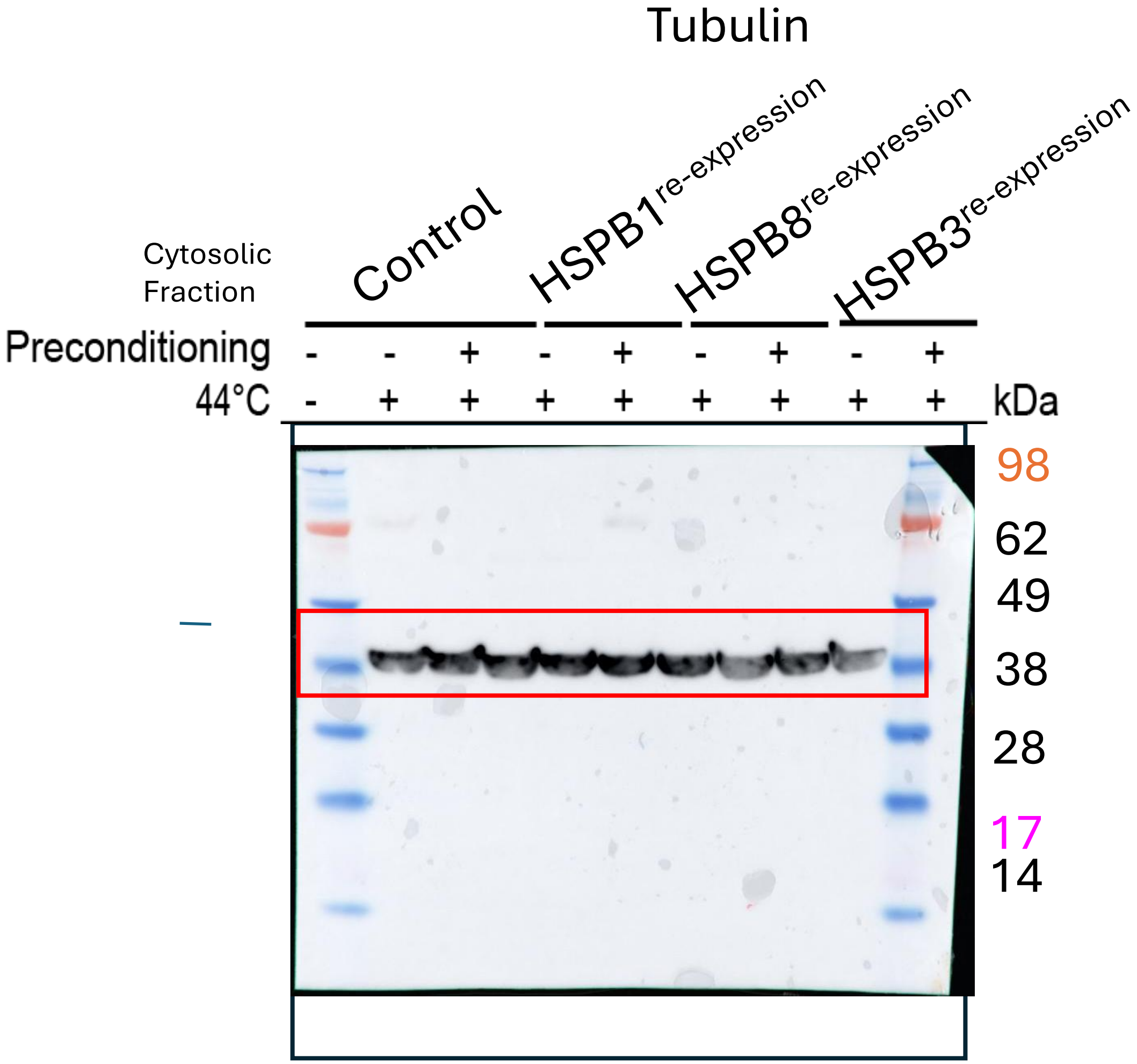

Bis-Tris 4-12% Gel
MES Buffer

Figure Supplementary Figure 1:
Proteinase K assay (HSPB8)

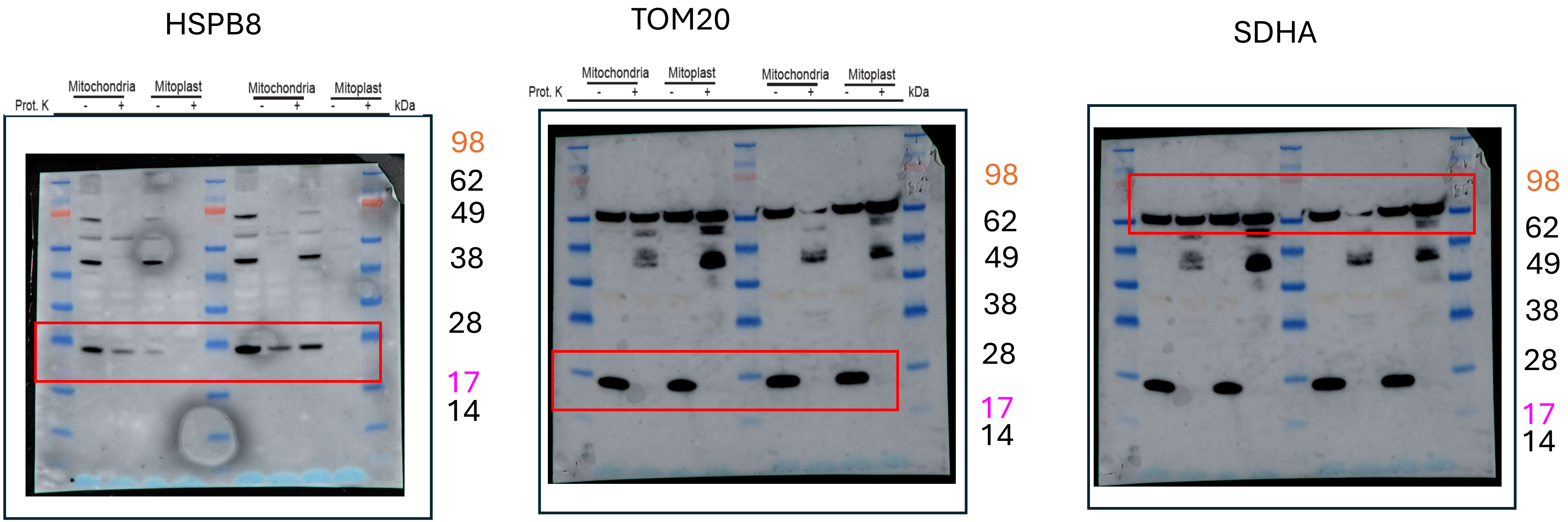

Bis-Tris 4-12% Gel
MES Buffer

Bis-Tris 4-12% Gel
MES Buffer

Bis-Tris 4-12% Gel
MES Buffer

Figure Supplementary Figure 1:
Proteinase K assay (HSPB8)

AIFM1

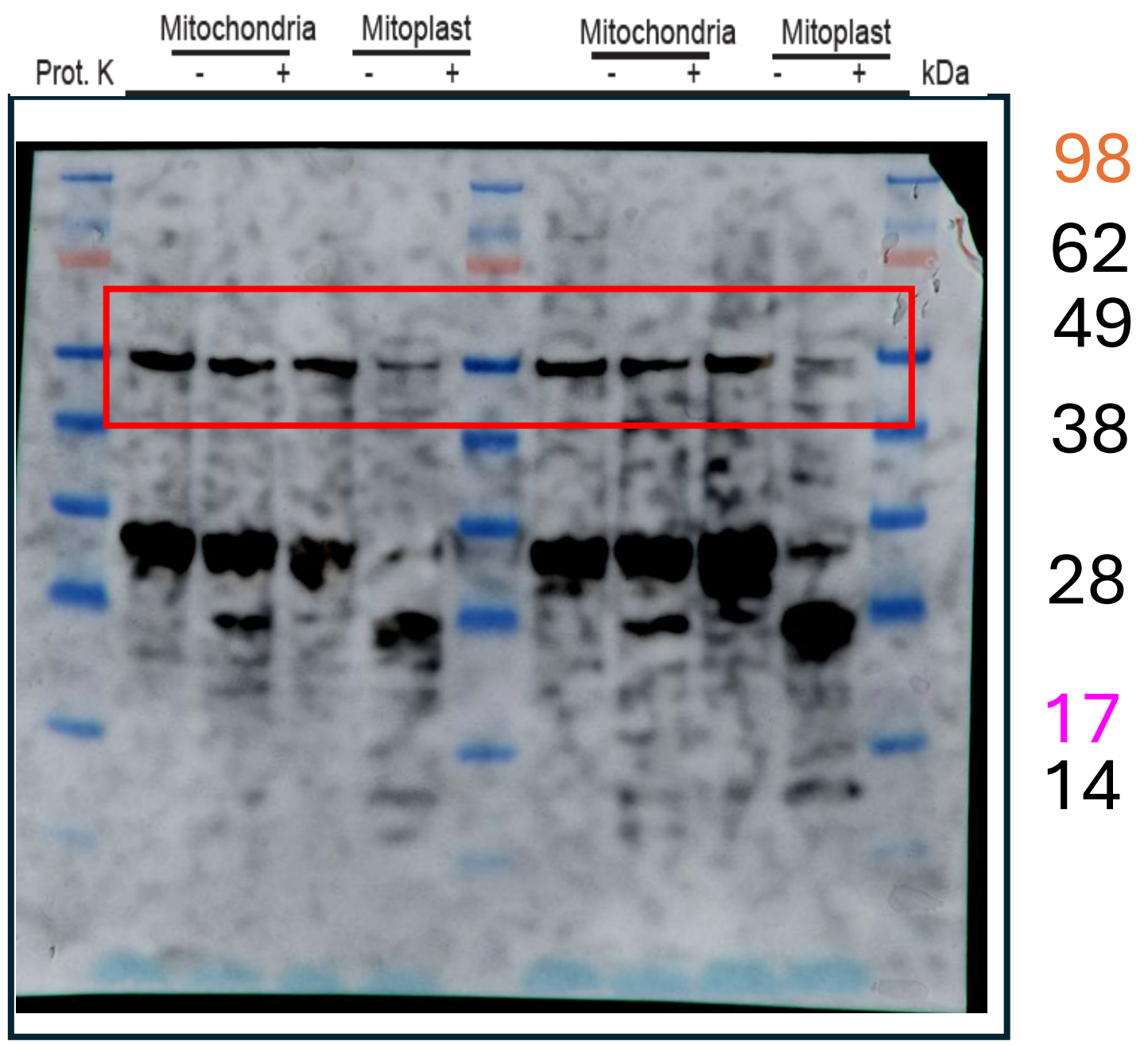

Bis-Tris 4-12% Gel
MES Buffer

HCCS

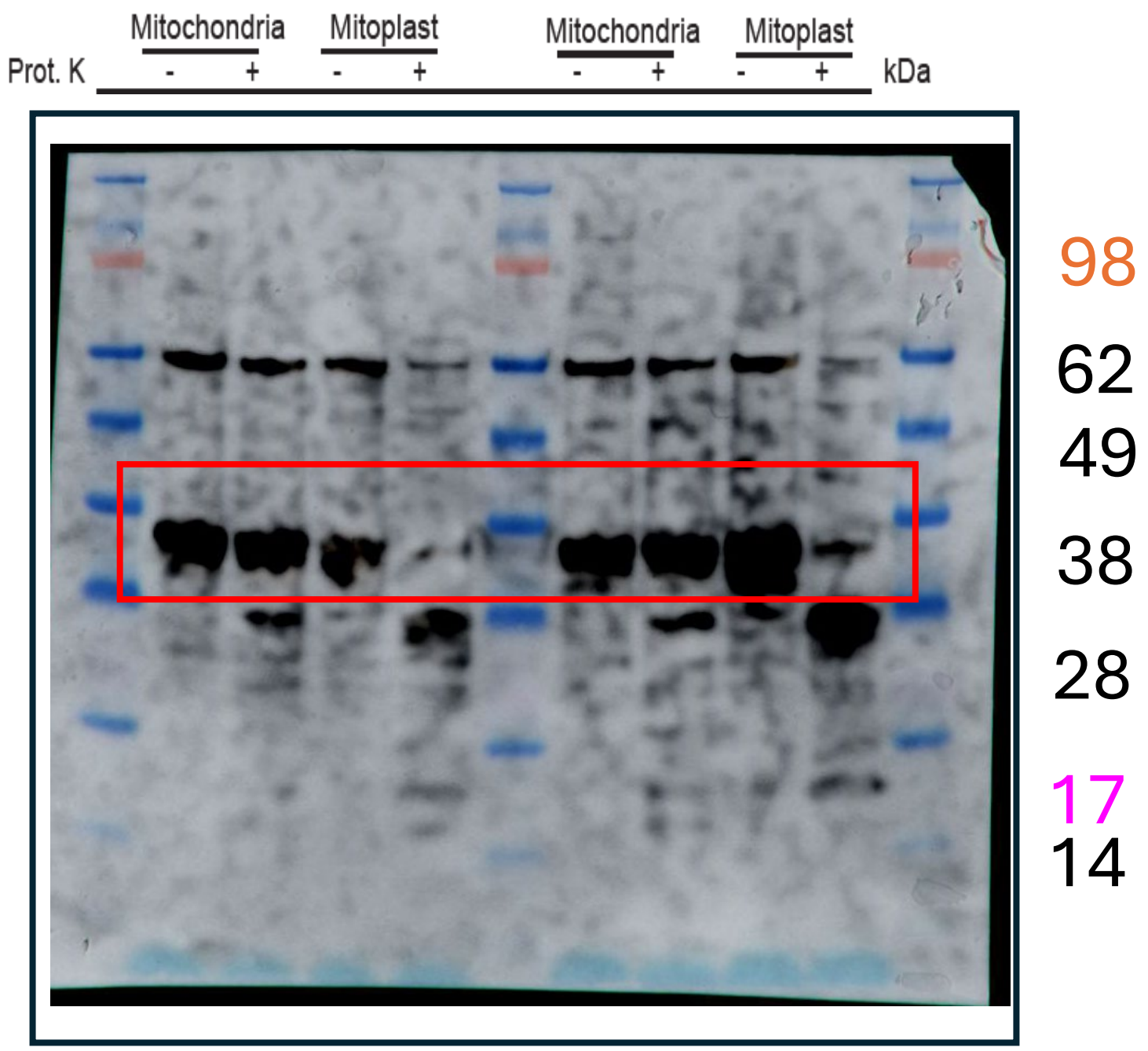

Bis-Tris 4-12% Gel
MES Buffer

Figure Supplementary Figure 2:
HSPB translocation in response to mild heat stress
SH-SY5Y Neurons

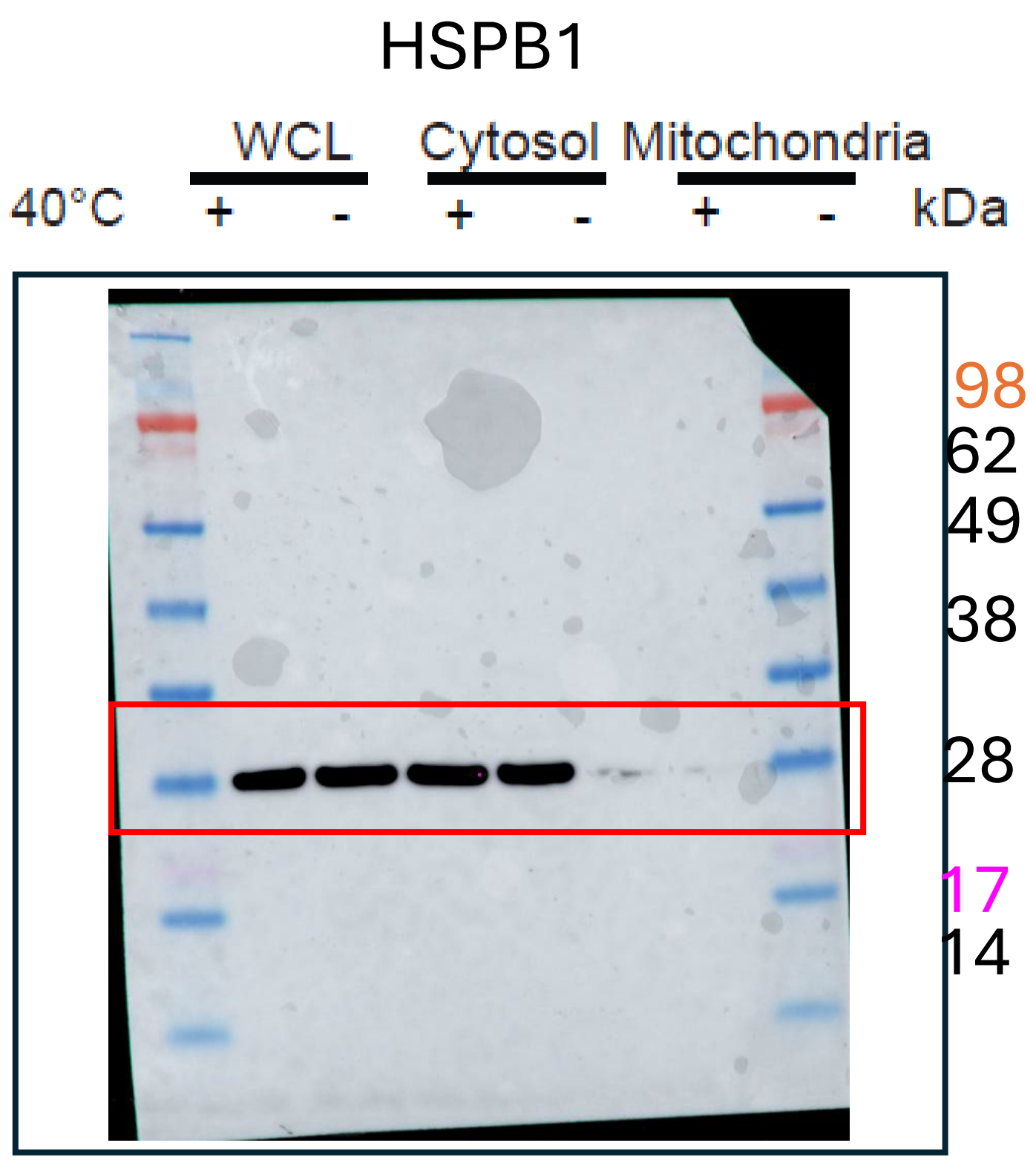

Bis-Tris 4-12% Gel
MES Buffer

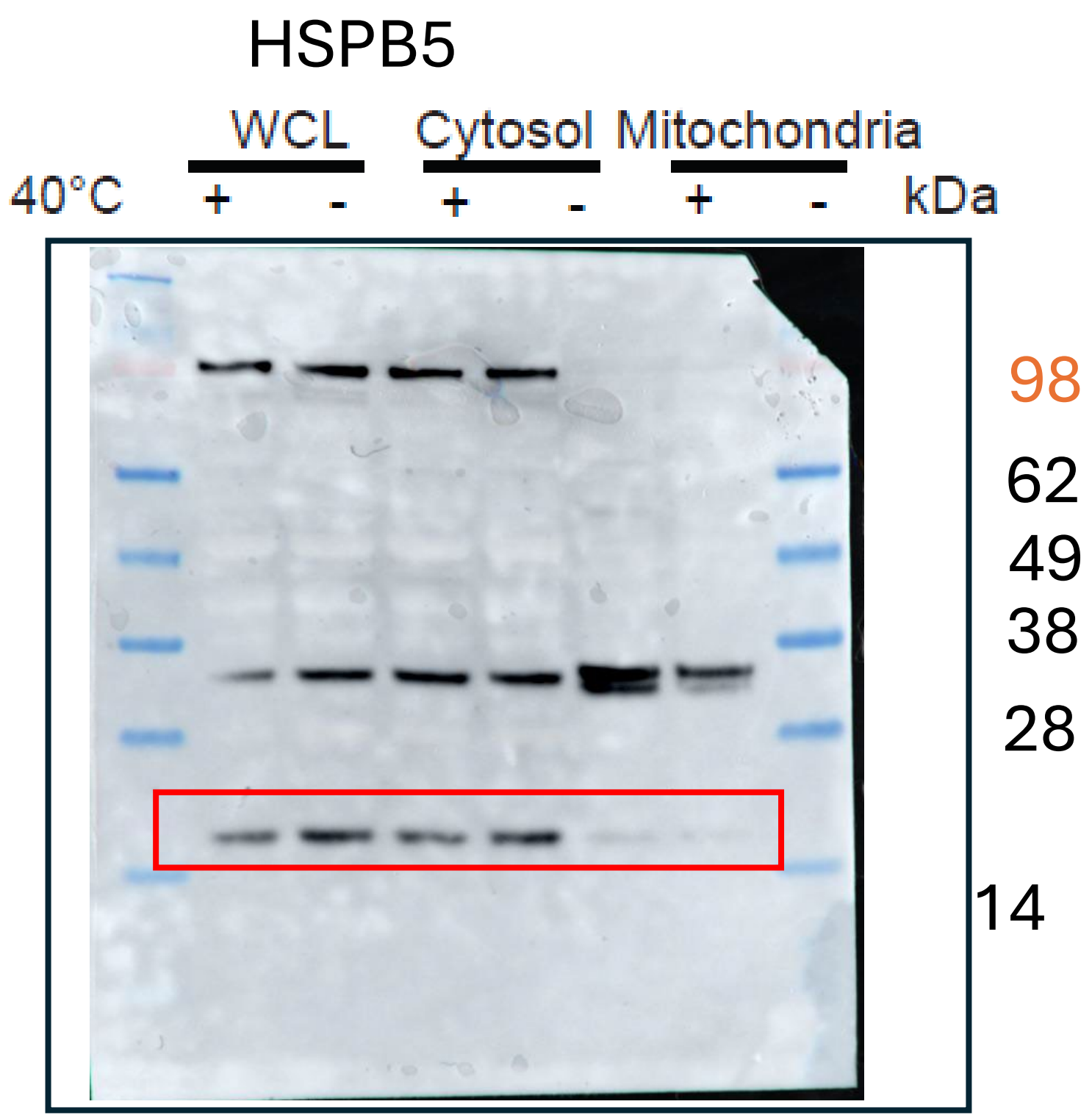

Bis-Tris 4-12% Gel
MES Buffer

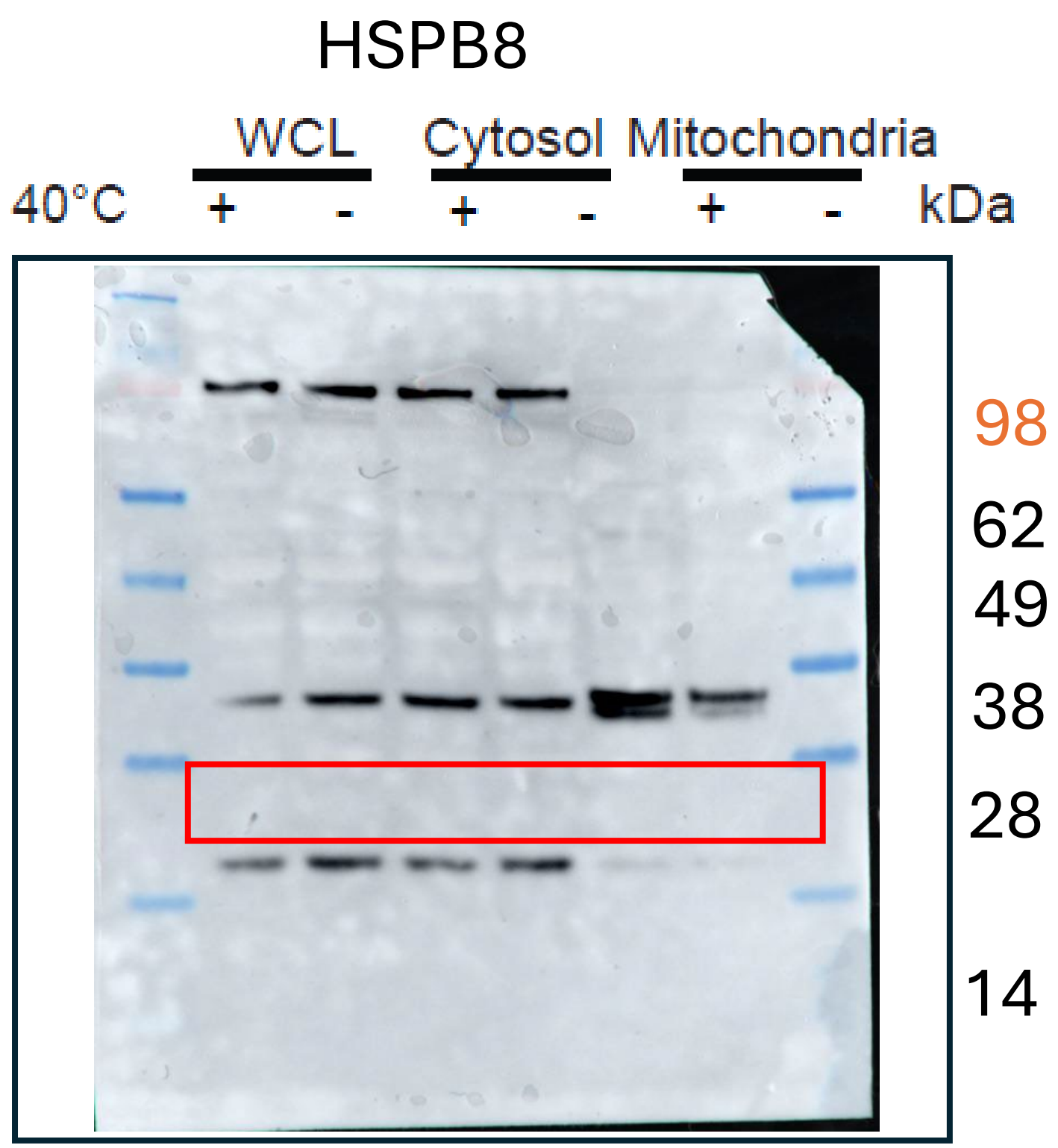

Bis-Tris 4-12% Gel
MES Buffer

Figure Supplementary Figure 2:
HSPB translocation in response to mild heat stress
SH-SY5Y Neurons

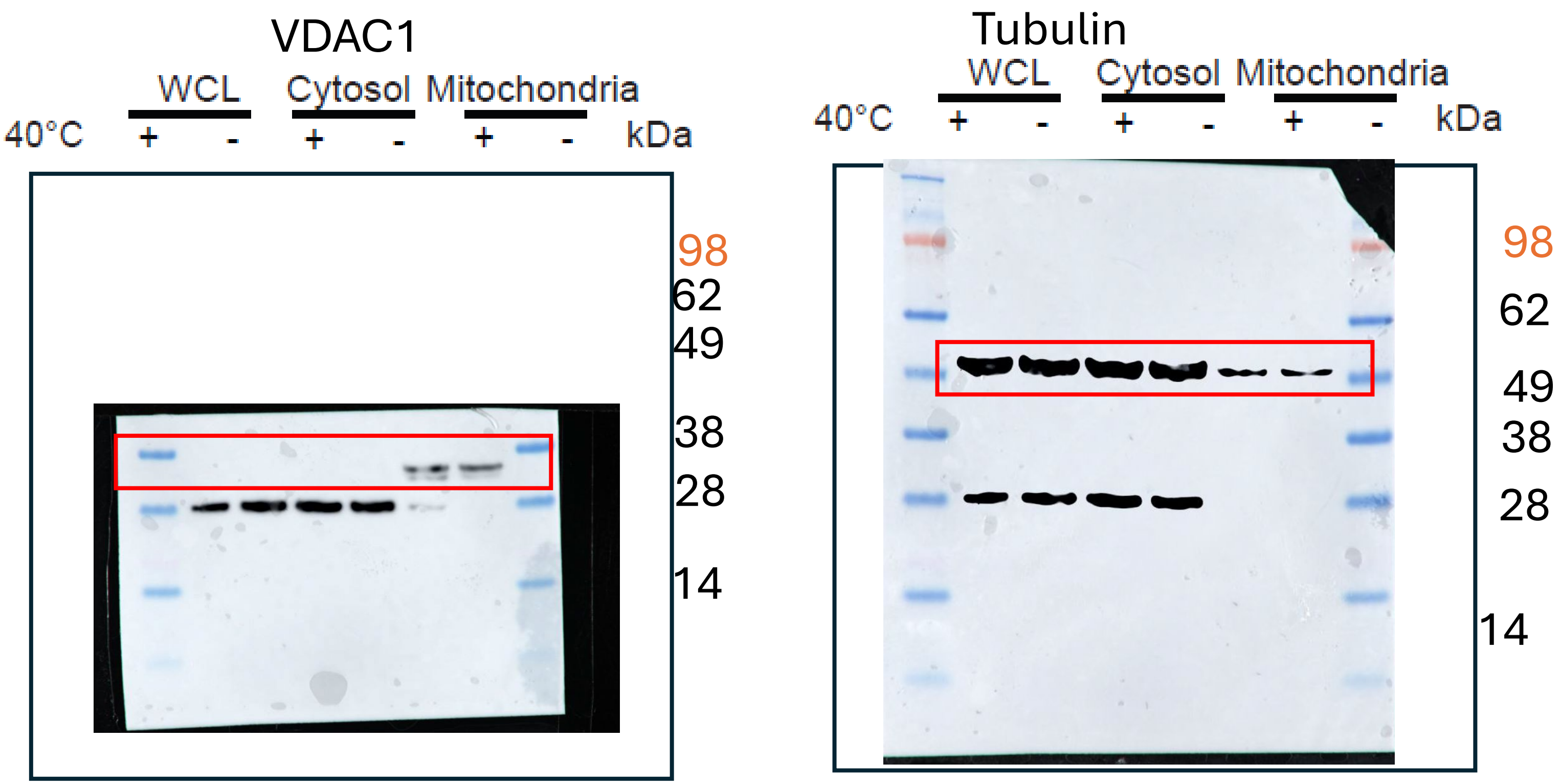

Bis-Tris 4-12% Gel
MES Buffer

Bis-Tris 4-12% Gel
MES Buffer

Figure Supplementary Figure 2:
HSPB translocation in response to mild heat stress
hiPSC-derived myotubes

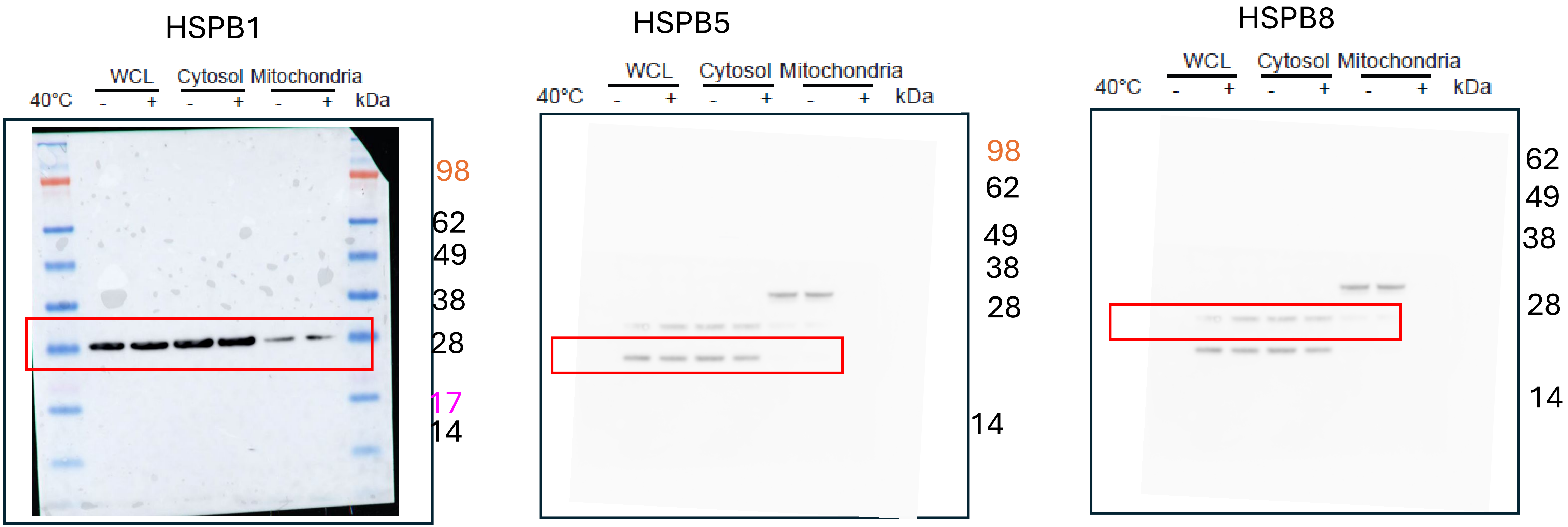

Bis-Tris 4-12% Gel
MES Buffer

Bis-Tris 4-12% Gel
MES Buffer

Bis-Tris 4-12% Gel
MES Buffer

Figure Supplementary Figure 2:
HSPB translocation in response to mild heat stress
hiPSC-derived myotubes

VDAC1

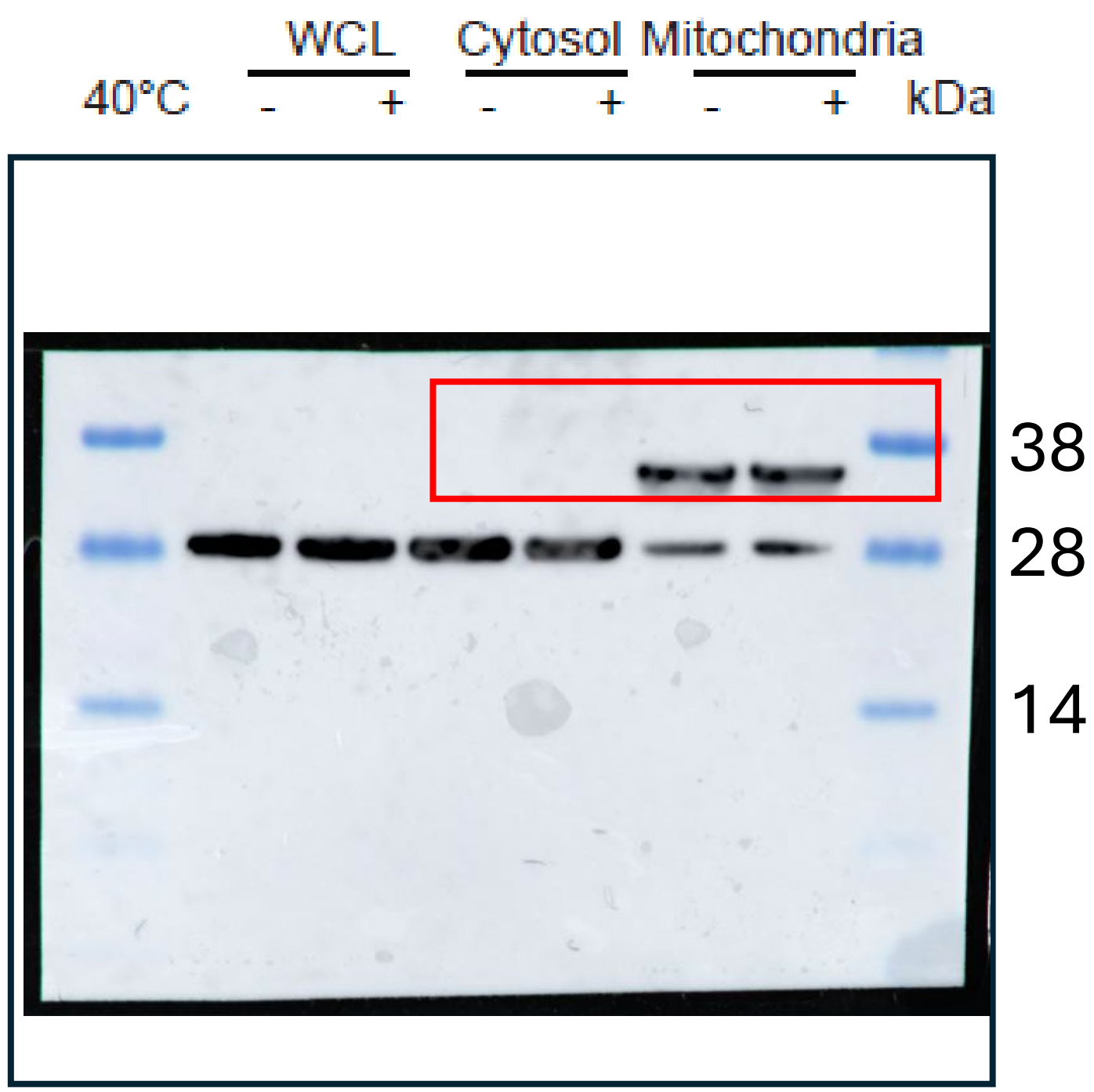

Bis-Tris 4-12% Gel
MES Buffer

Marker blot for HSPB5
(overexposed)

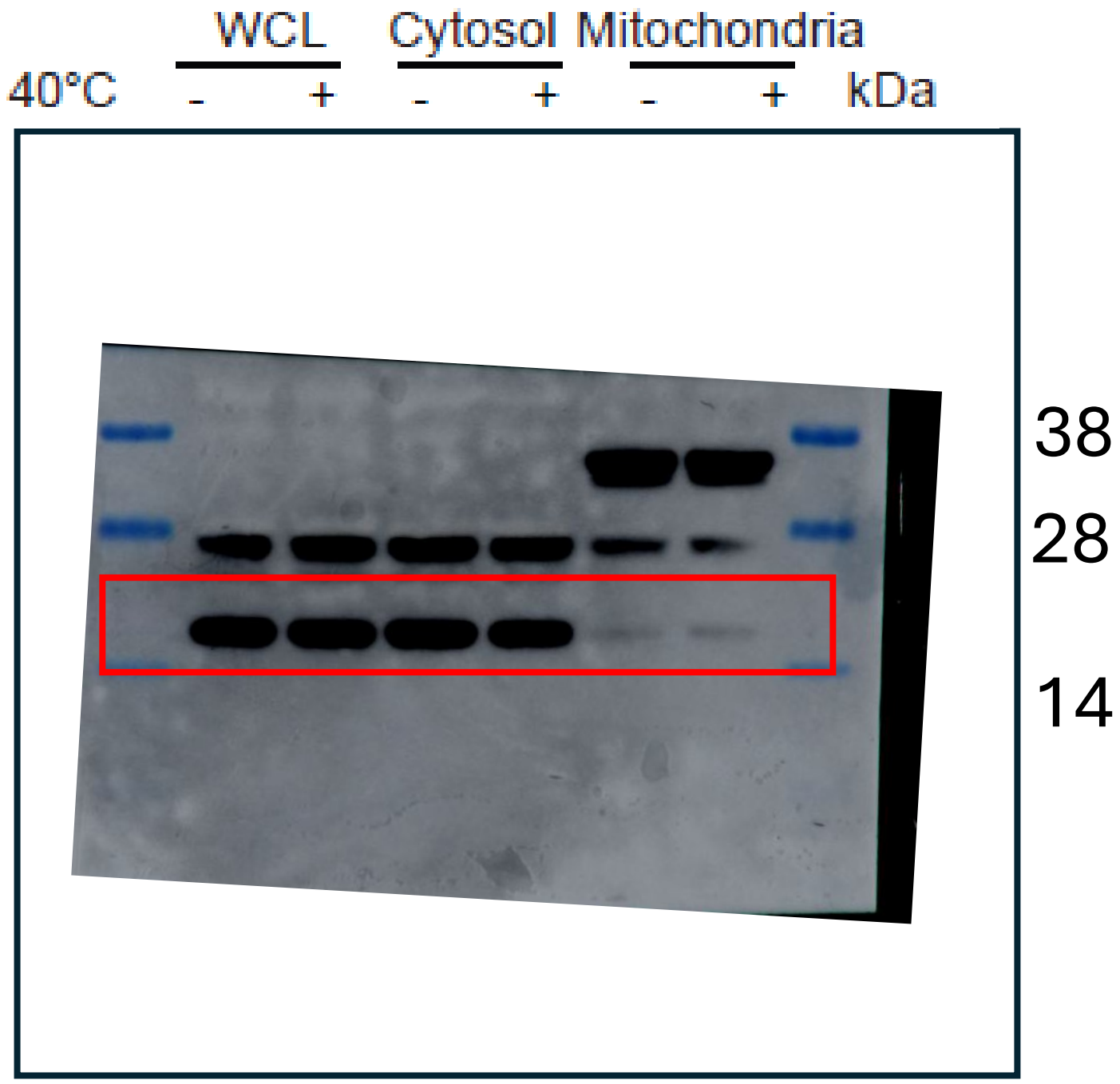

Bis-Tris 4-12% Gel
MES Buffer

Marker blot for HSPB8
(Overexposed)

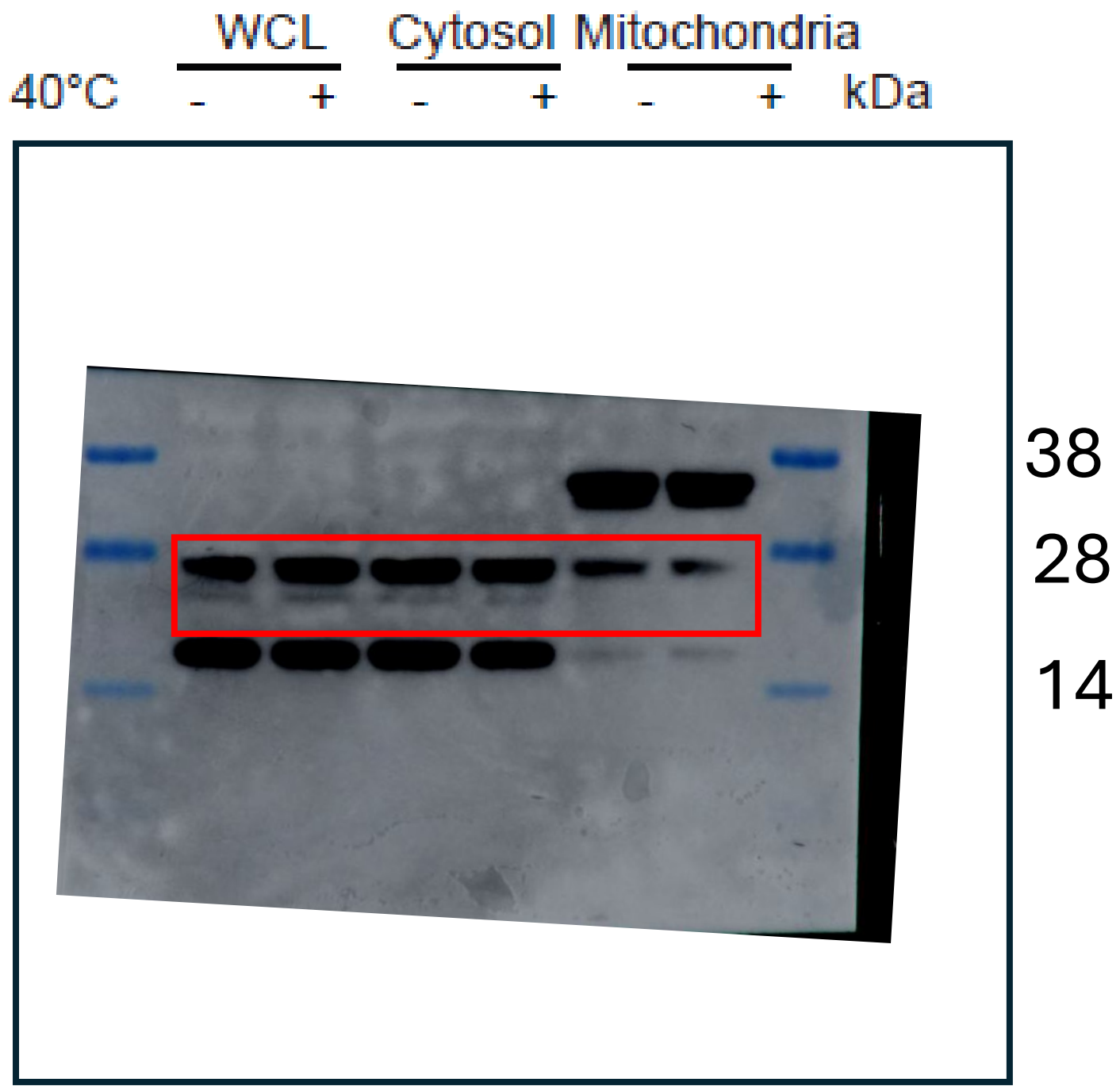

Bis-Tris 4-12% Gel
MES Buffer

Figure Supplementary Figure 2:
HSPB translocation in response to mild heat stress
hiPSC-derived myotubes

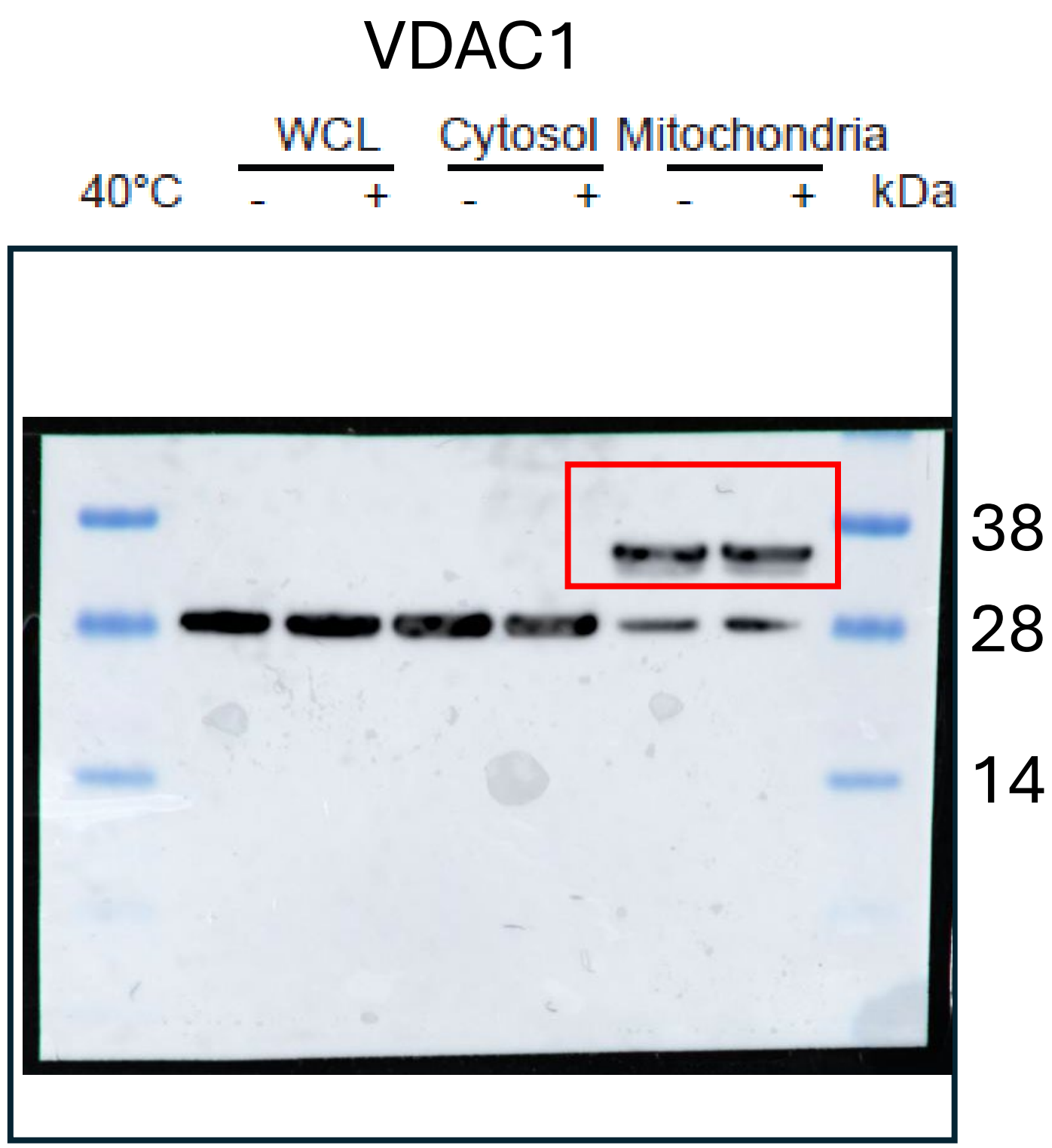

Bis-Tris 4-12% Gel
MES Buffer

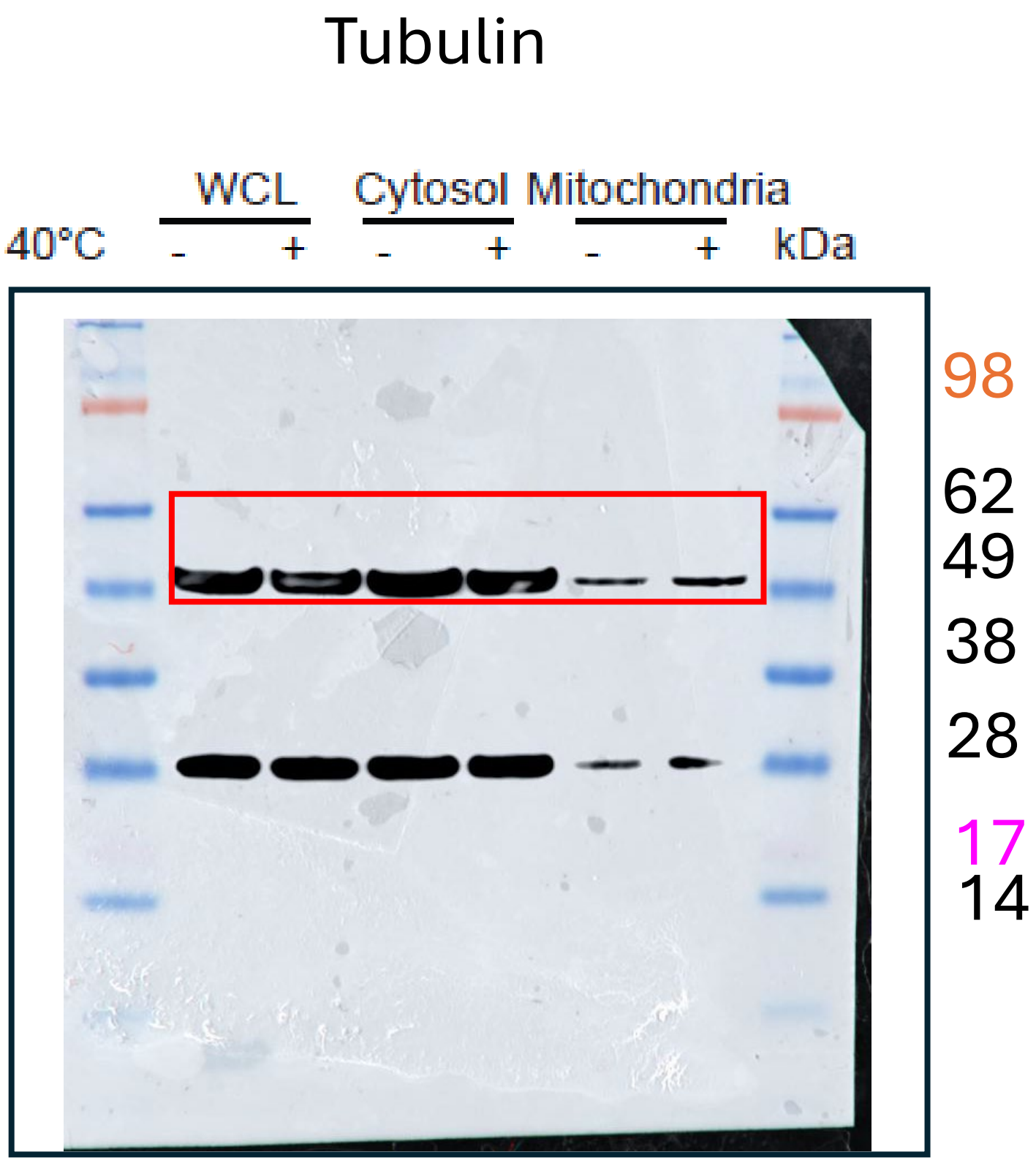

Bis-Tris 4-12% Gel
MES Buffer

Figure Supplementary Figure 2:
HSPB translocation in response to mild heat stress
N2a Neuroblasts

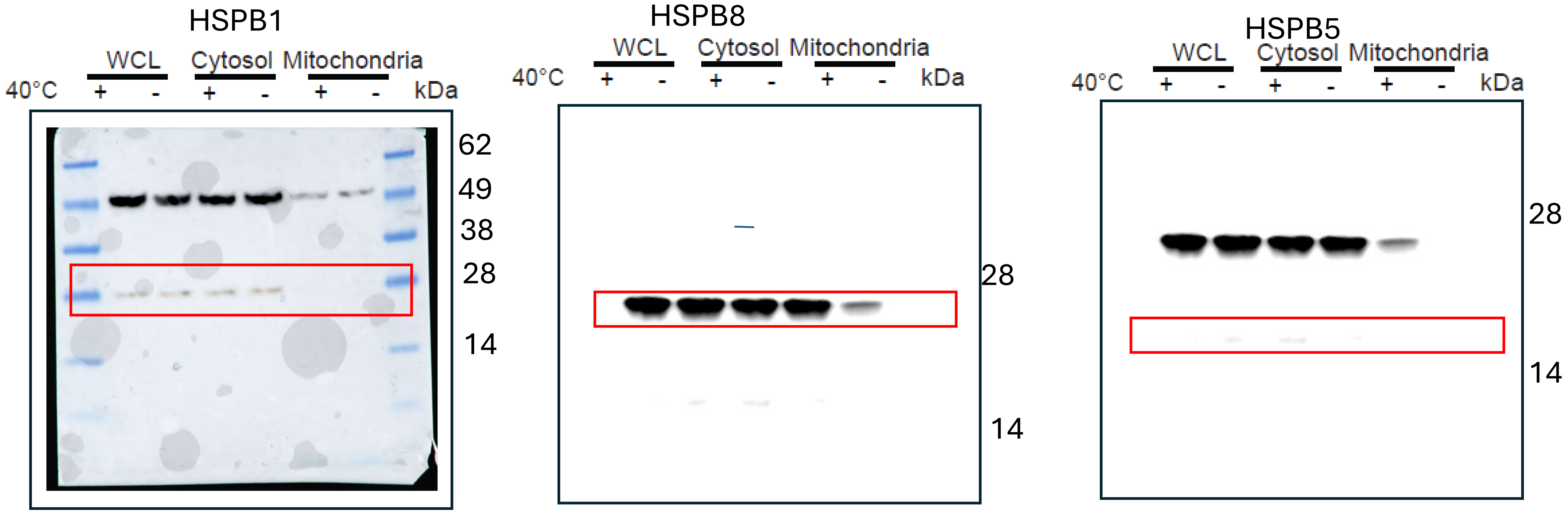

Bis-Tris 4-12% Gel
MES Buffer

Bis-Tris 4-12% Gel
MES Buffer

Bis-Tris 4-12% Gel
MES Buffer

Figure Supplementary Figure 2:
HSPB translocation in response to mild heat stress
N2a Neuroblasts

HSPB5 & 8 overexposed with marker

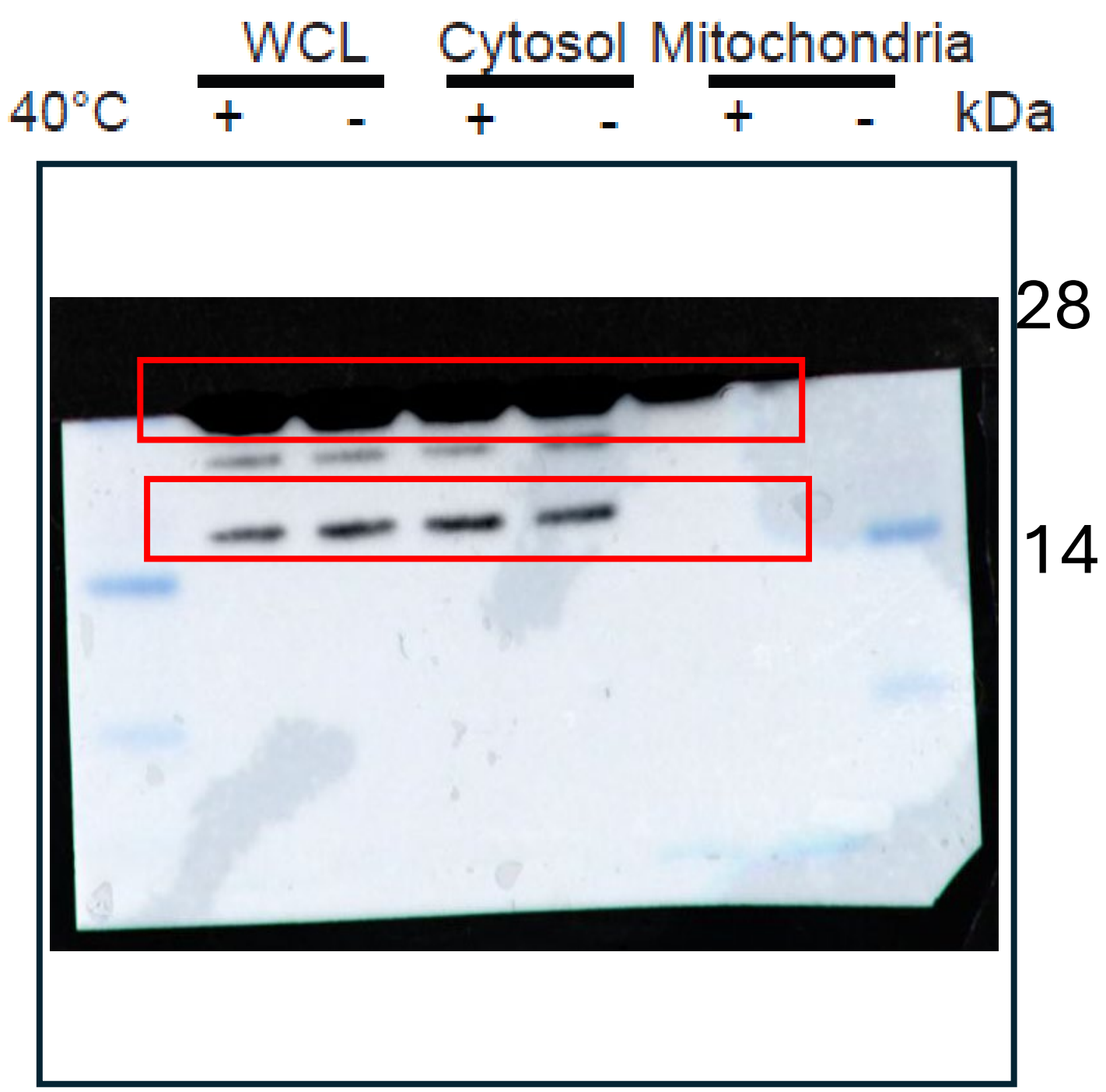

Bis-Tris 4-12% Gel
MES Buffer

VDAC1

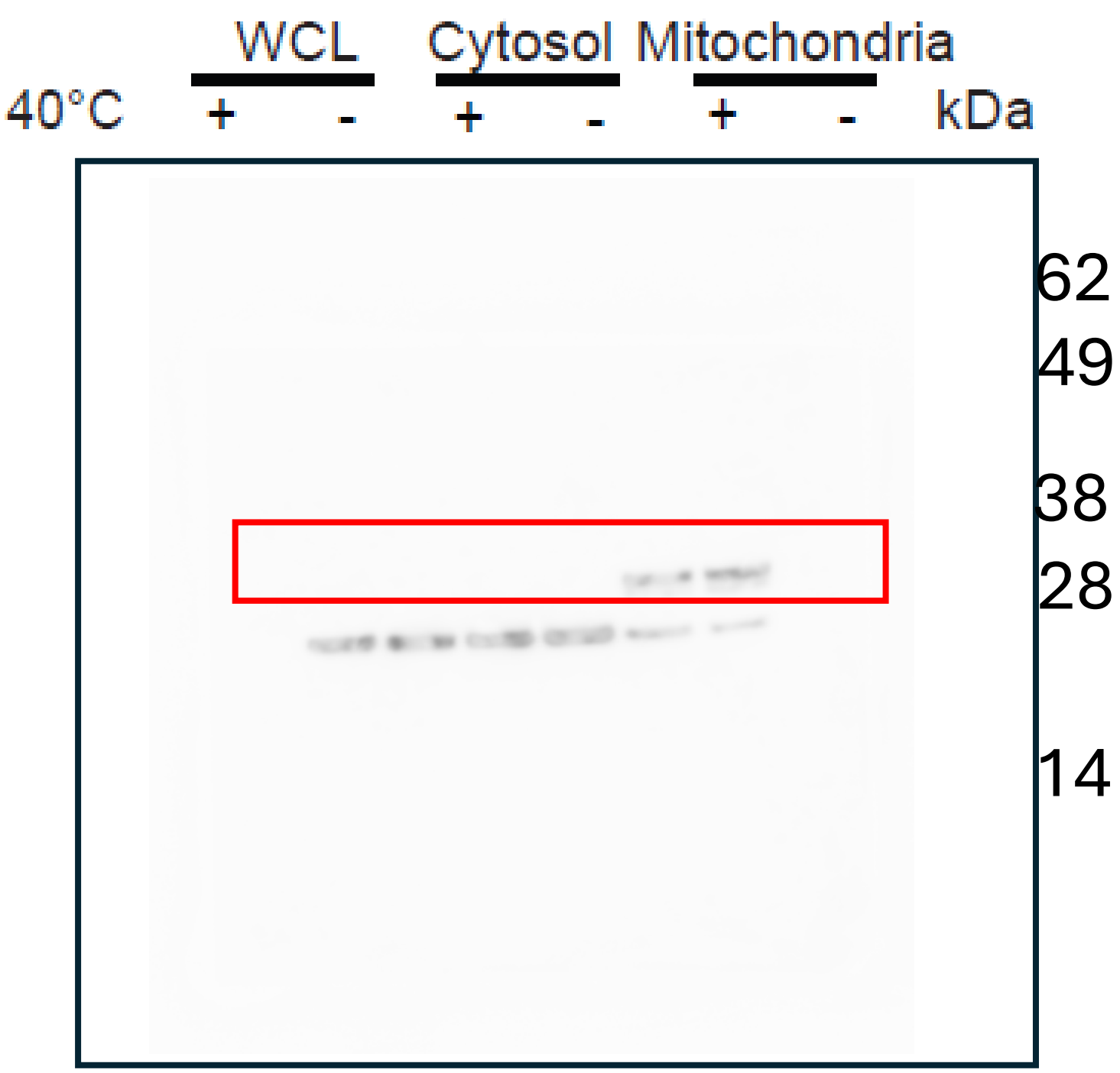

Bis-Tris 4-12% Gel
MES Buffer

VDAC1 Marker

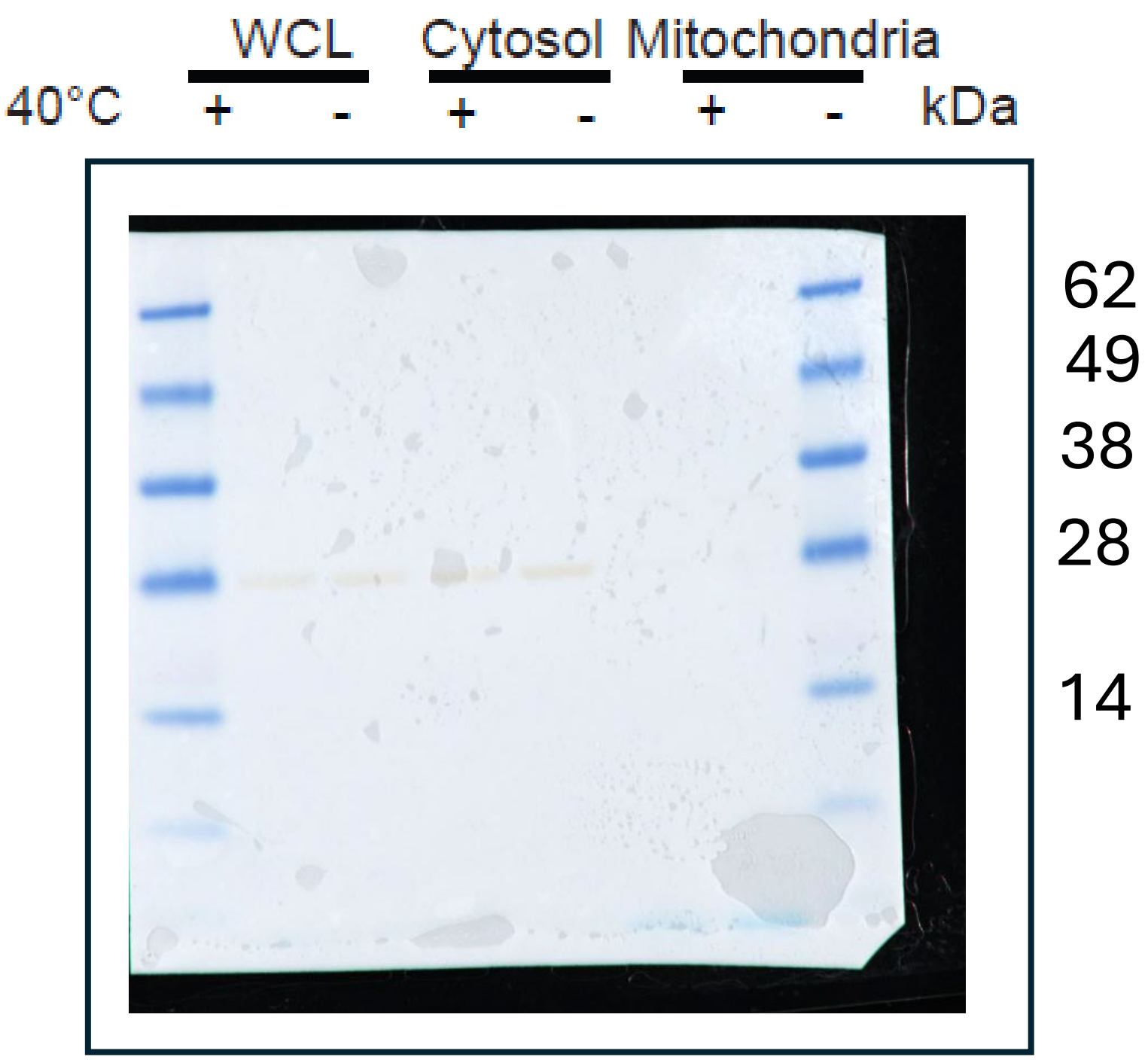

Bis-Tris 4-12% Gel
MES Buffer

Figure Supplementary Figure 2:
HSPB translocation in response to mild heat stress
N2a Neuroblasts

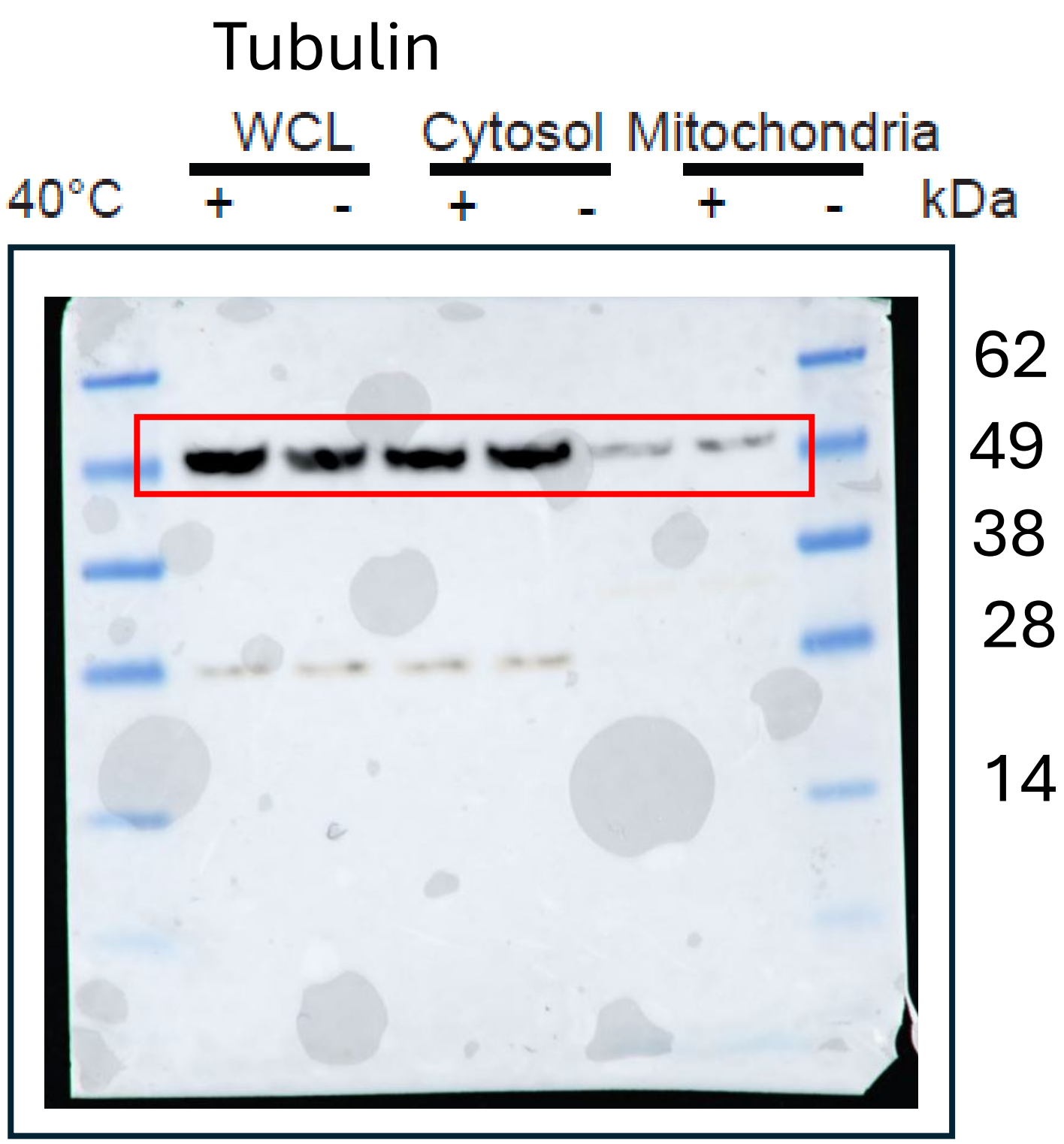

Bis-Tris 4-12% Gel
MES Buffer

Figure Supplementary Figure 3:
Co-IP (Rep1)

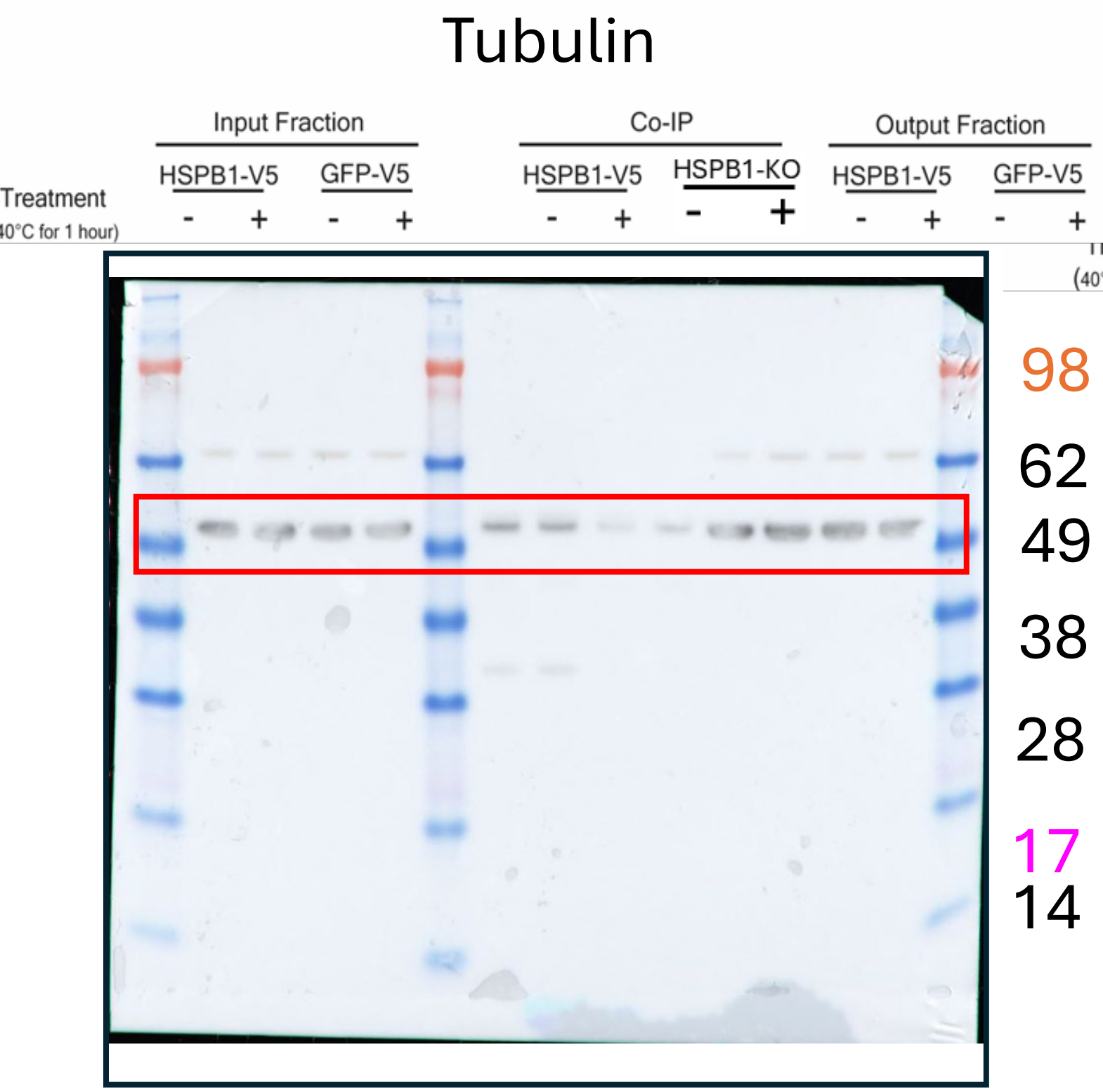

Bis-Tris 4-12% Gel
MES Buffer

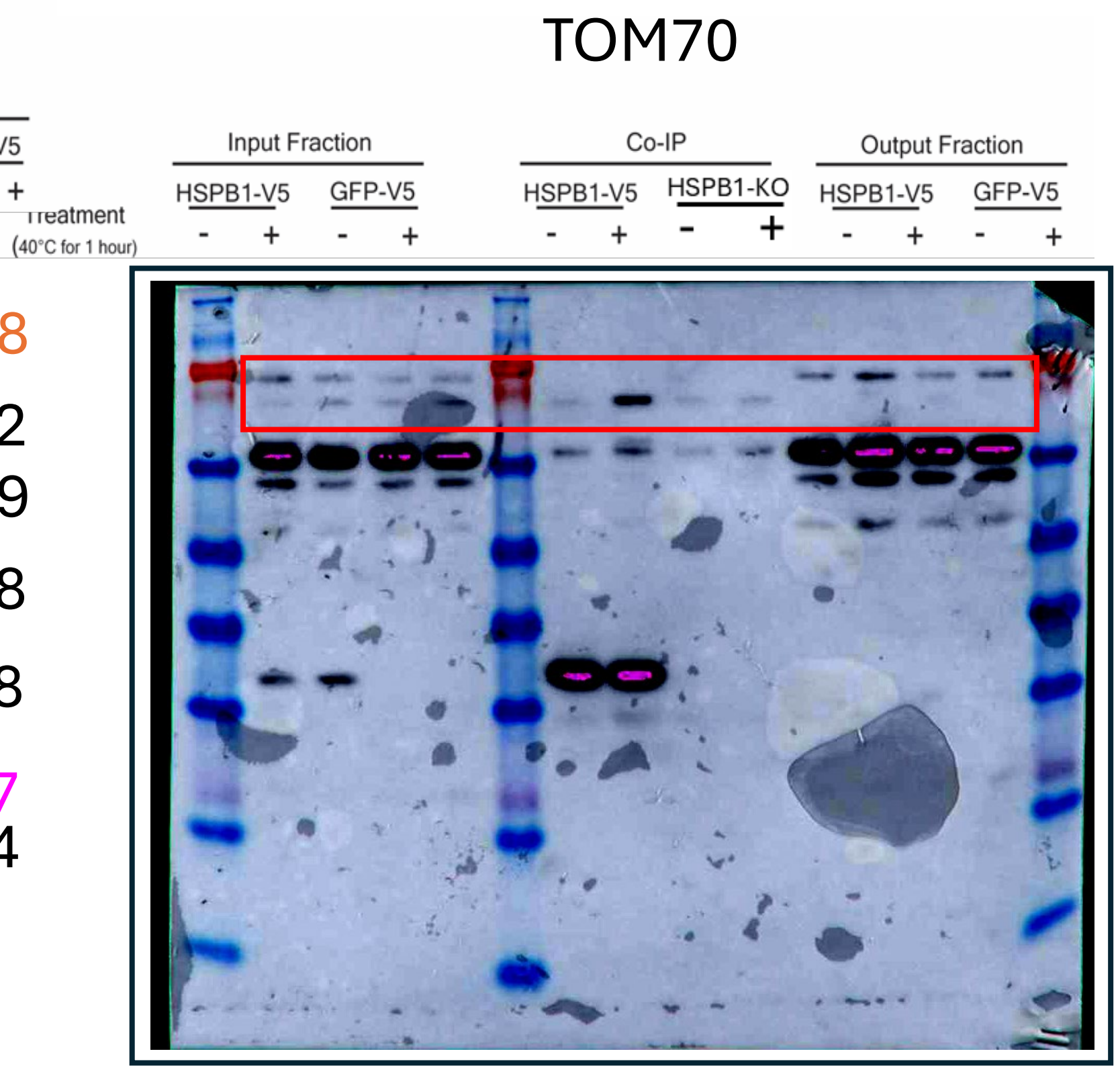

Bis-Tris 4-12% Gel
MES Buffer

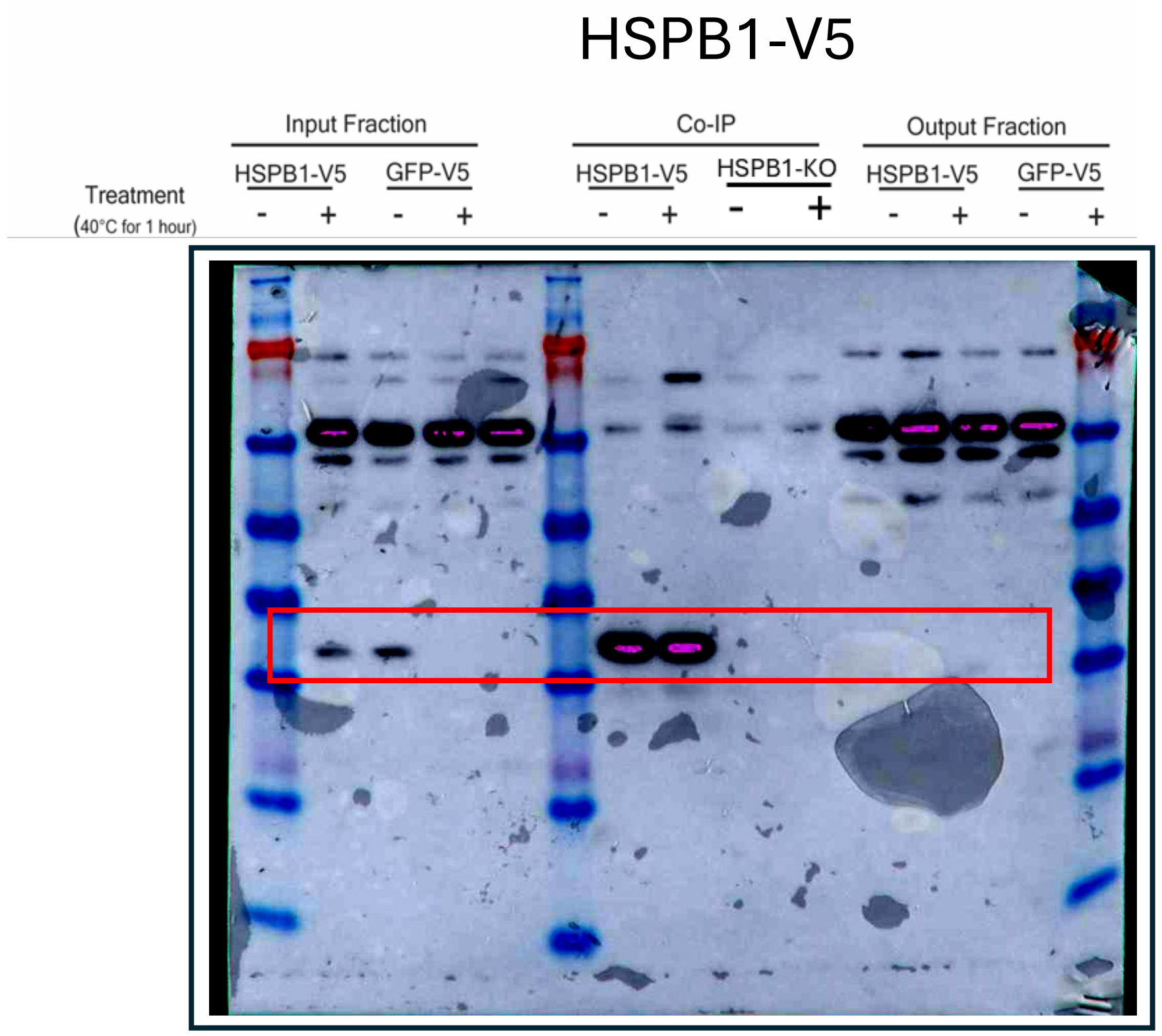

Bis-Tris 4-12% Gel
MES Buffer

Figure Supplementary Figure 3:
Co-IP (Rep 2)

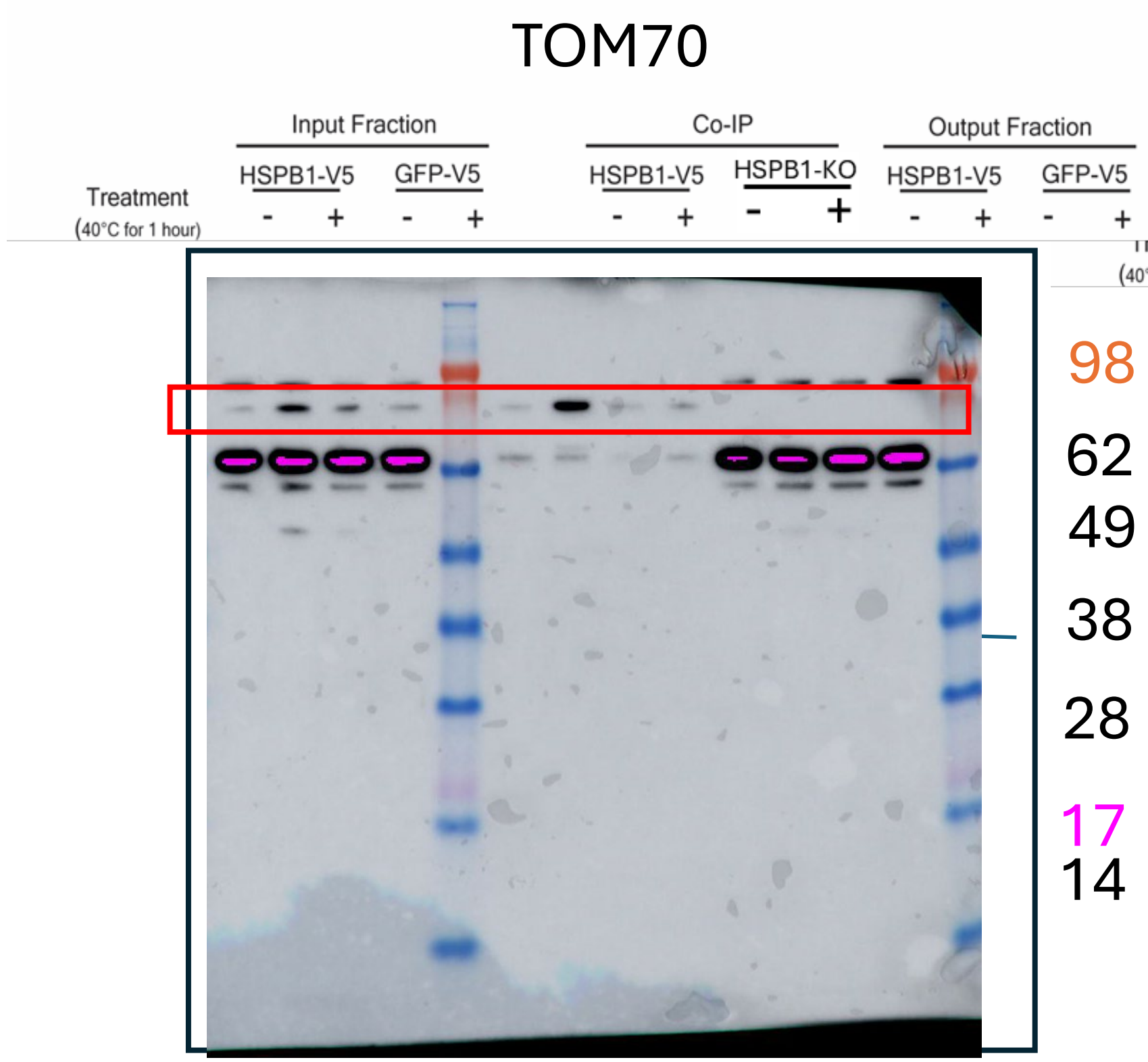

Bis-Tris 4-12% Gel
MES Buffer

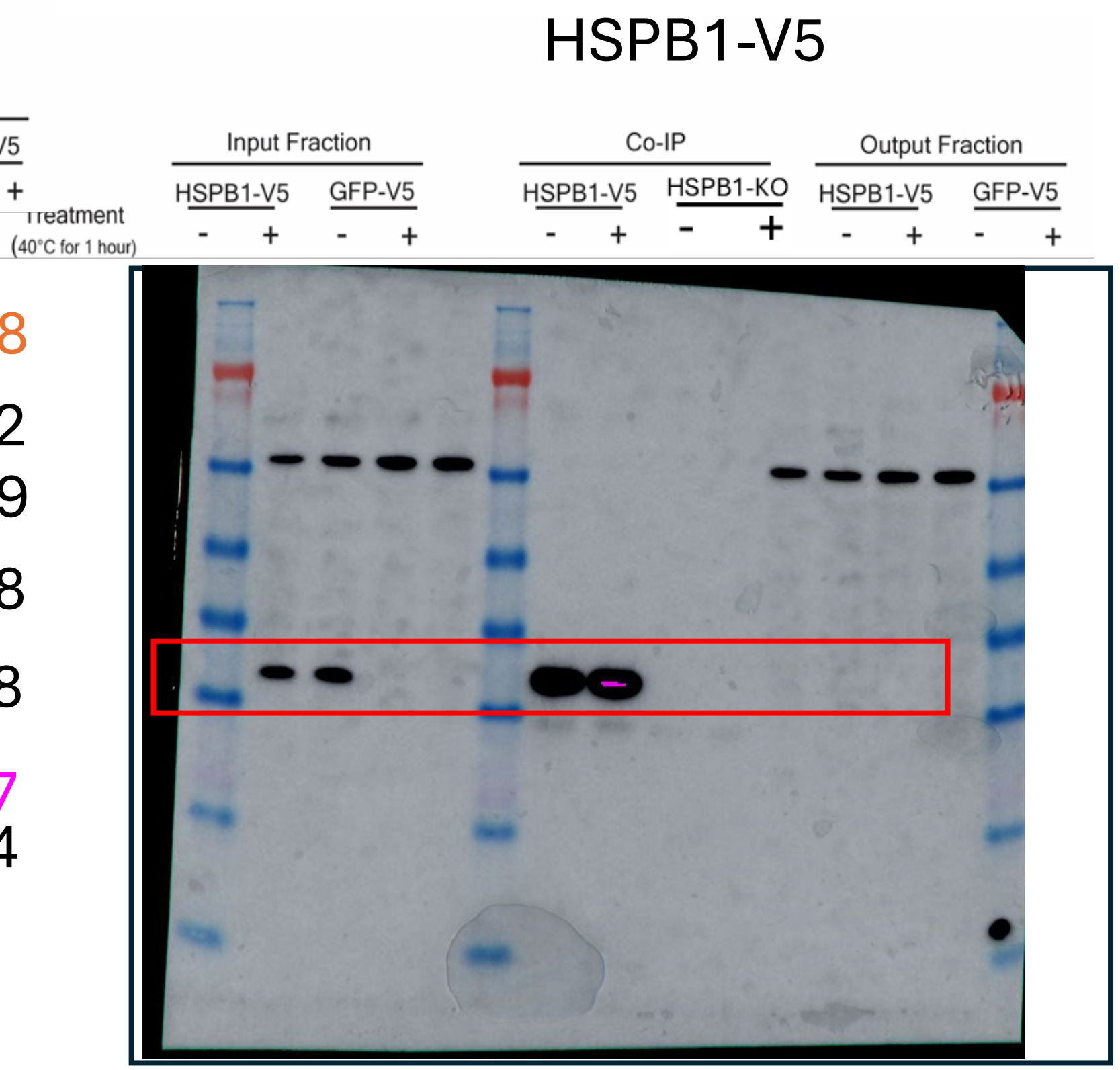

Bis-Tris 4-12% Gel
MES Buffer

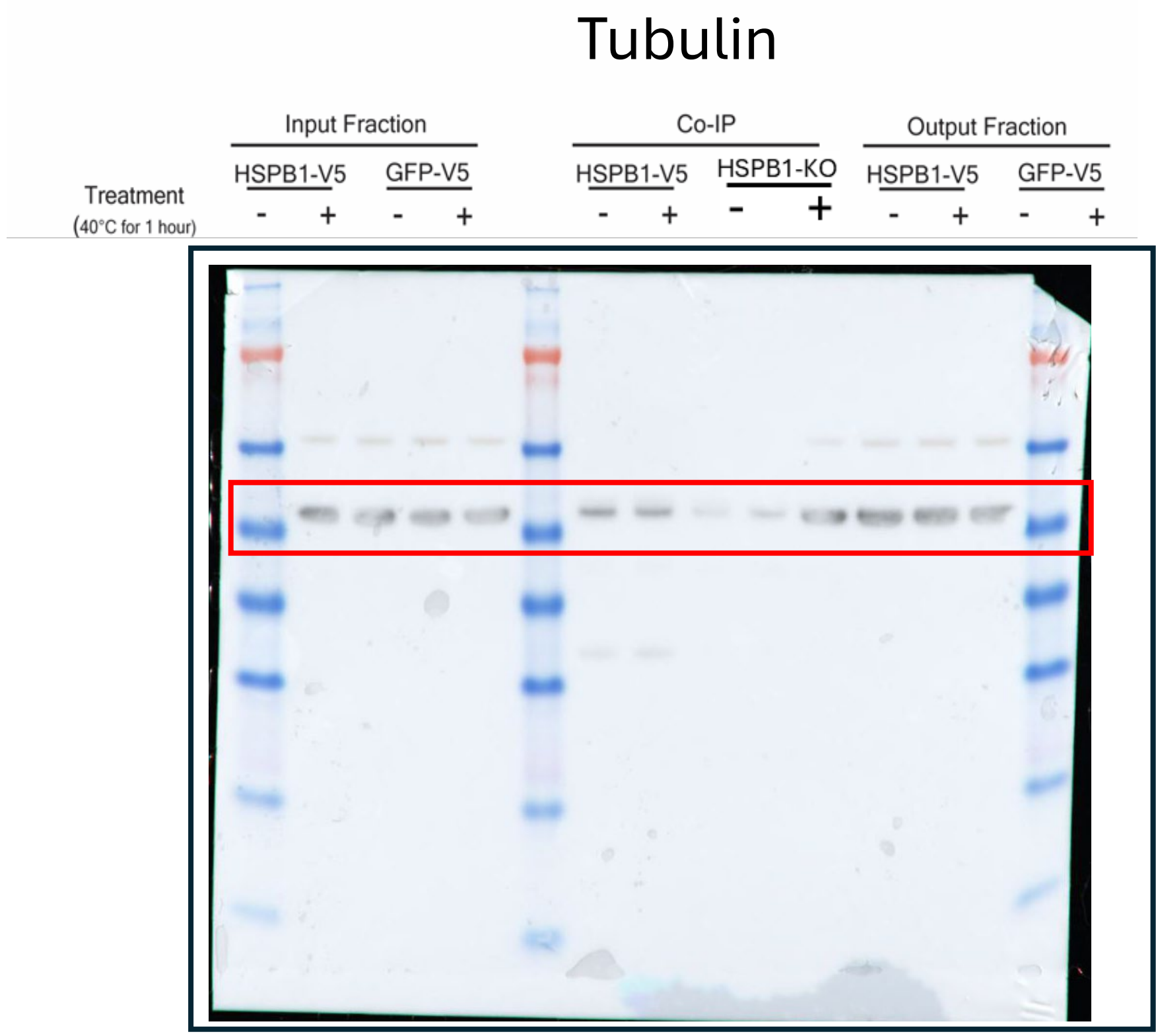

Bis-Tris 4-12% Gel
MES Buffer

Figure Supplementary Figure :
Co-IP (Rep 3)

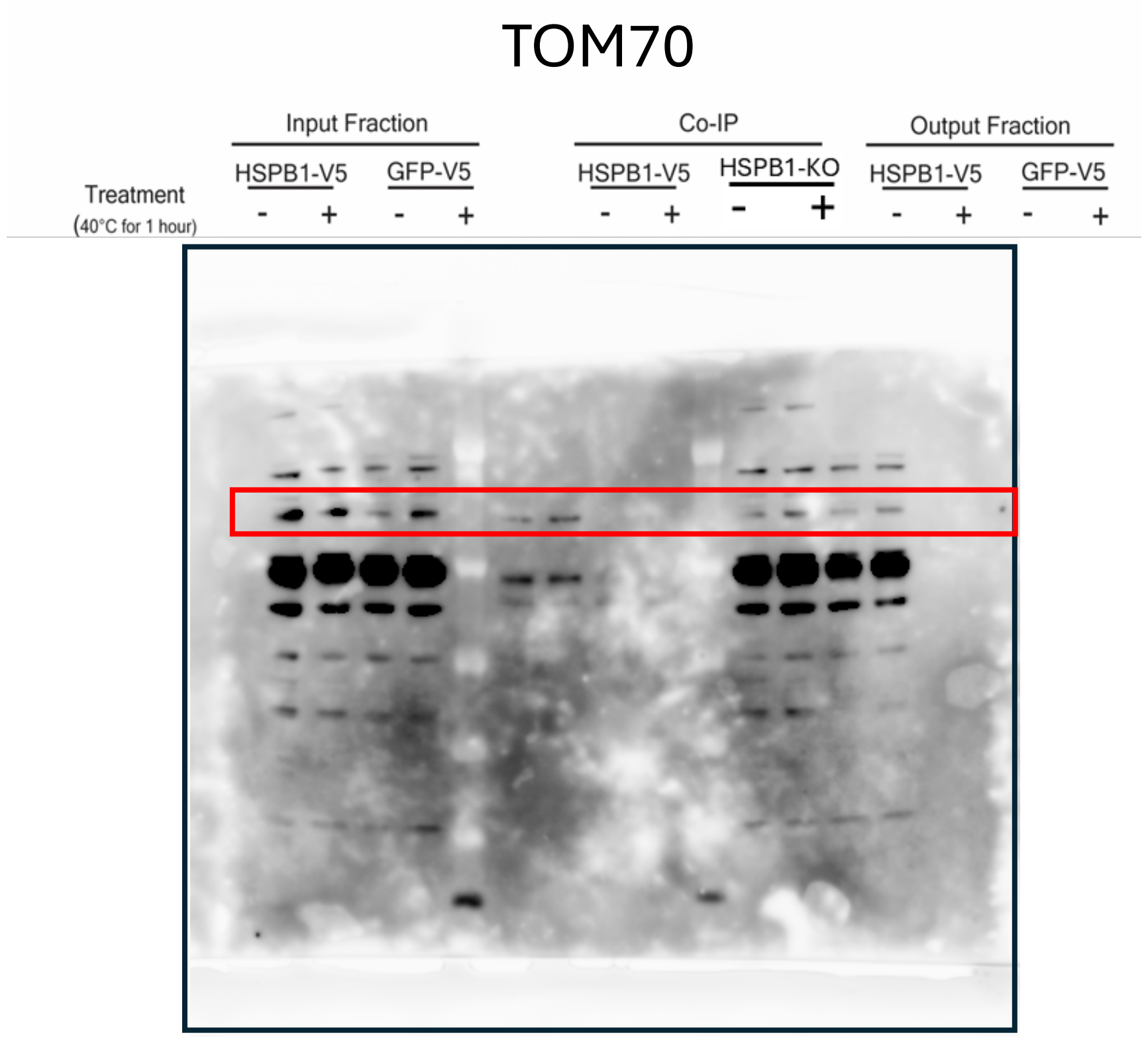

Bis-Tris 4-12% Gel
MES Buffer

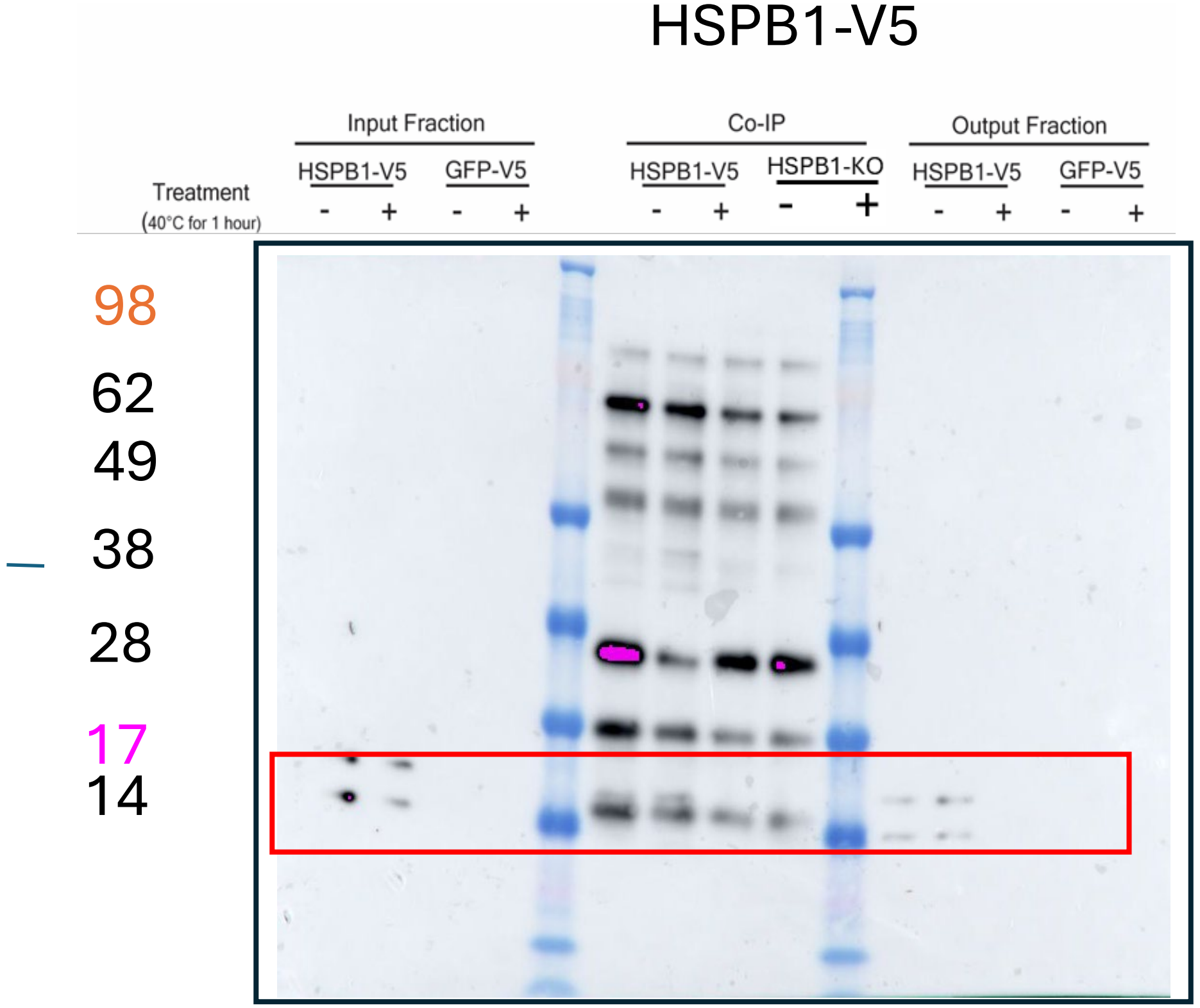

Bis-Tris 4-12% Gel
MES Buffer

Supplementary Figure S7

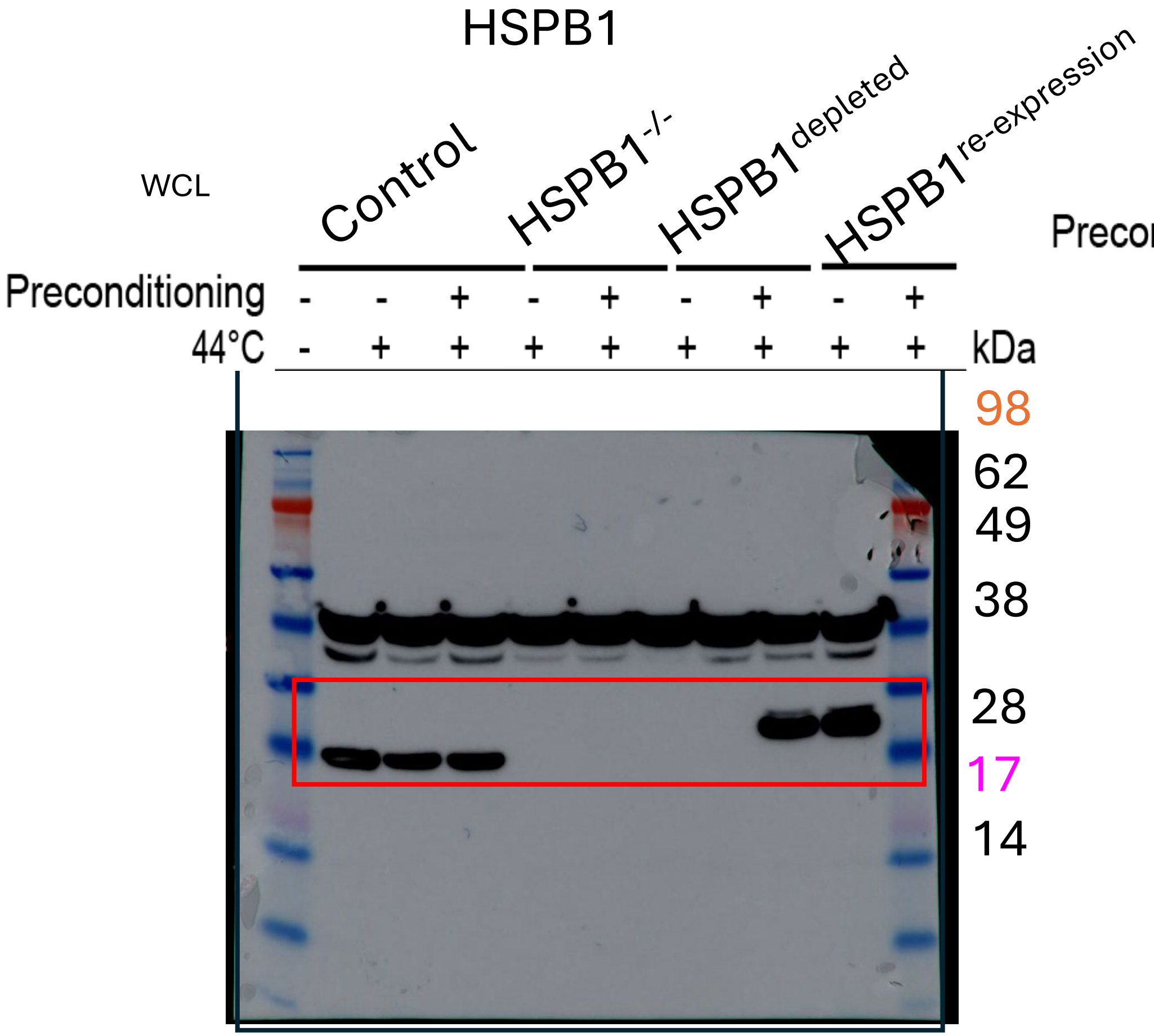

Bis-Tris 4-12% Gel
MES Buffer

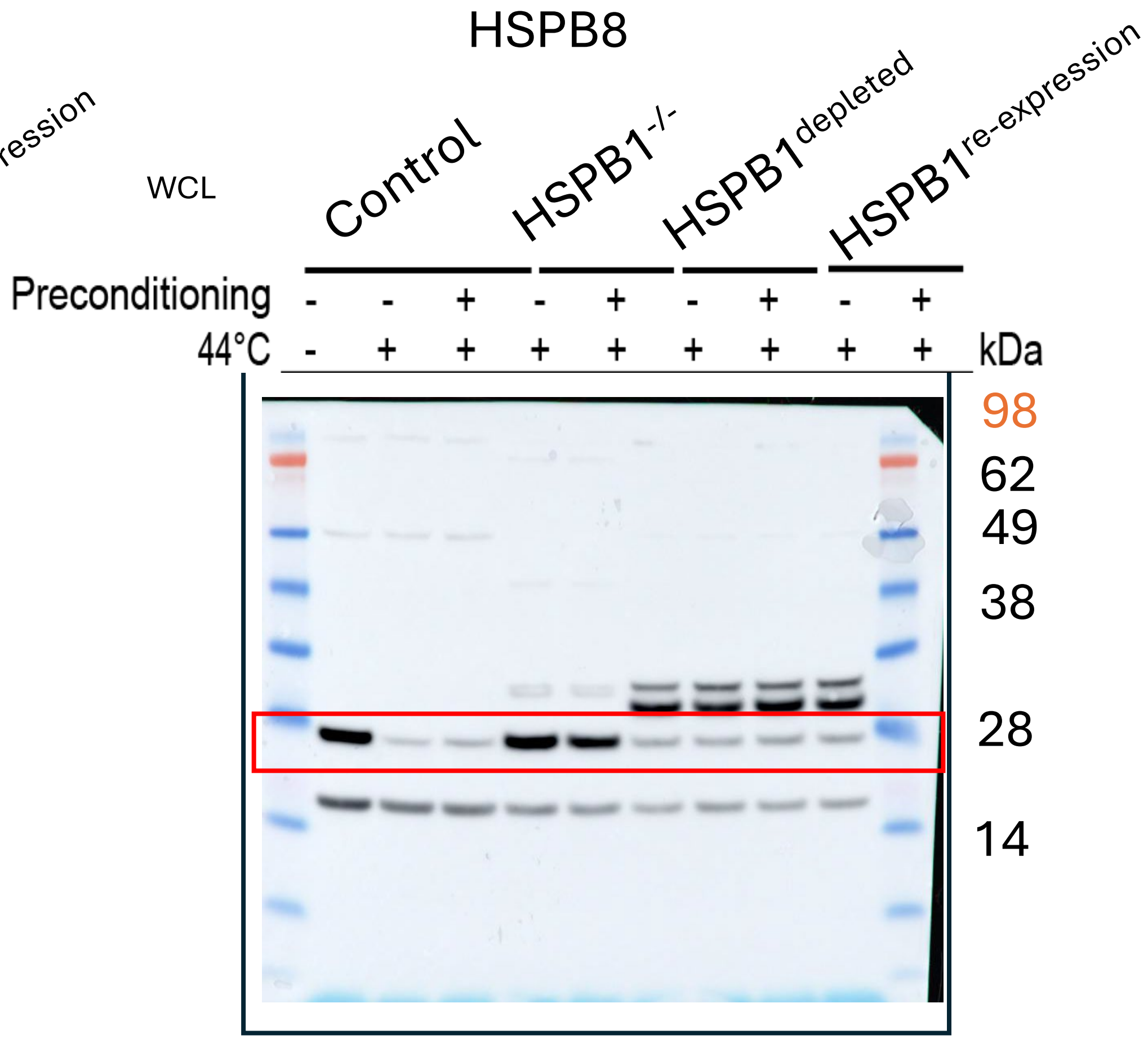

Bis-Tris 4-12% Gel
MES Buffer

Supplementary Figure S7

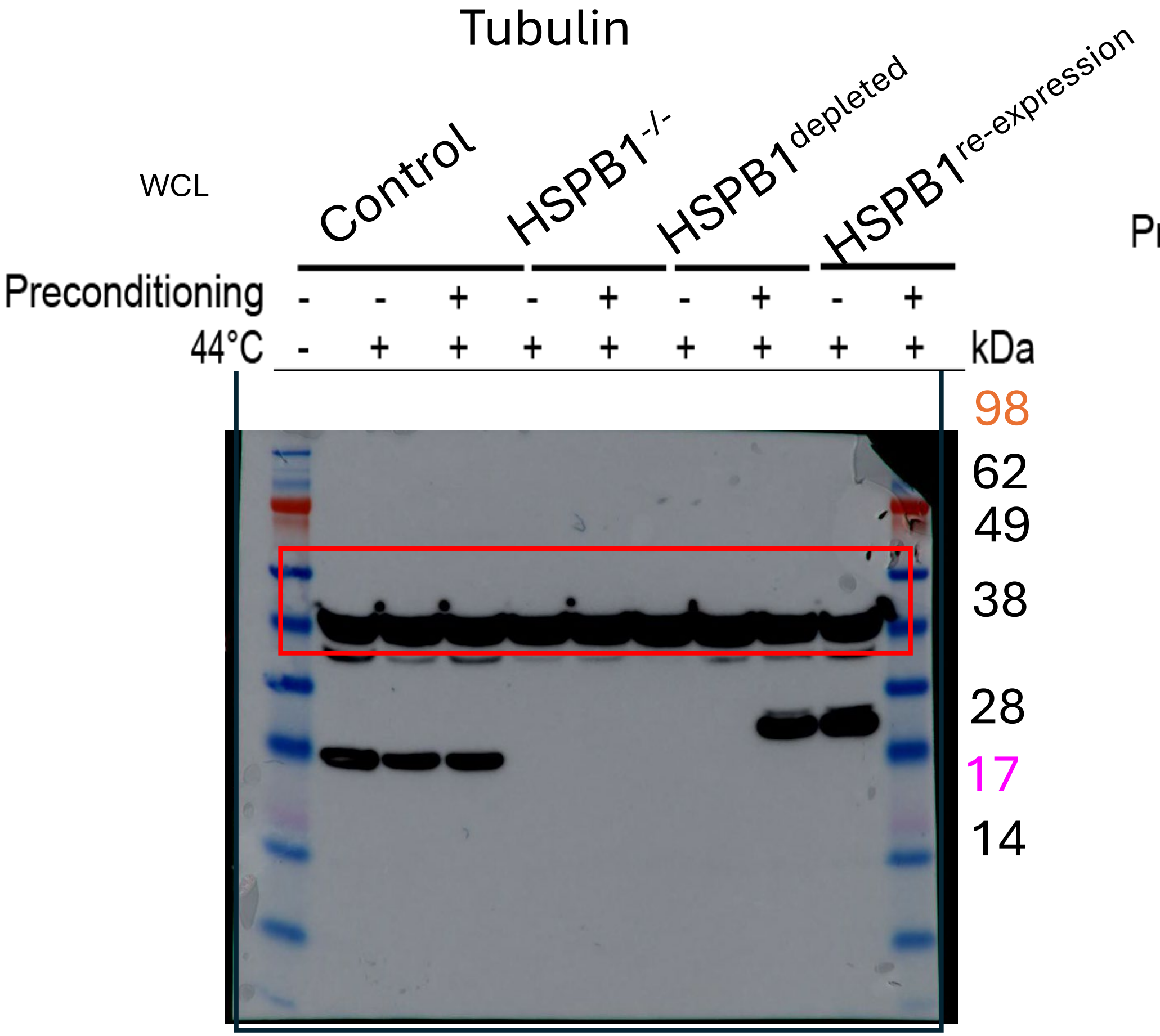

Bis-Tris 4-12% Gel
MES Buffer

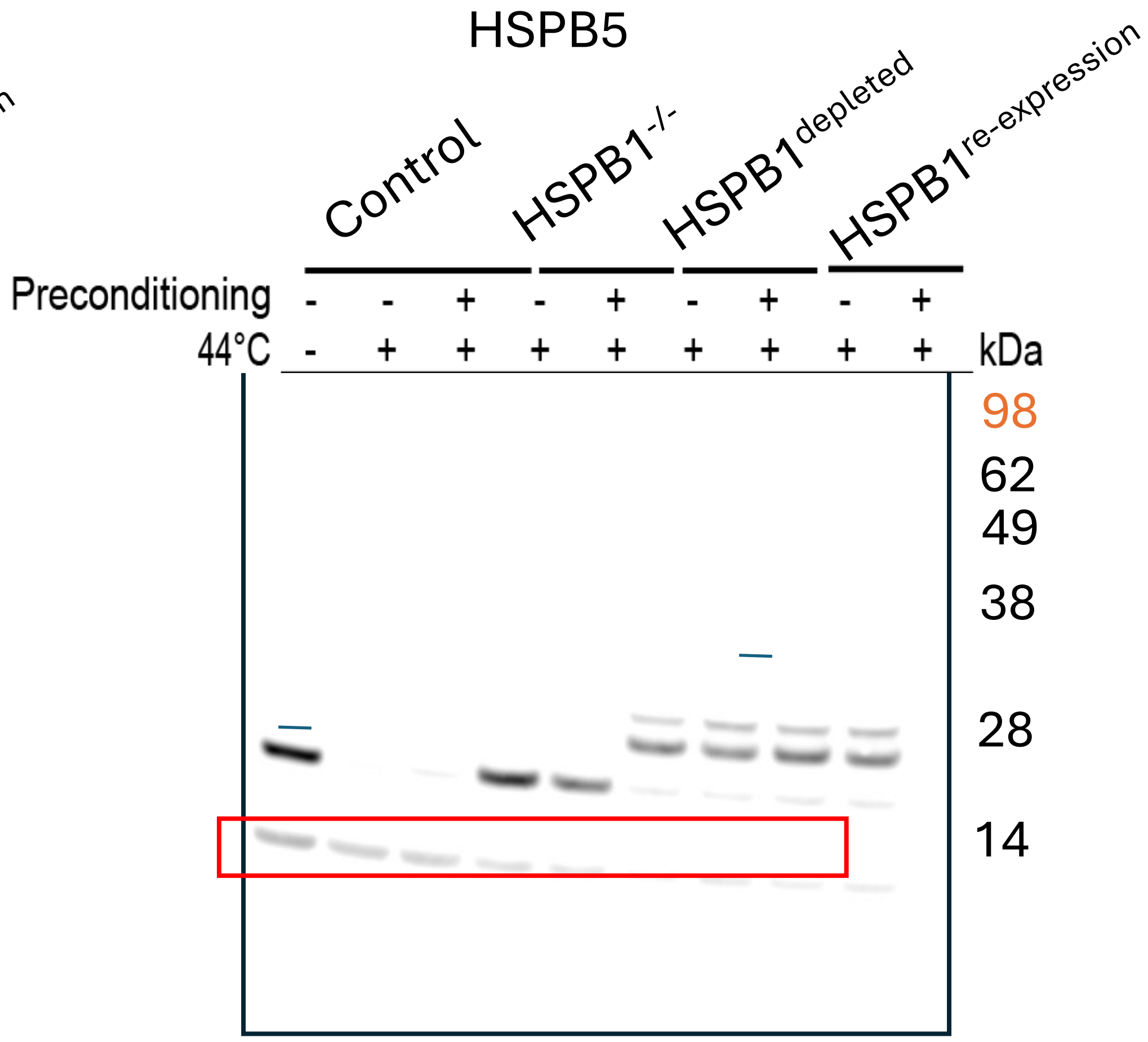

Bis-Tris 4-12% Gel
MES Buffer

Supplementary Figure S7

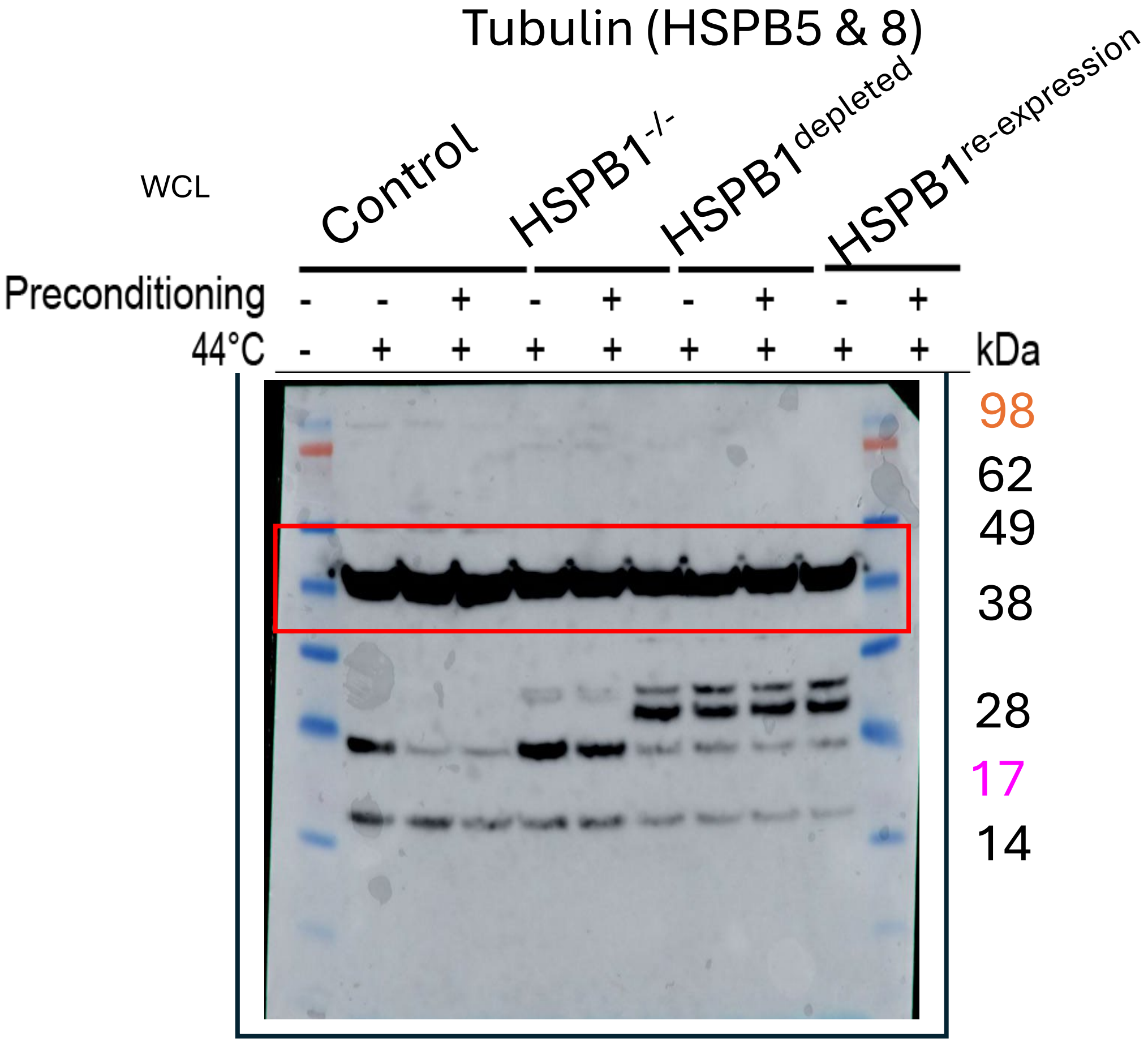

Bis-Tris 4-12% Gel
MES Buffer

Supplementary Figure S7

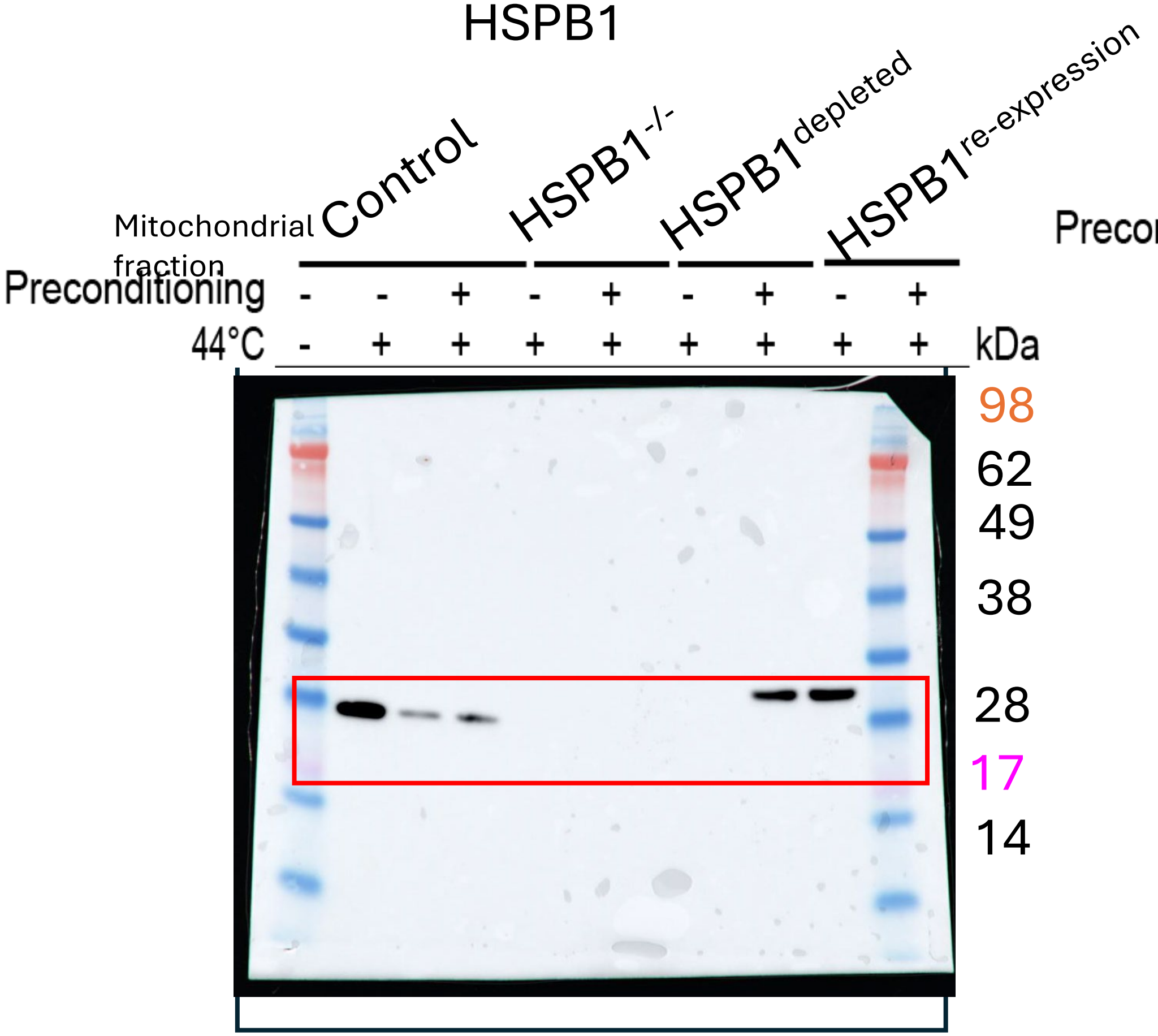

Bis-Tris 4-12% Gel
MES Buffer

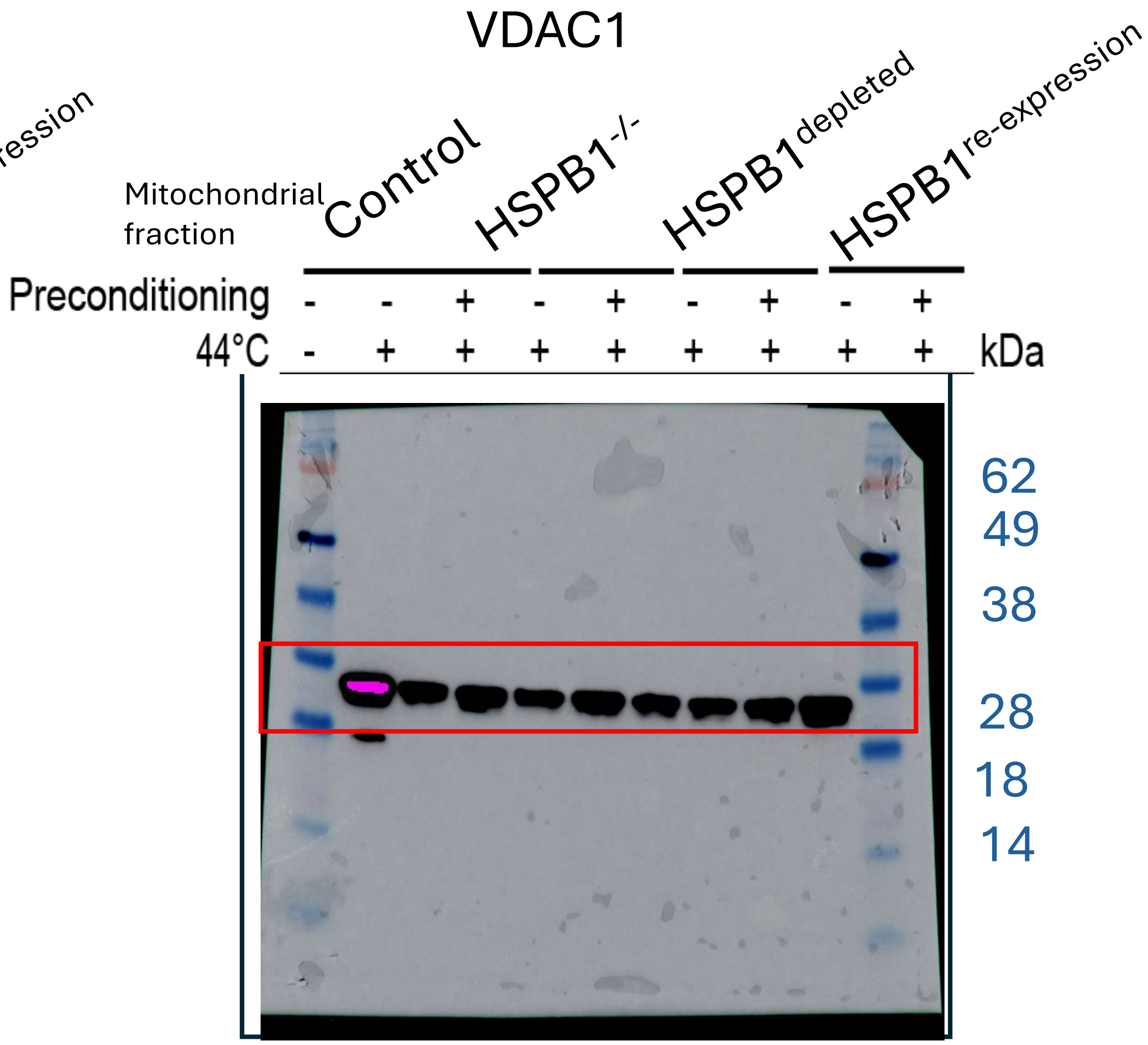

Bis-Tris 4-12% Gel
MES Buffer

Supplementary Figure S7

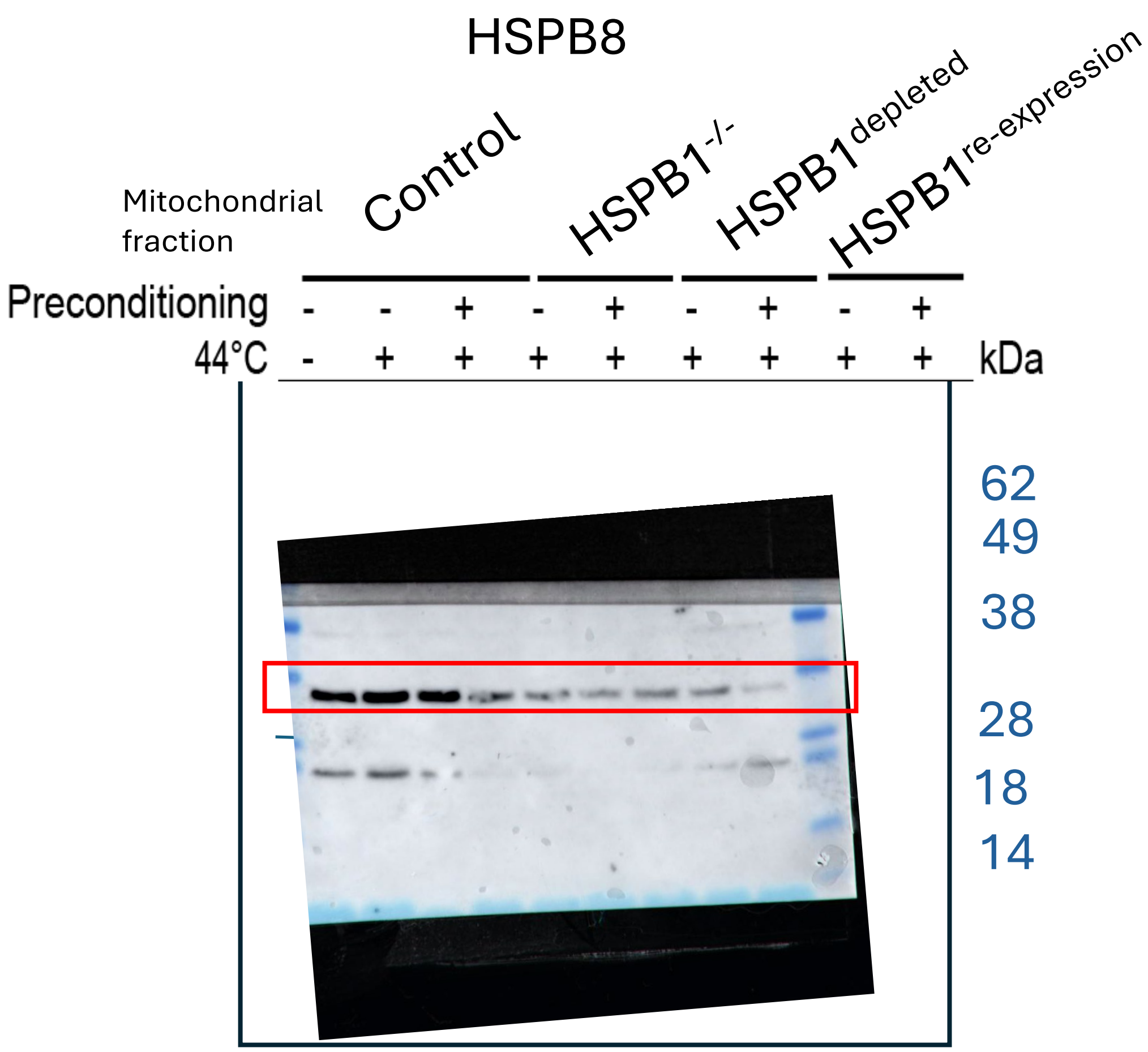

Bis-Tris 4-12% Gel
MES Buffer

Supplementary Figure S7

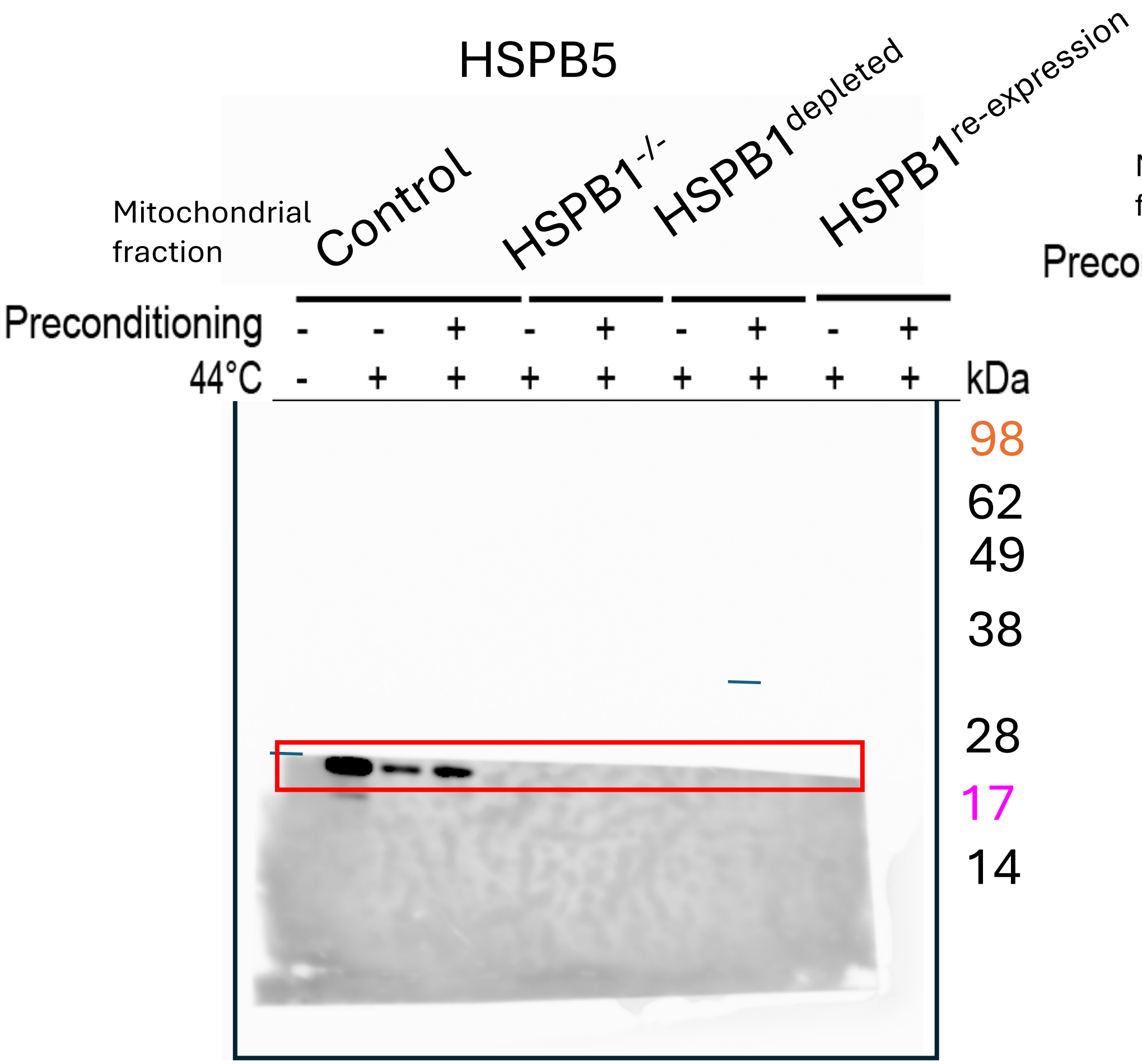

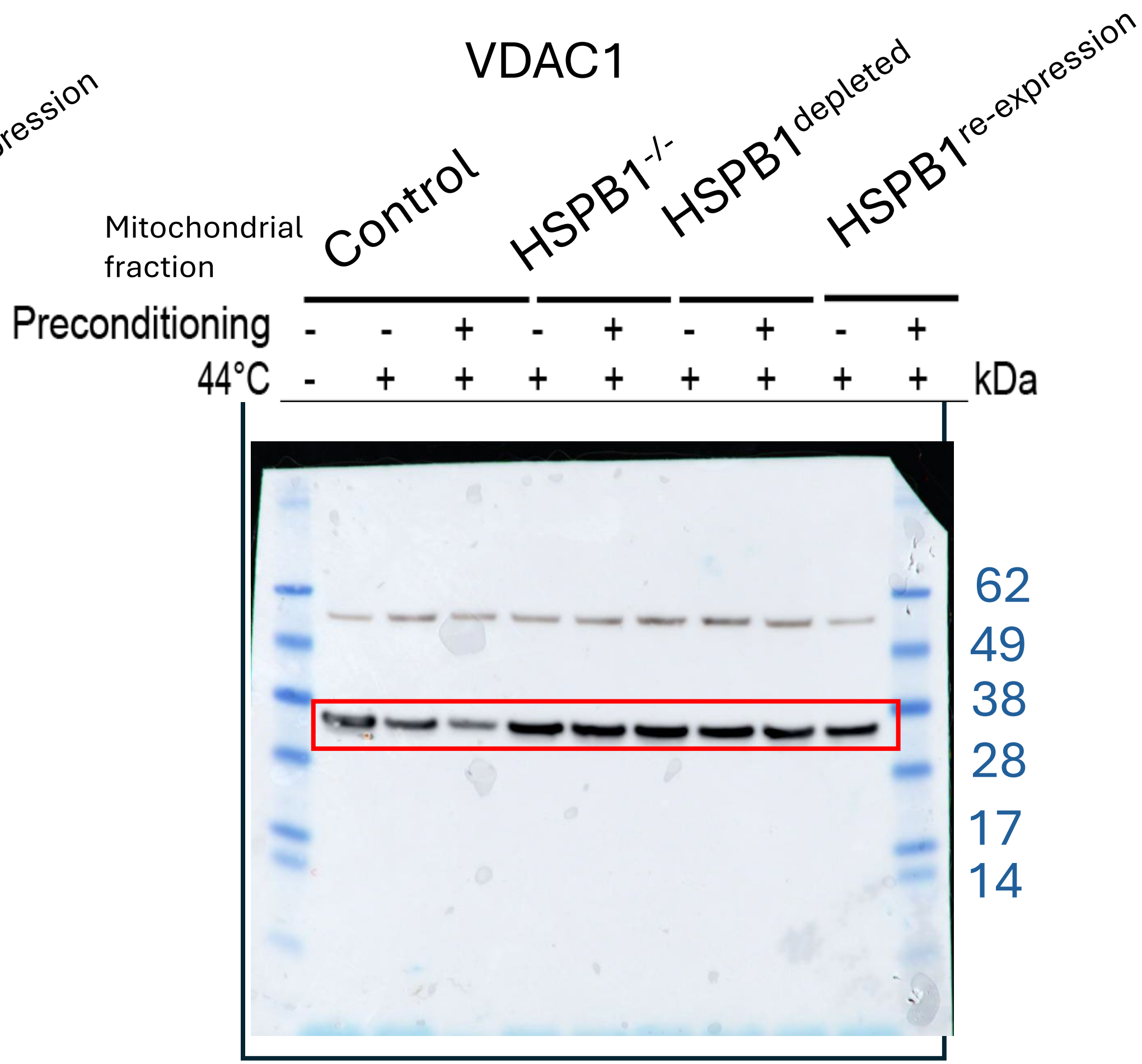

Supplementary Figure S8

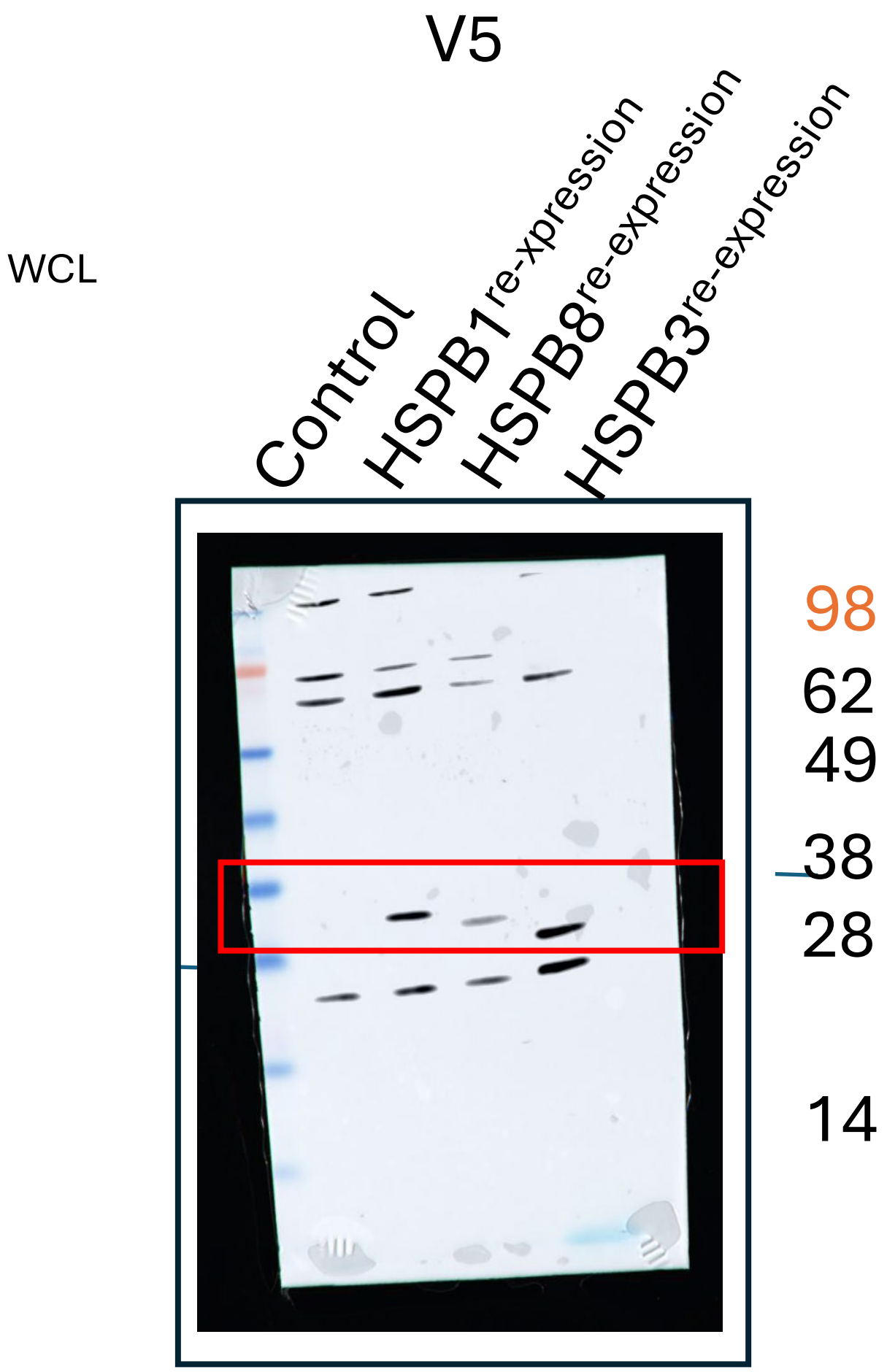

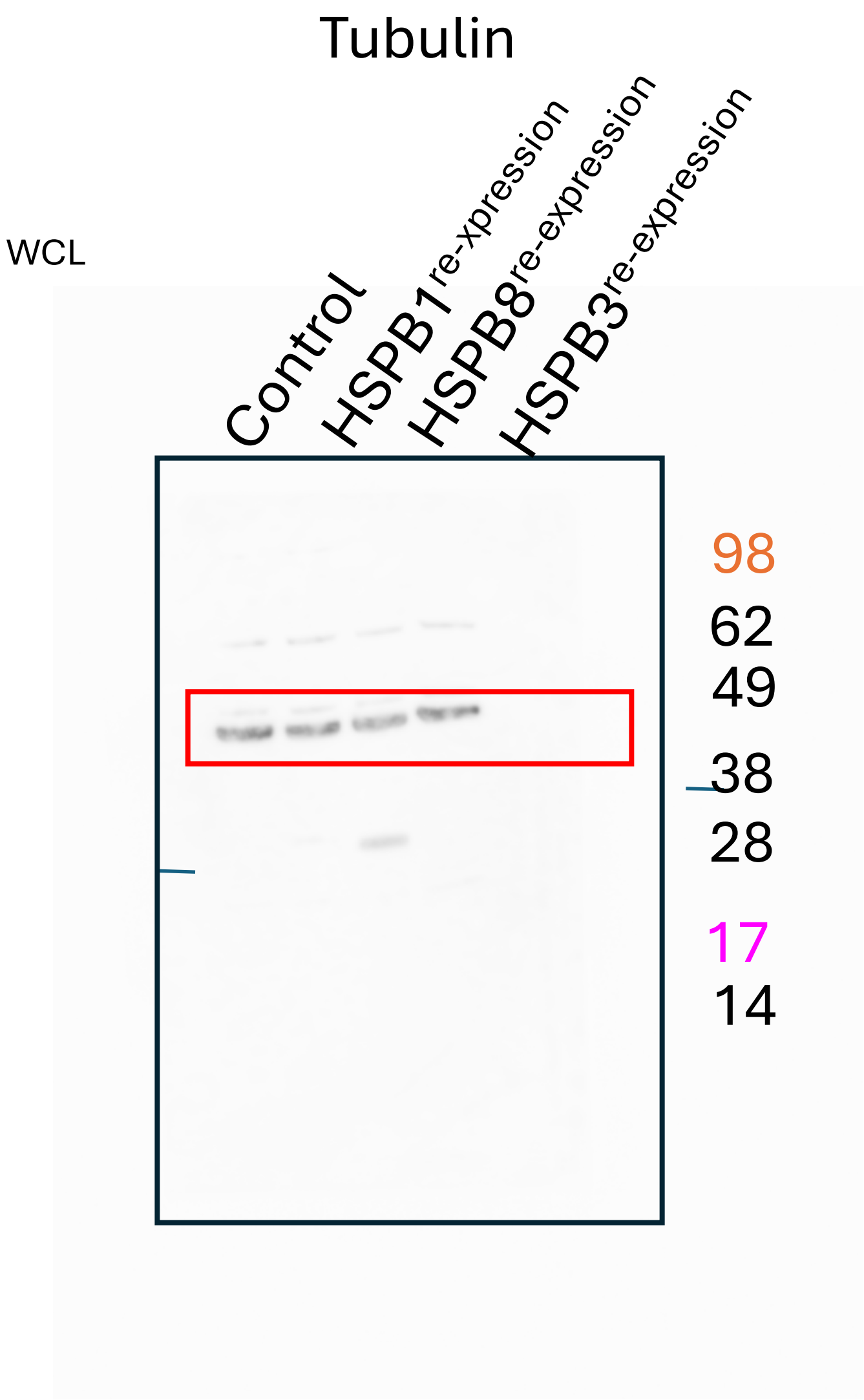

Supplementary Figure S8

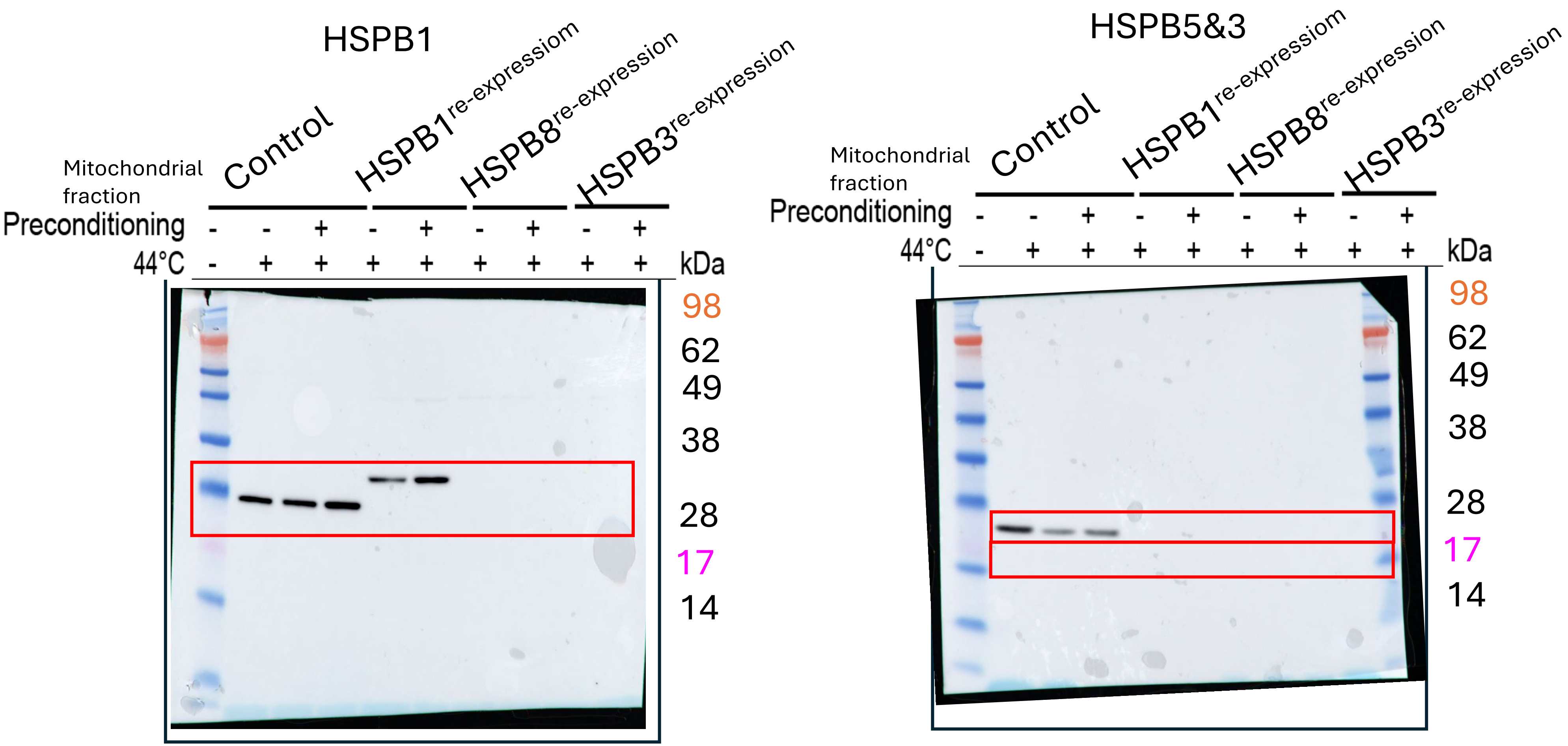

Bis-Tris 4-12% Gel
MES Buffer

Bis-Tris 4-12% Gel
MES Buffer

Supplementary Figure S8

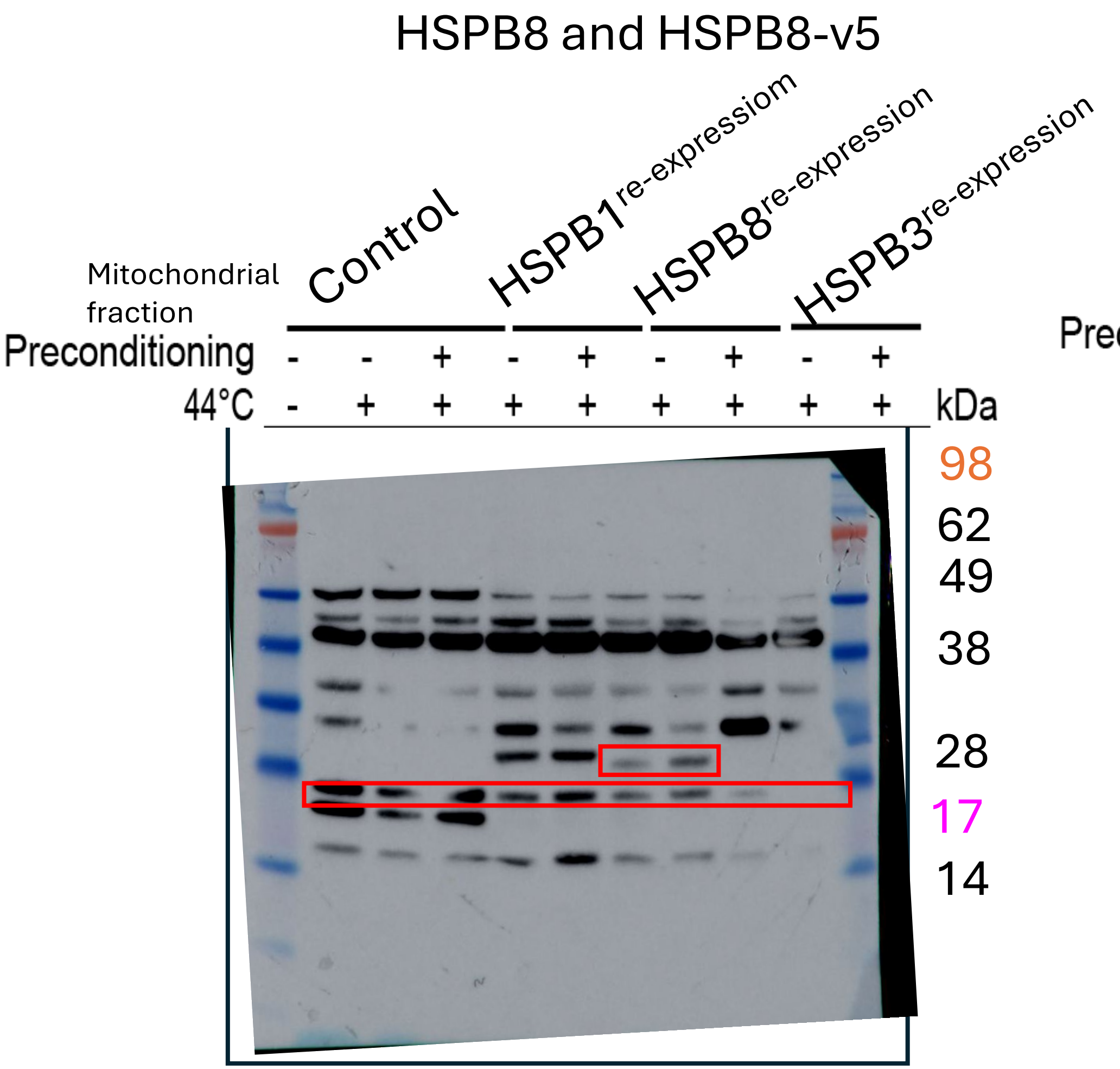

Bis-Tris 4-12% Gel
MES Buffer

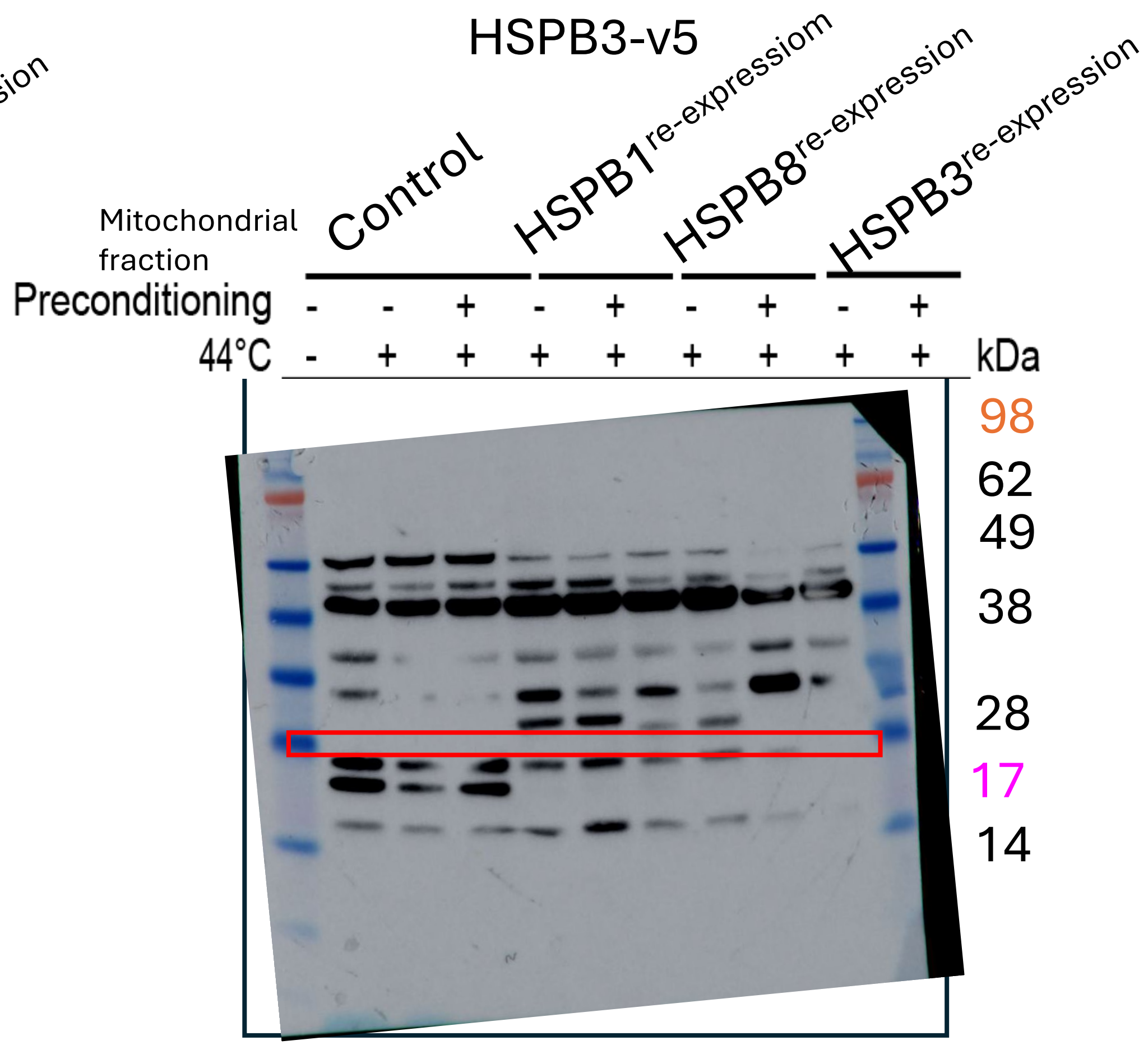

Bis-Tris 4-12% Gel
MES Buffer

Supplementary Figure S8

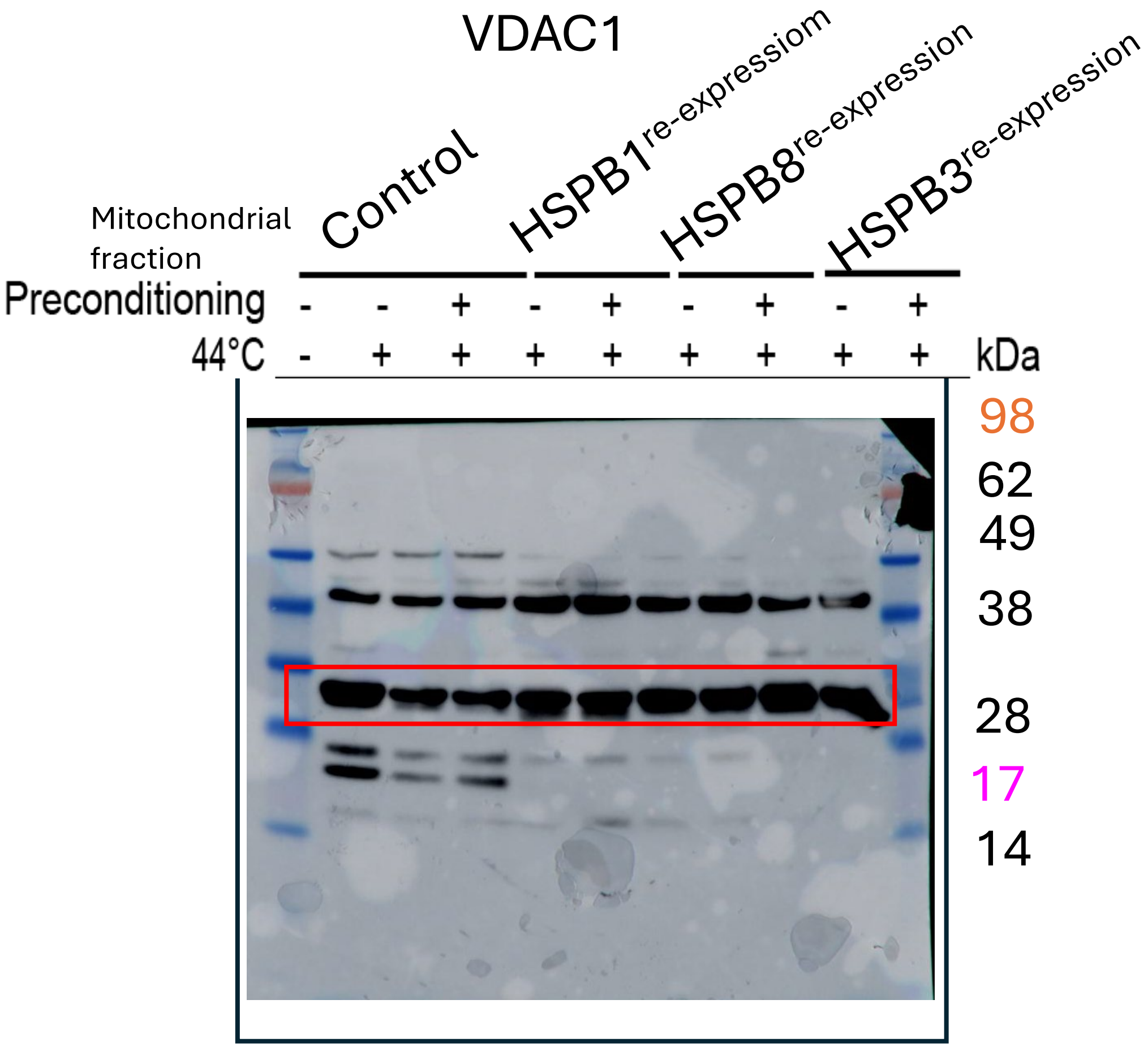

Bis-Tris 4-12% Gel
MES Buffer
